# Integrated Genomic and Epigenomic Analysis of Breast Cancer Brain Metastasis

**DOI:** 10.1371/journal.pone.0085448

**Published:** 2014-01-29

**Authors:** Bodour Salhia, Jeff Kiefer, Julianna T. D. Ross, Raghu Metapally, Rae Anne Martinez, Kyle N. Johnson, Danielle M. DiPerna, Kimberly M. Paquette, Sungwon Jung, Sara Nasser, Garrick Wallstrom, Waibhav Tembe, Angela Baker, John Carpten, Jim Resau, Timothy Ryken, Zita Sibenaller, Emanuel F. Petricoin, Lance A. Liotta, Ramesh K. Ramanathan, Michael E. Berens, Nhan L. Tran

**Affiliations:** 1 Integrated Cancer Genomics Division, Translational Genomics Research Institute, Phoenix, Arizona, United States of America; 2 Collaborative Center for Bioinformatics, Translational Genomics Research Institute, Phoenix, Arizona, United States of America; 3 Cancer and Cell Biology Division, Translational Genomics Research Institute, Phoenix, Arizona, United States of America; 4 Neurogenomics Division, Translational Genomics Research Institute, Phoenix, Arizona, United States of America; 5 Center for Personalized Diagnostics, Biodesign Institute, Arizona State University, Tempe, Arizona, United States of America; 6 The Van Andel Research Institute, Grand Rapids, Michigan, United States of America; 7 Iowa Spine and Brain Institute, Iowa City, Iowa, United States of America; 8 Department of Radiation Biology, University of Iowa, Iowa City, Iowa, United States of America; 9 Center for Applied Proteomics and Molecular Medicine, George Mason University, Manassas, Virginia, United States of America; 10 Virginia G. Piper Cancer Center, Scottsdale Healthcare, Scottsdale, Arizona, United States of America; Health Canada and University of Ottawa, Canada

## Abstract

The brain is a common site of metastatic disease in patients with breast cancer, which has few therapeutic options and dismal outcomes. The purpose of our study was to identify common and rare events that underlie breast cancer brain metastasis. We performed deep genomic profiling, which integrated gene copy number, gene expression and DNA methylation datasets on a collection of breast brain metastases. We identified frequent large chromosomal gains in 1q, 5p, 8q, 11q, and 20q and frequent broad-level deletions involving 8p, 17p, 21p and Xq. Frequently amplified and overexpressed genes included ATAD2, BRAF, DERL1, DNMTRB and NEK2A. The ATM, CRYAB and HSPB2 genes were commonly deleted and underexpressed. Knowledge mining revealed enrichment in cell cycle and G2/M transition pathways, which contained AURKA, AURKB and FOXM1. Using the PAM50 breast cancer intrinsic classifier, Luminal B, Her2+/ER negative, and basal-like tumors were identified as the most commonly represented breast cancer subtypes in our brain metastasis cohort. While overall methylation levels were increased in breast cancer brain metastasis, basal-like brain metastases were associated with significantly lower levels of methylation. Integrating DNA methylation data with gene expression revealed defects in cell migration and adhesion due to hypermethylation and downregulation of PENK, EDN3, and ITGAM. Hypomethylation and upregulation of KRT8 likely affects adhesion and permeability. Genomic and epigenomic profiling of breast brain metastasis has provided insight into the somatic events underlying this disease, which have potential in forming the basis of future therapeutic strategies.

## Introduction

Brain metastasis is the most common intracranial tumor, occurring in 15–40% of all cancer patients with metastatic disease [1], [2], [3]. The incidence of brain metastasis has increased in recent years, possibly due to prolonged survival of cancer patients receiving aggressive treatments for their primary or systemic disease [1], [2], [3]. Given their overall frequency in the population, lung and breast cancer are by far the most common tumors to develop brain metastases [1], [2], [3].

Epidemiological studies suggest that brain metastases occur with a frequency of approximately 10–16% in patients with breast cancer, although large autopsy studies indicate that frequencies may be as high as 18–30% [2], [3], [4], [5]. Brain metastases occur rapidly, usually within 2–3 years following diagnosis of systemic metastatic disease, and the median survival once there is brain involvement is a stifling 13 months with fewer than 2% of patients surviving greater than 2 years. Breast cancer involving the brain (parenchyma or leptomeninges) is considered a feature of late-stage progressive disease for which few effective treatments exist. Due to limitations imposed by the blood brain barrier (BBB), chemotherapy has not generally been used to treat most epithelial cancers that metastasize to the brain. Whole brain radiation can provide a survival benefit of 4–5 months, which can be further extended with stereotactic radiosurgery (SRS). Surgery can also lead to dramatic improvements in survival if fewer than three metastases exist and all are treated aggressively with surgery or SRS.

Currently there are few predictive measures for identification of patients at risk for developing brain metastasis from their primary cancer. In general, the development of brain metastases from breast cancer depends on several prognostic factors, including younger age, ethnicity, hormone receptor negative status, presence of BRCA1 germ-line mutations, and the expression of the epidermal growth factor receptor 2 (Her2/neu) proto-oncogene, all of which contribute to an increased rate of brain metastasis [2].

The overall goal of our study was to utilize array-based technologies to assemble a compendium of genomic and epigenomic events in a series of breast cancer brain metastases to understand the landscape of breast cancer brain metastatic lesions. The compendium would be interrogated for common and uncommon abnormalities in order to identify potential new therapeutic targets to control this fatal manifestation of breast cancer.

## Materials and Methods

### Sample Acquisition

Retrospective fresh-frozen samples of breast brain metastases (BBM) were obtained from The University of Toronto Nervous System Tissue Bank, University Health Network, Toronto, Canada (n = 23) and from The Department of Neurosurgery's brain tumor bank, University of Iowa Medical Center, Iowa City, Iowa (n = 12). Non-neoplastic brain samples were also obtained from the University of Toronto Tissue Bank (n = 2) and from The Department of Neurosurgery, University of Iowa Medical Center (n = 8). Ten non-neoplastic breast tissue specimens were purchased from Asterand (Detroit, MI). A series of 50 early-stage (grade 1 and 2) breast cancer specimens were obtained from the Manitoba Tumor Bank, Winnipeg, Manitoba. All samples were obtained under appropriate ethical procedures and informed patient consent at the respective institutions.

All human biospecimens used in this study were pre-existing and de-identified before shipment to the Translational Genomics Research Institute (TGen) for genomic analysis. TGen investigators did not have access to patient identifiers at any time before or after completion of the study. TGen investigators and the holder of patient identifiers entered into an agreement prohibiting the release of this information to TGen investigators under any circumstances. Therefore, the biospecimens do not qualify as human subjects and the study is exempt from Institutional Review Board, in accordance with the Office of Human Research Protections (OHRPs) Guidance on Research Involving Coded Private Information or Biological Specimens.

### gDNA and RNA Isolation

Genomic DNA (gDNA) was isolated from fresh-frozen tissue using the DNeasy Blood and Tissue Kit (Qiagen, Valencia, CA) with the following modifications. Approximately 25 mg frozen tissue was pulverized after a brief incubation in liquid nitrogen, then lysed in 180 µL ATL buffer. The sample was further disrupted using a hand-held tissue homogenizer (VWR, Radnor, PA) before adding 20 µL proteinase K solution. Lysates were incubated at 56°C for 72 hours. Following proteinase K treatment, lysates were centrifuged at 17,000× *g* to pellet particulate material. Genomic DNA was eluted in 100 µL T low E buffer (Teknova, Hollister, CA) and stored at 4°C. Total RNA, including small RNA, was isolated using the mirVana miRNA Isolation Kit (Ambion, Austin, TX) following the manufacturer's protocol and stored at −80°C. Genomic DNA and total RNA yields and purity were assessed using a NanoDrop 2000c (Thermo Scientific, Waltham, MA). Genomic DNA integrity was confirmed by agarose gel electrophoresis. Total RNA samples were evaluated for integrity using the Bioanalyzer RNA 6000 Nano LabChip Kit (Agilent Technologies, Santa Clara, CA) on a Bioanalyzer 2400 (Agilent Technologies). Only total RNA samples with RNA integrity number values of at least 7 (RIN≥7) were profiled. A total of 10 samples were dropped due to RIN values lower than 7.

### Copy Number Analysis

Array-based comparative genomic hybridization (aCGH) was performed on 19 BBM samples using the Agilent SurePrint G3 Human CGH Microarray Kit, 1×1M, which have an average probe spacing of 2.1 Kb (Agilent Technologies). Briefly, 800 ng of experimental and normal female reference (Promega, Madison, WI) gDNA were independently digested with Bovine DNAse I (Ambion) and directly labeled with Cy5-dUTP and Cy3-dUTP, respectively, using the BioPrime Array CGH Genomic Labeling Module (Invitrogen, Carlsbad, CA). Labeled DNA was purified using Vivaspin 500 columns (Satorius Stedim Biotech, Goettingen, Germany). Equal amounts of labeled, purified experimental and reference DNA were hybridized to the microarray in a rotary oven at 65°C for 40 hr at a rotation speed of 20 rpm. The slides were washed according to manufacturer's protocol and images were captured using an Agilent DNA microarray scanner set at default settings for array-based comparative genomic hybridization. Scanner images were extracted using Feature Extraction software v.10.5.1.1 (Agilent Technologies). Log_2_ data was imported into Agilent DNA Analytics 4.0.81 software for visualization and quality assessment. The aCGH data for 15 of 19 BBM samples, which passed quality control metrics, were segmented using the circular binary segmentation (CBS) algorithm [6], [7]. Genomic Identification of Significant Targets in Cancer (GISTIC) was then used to identify regions of the genome that were significantly amplified or deleted across the 15 breast brain metastasis samples. GISTIC calculated a statistic (G-score) for the frequency of occurrence and the amplitude of the aberration. The statistical significance of each aberration was computed by comparing the observed G-score to the results expected by chance. Regions with false-discovery rate (FDR) q-values less than 0.25 were considered statistically significant. In addition, copy number variation analysis was performed using Agilent's Genomics Workbench 6.5 software. The Aberration Detection Method 2 (ADM-2) algorithm was used to flag altered chromosomal regions and breakpoints (ADM-2 threshold of 5.5 within a 5.0 Mb window size containing at least 3 probes and with minimum 0.58 absolute average log ratio for the region).

### mRNA Expression Profiling

RNA from 35 BBM, 10 non-neoplastic brain (NBn) and 10 non-neoplastic breast (NBr) tissues were profiled using Agilent whole human genome 4×44K mRNA expression microarrays. A quick-amplification kit (Agilent Technologies) was used to amplify and label 500 ng target mRNA species into complementary RNA (cRNA) for oligo microarrays according to the manufacturer's protocol. For each two-color array, a commercial universal reference RNA (Stratagene, La Jolla, CA) was labeled with cyanine 5-CTP and cyanine-3-CTP (Perkin Elmer, Boston, MA). Complimentary RNA concentration and labeling efficiency were measured spectrophotometrically. Approximately 800 ng of both Cy5-labeled experimental cRNA and Cy3-labeled universal reference RNA were hybridized to each microarray (adjusting for labeling efficiency). Images were captured using an Agilent DNA microarray scanner set at default settings for gene expression. Scanned images were processed using Feature Extractor v. 10.5.1.1software by applying a LOWESS (locally weighted linear regression) correction for dye bias and background noise was subtracted from spot intensities. To filter the preprocessed data, genes with a background signal higher than feature signal were removed.

### Intrinsic Subtype Classification of Breast Brain Metastasis

The PAM50 gene expression classifier is a supervised, centroid-based prediction method to classify breast cancers into intrinsic molecular subtypes (Luminal A, Luminal B, HER2-enriched, basal-like, and normal-like) using a 50-gene signature. We applied this classifier to samples analyzed on the Agilent 4×44K mRNA expression platform. Normal samples were used as controls. The log ratio values of the probes were collapsed to gene level by taking the median of all probes matching to same gene.

### DNA Methylation Analysis

A total of 1 µg of DNA from 32 BBM, 12 NBr, 15 NBn samples and 48 early-stage primary breast cancer samples was bisulfite converted with the EZ DNA methylation kit (Zymo Research, Irvine, CA) and subsequently processed for hybridization onto the Infinium HumanMethylation27 BeadArray (Illumina, San Diego, CA) according to manufacturers' protocols. This array interrogates 27,578 CpG dinucleotides encompassing 14,495 genes. Bisulfite-treated DNA was subsequently amplified, fragmented and hybridized to locus-specific oligonucleotides on the BeadArray. Image processing and intensity data extraction were performed using an Illumina BeadArray Reader. The GenomeStudio Methylation software from Illumina was used for data assembly and acquisition. Methylated (M) and unmethylated (U) alleles were detected by fluorescence signal from single-nucleotide extension of the DNA fragments. Results were interpreted as a methylation ratio (β-value) of methylated signal (M) to the sum of methylated and unmethylated signal (M+U) for each locus. The average β value reports a methylation signal ranging from 0 to 1 spanning completely unmethylated to completely methylated, respectively. A differentially methylated locus was defined by having a statistically significant (*p*-value≤0.05 after computing a Mann Whitney non-parametric test) average β difference of at least |0.2| between groups.

### Data Deposition in Public Portals

The raw Agilent gene expression array data discussed in this publication have been deposited in NCBI's Gene Expression Omnibus and are accessible through GEO Series accession number GSE52604 (http://www.ncbi.nlm.nih.gov/geo/query/acc.cgi?acc=GSE52604). The aCGH (http://dx.doi.org/10.6084/m9.figshare.862978) and DNA methylation (http://dx.doi.org/10.6084/m9.figshare.855629) data are available online at Figshare.com.

### Pathway Analysis

Gene lists of interest were uploaded into IPA (Ingenuity® Systems, Redwood City, CA) and the Core Analysis workflow was run with default parameters. The Core Analysis provides an assessment of significantly altered pathways, molecular networks and biological processes represented in the samples' gene list.

### Quantitative Reverse-Transcriptase (RT)-PCR and Copy Number PCR Assays

Complimentary (cDNA) was synthesized using 100 ng of total RNA in a 20 µl reaction volume. The Superscript® III First Strand synthesis system (Life Technologies, Carlsbad, CA) was used with the following conditions: 10 minutes at 25°C, 30 minutes at 50°C, 5 minutes at 85°C and 20 minutes at 37°C with RNase H. SYBR green fluorescence was used for the detection of amplification after each cycle using the LightCycler 480 SYBR Green I Mastermix (Roche Applied Science, Indianapolis, IN). Quantitative PCR (qPCR) was subsequently performed on cDNA in a final volume of 25 µl using the LightCycler 480 instrument (Roche Applied Science). The qPCR cycling conditions were as follows: 5 minutes at 95°C for activation of Platinum® Taq DNA polymerase, 10 seconds at 95°C, 20 seconds at 59°C, and 30 seconds at 72°C for 45 cycles. Quantification was based on the number of cycles necessary to produce a detectable amount of product above background. The following primer pairs were used:

AURKB: Forward- 5′-ATTGCTGACTTCGGCTGG T-3′, Reverse: 5′-GTCCAGGGTGCCACA CAT-3′; FOXM1: forward: 5′-TGGCGATCTGCGAGATTT-3, Reverse: 5′- CCTCCTCAGCTA GCAGCACT-3′; ATAD2-forward: 5′-CCTGCAAGACCAAGATACCG-3′, Reverse: 5′-TTTCCTCCGCCTCTCAAAGT-3′; cMYC: forward: 5′-CTTCTCTCCGTCCTCGGATTCT-3′, reverse: 5′-GAAGGTGATCCAGACTCTGACCTT-3′; The β-actin, Histone and GAPDH genes were used as an internal reference control:

Histone: forward: 5′-CCACTGAACTTCTGATTCGC-3′, and reverse: 5′-GCGTGCTAGCTGGATGTCTT-3′; GAPDH: forward: 5′-CTGCACCACCAACTGCTTAG-3′ and reverse: 5′-GTCTTCTGGGTGGCAGTGAT-3′.

For each sample, the delta C_t_ value was calculated as the difference between the target gene C_t_ value and the C_t_ value of the geometric mean of the internal reference controls. The quantity of expression was calculated relative to the average of expression obtained from NBr and NBn samples (n = 6). The equation used for relative fold-change was 2^−ΔΔCT^.

Copy number validation was completed with the qBiomarker Copy Number PCR Assays (Qiagen) for ATAD2 (Assay ID 28855976), and cMYC (Assay ID 28877687). Samples were analyzed with the qBiomarker SYBR ROX Mastermix (Qiagen). A multi-copy reference assay, the qBiomarker Multicopy Reference Copy Number PCR Assay (MRef, Assay ID 30773761) was performed for each sample and served as the internal reference control. Data were analyzed with qBiomarker Copy Number PCR Assay Data Analysis Software.

All PCR reactions were run in triplicate, and melting curve analysis was performed to ensure specificity of the PCR product. Negative (no template) controls were run in parallel to confirm the absence of nonspecific fluorescence in samples.

## Results

### Copy Number Analysis

**Figure 1:**
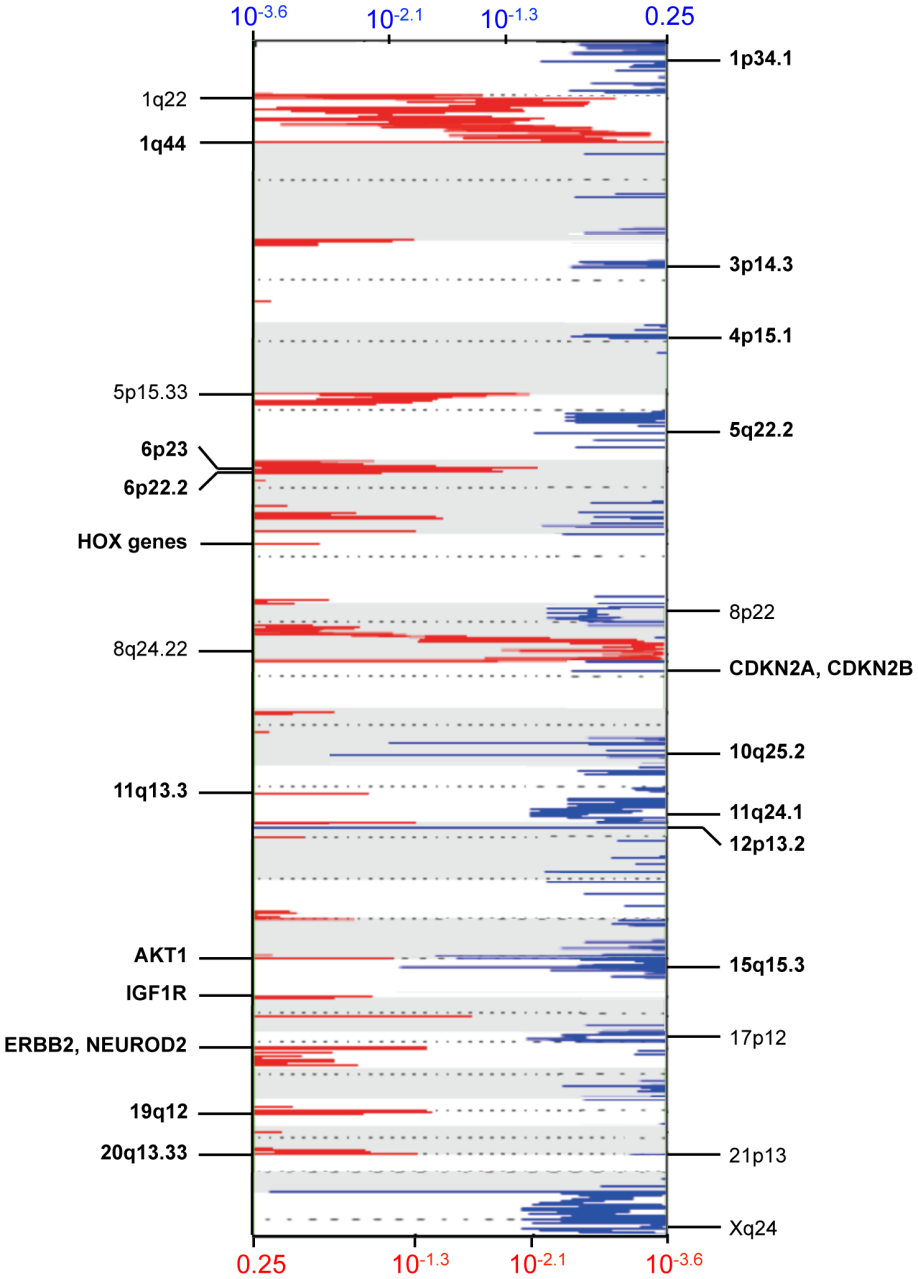
DNA Copy Number Analysis of Breast Brain Metastasis. GISTIC analysis was conducted on Agilent SurePrint G3 Human CGH Microarray data for 15 breast brain metastases. Significant false discovery rates (Q-values) for amplified (red) and deleted (blue) regions are plotted genome-wide. Annotations for a few of the significant regions are shown. Focal amplifications and deletions are annotated in boldface, and broad amplifications and deletions are annotated in non-boldface. Q-values for deleted and amplified genes are displayed along the x-axis on top and bottom of the figure, respectively.

Somatic copy number analysis (SCNAs) was conducted using the one-million feature aCGH platform in 15 breast brain metastases genomes to identify regions of gain or loss. GISTIC analysis identified 18 focal amplifications (**Figure 1**) and 4 regions of broad amplifications involving 1q, 5p, 8q, 11q, and 20q (**Figure 1**). Among the genes amplified in the focal regions were a cluster of HOX genes (HOXA7, HOXA9, HOXA10, HOXA11) on 7p15, AKT1 (14q32.33), IGF1R (15q26.3), ERBB2 and NEUROD2 (17q12), all of which have been reported in primary breast cancers (**Table S1a, File S1**). GISTIC also identified 37 focal deletions involving CDKN2A, CDKN2B, and DMRTA1 (**Figure 1**, **Table S1b, File S1**). Four regions of broad deletions included 8p, 17p, 21p and Xq (**Figure 1**).

### mRNA Expression

**Figure 2:**
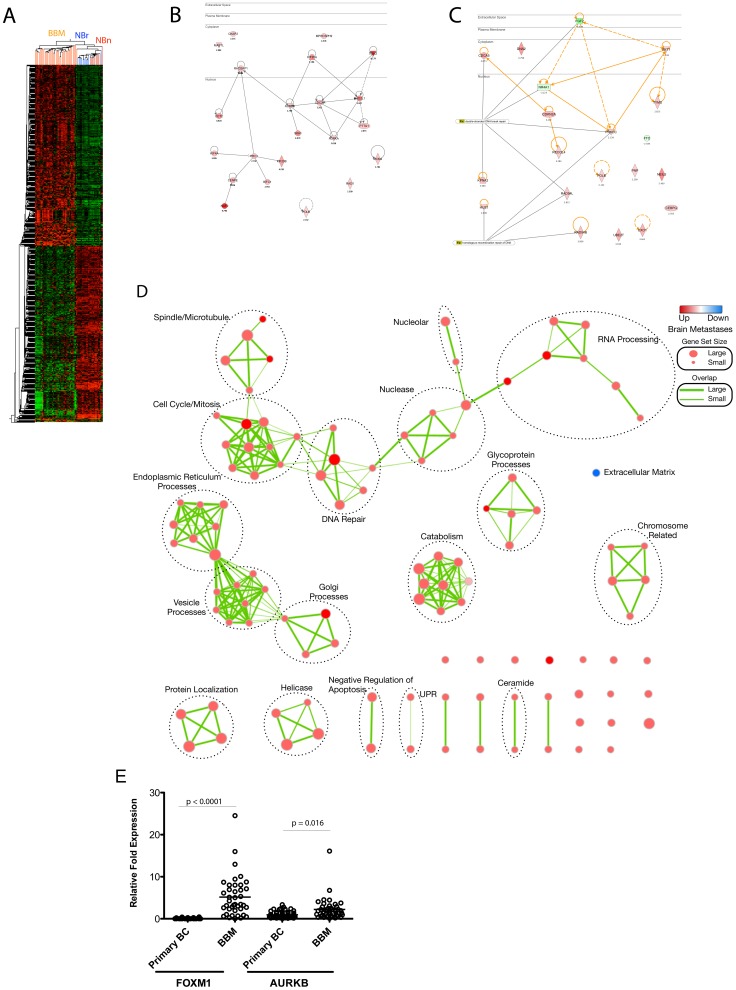
Analysis of Differentially Expressed Genes in Breast Brain Metastasis. A) Hierarchical clustering of 863 genes distinguishing breast brain metastases (BBM, orange ticks) from non-neoplastic breast (NBr, red ticks) and non-neoplastic brain tissue (NBn, blue ticks); B) Cell Cycle Gene Network. Genes that mapped to the ‘Cell Cycle’ categories were used to construct a direct interaction network; C) DNA Repair Network. Genes that mapped to the ‘DNA Repair’ category were used to construct a direct interaction network. Two DNA repair processes are highlighted and connected to genes annotated to those processes. For Figure 2B and 2C, the gene nodes are shaded red in proportion to the degree of upregulation. Gene nodes shaded in green are downregulated. Log_2_ ratios are listed under the individual nodes. Direct physical interaction relationships are represented by solid lines. Dotted lines represent indirect physical interactions; D) GSEA Enrichment Map. The results from the GSEA analysis comparing 35 breast cancer brain metastases to 10 non-neoplastic brain and 10 non-neoplastic breast were visualized using Cytoscape and Enrichment Map plug-in. The significant gene sets from the C5 gene ontology library are represented with a *p*-value of ≤0.05 and false discovery rate (Q) of <0.5. Each individual node represents one gene set with the size of node proportional to number of genes in the set and color intensity relates to degree of enrichment (red = up in tumor; blue = down in tumor). The relative overlap of the number of genes shared by individual nodes is represented by the thickness of the connecting edges. Interesting subgroups in the network are circled and manually annotated; E) Vertical scatter plots showing FOXM1 and AURKB overexpression by qRT-PCR in breast brain metastasis (BBM, n = 42) compared to early-stage primary breast tumors (n = 50). Fold expression was relative to expression in non-neoplastic breast or brain samples (n = 10).

Gene expression profiling was performed using Agilent whole human genome 4×44K mRNA expression microarrays to identify differentially expressed genes (DEG) in BBM samples and an independent set of non-neoplastic hyperplastic breast samples (NBr) and non-neoplastic brain samples (NBn). DEG were selected if differential expression was evident between tumor samples and NBn; between tumor samples and NBr but not between NBn and NBr. In addition, DEG were selected based on *p*-values≤0.05 and a fold-change ≥2 or≤−2. This comparison identified 863 differentially expressed genes (**Table S2, File S1**). A heatmap was generated in GeneSpring v12.1 to visualize the DEG list, which also shows a clear separation of tumor and non-neoplastic samples (**Figure 2A**). In order to identify biological concepts altered in the differentially regulated BBM gene list we submitted the gene list to IPA (Ingenuity® Systems) and applied the Core Analysis workflow. The functional analysis portion of the workflow identified biological functions and/or diseases most significantly altered in our DEG list. Significant categories were sorted based on activation/inhibition z-scores to identify the most significant distinguishing categories with respect to up-regulated and down-regulated genes. Fourteen specific functions had increased activation states as evidenced by z-scores ≥2 (**Table S3a, File S1**). Five of the fourteen functional annotations mapped to the ‘Cell Cycle’ category. The genes defining the five categories and their relationships were visualized as a network in (**Figure 2B**). ‘DNA repair’ was another noteworthy enriched category. The genes that mapped to this category were also visualized as a network (**Figure 2C**). Enrichment analysis was performed to annotate further the genes in this category. Two DNA repair processes, ‘double-stranded DNA break repair’ (CDCA5, FGF2, NR4A1, PRKDC, RAD54L, KPNA2) and ‘homologous recombination repair’ (HUS1, PRKDC, RAD54L and RAD54B) were identified and highlighted in **Figure 2C**. The functional annotation categories for down-regulated genes converged mostly on categories associated with ‘tissue morphology’ and ‘development’ (**Table S3b, File S1**).

To identify potential transcriptional regulators among the DEG we used the results of the upstream regulator analysis. This part of the Core Analysis workflow connects transcriptional regulators in the IPA database to the differentially expressed genes. Over-connected regulators are scored with a *p*-value, for gene enrichment, and z-score for degree of activation based on direction of regulation in database and concordance with direction of regulation in DEG. The list of upstream regulators was filtered for ‘transcription regulators’ upregulated (z-score ≥2) and downregulated (z-score≤2). Five specific transcription factors were identified as active and six were identified as inhibited (**Table S4a, File S1**). One of the inhibited transcription factors was TP53, implying that TP53 signaling is defective in BBM. Among the activated transcription factors, IRF1 and IRF7 seemed to be connected preferentially to genes involved in immune response such as ‘antiviral response’ and ‘antimicrobial response.’ This may indicate a possible infiltration of immune cells in the samples or could reflect an immunogenic response by the tumor cells. Two interesting transcriptional regulators highly scored were FOXM1 and TBX2 that had three commonly regulated genes. We constructed a combined network illustrating the downstream transcriptional functional targets for both transcription factors (**Figure S1, File S1**). A functional enrichment was performed on the resultant network indicating that the these two transcription factors control the expression of genes enriched for processes such as ‘cell cycle progression’ (*p*-value 4.1E-15), mitosis (*p*-value 4.97E-13) and ‘cytokinesis’ (*p*-value 1.98E-10, **Table S4b, File S1**). This observation, coupled with the above functional enrichment on the whole gene list suggests that breast cancer brain metastasis gene expression is associated with cell cycle/mitosis and may be driven by FOXM1 and TBX2.

Next, we used gene set enrichment analysis (GSEA) to identify sets of genes that are coordinately regulated in the BBM samples. The GSEA algorithm identifies gene sets enriched at the top (breast brain metastases) or bottom (non-neoplastic samples) of the ranked list of DEG. We conducted the GSEA analysis using only the c5 gene set library (GSEA | MSigDB), which contains only gene ontology gene sets. There were 109 gene sets significant at a FDR<50% and *p*-value of ≤0.05 that were upregulated in the breast brain metastases and only one gene set downregulated (full GSEA results can be found in **Table S5a–b, File S1**). We visualized the above-referenced gene sets using the Enrichment Map plug-in for cytoscape [8]. The enrichment map portrays the GSEA results as a network of gene sets (nodes) connected by edges representing overlapping genes. The enrichment map improves interpretation of GSEA results by allowing for the identification of functional groupings of the enriched gene sets. We manually inspected the resultant clusters and assigned summary labels to individual subnetworks of interest. Some interesting subnetworks enriched in the BBM samples include ‘Cell Cycle/Mitosis’, ‘DNA repair’, ‘Vesicle Processes’, ‘Protein Localization’ and ‘RNA processing’. The only category associated with the non-neoplastic samples was extracellular matrix (**Figure 2D**). We experimentally validated the expression of FOXM1 and AURKB in 42 breast brain metastasis samples (which included those used in the expression arrays) and in a series of 50 primary breast cancer samples by qRT-PCR. Both FOXM1 and AURKB were significantly upregulated in brain metastasis samples compared to primary breast cancer samples and non-neoplastic samples (**Figure 2E**).

### Breast Cancer Intrinsic Subtype Analysis of Breast Brain Metastasis

**Figure 3:**
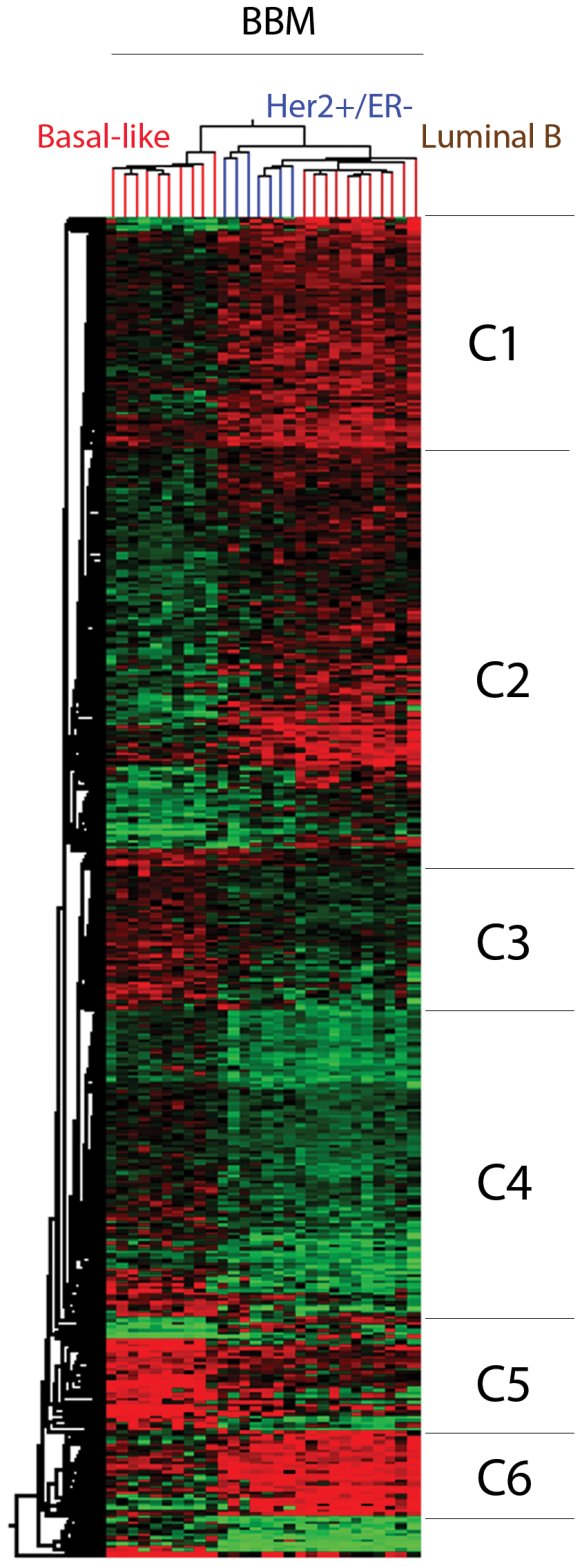
Hierarchical Clustering of Genes that Differentiated Brain Metastases Based on Their Breast Cancer Intrinsic Subtype Status. Six distinct clusters were identified and their respective genes further analyzed.

We used the PAM50 gene expression classifier to divide the breast brain metastatic samples into the common intrinsic subtypes known for breast cancer. From this analysis we identified the following subtypes in our sample cohort: 2 (5.7%) Luminal A, 12 (34.2%) Luminal B, 8 (22.8%) Her2+/ER−, 11(31.4%) basal-like, and 2 (5.7%) Normal-like tumors. An unsupervised clustering analysis of the 863 DEG failed to discriminate between the different subtypes (**Figure 2A**). Therefore, we performed an analysis of variance (ANOVA) to identify DEG between Luminal B, Her2+/ER− and basal-like tumors. A Post-hoc Tukey test and a Benjamini Hochberg multiple correction test were applied to the data. Differences with fold-change <2 were excluded. We excluded tumors classified as Luminal A and normal-like due to a very small sample size. There were 733 DEG between Luminal B and basal-like tumors; 492 DEG between Her2+/ER− and basal-like and 223 DEG between Luminal B and Her2+/ER− (**Table S6a–b, File S1**). The union of the differentially expressed genes between groups consisted of 774 unique genes (**Table S7, File S1**) or 886 probesets. Hierarchical clustering using this gene list was able to clearly distinguish the subtypes and six distinct gene clusters were identified (**Figure 3**, **Table S8a–f, File S1**).

The genes in each cluster were analyzed using ToppGene suite and data are presented in tables and as summary word clouds for enriched processes and signatures (**Table S9a–f, Figure S2, File S1**). Cluster 1, 2 and 6 contained genes upregulated in both the Her2+/ER− and Luminal B subtypes. However, expression was generally highest in the Luminal B brain metastases. Enrichment analysis of genes in clusters 1, 2 and 6 using the ToppFun module of the ToppGene suite reveals genes previously associated with breast cancer signatures [9]. Specifically, those signatures were associated with luminal subtypes and estrogen receptor(ER)-positive breast cancer. Additionally, cluster 1 has overexpression of RET, and ERBB3, which represent possible actionable therapeutic targets. Cluster 2, similar to clusters 1 and 6, contained genes also upregulated across the Her2+/ER− and Luminal B samples. This cluster contained GATA3, which is an ESR1 target gene, but was only expressed in the Luminal B tumors. Of note, in cluster 6, ESR1 was most highly expressed in Luminal B tumors, coincidentally with AR. Basal-like tumors had a negative expression value for ESR1 and AR. We also note highest expression of FLT3 and FOXA1 genes in Luminal B tumors. Expressed highly in both Her2+/ER− and Luminal tumors were the TFF3 and LRRC6. Genes in cluster 3 and 5 were, in general, were preferentially upregulated in the basal-like samples. The gene lists were consistent with known basal-like breast cancer genes and are also known to be lowly expressed in Luminal breast cancer. For example, Keratin 5, 6 and 14, as well as, CDH3 were present in cluster 5. In addition, FOXC1 and CHST3 in cluster 3 and UGT8, and CHODL genes in cluster 5 were among the highly expressed genes in basal-like tumors. Cluster 4 is unique in that it has a number of genes downregulated across the three subtypes, however, there was a more pronounced downregulation in the Her2+/ER− and Luminal B subtypes. Interestingly, this cluster contained a number of proliferation-associated genes, but they were relatively underexpressed. The basal cell marker gene, Keratin 17, is overexpressed in the basal samples across this cluster. WNT pathway members, FZD7 (C3), WNT6 (C4), WNT11 (C5) and FZD9 (4) were all preferentially expressed in basal-like tumors suggesting novel therapeutic opportunities for basal-like breast cancer brain metastases. Her2+/ER− tumors were associated with overexpression of TMEM45b, very reminiscent of a Her2 subtype. While these samples had some commonly expressed genes with the basal-like tumors, such as CLDN8, they were most similar to the Luminal B tumors. They also had the highest expression of TML5, CYP4F8, and PAX9 when compared to the other subtypes.

### DNA Methylation Analysis

**Figure 4:**
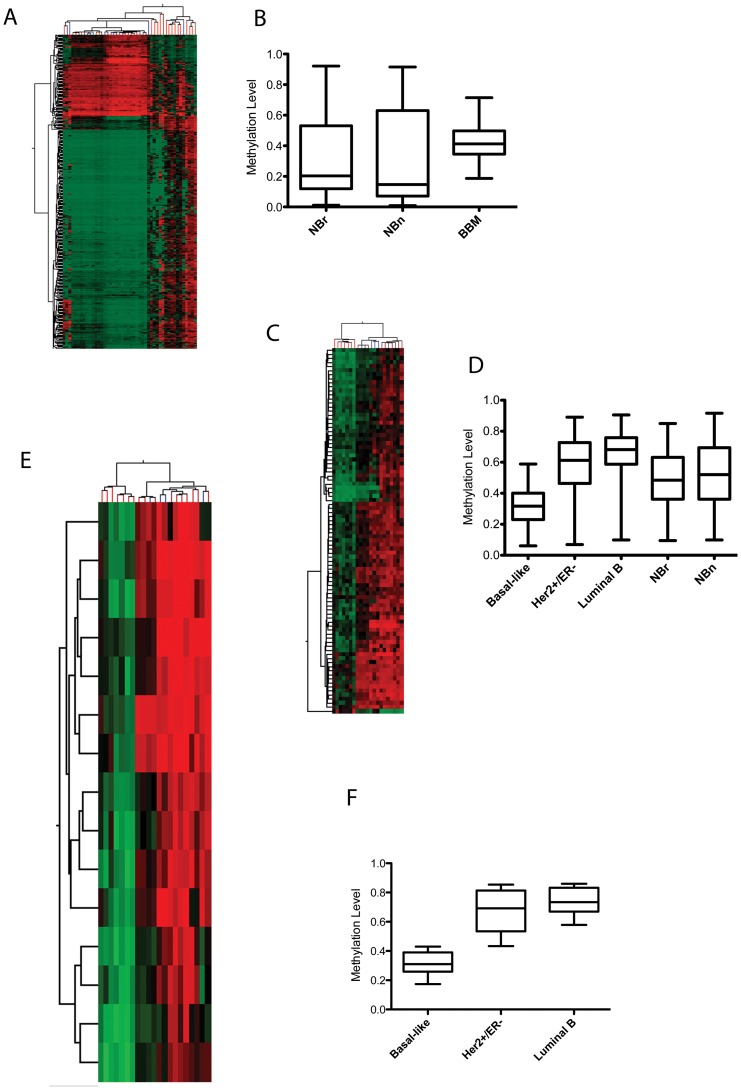
Differential Methylation Analysis in Breast Brain Metastases. A) Hierarchical clustering showing 425 tumor-specific differentially methylated loci (DML). Breast brain metastases (BBM, blue ticks) are distinguished from non-neoplastic breast (NBr) and non-neoplastic brain tissue (NBn, red ticks). B) Box plot demonstrating higher overall median methylation levels for 425 DML in BBM compared with NBr and NBn. C) Hierarchical clustering of 90 DML obtained by performing an ANOVA analysis and analyzing the union of genes between the different subtypes was able to distinguish BBM intrinsic subtype; D) Box plot representing the median methylation levels of 90 DML described above. NBr and NBn values are also shown. Basal-like breast brain metastases have overall lower methylation compared with the other groups. E) CpG Island Hypomethylator Phenotype (CIHMP) in basal-like brain metastases (red ticks) representing the 15 most hypo-methylated CpG Island loci when compared to Luminal B (maroon ticks) and Her2+/ER− tumors (blue ticks); F) Box plot graphing the methylation values of the 15 CpG loci most hypomethylated in basal-like breast brain metastases.

In order to identify alterations in DNA methylation we used the HumanMethylation27 BeadChip array. We compared BBM to NBn and NBr tissue and identified 425 differentially methylated loci (DML, **Figure 4A**, **Table S10, File S1**). The median methylation values were 0.4, 0.2 and 0.15 for breast brain metastasis, NBn and NBr respectively, indicating that breast brain metastasis was associated with hypermethylation (**Figure 4B**). Of the 425 loci, 117 were hypomethylated compared with non-neoplastic tissue and 308 were hypermethylated (**Table S10**). Only 23 of the hypo-methylated loci were associated with CpG islands, compared to 294 hyper-methylated loci, which occurred in CpG islands (**Table S10**). The 425 DML failed to discriminate between the molecular subtypes (**Figure 4A**), so we performed an ANOVA analysis to identify differentially methylated loci between the subtypes. A Post-hoc Tukey test and Benjamini Hochberg multiple correction were applied to the data. Differences with β values less than |0.2| were excluded. Due to small numbers of samples, we excluded tumors classified as Luminal A and normal-like. There were 95 DML between Luminal B and basal-like tumors; 71 DML between Her2+/ER− and basal-like tumors; and 13 DML between Luminal B and Her2+/ER− (**Table S11a–c, File S1**). The union of these loci resulted in 90 unique DML that discriminated between the subtypes (**Figure 4C**, **Table S11d, File S1**). When the union of these loci were examined, basal-like tumors had the lowest median methylation (0.32) compared with Her2+/ER− and Luminal B subtypes (0.61 and 0.68 respectively, **Figure 4D**), including non-neoplastic tissue examined. From these DML we identified a subset of 15 DML that were most hypomethylated in basal-like tumors compared to the other subtypes (**Figure 4E–F**, **Table S12, File S1**). This signature represents a potential CpG island hypomethylator phenotype (CIHMP) for basal-like breast brain metastasis and includes ALDH1A3, FANCG, TRIM29 and HOXA11 (**Table S12, File S1**).

### Combined aCGH and Gene Expression Analysis on Single Tumors

Next, we undertook a single-tumor-level analysis to identify a tumor specific, n = 1, investigation into altered biological concepts specific to single samples. A combined copy number and gene expression based analysis was conducted on 11 individual BBM samples. In brief, the ADM2 gene-level data were matched to gene-level mRNA expression data. Each sample was compared against non-neoplastic tissue as described above. Genes were filtered to include only those with a log_2_ ratio ≥2 or ≤−2. For copy number data, a filter was applied to the ADM2 log_2_ ratio to include only those genes with values ≥0.5 or ≤−0.8. The remaining data were then filtered for congruency to ensure consistency in direction of combined data, *i.e.*, genes needed to be amplified/overexpressed or deleted/underexpressed. The combined aCGH and mRNA expression lists for each sample were uploaded into MetaCore software (Thomson Reuters, New York, New York) for functional ontology enrichment, pathway mapping and knowledge mining. Each sample was interrogated with this workflow to identify biological concepts and observations which include single-gene alterations as well as pathway-based alterations. Expert review of data was conducted to identify and prioritize important biology and concepts for each sample (**Table S13, File S1**). Below we describe the top concepts identified in our samples.

We were able to identify at least one specific pathway/concept aberrantly operative in each sample. There were three samples that had alterations, which would predict interference with the ‘autophagy’ pathway. One sample had amplifications in two genes, eiF2AK3 and ATF6, which are crucial members of the ‘endoplasmic stress’ pathway. Multiple alterations were observed in one sample in the ‘WNT signaling’ pathway. Additionally, two samples had multiple alterations in the ‘chromosome condensation’ pathway. Lastly, six samples had amplifications and coupled overexpression of a histone gene cluster involving the genes HF3A and HF3B.

We also note a number of interesting genes (number of samples in parentheses) that were amplified and overexpressed: AKT1 (2), ATAD2 (7), AURKA (2), BRAF (3), DERL1 (6), DNMTRB (3), ESR2 (1), FASN (3), TNFRSF12A (2), PSENEN (4), HIF1A (2), IGF1R (1), NEK2A (6), MCL1 (1), PPFIA1 (1), RAF1 (2), PRL (1), RXRA (1), SRD5A2 (1), SUMO2 (1), TYMS (2), UBA1 (1), VEGFA (1), WNT3A (2) and WNT9A (2). Genes of interest (and number of samples) that were deleted and underexpressed were: CTNNA3 (2), ATM (4), TCF4(1), CDKN2A (1), CDKN2B (2), MSH6 (1), RB1 (1) and RPS6KA3 (2), CRYAB (4), HSPB2 (4).

**Figure 5:**
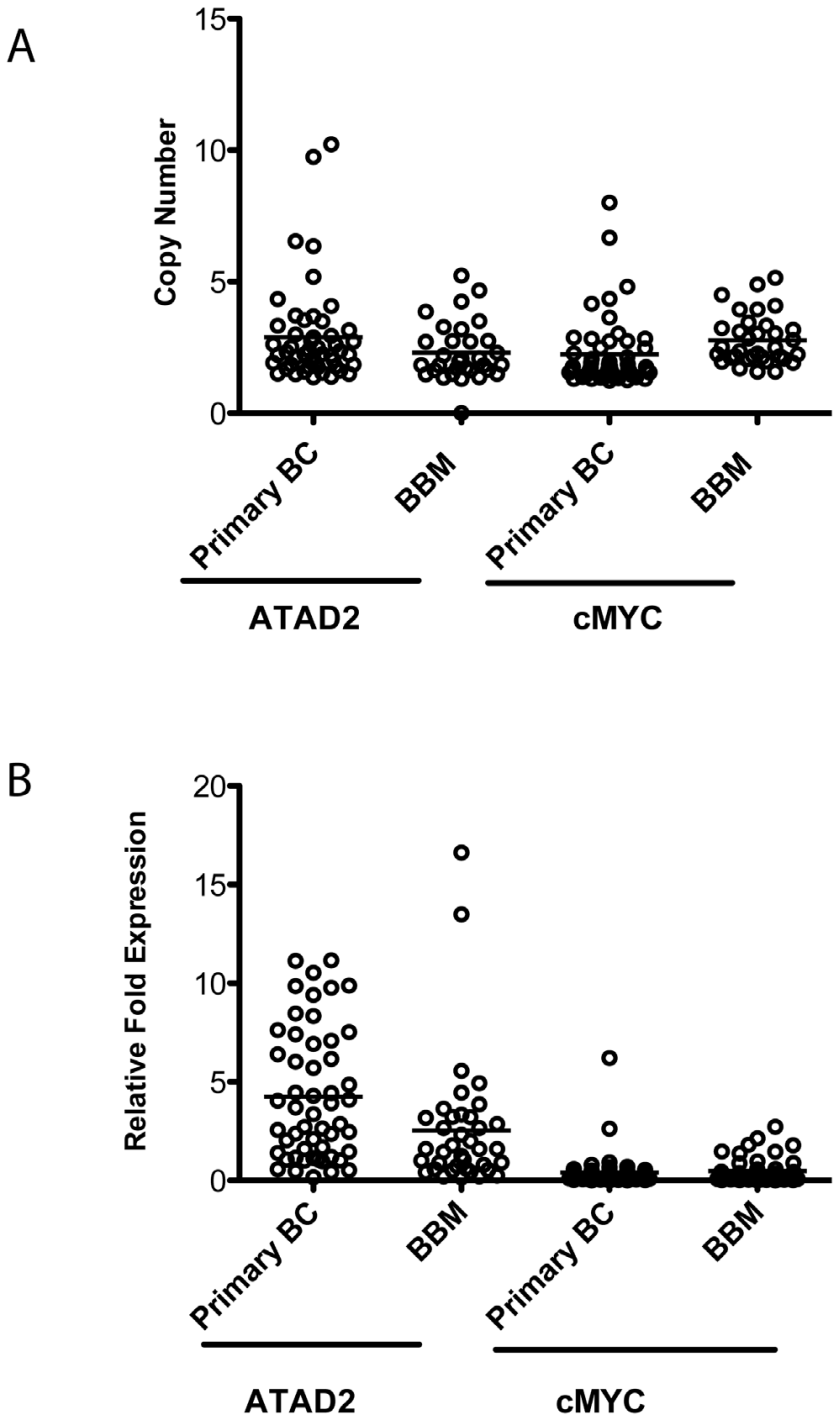
Validation of ATAD2 and cMYC Copy Number and Gene Expression in Breast Brain Metastasis (BBM, n = 42) and Early-Stage Primary Breast Cancer Samples (n = 50). A) Vertical scatter plots demonstrate a copy number gain of ATAD2 and cMYC by qPCR but with no difference between BBM and primary breast tumors. B) Vertical scatter plots show comparative overexpression of ATAD2 in both primary tumors and primary breast cancer, whereas cMYC is not expressed. None of the comparisons between primary tumors and BBM samples were statistically significant (*p*>0.05).

We selected the ATAD2 gene for copy number and gene expression validation by qRT-PCR and copy number qPCR assays due to its high frequency of alteration shown by aCGH and gene expression arrays. In addition, its position on 8q24, a known hotspot locus in breast cancer, further suggests the potential importance of this gene. Here we examined 42 breast brain metastases and 50 primary breast cancer samples. The BBM samples included all samples analyzed by aCGH and the gene expression array platform as well as additional samples. These data demonstrate that both metastatic and primary samples had a comparable increase in both copy number and expression of ATAD2 compared to non-neoplastic samples (**Figure 5**). The copy number data for the cMYC gene, which is also positioned on 8q24, demonstrate gene amplification in 10/15 samples by aCGH, but this was not accompanied by an increase in gene expression. Quantitative PCR for copy number determination in conjunction with qRT-PCR for expression analysis yielded similar findings where there was a noticeable, but similar cMYC amplification in both brain metastasis and primary breast cancer samples without any evidence of gene expression (**Figure 5**).

### Combined Gene Expression and DNA Methylation Analysis on Single Tumors

**Figure 6:**
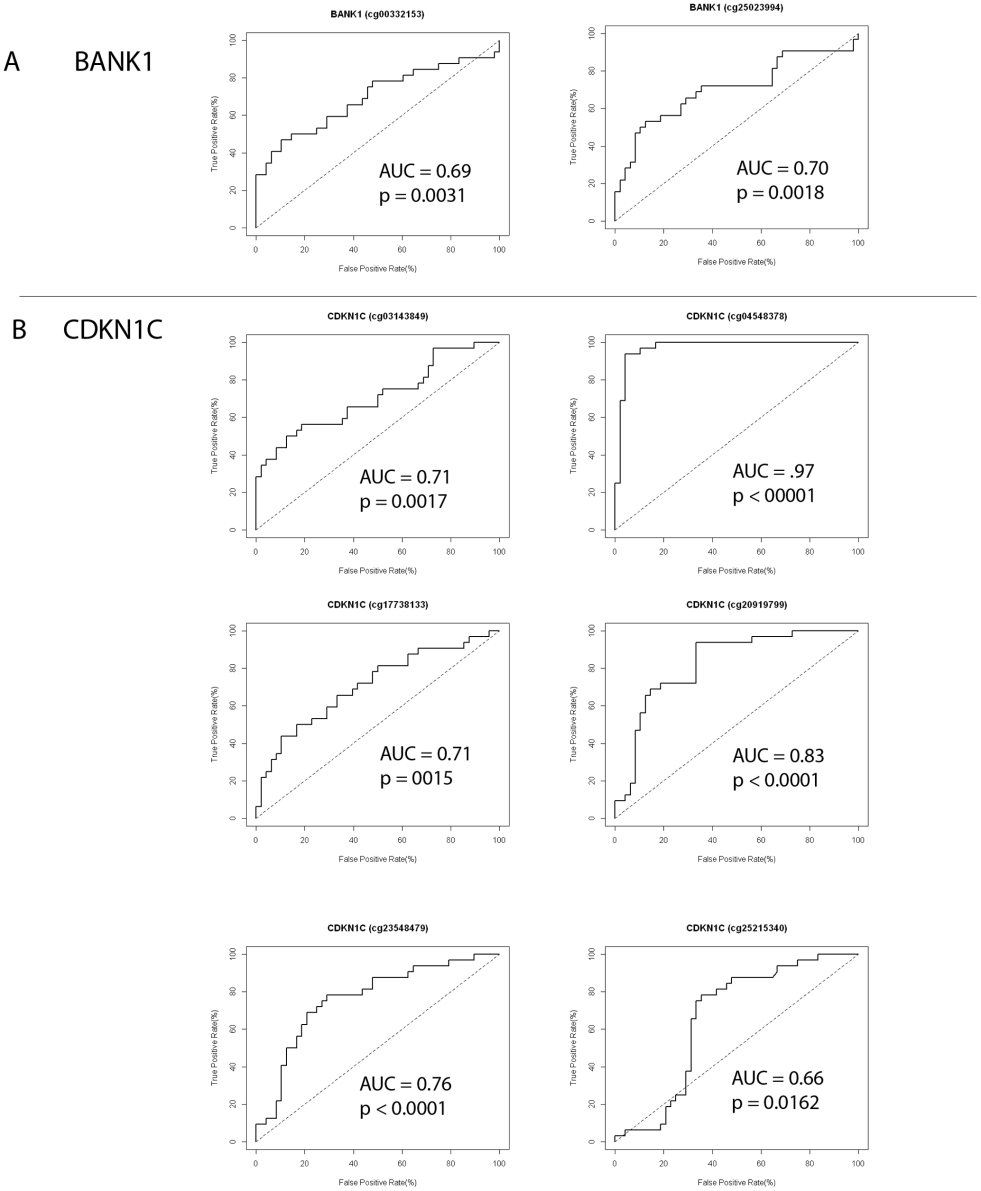
Receiver Operator Characteristic (ROC) Curve for BANK1 and CDKN1C for Determining Differential DNA Methylation Between BBM (n = 32) and Primary Breast Tumors (n = 48). The ROC curve is a graph of the true-positive rate versus the false-positive rate using different threshold values. Data plotted represent two BANK1 HumanMethylation27 array probes and 6/8 CDKN1C probes that demonstrate statistically significant differential methylation between BBM and primary tumors. The area under the curve (AUC) and *p*values are indicated on the graphs.

Similarly, a combined gene expression and methylation analysis was conducted on a sample-by-sample basis for 11 samples in our cohort. Gene expression and methylation data for each sample were compared against non-neoplastic tissue. Differentially expressed genes with log_2_ fold-changes ≥2 or ≤2 and which had a corresponding methylation change with delta beta values >|0.2| were used for further analysis. The resultant gene lists were uploaded into IPA and the Core Analysis workflow was run with default parameters. Analysis of Molecular Functions demonstrated (sample number in parentheses) defects in ‘Cellular Growth and Proliferation’ (7), ‘Cellular Development’ (7), ‘Cellular Movement’ (7) and ‘Cell-Cell Signaling Interactions’ (8). Three samples had predicted decreased activity of cell movement and invasion of tumor cells. Two samples had predicted decrease in the motility of hematological cells such as leukocytes and granulocytes and one sample had predicted decreased activity in cell chemotaxis. Hyper-methylated and down-regulated genes most frequently contributing to cell motility and adhesion included: VAV1 (2), PENK (6), EDN3 (6), EDNRB (4), RELN (5) and ITGAM (4). Other genes affecting cell growth and proliferation which were frequently hypermethylated and downregulated included: CDKN1C (6), CDKN2B (3), CCND2 (4) and BANK1 (7). Other genes of interest include USP44 (6), and CRYAB (4), HSPB2 (1). In eight samples, KRT8 (affecting adhesion and permeability of tight junctions) was found to be hypomethylated and upregulated. We used receiver operator characteristic (ROC) analysis to compare the methylation status of BANK1 and CDKN1C in breast brain metastasis samples to a series of 48 early-stage primary breast cancer samples. The areas under the ROC curves were statistically significant for BANK1 (2/2 HumanMethylation27 array probes) and 6/8 CDKN1C methylation probes representing different CpG loci (*p*<0.01, Mann-Whitney U test). The data demonstrate differential methylation between primary breast tumors and brain metastases, where the metastatic samples were significantly more hypermethylated than primary tumors. The ROC curves for BANK1 and CDKN1C are shown in **Figure 6**. These data highlight the importance of the epigenetic silencing of BANK1 and CDKN1C in the development of BBM.

There was also hypomethylation and upregulation of six X-linked MAGE genes (1 Basal sample), histone gene cluster (7), DNMT3B (4), and IL20 (5). CHODL was upregulated in basal like tumors and downregulated in Her2 and Luminal B tumors, but we also noted hypermethylation and downregulation of CHODL in four Luminal B tumors. Similarly, TFF3 is highly expressed in Luminal B tumors and TFF1 is hypomethylated and overexpressed in fourLuminal B tumors.

We further examined each sample for enrichment in canonical pathways and identified ‘IL8 signaling’, ‘hepatic fibrosis/hepatic stellate cell activation signaling’ and ‘thyroid hormone metabolism signaling’ to be among the most frequently enriched pathways (**Table S14, File S1**). In particular, enrichment of ‘IL8 signaling’ was due mainly to the hypermethylation and downregulation of several key genes such as ANGPT1, ANGPT2, KDR, ITGAM, ITGB2, PIK3CG, PIK3CD and TEK. BRAF and BCL2 were among the hypomethylated and overexpressed genes in this pathway.

### Combined Copy Number, Gene Expression and DNA Methylation Analysis on Single Tumors for the Identification of Putative Tumor Suppressor Genes and Oncogenes

We next combined all three datasets to identify genes that had a one-copy deletion, downregulation and hypermethylation in 11 individual samples; this was anticipated to be an indication of tumor suppressor function for these genes. We identified the following genes (chromosome and frequency in parenthesis): BNC1 (15q25, 2), CHODL (21q11, 2), CRYAB (11q23,2), EDNRB (13q22, 1), FHL1 (Xq26, 1), HS3ST3A1 (17p12, 2), KL (13q13, 1), ME3 (11q14, 2), PENK (8q12, 1), PIK3CD (2), SCARA3 (8p21, 2), SCN3B (11q24, 2), SMYD4 (17p,12, 2), SOX7 (8p23, 2).

Other candidate tumor suppressor genes included those that demonstrated homozygous deletions and loss of expression. Of 11 samples, only the three basal-like samples had evidence of numerous homozygous deletions, which were as follows: Sample BBM6 - CDKN2A, CDKN2B, DMD, DMRTA1, GTPBP6, PLCXD1, PPP2R3B, SPRY2; Sample BBM9 - DMRT2, DMRT3, DOCK8, KANK1, ODZ1, RB1, SMARCA2, STAG2, VLDLR; Sample BBM15 - ARHGAP24, FBXO11, FOXN2, GABPB1, HABP2, LHCGR, LILRB2, MSH6. One luminal tumor had a homozygous deletion of the QRFPR gene.

Similarly, we identified genes that were amplified, upregulated and hypomethylated. These include: CAPN9 (1q42, 3), CEBPG (19q13, 2), CTSE (1q32, 2), DNMT3B (20q11, 2), HIST1H2BJ (6p22, 2), HMGN1 (21q22, 2), IL20 (1q32, 2), MAT1A (10q32, 2), PSCA (8q24, 2), SRMS (20q13, 2) and TSPYL5 (8q22, 2).

## Discussion

The brain is a common sanctuary site of metastatic disease in patients with breast cancer and brain metastases are becoming increasingly prevalent as greater control over systemic disease is achieved. Given the poor clinical outcomes of patients with breast brain metastases, there is urgency to better understand the mechanisms underlying the pathogenesis of brain metastasis as well as to identify novel targeted therapies. Accordingly, we performed a comprehensive genomics and epigenomics analysis using microarray technology to measure alterations at the level of mRNA expression, DNA copy number and DNA methylation.

Copy number analysis identified a number of focal and broad regions of amplifications and deletions. Among the most notable regions of broad gains in our samples were 1q, 5p, 8q, 11q and 20q. Broad-level deletions were identified in 8p, 17p, 21p and Xq. Previous studies have shown that ductal carcinoma *in situ* (DCIS) were associated with chromosomal gains in 1q, 8q and 17q [10]. Most commonly, deletions in DCIS have been shown to occur in 8p, 11q, 13q, 14q and 16q [11], [12]. In invasive breast cancer, gains of 1q, 6p, 8q, 11q, 16p, 17q and 20q are most common and chromosomal losses have been identified in 1p, 8p, 11q, 16q, 18q and 22 [11], [12]. These data suggest some clear overlap of regions involved in primary breast cancer, but also point towards the emergence of other chromosomal alterations that may be unique to breast cancer brain metastasis. Certainly, concomitant gain of 8q with loss of 8p has been previously described for breast cancer, as well as prostate cancer, and has been associated with disease progression and poor patient prognosis [13]. Known breast cancer oncogenes such as MYC (8q) and ERRB2 (17q), while among the common regions of amplifications, were not among the highly expressed genes in our samples.

ATAD2 (8q24), and DERL1 (8q24) were among the frequently amplified and over-expressed genes suggesting they could play an important role in breast brain metastasis. ATAD2 may be a transcriptional coactivator of ESR1 required to induce the expression of estradiol target genes such as CCND1, MYC and E2F1 and may be required for histone hyperacetylation [14]. It has also been identified as a MYC cofactor and correlates with poor breast cancer outcomes [15]. The protein encoded by this gene contains two AAA domains and a bromodomain. AAA family proteins often perform chaperone-like functions that assist in the assembly, operation or disassembly of protein complexes. DERL1 encodes a member of the derlin family of proteins and is thought to participate in an endoplasmic reticulum (ER)-associated degradation response and retrotranslocate misfolded/unfolded proteins into the cytosol for proteosomal degradation. Data in breast cancer cells show that DERL1 expression is increased by ER stress while DERL1 knockdown resulted in decreased development of cancer cells [16]. The NEK2A gene on 1q32 was another frequently amplified and over-expressed gene in the samples we analyzed. The gene encodes a protein serine/threonine kinase that is involved in mitotic regulation. It has recently been described to contribute to the growth potential of DCIS and IDC and expression correlated to higher histological grade and lymph node metastasis [17].

Several samples had co-deletion and downregulation of CRYAB and HSPB2 (both on 11q23) due to deletion and/or hypermethylation. CRYAB (B crystallin) and HSPB2 are two members of the multi-gene small heat shock proteins (sHSPs) family that are typically coexpressed in the mammalian heart, but the biological roles remain poorly defined [18], [19]. CRYAB has been implicated in stress-inducible translocation, antiapoptosis, remodeling of the cytoskeleton, cardioprotection and inheritable cardiomyopathy in humans [19]. It has been reported that 11q22-23 is a frequent target for deletion during the development of many solid tumor types, including breast, ovarian, cervix, stomach, bladder carcinomas and melanoma [20], suggesting tumor suppressor functions in solid tumors including breast brain metastases. Integrated copy number and gene expression analysis also reveal BRAF, AKT1, and IGF1R amplifications and deletion/downregulation of ATM, all of which belong to pathways that lend themselves to therapeutic targeting.

Differential expression analysis reveals significant ontologic profiles associated with G2–M checkpoint and proliferation. A central player in the G2-M cascade is FOXM1, which was overexpressed in a large percentage of breast brain metastases. FOXM1 is a transcriptional activator involved in proliferation, cell-cycle control, and mitosis, through the regulation of many genes involved in the mitotic checkpoint, such as AURKA, AURKB, PLK1, and CENPF. The FOXM1 gene was also recently highlighted as significantly deregulated in serous ovarian tumors and metastatic triple-negative breast cancer [21]. Our DNA methylation analysis demonstrates an overall increase in methylation compared to non-neoplastic tissue. This is interesting since much more of the cancer genome is generally subject to lower methylation levels rather than higher levels of methylation [22]. Nevertheless, this is consistent with our findings demonstrating upregulation due to amplification and/or hypomethylation of DNMT3B and MAT1A. We did however demonstrate lower overall methylation in basal-like tumors, a finding also consistent with basal-like primary breast cancer (TCGA) [23].

Functional annotation of our epigenetically-regulated genes demonstrates a strong relationship to inflammatory and immunological responses and disorders. We identified a propensity of genes related to both tumor and immune cell migration and adhesion to be epigenetically silenced in a high percentage of samples. This phenomenon could be the result of immune cell infiltration into the brain and/or could be also be explained by the fact that progression of metastatic cells from the blood stream into the perivascular space and then to brain parenchyma share similar mechanisms as those employed by cells of the systemic immune system [24]. The latter scenario can be explained by considering that once cells have colonized the brain, migratory-promoting genes of the tumor cells are repressed, as cells have likely reached their ‘final destination’. This would seem to be accompanied by activation of proliferative genes. However, cancer progression does involve the activation of stromal cells including pericytes, fibroblasts and leukocytes [24]. Extravasation of metastatic cells may cause damage to components of the BBB, which may facilitate entry of systemic immune cells in the perivascular space and are known to have both tumor preventing and promoting roles [24].

Over the last decade, there has been a marked improvement in the understanding of the molecular profile of breast cancer, which has suggested that breast cancer may behave as a multiplicity of diseases [25], [26], [27]. Gene expression studies using DNA microarrays have identified at least four distinct subtypes of breast cancer, including Luminal A, Luminal B, HER2+/ER−, and the basal-like subtype [25], [26], [27]. Recent work has shown that compared to the Luminal subtypes, Her2+/ER− and basal-like subtypes have a greater predilection for seeding the brain, a much shorter latency period for doing so and worst overall survival [5], [28], [29]. Applying the PAM50 classifier to our breast cancer brain metastasis series identified a relatively high number of Luminal B tumors compared to Her2+/ER− and basal-like subtypes. Certainly, the availability of samples at the time of accrual could have impacted the frequency of the subtypes in our series. Additionally, since we were not able to obtain the matched primary breast tumors, we were not able to confirm the subtype of the primary tumor to determine if receptor conversion has occurred. However, previous studies have analyzed gene expression signatures known in primary tumors in metastatic tumors [30], [31].

In summary, the breast brain metastases in our series generally appeared to retain molecular features consistent mainly with breast cancer and with their respective subtypes. Identifying changes unique to metastatic tumors will ultimately require study of primary/metastatic pairs. However, whether intrinsic subtype switching has occurred or not, it is clear from this study that molecular signatures resembling those known for primary breast tumors and its subtypes can be identified. Ensuring optimal success for developing novel therapies for breast cancer brain metastases therefore requires consideration of the tumor intrinsic subtype status. Due to the BBB and the unique environment of the brain, novel therapeutic approaches for brain metastases warrant intensive research efforts. Our study has highlighted a number of fundamental genetic and epigenetic aberrations occurring in brain metastases from primary breast tumor with strong implications for future targeted therapies that are aimed at alleviating the burden of this clinically unmet need.

## Supporting Information

File S1 **Supporting figures and tables.** Figure S1: Combined Network for Upstream Analysis of FOXM1 and TBX2. The downstream genes connected to FOXM1 and TBX2 were illustrated as a network in IPA. The mRNA expression ratios are listed below the gene nodes. The legend within figure describes the node and edge color keys. Figure S2: Word Cloud Analysis of Cluster Enrichments. We have used word clouds to visually summarize the textual results from the enrichment analysis of each gene cluster as observed in Figure 3. The results were generated using www.wordle.net web resource. The larger the word, the more times it is mentioned in the enrichment categories. Supplementary Tables in File S1. Table S1a. Table S1b. Table S2. Table S3a. Table S3b. Table S4a. Figure S1. Table S4b. Table S5a–b. Table S6a–b. Table S7. Table S8a–f. Table S9a–f. Figure S2. Table S10. Table S11a–c. Table S11d. Table S12. Table S13. Table S14.
(ZIP)
