# Supplementary material for: Integrated Genomic and Epigenomic Analysis of Breast Cancer Brain Metastasis
Source: PLoS One. 2014 Jan 29;9(1):e85448. doi: 10.1371/journal.pone.0085448 (PMC3906004; doi:10.1371/journal.pone.0085448)
Supplement: File S1 — Supporting figures and tables. Figure S1: Combined Network for Upstream Analysis of FOXM1 and TBX2. The downstream genes connected to FOXM1 and TBX2 were illustrated as a network in IPA. The mRNA expression ratios are listed below the gene nodes. The legend within figure describes the node and edge color keys. Figure S2: Word Cloud Analysis of Cluster Enrichments. We have used word clouds to visually summarize the textual results from the enrichment analysis of each gene cluster as observed in Figure 3. The results were generated using www.wordle.net web resource. The larger the word, the more times it is mentioned in the enrichment categories. Supplementary Tables in File S1. Table S1a. Table S1b. Table S2. Table S3a. Table S3b. Table S4a. Figure S1. Table S4b. Table S5a–b. Table S6a–b. Table S7. Table S8a–f. Table S9a–f. Figure S2. Table S10. Table S11a–c. Table S11d. Table S12. Table S13. Table S14. (ZIP) [file pone.0085448.s001.zip › Supplementary Table S10.pdf]

Supplementary Table 10. List of Differentially Methylated Loci (DML) between BBM and NBn and NBr.

* DMV = differentially methylated value, CHR = chromosome, TSS = transcription start site

Gene Symbol	Illumina TargetID	DMV*	GenBank Accession	CHR*	CpG Island	Distance to TSS*
**Hypermethylated DML**						
**Non-CpG Island**						
ANGPTL2	cg11213150	0.22	NM_012098.2	9	No	587
ANGPTL2	cg09427311	0.21	NM_012098.2	9	No	686
C12orf34	cg02351381	0.24	NM_032829.1	12	No	226
C12orf34	cg01335367	0.22	NM_032829.1	12	No	364
CLIPR-59	cg06432655	0.27	NM_015526.1	19	No	370
COL5A2	cg22774472	0.24	NM_000393.2	2	No	148
FSD1	cg25902889	0.30	NM_024333.1	19	No	399
GSTM2	cg03070194	0.22	NM_000848.2	1	No	7
KCNN3	cg11902458	0.23	NM_002249.3	1	No	8
PRKCG	cg26626089	0.26	NM_002739.3	19	No	398
RAB22A	cg26029902	0.28	NM_020673.2	20	No	346
SLC8A2	cg22123464	0.29	NM_015063.1	19	No	221
SPARCL1	cg19466563	0.24	NM_004684.2	4	No	0
SULT1C2	cg10236239	0.24	NM_006588.2	2	No	93
TRPM1	cg18085517	0.22	NM_002420.3	15	No	361
UGT3A1	cg23317501	0.27	NM_152404.2	5	No	284
**CpG Island**						

ABCA3	cg00949442	0.32	NM_001089.1	16	Yes	1234
ABHD9	cg05488632	0.24	NM_024794.1	19	Yes	57
ACE	cg02131967	0.21	NM_152830.1	17	Yes	328
ACTL6B	cg08572611	0.35	NM_016188.3	7	Yes	136
ACTN2	cg21376883	0.27	NM_001103.1	1	Yes	433
ADCY4	cg12265829	0.26	NM_139247.2	14	Yes	158
ADHFE1	cg01988129	0.24	NM_144650.1	8	Yes	202
ADRA2B	cg21542793	0.27	NM_000682.4	2	Yes	3
ADRB1	cg14826456	0.27	NM_000684.1	10	Yes	289
ADRB3	cg17619823	0.23	NM_000025.1	8	Yes	709
AEBP1	cg02126753	0.26	NM_001129.3	7	Yes	38
AKR1B1	cg13801416	0.31	NM_001628.2	7	Yes	31
AKR1B1	cg18416881	0.31	NM_001628.2	7	Yes	65
ALOX15	cg15843823	0.24	NM_001140.3	17	Yes	4
ALOX15	cg09872233	0.23	NM_001140.3	17	Yes	35
ALPL	cg20645065	0.31	NM_000478.2	1	Yes	269
ALS2CR11	cg11052143	0.21	NM_152525.3	2	Yes	122
ALX4	cg04970352	0.24	NT_009237.17	11	Yes	
ATP5G2	cg08995424	0.22	NM_005176.4	12	Yes	462
ATP8A2	cg12111714	0.30	NM_016529.3	13	Yes	357
ATP8A2	cg18236477	0.28	NM_016529.3	13	Yes	49
BANK1	cg00332153	0.26	NM_017935.2	4	Yes	114
BHMT	cg10660256	0.29	NM_001713.1	5	Yes	0
BOLL	cg17560332	0.23	NM_033030.3	2	Yes	365
BTG4	cg23211240	0.28	NM_017589.2	11	Yes	560
BTG4	cg22879515	0.21	NM_017589.2	11	Yes	451
C19orf35	cg20973210	0.25	NM_198532.1	19	Yes	554
C1orf114	cg08047907	0.37	NM_021179.1	1	Yes	188
C1orf114	cg13958426	0.36	NM_021179.1	1	Yes	33
C2orf10	cg13121699	0.23	NM_194250.1	2	Yes	696
CA3	cg18674980	0.23	NM_005181.2	8	Yes	123

CASP2	cg09243021	0.23	NM_001224.3	7	Yes	383
CD38	cg26043257	0.26	NM_001775.2	4	Yes	307
CD8A	cg17108819	0.30	NM_001768.4	2	Yes	114
CD8B1	cg26850754	0.26	NM_004931.2	2	Yes	242
CDKN2A	cg09099744	0.30	NT_008413.17	9	Yes	
CDKN2A	cg07752420	0.23	NT_008413.17	9	Yes	
CDO1	cg12880658	0.24	NM_001801.2	5	Yes	19
CDO1	cg07644368	0.22	NM_001801.2	5	Yes	380
CENTA2	cg05052633	0.21	NM_018404.1	17	Yes	552
CHAD	cg06818777	0.23	NM_001267.1	17	Yes	297
CHODL	cg24130010	0.27	NM_024944.2	21	Yes	26
CIDEA	cg20950011	0.27	NM_001279.2	18	Yes	7
CLDN11	cg20449692	0.31	NM_005602.4	3	Yes	255
CLDN6	cg07384961	0.28	NM_021195.3	16	Yes	34
CLIC6	cg10722799	0.28	NM_053277.1	21	Yes	11
CNTN2	cg17860158	0.23	NM_005076.2	1	Yes	229
CNTN2	cg03732545	0.23	NM_005076.2	1	Yes	210
COL11A2	cg10143146	0.29	NM_080679.1	6	Yes	1132
COL14A1	cg23196831	0.21	NM_021110.1	8	Yes	185
COL21A1	cg13830624	0.24	NM_030820.2	6	Yes	51
COLEC12	cg19461621	0.21	NM_030781.2	18	Yes	294
CPNE5	cg21716693	0.24	NM_020939.1	6	Yes	338
CR1	cg14726637	0.27	NM_000573.2	1	Yes	194
CRMP1	cg03544320	0.32	NM_001313.3	4	Yes	94
CXCL5	cg10088985	0.23	NM_002994.3	4	Yes	103
CYB5R1	cg18275051	0.23	NM_016243.2	1	Yes	710
CYGB	cg21301440	0.24	NM_134268.3	17	Yes	768
CYYR1	cg10238818	0.31	NM_052954.2	21	Yes	27
DCC	cg02624705	0.24	NT_010966.13	18	Yes	
DES	cg18182399	0.25	NM_001927.3	2	Yes	76
DGKI	cg06277657	0.32	NM_004717.2	7	Yes	765

DKFZp434I1020	cg17886204	0.23	NM_194295.1	15	Yes	138
DLX5	cg11500797	0.29	NT_007933.14	7	Yes	
DLX5	cg16924616	0.26	NM_005221.4	7	Yes	526
DMN	cg13191049	0.20	NM_145728.1	15	Yes	377
DNM3	cg23391785	0.29	NM_015569.2	1	Yes	334
DPYS	cg10303487	0.32	NM_001385.1	8	Yes	219
DPYSL4	cg12109455	0.24	NM_006426.1	10	Yes	333
DRD4	cg06825142	0.29	NM_000797.2	11	Yes	135
DRD5	cg09936561	0.26	NM_000798.3	4	Yes	20
DSC3	cg15439862	0.22	NM_001941.2	18	Yes	96
DYDC1	cg17703212	0.24	NM_138812.1	10	Yes	757
EBF	cg05056120	0.23	NM_024007.2	5	Yes	933
EDG2	cg14563260	0.22	NM_001401.3	9	Yes	955
EDN3	cg04048259	0.27	NM_000114.2	20	Yes	153
EFCAB1	cg22836229	0.33	NM_024593.2	8	Yes	74
EFCBP2	cg02899772	0.25	NM_019065.2	16	Yes	362
EIF5A2	cg10541755	0.23	NM_020390.5	3	Yes	554
EOMES	cg15540820	0.33	NM_005442.2	3	Yes	1498
EPHA10	cg07447922	0.29	NM_173641.1	1	Yes	38
EPO	cg08575537	0.41	NM_000799.2	7	Yes	203
ESX1	cg04721883	0.23	NM_153448.2	X	Yes	11
ESX1	cg09261015	0.23	NM_153448.2	X	Yes	59
EVX1	cg27626299	0.29	NM_001989.2	7	Yes	10
EYA4	cg01805282	0.26	NT_025741.14	6	Yes	
FAM78A	cg12998491	0.22	NM_033387.2	9	Yes	625
FBN2	cg27223047	0.31	NM_001999.3	5	Yes	1090
FBXO39	cg20723355	0.29	NM_153230.1	17	Yes	25
FBXO39	cg02613386	0.29	NM_153230.1	17	Yes	39
FLJ21511	cg25484904	0.24	NM_025087.1	4	Yes	250
FLJ30934	cg19018097	0.28	NM_152760.2	11	Yes	82
FLJ46156	cg18871276	0.24	NM_198499.1	14	Yes	401

FLJ90650	cg25044651	0.31	NM_173800.3	5	Yes	449
FLJ90650	cg15489294	0.25	NM_173800.3	5	Yes	418
FLRT2	cg17410236	0.23	NM_013231.4	14	Yes	7
FLT4	cg00489401	0.34	NM_182925.1	5	Yes	691
FOXE1	cg15802898	0.25	NM_004473.3	9	Yes	336
FOXE3	cg18815943	0.26	NM_012186.1	1	Yes	570
FOXL1	cg06995715	0.24	NM_005250.1	16	Yes	260
FOXL2	cg17503456	0.25	NM_023067.2	3	Yes	253
FZD2	cg25228126	0.32	NM_001466.2	17	Yes	385
G0S2	cg08185241	0.24	NM_015714.2	1	Yes	99
GAL3ST3	cg21238818	0.31	NM_033036.1	11	Yes	9
GALR1	cg04534765	0.34	NM_001480.2	18	Yes	636
GALR2	cg14165663	0.28	NM_003857.2	17	Yes	623
GATA4	cg13434842	0.32	NT_077531.3	8	Yes	
GATA4	cg09626984	0.31	NT_077531.3	8	Yes	
GATA4	cg20279283	0.29	NT_077531.3	8	Yes	
GCM2	cg09829319	0.26	NM_004752.1	6	Yes	140
GDA	cg08768421	0.23	NM_004293.2	9	Yes	211
GDF10	cg07773116	0.25	NM_004962.2	10	Yes	666
GDF7	cg05899618	0.25	NM_182828.2	2	Yes	577
GEFT	cg11452221	0.25	NM_133483.1	12	Yes	388
GIPC2	cg24496666	0.26	NM_017655.4	1	Yes	390
GLP1R	cg25014318	0.29	NM_002062.2	6	Yes	159
GNG4	cg09649610	0.30	NM_004485.2	1	Yes	15
GPC2	cg18691434	0.27	NM_152742.1	7	Yes	435
GPR124	cg20011352	0.26	NM_032777.6	8	Yes	495
GPR25	cg21870884	0.34	NM_005298.2	1	Yes	263
GRASP	cg04034767	0.30	NM_181711.1	12	Yes	159
GREM1	cg21296230	0.24	NM_013372.5	15	Yes	331
GRIN3A	cg18794577	0.32	NM_133445.1	9	Yes	168
GRM6	cg14859460	0.30	NM_000843.2	5	Yes	120

GRM7	cg02332525	0.28	NM_181874.1	3	Yes	226
GSH1	cg26609631	0.26	NM_145657.1	13	Yes	34
GUCY2D	cg25465406	0.28	NM_000180.1	17	Yes	110
H2AFY	cg01550148	0.27	NM_004893.2	5	Yes	33
HIST1H1A	cg14652095	0.26	NM_005325.2	6	Yes	169
HIST1H2BB	cg21250296	0.22	NM_021062.2	6	Yes	520
HIST1H3C	cg25438963	0.22	NM_003531.2	6	Yes	24
HLA-G	cg21529533	0.24	NM_002127.3	6	Yes	497
HOM-TES-103	cg00363813	0.30	NM_015438.1	12	Yes	357
HOM-TES-103	cg01493517	0.29	NM_015438.1	12	Yes	218
HOXA2	cg09871315	0.27	NM_006735.3	7	Yes	288
HOXA4	cg24169822	0.22	NM_002141.2	7	Yes	595
HOXA9	cg27009703	0.29	NM_152739.2	7	Yes	255
HOXB13	cg15786837	0.24	NM_006361.4	17	Yes	847
HOXD10	cg21591742	0.30	NM_002148.2	2	Yes	502
HOXD12	cg23130254	0.27	NM_021193.2	2	Yes	58
HOXD9	cg14991487	0.24	NM_014213.2	2	Yes	316
HRH3	cg10605520	0.24	NM_007232.1	20	Yes	818
HS3ST2	cg19064258	0.27	NM_006043.1	16	Yes	257
HSPA2	cg27120999	0.27	NT_026437.11	14	Yes	
HSPB6	cg24673765	0.26	NM_144617.1	19	Yes	61
HTR7	cg06291867	0.27	NM_019859.2	10	Yes	509
INA	cg25764191	0.26	NM_032727.2	10	Yes	295
ITGA4	cg20415809	0.21	NM_000885.4	2	Yes	236
K6IRS2	cg20050826	0.21	NM_080747.1	12	Yes	3
KCNA2	cg22165175	0.24	NM_004974.2	1	Yes	570
KCNC1	cg27409364	0.29	NM_004976.2	11	Yes	203
KCNC3	cg06572160	0.22	NM_004977.2	19	Yes	733
KCNG3	cg27553955	0.23	NM_133329.4	2	Yes	911
KCNJ10	cg05768141	0.22	NM_002241.2	1	Yes	191
KCNK17	cg08315770	0.30	NM_031460.2	6	Yes	313

KCNQ1DN	cg01530101	0.25	NT_009237.17	11	Yes	
KIF5A	cg04270799	0.23	NM_004984.2	12	Yes	212
KL	cg23282559	0.25	NM_004795.2	13	Yes	559
LAMA1	cg07846220	0.27	NM_005559.2	18	Yes	133
LEP	cg19594666	0.31	NM_000230.1	7	Yes	51
LEP	cg12782180	0.25	NM_000230.1	7	Yes	399
LOC112937	cg19674669	0.25	NM_138416.1	11	Yes	635
LOC387758	cg04622802	0.20	NM_203371.1	11	Yes	244
MLNR	cg02620013	0.23	NM_001507.1	13	Yes	207
MSC	cg23710218	0.27	NM_005098.2	8	Yes	33
MT3	cg16158681	0.26	NM_005954.2	16	Yes	158
NAALAD2	cg05500015	0.23	NM_005467.2	11	Yes	63
NEFH	cg02994956	0.27	NM_021076.2	22	Yes	315
NELL1	cg17371081	0.24	NM_006157.2	11	Yes	179
NEUROD1	cg22359606	0.28	NM_002500.1	2	Yes	238
NEUROG1	cg04330449	0.31	NT_034772.5	5	Yes	
NID2	cg22881914	0.37	NM_007361.2	14	Yes	201
NKX6-2	cg08441806	0.35	NM_177400.1	10	Yes	303
NOS1	cg03538436	0.25	NM_000620.1	12	Yes	212
NPTX2	cg12799895	0.32	NM_002523.1	7	Yes	43
NPY	cg05158615	0.24	NM_000905.2	7	Yes	250
NR2E1	cg03958979	0.31	NM_003269.2	6	Yes	875
NT5E	cg17966619	0.22	NM_002526.1	6	Yes	353
OSR1	cg06509239	0.22	NM_145260.2	2	Yes	197
PABPC5	cg04875162	0.25	NM_080832.1	X	Yes	229
PAQR9	cg00970325	0.23	NM_198504.1	3	Yes	998
PAX7	cg07536847	0.30	NM_002584.1	1	Yes	268
PAX7	cg11428724	0.23	NM_002584.1	1	Yes	132
PDE4DIP	cg11935147	0.27	NM_001002812.1	1	Yes	192
PDE9A	cg00516481	0.22	NM_002606.2	21	Yes	660
PDGFRB	cg12727795	0.24	NM_002609.3	5	Yes	273

PHOX2A	cg08876932	0.30	NM_005169.2	11	Yes	343
PHOX2A	cg18722841	0.30	NM_005169.2	11	Yes	222
PKDREJ	cg11377136	0.29	NM_006071.1	22	Yes	259
PLA2G7	cg18630040	0.25	NM_005084.2	6	Yes	119
POU4F1	cg08097882	0.21	NM_006237.2	13	Yes	179
POU4F1	cg15604467	0.20	NM_006237.2	13	Yes	230
POU4F2	cg24199834	0.28	NM_004575.1	4	Yes	38
POU4F2	cg13262687	0.22	NM_004575.1	4	Yes	509
PPFIA2	cg24719984	0.23	NM_003625.2	12	Yes	355
PPGB	cg08260891	0.21	NM_000308.1	20	Yes	1402
PRAC	cg12374721	0.29	NM_032391.2	17	Yes	242
PRAC	cg01543654	0.22	NM_032391.2	17	Yes	757
PRDM14	cg01295203	0.28	NM_024504.2	8	Yes	637
PRKAR1B	cg13577076	0.25	NM_002735.1	7	Yes	323
PRKCB1	cg05436658	0.26	NM_002738.5	16	Yes	246
PRPH	cg09595479	0.26	NM_006262.3	12	Yes	303
PRR3	cg04273431	0.25	NM_025263.1	6	Yes	1448
PRRT1	cg13035743	0.30	NM_030651.2	6	Yes	35
PTF1A	cg11438428	0.26	NM_178161.1	10	Yes	134
PTGDR	cg09516965	0.36	NM_000953.2	14	Yes	98
PTGER2	cg06738602	0.24	NM_000956.2	14	Yes	479
PTGER2	cg25823578	0.21	NM_000956.2	14	Yes	199
PTPRO	cg10646402	0.26	NM_002848.2	12	Yes	3
RAB11FIP4	cg10149836	0.26	NM_032932.3	17	Yes	670
RAB31	cg17982102	0.25	NM_006868.2	18	Yes	369
RBPSUHL	cg21835643	0.29	NM_014276.2	20	Yes	130
RCN3	cg04378886	0.27	NM_020650.2	19	Yes	520
RELN	cg17923358	0.22	NM_005045.2	7	Yes	344
RHOJ	cg18771300	0.24	NM_020663.2	14	Yes	592
RND2	cg05270634	0.21	NM_005440.3	17	Yes	180
RUSC2	cg15271616	0.22	NM_014806.1	9	Yes	391

SALL3	cg15191648	0.29	NM_171999.1	18	Yes	13
SCARF2	cg14785479	0.27	NM_182895.1	22	Yes	389
SCNN1B	cg23113963	0.27	NM_000336.1	16	Yes	347
SCRL	cg09697795	0.23	NM_152358.2	19	Yes	159
SCRL	cg01857260	0.23	NM_152358.2	19	Yes	14
SCTR	cg15250797	0.22	NM_002980.1	2	Yes	382
SCUBE3	cg00347904	0.25	NM_152753.2	6	Yes	318
SCUBE3	cg21604042	0.25	NM_152753.2	6	Yes	789
SEC31L2	cg20831708	0.36	NM_015490.3	10	Yes	222
SFRP1	cg22418909	0.30	NM_003012.3	8	Yes	242
SFRP1	cg13398291	0.25	NT_007995.14	8	Yes	
SFRP1	cg15839448	0.25	NM_003012.3	8	Yes	450
SFRP2	cg23207990	0.28	NT_016354.18	4	Yes	
SIX6	cg19456540	0.25	NM_007374.1	14	Yes	263
SIX6	cg14611174	0.21	NM_007374.1	14	Yes	185
SLC16A12	cg12005098	0.24	NM_213606.1	10	Yes	25
SLC16A12	cg09186006	0.24	NM_213606.1	10	Yes	14
SLC18A3	cg14008883	0.29	NM_003055.1	10	Yes	564
SLC18A3	cg11389172	0.27	NM_003055.1	10	Yes	48
SLC22A3	cg07237939	0.25	NM_021977.2	6	Yes	329
SLC2A14	cg05521696	0.25	NM_153449.2	12	Yes	0
SLC38A4	cg07601320	0.28	NM_018018.2	12	Yes	102
SLC5A7	cg16232126	0.28	NM_021815.2	2	Yes	10
SLC6A2	cg04490714	0.29	NM_001043.2	16	Yes	8
SLC9A3	cg02748539	0.24	NM_004174.1	5	Yes	178
SMOC2	cg11612345	0.23	NM_022138.1	6	Yes	460
SNCAIP	cg04747322	0.26	NM_005460.2	5	Yes	512
SNN	cg09816471	0.23	NM_003498.3	16	Yes	457
SOX1	cg06675478	0.28	NM_005986.2	13	Yes	127
SOX17	cg02919422	0.28	NM_022454.2	8	Yes	49
SOX7	cg08056146	0.22	NM_031439.2	8	Yes	9

SPAG6	cg06908778	0.30	NM_012443.2	10	Yes	203
SPATS1	cg22970435	0.25	NM_145026.2	6	Yes	258
SST	cg02164046	0.34	NM_001048.3	3	Yes	53
SSTR4	cg17586860	0.29	NM_001052.1	20	Yes	215
ST8SIA2	cg20339230	0.25	NM_006011.3	15	Yes	220
STAC2	cg17471928	0.25	NM_198993.2	17	Yes	144
STK33	cg08788717	0.27	NM_030906.2	11	Yes	3
SV2A	cg01705587	0.23	NM_014849.2	1	Yes	66
SYCP1	cg18087477	0.24	NM_003176.2	1	Yes	142
SYDE1	cg04981492	0.25	NM_033025.4	19	Yes	499
SYN2	cg15873301	0.29	NM_133625.2	3	Yes	406
SYT2	cg22594309	0.23	NM_177402.3	1	Yes	41
TAL1	cg19797376	0.26	NM_003189.1	1	Yes	32
TCTEX1D1	cg24110050	0.26	NM_152665.1	1	Yes	289
TLX3	cg25720804	0.28	NM_021025.2	5	Yes	101
TMEM130	cg15279364	0.24	NM_152913.1	7	Yes	680
TNFRSF10C	cg14015044	0.22	NM_003841.2	8	Yes	185
TNFRSF10D	cg10964421	0.25	NT_023666.17	8	Yes	
TNFRSF10D	cg23051664	0.22	NT_023666.17	8	Yes	
TNFRSF8	cg17897879	0.23	NM_001243.3	1	Yes	3
TP73	cg04391111	0.30	NT_004321.17	1	Yes	
TP73	cg00565688	0.27	NM_005427.1	1	Yes	917
TRH	cg01009664	0.35	NM_007117.1	3	Yes	50
TUBB6	cg07307078	0.29	NM_032525.1	18	Yes	627
UCP1	cg14223995	0.26	NM_021833.3	4	Yes	208
UGT3A2	cg07084163	0.21	NM_174914.2	5	Yes	201
UNQ9433	cg17162024	0.32	NM_207413.1	8	Yes	433
VSX1	cg06151165	0.34	NM_014588.4	20	Yes	513
VSX1	cg23097006	0.31	NM_014588.4	20	Yes	1050
WDR8	cg26128092	0.28	NM_017818.2	1	Yes	913
WIT-1	cg19718882	0.22	NM_015855.2	11	Yes	761

WNT2	cg01830294	0.30	NM_003391.1	7	Yes	149
WRN	cg09945801	0.24	NM_000553.2	8	Yes	639
WT1	cg15107670	0.26	NM_000378.3	11	Yes	328
ZFP41	cg12680609	0.30	NM_173832.3	8	Yes	609
ZFP42	cg06274159	0.33	NM_174900.2	4	Yes	58
ZNF132	cg13877915	0.30	NM_003433.2	19	Yes	83
ZNF154	cg08668790	0.29	NM_003444.1	19	Yes	100
ZNF454	cg03355526	0.29	NM_182594.1	5	Yes	191
ZNF454	cg23037403	0.24	NM_182594.1	5	Yes	41
ZNF540	cg03975694	0.28	NM_152606.2	19	Yes	164
ZNF660	cg22598028	0.32	NM_173658.1	3	Yes	36
ZNF677	cg16708981	0.27	NM_182609.1	19	Yes	498
ZNF96	cg02622316	0.30	NM_014724.2	6	Yes	98
**Hypomethylated DML**						
**Non-CpG Island**						
AIM2	cg10636246	-0.23	NM_004833.1	1	No	326
AIM2	cg11003133	-0.29	NM_004833.1	1	No	256
ANXA9	cg07337598	-0.25	NM_003568.1	1	No	611
APCS	cg26353877	-0.24	NM_001639.2	1	No	270
C11orf38	cg23743472	-0.22	NM_212555.1	11	No	520
C17orf73	cg18490846	-0.28	NM_017928.1	17	No	843
C1orf116	cg01119135	-0.33	NM_023938.4	1	No	567
C1orf158	cg24338843	-0.22	NM_152290.1	1	No	504
C6	cg11976616	-0.22	NM_000065.1	5	No	440
C8ORFK32	cg06276653	-0.24	NM_015912.2	8	No	12
CD1B	cg04574507	-0.28	NM_001764.1	1	No	220
CLDN17	cg13792279	-0.22	NM_012131.1	21	No	386
CNKSR1	cg13553204	-0.30	NM_006314.1	1	No	545

CNTN4	cg10503138	-0.24	NM_175607.1	3	No	144
CNTNAP4	cg06793062	-0.27	NM_033401.2	16	No	119
CPA3	cg13424229	-0.21	NM_001870.1	3	No	1247
CRP	cg08474603	-0.27	NM_000567.2	1	No	52
CYP11B1	cg09120035	-0.24	NM_000497.3	8	No	91
CYP3A43	cg13364756	-0.27	NM_022820.3	7	No	443
DARC	cg18552413	-0.27	NM_002036.2	1	No	100
DDR1	cg03270204	-0.25	NM_001954.3	6	No	223
DEFB103A	cg25214366	-0.24	NM_018661.2	8	No	197
DEFB119	cg18462653	-0.25	NM_153289.2	20	No	230
DENND2D	cg00619207	-0.23	NM_024901.3	1	No	87
EFNA3	cg17582777	-0.26	NM_004952.3	1	No	1248
ERAF	cg02989940	-0.26	NM_016633.2	16	No	31
FCER1A	cg14696870	-0.23	NM_002001.2	1	No	627
FCRL5	cg03329572	-0.22	NM_031281.1	1	No	943
FLJ20184	cg27126442	-0.28	NM_017700.1	4	No	29
FLJ25410	cg05215575	-0.28	NM_144605.1	16	No	375
FLJ44674	cg13897627	-0.28	NM_207449.1	16	No	32
FSHB	cg27420123	-0.30	NM_000510.2	11	No	1090
GPLD1	cg14023451	-0.25	NM_001503.2	6	No	37
GRB7	cg03684977	-0.34	NM_005310.2	17	No	384
HBG1	cg01598642	-0.21	NM_000559.2	11	No	952
IL20	cg01103730	-0.30	NM_018724.3	1	No	450
IVL	cg05440289	-0.23	NM_005547.2	1	No	80
KRT4	cg17667972	-0.27	NM_002272.1	12	No	42
KRT8	cg01835489	-0.31	NM_002273.2	12	No	450
KRTAP11-1	cg07014174	-0.27	NM_175858.2	21	No	114
KRTAP13-1	cg02764897	-0.23	NM_181599.1	21	No	31
KRTAP13-3	cg16431978	-0.23	NM_181622.1	21	No	298
KRTAP13-4	cg14062083	-0.27	NM_181600.1	21	No	235
KRTAP15-1	cg16812893	-0.25	NM_181623.1	21	No	429

KRTAP8-1	cg24423088	-0.31	NM_175857.3	21	No	205
LOC116123	cg24272559	-0.31	NM_138784.1	1	No	437
LOC122258	cg21624282	-0.22	NM_145248.2	13	No	114
MAGEA1	cg23776892	-0.22	NM_004988.3	X	No	145
MAGEA12	cg02504280	-0.21	NM_005367.4	X	No	1335
MAGEB6	cg16122592	-0.23	NM_173523.2	X	No	155
MAGEB6	cg10127415	-0.23	NM_173523.2	X	No	34
MB	cg24820809	-0.29	NM_005368.2	22	No	149
MGC9712	cg06194808	-0.41	NM_152689.2	7	No	470
MUC15	cg03087937	-0.25	NM_145650.2	11	No	224
MYH1	cg00134787	-0.24	NM_005963.2	17	No	460
NALP10	cg20311730	-0.22	NM_176821.2	11	No	135
NALP9	cg08475088	-0.25	NM_176820.2	19	No	477
NIPSNAP1	cg13797031	-0.26	NM_003634.1	22	No	414
OR10A5	cg22951794	-0.22	NM_178168.1	11	No	366
OR12D3	cg20856834	-0.26	NM_030959.2	6	No	546
OR1G1	cg27622610	-0.27	NM_003555.1	17	No	660
OR5I1	cg25890048	-0.25	NM_006637.1	11	No	433
OR5V1	cg24076830	-0.22	NM_030876.4	6	No	1079
OR8B8	cg14620221	-0.23	NM_012378.1	11	No	42
OR8B8	cg16612699	-0.24	NM_012378.1	11	No	237
PAEP	cg01055695	-0.23	NM_001018049.1	9	No	654
PROM2	cg20775254	-0.30	NM_144707.1	2	No	504
PRSS2	cg13944141	-0.25	NM_002770.2	7	No	46
REG1A	cg05828624	-0.23	NM_002909.3	2	No	115
REG1B	cg07841014	-0.25	NM_006507.2	2	No	374
REG3A	cg24240626	-0.23	NM_138937.1	2	No	159
REG3A	cg27342801	-0.30	NM_138937.1	2	No	394
REG3G	cg00627233	-0.21	NM_001008387.1	2	No	915
REG3G	cg00918005	-0.27	NM_001008387.1	2	No	384
RGPD5	cg02148642	-0.28	NM_032260.2	2	No	749

RIMBP2	cg09847584	-0.26	NM_015347.3	12	No	24
RORC	cg25112191	-0.26	NM_005060.3	1	No	88
SCGB2A1	cg16986846	-0.26	NM_002407.1	11	No	34
SCGB2A2	cg22862656	-0.26	NM_002411.1	11	No	29
SCN7A	cg25995212	-0.25	NM_002976.1	2	No	494
SIGLEC12	cg18986165	-0.20	NM_033329.1	19	No	947
SLC24A2	cg12348970	-0.25	NM_020344.1	9	No	383
SPDEF	cg07705908	-0.29	NM_012391.1	6	No	166
SULT2A1	cg19139729	-0.24	NM_003167.2	19	No	31
TFF1	cg02643667	-0.32	NM_003225.2	21	No	55
TMPRSS11F	cg20695587	-0.26	NM_207407.1	4	No	172
TNFSF11	cg21094154	-0.21	NM_003701.2	13	No	326
TPO	cg10370591	-0.29	NM_000547.3	2	No	196
TRHR	cg22268164	-0.22	NM_003301.1	8	No	26
UNQ473	cg15937958	-0.26	NM_198477.1	19	No	3
UNQ9391	cg01072821	-0.23	NM_198464.1	8	No	916
VSIG9	cg20832020	-0.21	NM_173799.2	3	No	37
XDH	cg26767897	-0.31	NM_000379.2	2	No	265
ZNF80	cg03109316	-0.25	NM_007136.1	3	No	1379
**CpG Island**						
C11orf39	cg25943276	-0.23	NM_207432.1	11	Yes	434
C20orf151	cg02537838	-0.28	NM_080833.1	20	Yes	6
CALN1	cg21003606	-0.26	NM_031468.2	7	Yes	66
DEGS1	cg23213217	-0.31	NM_144780.1	1	Yes	773
ELF5	cg01473816	-0.23	NM_001422.2	11	Yes	49
FFAR2	cg15479752	-0.30	NM_005306.1	19	Yes	245
FGF6	cg21902327	-0.24	NM_020996.1	12	Yes	6
FLJ00060	cg03602500	-0.33	NM_033206.1	19	Yes	449
GPR56	cg04001668	-0.33	NM_201525.1	16	Yes	389
GRM4	cg01962826	-0.32	NM_000841.1	6	Yes	476

H19	cg26808784	-0.25	NT_009237.17	11	Yes	
ILDR1	cg04059863	-0.27	NM_175924.2	3	Yes	600
KDELR2	cg27221338	-0.27	NM_006854.2	7	Yes	1325
KRTAP6-2	cg19306866	-0.21	NM_181604.1	21	Yes	196
MAGEA3	cg07545232	-0.21	NM_005362.3	X	Yes	2
MC3R	cg19226099	-0.24	NM_019888.2	20	Yes	296
MGC10993	cg12108912	-0.33	NM_030577.1	2	Yes	806
MORC1	cg19857541	-0.25	NM_014429.2	3	Yes	168
MST1R	cg08687163	-0.24	NM_002447.1	3	Yes	217
NTF3	cg02554564	-0.26	NM_002527.3	12	Yes	135
PTPN6	cg04956511	-0.23	NM_002831.3	12	Yes	268
SERPINB5	cg20837735	-0.31	NM_002639.2	18	Yes	42
SLC2A10	cg27610561	-0.25	NM_030777.3	20	Yes	495
