# Supplementary material for: Integrated Genomic and Epigenomic Analysis of Breast Cancer Brain Metastasis
Source: PLoS One. 2014 Jan 29;9(1):e85448. doi: 10.1371/journal.pone.0085448 (PMC3906004; doi:10.1371/journal.pone.0085448)
Supplement: File S1 — Supporting figures and tables. Figure S1: Combined Network for Upstream Analysis of FOXM1 and TBX2. The downstream genes connected to FOXM1 and TBX2 were illustrated as a network in IPA. The mRNA expression ratios are listed below the gene nodes. The legend within figure describes the node and edge color keys. Figure S2: Word Cloud Analysis of Cluster Enrichments. We have used word clouds to visually summarize the textual results from the enrichment analysis of each gene cluster as observed in Figure 3. The results were generated using www.wordle.net web resource. The larger the word, the more times it is mentioned in the enrichment categories. Supplementary Tables in File S1. Table S1a. Table S1b. Table S2. Table S3a. Table S3b. Table S4a. Figure S1. Table S4b. Table S5a–b. Table S6a–b. Table S7. Table S8a–f. Table S9a–f. Figure S2. Table S10. Table S11a–c. Table S11d. Table S12. Table S13. Table S14. (ZIP) [file pone.0085448.s001.zip › Supplementary Table S11a.pdf]

Supplementary Table 11a. There are 95 differentially methylated loci between Basal-like andLuminal B BBM tumors.

* DMV = differentially methylated value, CHR = chromosome, TSS = transcription start site

Gene Symbol	Illumina TargetID	DMV	GeneBank Accession	CHR	CpG Island	Distance to TSS
ADAM12	cg13488201	0.46	NM_003474.3	10	Yes	258
ALDH1A3	cg23191950	0.48	NM_000693.1	15	Yes	987
ALDH1A3	cg19224278	0.43	NM_000693.1	15	Yes	1044
ALX4	cg08914623	0.42	NT_009237.17	11	Yes	
AP2A2	cg01321151	0.33	NM_012305.2	11	Yes	318
APC2	cg18133957	0.39	NM_005883.2	19	Yes	345
ARF5	cg12288726	0.43	NM_001662.2	7	Yes	660
ARMC2	cg03874127	0.33	NM_032131.3	6	Yes	490
C19orf19	cg18493147	0.37	NM_182577.1	19	Yes	538
C1orf35	cg14925024	0.41	NM_024319.2	1	Yes	683
C20orf24	cg19562969	0.32	NM_018840.2	20	Yes	585
C21orf124	cg23178308	-0.47	NM_032920.1	21	Yes	4
CDKL1	cg16077929	0.35	NM_004196.3	14	No	108
CEBPG	cg15046693	0.46	NM_001806.2	19	Yes	733
CGI-69	cg03192551	0.44	NM_016016.1	17	Yes	968
CHI3L1	cg03625911	0.36	NM_001276.1	1	No	140
CORIN	cg02553486	0.39	NM_006587.2	4	Yes	36
CPM	cg02266731	0.35	NM_001874.3	12	No	313
CSF1R	cg07260017	0.34	NM_005211.2	5	No	455
CYB5R2	cg03826976	0.39	NM_016229.2	11	Yes	612
DDEFL1	cg11155265	0.25	NM_017707.2	1	Yes	774
DPM1	cg16332224	0.27	NM_003859.1	20	Yes	1346
EPAS1	cg17518825	0.32	NM_001430.3	2	Yes	1102

ESX1	cg04721883	0.48	NM_153448.2	X	Yes	11
ESX1	cg09261015	0.44	NM_153448.2	X	Yes	59
FANCG	cg05293216	0.42	NM_004629.1	9	Yes	799
FBXO17	cg24010336	0.34	NM_024907.5	19	Yes	878
FGF19	cg26096837	0.51	NM_005117.2	11	Yes	868
FLJ20701	cg02799466	0.31	NM_017933.3	2	No	430
FLJ30046	cg08504583	0.37	NM_144595.2	13	No	95
FLJ30046	cg19782598	0.31	NM_144595.2	13	No	204
FLJ46358	cg12682367	0.36	NM_207439.2	13	No	244
FOXH1	cg05113558	0.39	NM_003923.1	8	Yes	625
GFAP	cg21944455	0.36	NM_002055.2	17	No	1401
GFPT1	cg25195673	0.38	NM_002056.1	2	Yes	620
GPM6B	cg21229055	0.43	NM_001001995.1	X	Yes	402
GPR62	cg08072716	0.30	NM_080865.2	3	Yes	295
GPRASP2	cg00862041	0.31	NM_138437.3	X	Yes	323
H-plk	cg00245878	0.35	NM_015852.1	7	No	539
HOXA11	cg17950095	0.40	NM_005523.4	7	Yes	130
HTR3D	cg14483391	0.37	NM_182537.2	3	No	105
KCNAB1	cg23873703	0.37	NM_003471.2	3	Yes	277
KCNQ1	cg27491887	0.39	NT_009237.17	11	Yes	
KCNQ1DN	cg05656180	0.34	NT_009237.17	11	Yes	
KRT17	cg00214794	0.28	NM_000422.1	17	No	186
LAIR1	cg06238491	0.40	NM_002287.2	19	No	22
LASS2	cg18611122	0.37	NM_013384.3	1	Yes	577
LOC348840	cg02141570	0.20	NM_182631.1	3	Yes	1042
LRFN4	cg05111110	0.35	NM_024036.3	11	Yes	305
LRRC17	cg20442697	0.45	NM_005824.1	7	No	20
LRRC17	cg02724472	0.24	NM_005824.1	7	No	159
LY6D	cg07572435	0.38	NM_003695.2	8	No	5
MAPRE1	cg14303674	0.32	NM_012325.1	20	Yes	426
MGC39606	cg17231524	0.28	NM_203306.1	X	Yes	634

MGC40222	cg22572779	0.40	NM_152738.1	6	No	13
MIA	cg25152942	0.37	NM_006533.1	19	No	298
MLZE	cg24243265	0.38	NM_031415.1	8	No	717
MPI	cg13828047	0.37	NM_002435.1	15	Yes	280
NOL10	cg02196655	0.47	NM_024894.1	2	Yes	666
NUP50	cg07986773	0.31	NM_153645.1	22	Yes	1117
PAK2	cg21030598	0.23	NM_002577.3	3	Yes	490
PCYT1B	cg20922422	0.41	NM_004845.3	X	Yes	316
PF4V1	cg20615832	0.38	NM_002620.1	4	Yes	159
PKP1	cg09009380	0.41	NM_001005337.1	1	Yes	394
PPP1R14A	cg12830694	0.34	NM_033256.1	19	No	624
PRTN3	cg09134726	0.34	NM_002777.3	19	No	97
RABGEF1	cg18884741	0.39	NM_014504.1	7	No	923
RB1	cg13221796	0.31	NM_000321.1	13	No	992
SCARA5	cg07634191	0.45	NM_173833.3	8	Yes	20
SCNN1D	cg13587552	0.30	NM_002978.2	1	No	1135
SEMA3B	cg14911395	0.54	NT_022517.17	3	Yes	
SEMA3B	cg26918728	0.42	NT_022517.17	3	Yes	
SETX	cg06353948	0.39	NM_015046.4	9	Yes	644
SGPP2	cg11300809	0.33	NM_152386.2	2	Yes	685
SLC25A10	cg07845392	0.33	NM_012140.3	17	Yes	1213
SLC35C1	cg10516359	0.47	NM_018389.3	11	Yes	427
SMCR7	cg26771272	0.39	NM_139162.2	17	Yes	686
SPAG4L	cg08642068	0.27	NM_080675.3	20	No	463
SPOCK2	cg10983208	0.43	NM_014767.1	10	Yes	447
TINAGL1	cg14869028	0.33	NM_022164.1	1	Yes	1
TLR1	cg03430998	0.31	NM_003263.3	4	No	645
TM4SF18	cg11761535	0.37	NM_138786.1	3	No	388
TNFAIP2	cg20368904	0.42	NM_006291.2	14	Yes	424
TRIM29	cg13625403	0.41	NM_058193.1	11	Yes	353
TRPM6	cg16509045	0.29	NM_017662.3	9	No	47

TSCOT	cg10726357	0.28	NM_033051.2	9	Yes	498
TTC9B	cg00058938	0.32	NM_152479.3	19	No	435
TTLL11	cg27218220	0.40	NM_194252.1	9	Yes	833
TTLL3	cg03375833	0.28	NM_015644.2	3	No	701
ULK1	cg18081313	0.32	NM_003565.1	12	Yes	1087
UNQ467	cg10305797	0.45	NM_207392.1	19	No	321
VTCN1	cg22424746	0.35	NM_024626.1	1	No	234
ZFR	cg13954292	0.51	NM_016107.3	5	Yes	679
ZNF38	cg19370284	0.38	NM_145914.2	7	No	411
ZNF619	cg21942438	0.21	NM_173656.1	3	No	546
