# Supplementary material for: Integrated Genomic and Epigenomic Analysis of Breast Cancer Brain Metastasis
Source: PLoS One. 2014 Jan 29;9(1):e85448. doi: 10.1371/journal.pone.0085448 (PMC3906004; doi:10.1371/journal.pone.0085448)
Supplement: File S1 — Supporting figures and tables. Figure S1: Combined Network for Upstream Analysis of FOXM1 and TBX2. The downstream genes connected to FOXM1 and TBX2 were illustrated as a network in IPA. The mRNA expression ratios are listed below the gene nodes. The legend within figure describes the node and edge color keys. Figure S2: Word Cloud Analysis of Cluster Enrichments. We have used word clouds to visually summarize the textual results from the enrichment analysis of each gene cluster as observed in Figure 3. The results were generated using www.wordle.net web resource. The larger the word, the more times it is mentioned in the enrichment categories. Supplementary Tables in File S1. Table S1a. Table S1b. Table S2. Table S3a. Table S3b. Table S4a. Figure S1. Table S4b. Table S5a–b. Table S6a–b. Table S7. Table S8a–f. Table S9a–f. Figure S2. Table S10. Table S11a–c. Table S11d. Table S12. Table S13. Table S14. (ZIP) [file pone.0085448.s001.zip › Supplementary Table S11c.pdf]

Supplementary Table 11c. There are 71 differentially methylated loci between basal-like and Her2+/ER- BBM tumors.

* DMV = differentially methylated value, CHR = chromosome, TSS = transcription start site

Gene Symbol	Illumina TargetID	DMV	GeneBank Accession	CHR	CpG Island	Distance to TSS
KRT17	cg00214794	0.34	NM_000422.1	17	No	186
H-plk	cg00245878	0.40	NM_015852.1	7	No	539
CPM	cg02266731	0.33	NM_001874.3	12	No	313
CORIN	cg02553486	0.23	NM_006587.2	4	Yes	36
LRRC17	cg02724472	0.22	NM_005824.1	7	No	159
FLJ20701	cg02799466	0.32	NM_017933.3	2	No	430
TTLL3	cg03375833	0.35	NM_015644.2	3	No	701
TLR1	cg03430998	0.22	NM_003263.3	4	No	645
CYB5R2	cg03826976	0.27	NM_016229.2	11	Yes	612
ARMC2	cg03874127	0.22	NM_032131.3	6	Yes	490
LRFN4	cg05111110	0.32	NM_024036.3	11	Yes	305
FOXH1	cg05113558	0.42	NM_003923.1	8	Yes	625
FANCG	cg05293216	0.38	NM_004629.1	9	Yes	799
LAIR1	cg06238491	0.32	NM_002287.2	19	No	22
SETX	cg06353948	0.20	NM_015046.4	9	Yes	644
ICAM3	cg06855803	0.22	NM_002162.2	19	No	57
CSF1R	cg07260017	0.27	NM_005211.2	5	No	455
LY6D	cg07572435	0.45	NM_003695.2	8	No	5
SCARA5	cg07634191	0.30	NM_173833.3	8	Yes	20
SLC25A10	cg07845392	0.32	NM_012140.3	17	Yes	1213
NUP50	cg07986773	0.35	NM_153645.1	22	Yes	1117
FLJ30046	cg08504583	0.32	NM_144595.2	13	No	95
SPAG4L	cg08642068	0.25	NM_080675.3	20	No	463
ALX4	cg08914623	0.28	NT_009237.17	11	Yes	

PKP1	cg09009380	0.26	NM_001005337.1	1	Yes	394
PRTN3	cg09134726	0.31	NM_002777.3	19	No	97
UNQ467	cg10305797	0.27	NM_207392.1	19	No	321
SLC35C1	cg10516359	0.43	NM_018389.3	11	Yes	427
TSCOT	cg10726357	0.26	NM_033051.2	9	Yes	498
TM4SF18	cg11761535	0.34	NM_138786.1	3	No	388
ARF5	cg12288726	0.33	NM_001662.2	7	Yes	660
FLJ46358	cg12682367	0.25	NM_207439.2	13	No	244
PPP1R14A	cg12830694	0.34	NM_033256.1	19	No	624
RB1	cg13221796	0.28	NM_000321.1	13	No	992
SCNN1D	cg13587552	0.27	NM_002978.2	1	No	1135
TRIM29	cg13625403	0.43	NM_058193.1	11	Yes	353
MPI	cg13828047	0.26	NM_002435.1	15	Yes	280
ZFR	cg13954292	0.44	NM_016107.3	5	Yes	679
HTR3D	cg14483391	0.30	NM_182537.2	3	No	105
SEMA3B	cg14911395	0.25	NT_022517.17	3	Yes	
C1orf35	cg14925024	0.36	NM_024319.2	1	Yes	683
CDKL1	cg16077929	0.27	NM_004196.3	14	No	108
DPM1	cg16332224	0.33	NM_003859.1	20	Yes	1346
TRPM6	cg16509045	0.24	NM_017662.3	9	No	47
EPAS1	cg17518825	0.28	NM_001430.3	2	Yes	1102
HOXA11	cg17950095	0.26	NM_005523.4	7	Yes	130
ULK1	cg18081313	0.27	NM_003565.1	12	Yes	1087
APC2	cg18133957	0.23	NM_005883.2	19	Yes	345
C19orf19	cg18493147	0.34	NM_182577.1	19	Yes	538
LASS2	cg18611122	0.24	NM_013384.3	1	Yes	577
RABGEF1	cg18884741	0.40	NM_014504.1	7	No	923
ALDH1A3	cg19224278	0.36	NM_000693.1	15	Yes	1044
ZNF38	cg19370284	0.31	NM_145914.2	7	No	411
C20orf24	cg19562969	0.27	NM_018840.2	20	Yes	585
FLJ30046	cg19782598	0.28	NM_144595.2	13	No	204

TNFAIP2	cg20368904	0.36	NM_006291.2	14	Yes	424
LRRC17	cg20442697	0.34	NM_005824.1	7	No	20
PF4V1	cg20615832	0.39	NM_002620.1	4	Yes	159
GFAP	cg21944455	0.33	NM_002055.2	17	No	1401
VTCN1	cg22424746	0.23	NM_024626.1	1	No	234
MGC40222	cg22572779	0.39	NM_152738.1	6	No	13
ALDH1A3	cg23191950	0.41	NM_000693.1	15	Yes	987
KCNAB1	cg23873703	0.50	NM_003471.2	3	Yes	277
MLZE	cg24243265	0.26	NM_031415.1	8	No	717
MIA	cg25152942	0.33	NM_006533.1	19	No	298
GFPT1	cg25195673	0.36	NM_002056.1	2	Yes	620
FGF19	cg26096837	0.28	NM_005117.2	11	Yes	868
SEMA3B	cg26918728	0.38	NT_022517.17	3	Yes	
TTLL11	cg27218220	0.38	NM_194252.1	9	Yes	833
KCNQ1	cg27491887	0.26	NT_009237.17	11	Yes	
