# Supplementary material for: Integrated Genomic and Epigenomic Analysis of Breast Cancer Brain Metastasis
Source: PLoS One. 2014 Jan 29;9(1):e85448. doi: 10.1371/journal.pone.0085448 (PMC3906004; doi:10.1371/journal.pone.0085448)
Supplement: File S1 — Supporting figures and tables. Figure S1: Combined Network for Upstream Analysis of FOXM1 and TBX2. The downstream genes connected to FOXM1 and TBX2 were illustrated as a network in IPA. The mRNA expression ratios are listed below the gene nodes. The legend within figure describes the node and edge color keys. Figure S2: Word Cloud Analysis of Cluster Enrichments. We have used word clouds to visually summarize the textual results from the enrichment analysis of each gene cluster as observed in Figure 3. The results were generated using www.wordle.net web resource. The larger the word, the more times it is mentioned in the enrichment categories. Supplementary Tables in File S1. Table S1a. Table S1b. Table S2. Table S3a. Table S3b. Table S4a. Figure S1. Table S4b. Table S5a–b. Table S6a–b. Table S7. Table S8a–f. Table S9a–f. Figure S2. Table S10. Table S11a–c. Table S11d. Table S12. Table S13. Table S14. (ZIP) [file pone.0085448.s001.zip › Supplementary Table S11d.pdf]

Supplementary Table 11d. A list of differentially methylated loci that can discriminate between three intrinsic BBM subtypes. See Figure 4c for associated heat map. Values for Non-neoplastic brain (NBn) and non-neoplastic breast (NBr) are also shown in the the table.

Gene Symbol	Illumina TargetID	GeneBank Accession	Basal-like	Her2+/ER-	Luminal B	NBr	NBn	CHR	CpG Island	Distance to TSS
TTC9B	cg00058938	NM_152479.3	0.22	0.29	0.55	0.35	0.32	19	No	435
KRT17	cg00214794	NM_000422.1	0.42	0.73	0.68	0.63	0.68	17	No	186
H-plk	cg00245878	NM_015852.1	0.37	0.76	0.71	0.68	0.74	7	No	539
AP2A2	cg01321151	NM_012305.2	0.26	0.40	0.59	0.34	0.42	11	Yes	318
LOC348840	cg02141570	NM_182631.1	0.36	0.55	0.57	0.42	0.36	3	Yes	1042
NOL10	cg02196655	NM_024894.1	0.15	0.28	0.62	0.25	0.22	2	Yes	666
CPM	cg02266731	NM_001874.3	0.49	0.75	0.78	0.66	0.70	12	No	313
CORIN	cg02553486	NM_006587.2	0.19	0.42	0.58	0.24	0.28	4	Yes	36
LRRC17	cg02724472	NM_005824.1	0.30	0.53	0.56	0.37	0.30	7	No	159
FLJ20701	cg02799466	NM_017933.3	0.39	0.71	0.70	0.53	0.60	2	No	430
CGI-69	cg03192551	NM_016016.1	0.06	0.07	0.52	0.11	0.24	17	Yes	968
TTLL3	cg03375833	NM_015644.2	0.39	0.75	0.68	0.51	0.66	3	No	701
TLR1	cg03430998	NM_003263.3	0.34	0.56	0.65	0.45	0.44	4	No	645
CHI3L1	cg03625911	NM_001276.1	0.27	0.44	0.62	0.48	0.60	1	No	140
CYB5R2	cg03826976	NM_016229.2	0.11	0.39	0.50	0.17	0.11	11	Yes	612
ARMC2	cg03874127	NM_032131.3	0.44	0.65	0.76	0.57	0.60	6	Yes	490
LRFN4	cg05111110	NM_024036.3	0.57	0.87	0.91	0.84	0.92	11	Yes	305
FOXH1	cg05113558	NM_003923.1	0.47	0.85	0.81	0.71	0.78	8	Yes	625
FANCG	cg05293216	NM_004629.1	0.33	0.69	0.73	0.57	0.71	9	Yes	799
KCNQ1DN	cg05656180	NT_009237.17	0.14	0.32	0.50	0.21	0.15	11	Yes	
LAIR1	cg06238491	NM_002287.2	0.30	0.59	0.67	0.44	0.48	19	No	22
SETX	cg06353948	NM_015046.4	0.47	0.66	0.85	0.76	0.84	9	Yes	644
ICAM3	cg06855803	NM_002162.2	0.39	0.60	0.58	0.47	0.46	19	No	57
CSF1R	cg07260017	NM_005211.2	0.37	0.61	0.69	0.46	0.59	5	No	455
LY6D	cg07572435	NM_003695.2	0.36	0.82	0.75	0.69	0.77	8	No	5
SCARA5	cg07634191	NM_173833.3	0.21	0.50	0.65	0.32	0.31	8	Yes	20
SLC25A10	cg07845392	NM_012140.3	0.23	0.55	0.56	0.26	0.33	17	Yes	1213
NUP50	cg07986773	NM_153645.1	0.26	0.61	0.57	0.44	0.52	22	Yes	1117
GPR62	cg08072716	NM_080865.2	0.33	0.44	0.63	0.43	0.42	3	Yes	295
FLJ30046	cg08504583	NM_144595.2	0.42	0.72	0.77	0.64	0.52	13	No	95
SPAG4L	cg08642068	NM_080675.3	0.56	0.80	0.81	0.74	0.77	20	No	463
ALX4	cg08914623	NT_009237.17	0.09	0.37	0.52	0.15	0.10	11	Yes	

PKP1	cg09009380	NM_001005337.1	0.26	0.51	0.67	0.34	0.41	1	Yes	394
PRTN3	cg09134726	NM_002777.3	0.38	0.70	0.72	0.58	0.66	19	No	97
UNQ467	cg10305797	NM_207392.1	0.18	0.47	0.65	0.48	0.59	19	No	321
SLC35C1	cg10516359	NM_018389.3	0.36	0.83	0.86	0.77	0.86	11	Yes	427
TSCOT	cg10726357	NM_033051.2	0.39	0.65	0.67	0.52	0.53	9	Yes	498
SPOCK2	cg10983208	NM_014767.1	0.06	0.26	0.51	0.09	0.13	10	Yes	447
DDEFL1	cg11155265	NM_017707.2	0.24	0.43	0.49	0.31	0.33	1	Yes	774
SGPP2	cg11300809	NM_152386.2	0.27	0.45	0.59	0.45	0.50	2	Yes	685
TM4SF18	cg11761535	NM_138786.1	0.30	0.66	0.69	0.57	0.56	3	No	388
ARF5	cg12288726	NM_001662.2	0.28	0.63	0.73	0.48	0.56	7	Yes	660
FLJ46358	cg12682367	NM_207439.2	0.27	0.52	0.63	0.51	0.59	13	No	244
PPP1R14A	cg12830694	NM_033256.1	0.57	0.88	0.88	0.79	0.86	19	No	624
RB1	cg13221796	NM_000321.1	0.42	0.69	0.71	0.57	0.68	13	No	992
ADAM12	cg13488201	NM_003474.3	0.06	0.16	0.54	0.16	0.14	10	Yes	258
SCNN1D	cg13587552	NM_002978.2	0.57	0.81	0.84	0.80	0.81	1	No	1135
TRIM29	cg13625403	NM_058193.1	0.43	0.85	0.84	0.71	0.86	11	Yes	353
MPI	cg13828047	NM_002435.1	0.18	0.43	0.55	0.25	0.26	15	Yes	280
ZFR	cg13954292	NM_016107.3	0.31	0.75	0.82	0.65	0.75	5	Yes	679
MAPRE1	cg14303674	NM_012325.1	0.21	0.34	0.54	0.35	0.40	20	Yes	426
HTR3D	cg14483391	NM_182537.2	0.33	0.63	0.70	0.60	0.65	3	No	105
TINAGL1	cg14869028	NM_022164.1	0.12	0.27	0.46	0.22	0.20	1	Yes	1
SEMA3B	cg14911395	NT_022517.17	0.20	0.45	0.75	0.46	0.44	3	Yes	
C1orf35	cg14925024	NM_024319.2	0.27	0.64	0.69	0.51	0.52	1	Yes	683
CEBPG	cg15046693	NM_001806.2	0.21	0.40	0.66	0.44	0.54	19	Yes	733
CDKL1	cg16077929	NM_004196.3	0.37	0.64	0.72	0.54	0.62	14	No	108
DPM1	cg16332224	NM_003859.1	0.55	0.89	0.83	0.85	0.85	20	Yes	1346
TRPM6	cg16509045	NM_017662.3	0.30	0.55	0.60	0.48	0.52	9	No	47
EPAS1	cg17518825	NM_001430.3	0.36	0.63	0.66	0.47	0.51	2	Yes	1102
HOXA11	cg17950095	NM_005523.4	0.15	0.43	0.58	0.32	0.34	7	Yes	130
ULK1	cg18081313	NM_003565.1	0.48	0.78	0.83	0.70	0.69	12	Yes	1087
APC2	cg18133957	NM_005883.2	0.33	0.60	0.76	0.47	0.36	19	Yes	345
C19orf19	cg18493147	NM_182577.1	0.44	0.81	0.84	0.62	0.78	19	Yes	538
LASS2	cg18611122	NM_013384.3	0.45	0.65	0.77	0.49	0.41	1	Yes	577
RABGEF1	cg18884741	NM_014504.1	0.36	0.76	0.75	0.65	0.77	7	No	923
ALDH1A3	cg19224278	NM_000693.1	0.17	0.53	0.61	0.40	0.30	15	Yes	1044
ZNF38	cg19370284	NM_145914.2	0.44	0.76	0.83	0.74	0.73	7	No	411
C20orf24	cg19562969	NM_018840.2	0.28	0.61	0.66	0.46	0.55	20	Yes	585
FLJ30046	cg19782598	NM_144595.2	0.36	0.67	0.69	0.50	0.46	13	No	204
TNFAIP2	cg20368904	NM_006291.2	0.39	0.75	0.80	0.55	0.53	14	Yes	424
LRRC17	cg20442697	NM_005824.1	0.23	0.59	0.70	0.33	0.29	7	No	20
PF4V1	cg20615832	NM_002620.1	0.13	0.53	0.52	0.29	0.26	4	Yes	159

PAK2	cg21030598	NM_002577.3	0.26	0.48	0.52	0.36	0.35	3	Yes	490
ZNF619	cg21942438	NM_173656.1	0.52	0.68	0.72	0.58	0.68	3	No	546
GFAP	cg21944455	NM_002055.2	0.35	0.67	0.70	0.52	0.50	17	No	1401
VTCN1	cg22424746	NM_024626.1	0.32	0.54	0.66	0.57	0.69	1	No	234
MGC40222	cg22572779	NM_152738.1	0.26	0.68	0.68	0.58	0.70	6	No	13
C21orf124	cg23178308	NM_032920.1	0.59	0.37	0.10	0.22	0.18	21	Yes	4
ALDH1A3	cg23191950	NM_000693.1	0.29	0.68	0.74	0.52	0.48	15	Yes	987
KCNAB1	cg23873703	NM_003471.2	0.30	0.82	0.70	0.64	0.68	3	Yes	277
FBXO17	cg24010336	NM_024907.5	0.29	0.39	0.62	0.36	0.37	19	Yes	878
MLZE	cg24243265	NM_031415.1	0.45	0.68	0.80	0.72	0.76	8	No	717
MIA	cg25152942	NM_006533.1	0.22	0.57	0.61	0.39	0.45	19	No	298
GFPT1	cg25195673	NM_002056.1	0.28	0.64	0.66	0.50	0.51	2	Yes	620
FGF19	cg26096837	NM_005117.2	0.29	0.57	0.79	0.47	0.39	11	Yes	868
SMCR7	cg26771272	NM_139162.2	0.36	0.50	0.76	0.63	0.72	17	Yes	686
SEMA3B	cg26918728	NT_022517.17	0.43	0.81	0.85	0.72	0.76	3	Yes	
TTLL11	cg27218220	NM_194252.1	0.45	0.81	0.83	0.70	0.80	9	Yes	833
KCNQ1	cg27491887	NT_009237.17	0.20	0.47	0.61	0.37	0.40	11	Yes	
