# Supplementary material for: Integrated Genomic and Epigenomic Analysis of Breast Cancer Brain Metastasis
Source: PLoS One. 2014 Jan 29;9(1):e85448. doi: 10.1371/journal.pone.0085448 (PMC3906004; doi:10.1371/journal.pone.0085448)
Supplement: File S1 — Supporting figures and tables. Figure S1: Combined Network for Upstream Analysis of FOXM1 and TBX2. The downstream genes connected to FOXM1 and TBX2 were illustrated as a network in IPA. The mRNA expression ratios are listed below the gene nodes. The legend within figure describes the node and edge color keys. Figure S2: Word Cloud Analysis of Cluster Enrichments. We have used word clouds to visually summarize the textual results from the enrichment analysis of each gene cluster as observed in Figure 3. The results were generated using www.wordle.net web resource. The larger the word, the more times it is mentioned in the enrichment categories. Supplementary Tables in File S1. Table S1a. Table S1b. Table S2. Table S3a. Table S3b. Table S4a. Figure S1. Table S4b. Table S5a–b. Table S6a–b. Table S7. Table S8a–f. Table S9a–f. Figure S2. Table S10. Table S11a–c. Table S11d. Table S12. Table S13. Table S14. (ZIP) [file pone.0085448.s001.zip › Supplementary Table S12.pdf]

Supplemetnary Table 12. CpG Island Hypomethylator Phenotype (CIHMP) in basal-like tumors is comprised of 15 CpG Island loci.

Gene Symbol	Illumina TargetID	GeneBank Accession	CHR*	CpG Island	Distance to transcription start site
ALDH1A3	cg19224278	NM_000693.1	15	Yes	1044
ALDH1A3	cg23191950	NM_000693.1	15	Yes	987
ARF5	cg12288726	NM_001662.2	7	Yes	660
C1orf35	cg14925024	NM_024319.2	1	Yes	683
FANCG	cg05293216	NM_004629.1	9	Yes	799
HOXA11	cg17950095	NM_005523.4	7	Yes	130
KCNAB1	cg23873703	NM_003471.2	3	Yes	277
PKP1	cg09009380	NM_001005337.1	1	Yes	394
SCARA5	cg07634191	NM_173833.3	8	Yes	20
SEMA3B	cg26918728	NT_022517.17	3	Yes	
SLC35C1	cg10516359	NM_018389.3	11	Yes	427
TNFAIP2	cg20368904	NM_006291.2	14	Yes	424
TRIM29	cg13625403	NM_058193.1	11	Yes	353
TTLL11	cg27218220	NM_194252.1	9	Yes	833
ZFR	cg13954292	NM_016107.3	5	Yes	679

* CHR = chromosome
