# Supplementary material for: Integrated Genomic and Epigenomic Analysis of Breast Cancer Brain Metastasis
Source: PLoS One. 2014 Jan 29;9(1):e85448. doi: 10.1371/journal.pone.0085448 (PMC3906004; doi:10.1371/journal.pone.0085448)
Supplement: File S1 — Supporting figures and tables. Figure S1: Combined Network for Upstream Analysis of FOXM1 and TBX2. The downstream genes connected to FOXM1 and TBX2 were illustrated as a network in IPA. The mRNA expression ratios are listed below the gene nodes. The legend within figure describes the node and edge color keys. Figure S2: Word Cloud Analysis of Cluster Enrichments. We have used word clouds to visually summarize the textual results from the enrichment analysis of each gene cluster as observed in Figure 3. The results were generated using www.wordle.net web resource. The larger the word, the more times it is mentioned in the enrichment categories. Supplementary Tables in File S1. Table S1a. Table S1b. Table S2. Table S3a. Table S3b. Table S4a. Figure S1. Table S4b. Table S5a–b. Table S6a–b. Table S7. Table S8a–f. Table S9a–f. Figure S2. Table S10. Table S11a–c. Table S11d. Table S12. Table S13. Table S14. (ZIP) [file pone.0085448.s001.zip › Supplementary Table S13.pdf]

Supplementary Table 13. Selected concepts (genes or gene complexes) with alterations in both copy number and gene expression.

Concepts are listed vertically and 11 samples are listed horizonatally. Red and green boxes indicate samples with the specific alteration.

Concept	BBM1	BBM5	BBM6	BBM9	BBM10	BBM14	BBM15	BBM16	BBM42	BBM43	BBM44
**Amplified and Overexpressed**											
AKT1							■		■		
AURKA	■		■	■							
ATAD2		■	■	■	■						
Cbx genes	■		■	■	■		■		■		■
BRAF	■		■				■				
DNMT1 and DNMT3B	■		■				■				
DERL1		■		■	■		■		■		
ER Stress Modulators (eIF2AK3 and ATF6)		■		■	■		■		■		
ESR2									■		
FASN	■		■	■							
FN14					■		■				
Gamma-Secretase complex		■	■	■			■				
HIF1							■		■		
Histone Cluster	■	■	■	■	■		■			■	
IGF1R	■										
MCL1			■								
NEK2		■	■	■	■		■		■		
PPFIA1					■			■			
PRL	■										
RAF1				■			■				
RAG1AP1				■							
SRD5A2		■	■	■	■		■				
SUMO2										■	
TYMS			■	■							
UBA1								■			
VEGFA							■				
WNT3A		■							■		
WNT9A							■		■		
**Deleted and downregulated**											
ATM	■		■						■		■
CDKN2A			■								
CDKN2B	■		■								
CRYAB	■		■						■		■
CTNNA3		■		■							
HSPB2	■		■						■		■
MSH6							■				
RB				■							
RPS6KA3				■	■						
TCF4						■					
