# Supplementary material for: Integrated Genomic and Epigenomic Analysis of Breast Cancer Brain Metastasis
Source: PLoS One. 2014 Jan 29;9(1):e85448. doi: 10.1371/journal.pone.0085448 (PMC3906004; doi:10.1371/journal.pone.0085448)
Supplement: File S1 — Supporting figures and tables. Figure S1: Combined Network for Upstream Analysis of FOXM1 and TBX2. The downstream genes connected to FOXM1 and TBX2 were illustrated as a network in IPA. The mRNA expression ratios are listed below the gene nodes. The legend within figure describes the node and edge color keys. Figure S2: Word Cloud Analysis of Cluster Enrichments. We have used word clouds to visually summarize the textual results from the enrichment analysis of each gene cluster as observed in Figure 3. The results were generated using www.wordle.net web resource. The larger the word, the more times it is mentioned in the enrichment categories. Supplementary Tables in File S1. Table S1a. Table S1b. Table S2. Table S3a. Table S3b. Table S4a. Figure S1. Table S4b. Table S5a–b. Table S6a–b. Table S7. Table S8a–f. Table S9a–f. Figure S2. Table S10. Table S11a–c. Table S11d. Table S12. Table S13. Table S14. (ZIP) [file pone.0085448.s001.zip › Supplementary Table S14.pdf]

Supplementary Table 14.Enrichment of biological pathways using Ingenuity Systems Analysis that have alterations in both DNA methylation and gene expression. Pathways are listed vertically and 11 samples are listed horizonatally. Red boxes indicate which samples have which alterations. Genes within a pathway can be either hyper-or hypomethylated.

Top Canonical Pathways	BBM 1	BBM 5	BBM 6	BBM 9	BBM10	BBM 14	BBM 15	BBM 16	BBM 42	BBM 43	BBM 44
Acute Phase Response Signaling				■							
Alanine Biosynthesis II				■							
Alanine Degradation III				■							
AMPK signaling											■
Atherosclerosis Signaling										■	
Bladder Cancer Signaling	■						■				
Cardiomyocyte Differentiation via BMP Receptors			■								
Cellular Effects of Sildenafil			■								
cholesterol biosynthesis I							■				
cholesterol biosynthesis II							■				
Chondroitin Sulfate Biosynthesis Late Stage		■									
Dermatan Sulfate Biosynthsis Late Stage		■									
Dopamine Degradation								■			
FXR/RXR Activation		■									
G-Protein Coupled Receptor Signaling	■										
Glucocorticoid Receptor Signaling				■							
glycine biosynthesis								■			
Glycine Cleavage Complex					■						
Heparan Sulphate Biosynthesis						■					
Heparan Sulphate Biosynthesis Late Stage						■					
Hepatic Cholestasasis	■										
Hepatic Fibrosis/Hepatic Stellate Cell Activation	■								■	■	
IL-8 Signaling	■		■						■	■	■
inhibition of MMP										■	
Leukocyte Extravasation Signaling	■										
LPS/IL1 Mediated inhibition of RXR function						■					
LXR/RXR activation										■	
Natural Killer Cell Signaling							■				
Pregnenolone biosynthesis									■		
Protein Kinase A Signaling		■									
Protein Ubiquitination Pathway					■						
RhoA Signaling			■								
RhoGDI Signaling			■								
role of ostoblasts, osteoclasts and chondrocytes in rheumatoid arthritis											■
Role of pattern recognition receptors in bacteria and viruses							■				
Role of tissue factor in cancer									■		
Serotonin Degradation								■			
Sorbitol degradation								■			
Sulfate Activation for Sulfonation				■							
Thyroamine and Iodothyroamine metabolism					■	■					■
Thyroid hormone metabolism 1					■	■					■
Thyroid hormone metabolism 2					■						
tRNA splicing		■									
UDP-D-xylose and UDP-D glucuronate biosynthesis								■			
Wnt/B-catenin signaling											■
**Pathways specifically associated with Hypomethylated and upregulated genes**	**p-value**										

Role of cytokinesis in mediating communication between immune cells	5.23E-04
DNA methylation and transcriptional repression signaling	1.56E-03
Communication between innate and adaptive immune cells	2.50E-03
Hepatic cholestasis	3.23E-03
FXR/RXR Activation	1.75E-03
**Pathways specifically associated with Hypermethylation and Downregulation**	
G-Protein Coupled Receptor Signaling	1.67E-06
Protein Kinase A Signaling	1.09E-05
IL-8 Signaling	1.41E-05
cAMP-mediated signaling	1.47E-05
Macropinocytosis signaling	8.41E-05
