# Supplementary material for: Integrated Genomic and Epigenomic Analysis of Breast Cancer Brain Metastasis
Source: PLoS One. 2014 Jan 29;9(1):e85448. doi: 10.1371/journal.pone.0085448 (PMC3906004; doi:10.1371/journal.pone.0085448)
Supplement: File S1 — Supporting figures and tables. Figure S1: Combined Network for Upstream Analysis of FOXM1 and TBX2. The downstream genes connected to FOXM1 and TBX2 were illustrated as a network in IPA. The mRNA expression ratios are listed below the gene nodes. The legend within figure describes the node and edge color keys. Figure S2: Word Cloud Analysis of Cluster Enrichments. We have used word clouds to visually summarize the textual results from the enrichment analysis of each gene cluster as observed in Figure 3. The results were generated using www.wordle.net web resource. The larger the word, the more times it is mentioned in the enrichment categories. Supplementary Tables in File S1. Table S1a. Table S1b. Table S2. Table S3a. Table S3b. Table S4a. Figure S1. Table S4b. Table S5a–b. Table S6a–b. Table S7. Table S8a–f. Table S9a–f. Figure S2. Table S10. Table S11a–c. Table S11d. Table S12. Table S13. Table S14. (ZIP) [file pone.0085448.s001.zip › Supplementary Table S1a.pdf]

Supplementary Table 1a. List amplified regions and associated genes as predicted by GISTIC
The q-value of the peak region are shown.
Residual q-values represent the q-value of the peak of the region after removing amplifications or deletions that overlap other more significant peak regions in the same chromosome.
Wide peak boundaries are most likely to contain the targeted genes.

**cytoband**	1q22	1q44	3p26.3	5p15.33	6p23	6p22.2	6q27	7p15.2	7q36.1	8q24.22	9p24.2
**q value**	0.0014259	0.00048228	0.044029	0.0075321	0.0030265	0.013358	0.042284	0.133	0.12085	0.00048228	0.012919
**residual q value**	0.020848	0.00048228	0.044029	0.0075321	0.0030265	0.24701	0.042284	0.1772	0.12085	0.00048228	0.012919
**wide peak boundaries**	chr1:153173016-153476510	chr1:247116757-247249719	chr3:76073-242115	chr5:1-2541000	chr6:14225271-14765899	chr6:23626844-25542600	chr6:166855664-170899992	chr7:27162657-27201467	chr7:142691612-150864978	chr8:131846077-133227088	chr9:3827029-3847280
**genes in wide peak**	hsa-mir-92b	ZNF692	CHL1	NDUFS6	CD83	GPLD1	CCR6	hsa-mir-196b	hsa-mir-671	ADCY8	GLIS3
	CKS1B	PGBD2		SDHA		ALDH5A1	GPR31	HOXA7	ABP1	KCNQ3	
	EFNA1			SLC6A3		C6orf32	KIF25	HOXA9	CASP2	EFR3A	
	EFNA3			SLC9A3		KIAA0319	MLLT4	HOXA10	CDK5	OC90	
	EFNA4			TERT		GMNN	PDCD2	HOXA11	CLCN1		
	GBA			TRIP13		DCDC2	PSMB1		EPHA1		
	MTX1			PDCD6		TTRAP	RPS6KA2		EZH2		
	MUC1			SLC12A7		LRRC16A	TBP		GBX1		
	SHC1			TPPP		THEM2	TCP10		KCNH2		
	THBS3			EXOC3		MRS2	TCTE3		NOS3		
	ADAM15			IRX4		C6orf62	THBS2		RARRES2		
	PMVK			CEP72		NRSN1	RNASET2		RHEB		
	ZBTB7B			AHRR		KAAG1	FGFR1OP		SLC4A2		
	DPM3			MRPL36			DLL1		SMARCD3		
	LENEP			BRD9			UNC93A		ZYX		
	RAG1AP1			ZDHHC11			PHF10		ARHGEF5		
	PBXIP1			LPCAT1			C6orf70		ZNF212		
	TRIM46			CLPTM1L			SMOC2		ZNF282		
	FLAD1			NKD2			FRMD1		CUL1		
	PYGO2			LOC116349			TTLL2		ACCN3		
	DCST2			CCDC127			FAM120B		PDIA4		
	DCST1			PLEKHG4B			DACT2		FAM131B		
	KRTCAP2			SLC6A19			WDR27		FAM115A		
				SLC6A18			C6orf120		ABCF2		
				LOC389257			C6orf124		FASTK		
									ABCB8		
									SSPO		
									CNTNAP2		
									GIMAP2		
									OR2F1		
									TPK1		
									ZNF777		
									TMEM176B		
									REPIN1		
									NUB1		
									CSGlcA-T		
									GIMAP4		
									GIMAP5		
									TMEM176A		
									ZNF398		
									LRRC61		
									ZNF767		
									TMUB1		
									KRBA1		
									C7orf29		
									CENTG3		
									TMEM139		
									NOBOX		
									OR2A14		
									OR6B1		
									OR2F2		
									ZNF786		
									ASB10		
									GIMAP8		
									CRYGN		
									ZNF425		
									ZNF783		
									ZNF746		
									ATP6V0E2		
									GIMAP7		
									ZNF467		
									GIMAP1		
									C7orf33		
									TAS2R41		
									FAM139A		
									ZNF775		
									ATG9B		
									TAS2R60		
									CTAGE6		

									OR2A12		
									OR2A1		
									WDR86		
									OR2A25		
									OR2A5		
									OR2A7		
									OR2A42		
									LOC402715		
									LOC441294		
									OR2A2		
									FLJ43692		
									GIMAP6		
									LOC643641		
									LOC730647		

**cytoband**	10p14	11q13.3	12p13.33	13q34	14q32.33	15q26.3	16q12.2	17q12	19q12	20p12.2	20q13.33
**q value**	0.11289	0.07652	0.043208	0.09071	0.057401	0.073314	0.019449	0.037234	0.034376	0.19322	0.041429
**residual q value**	0.11289	0.07652	0.043208	0.09071	0.057401	0.073314	0.019449	0.037234	0.034376	0.19322	0.041429
**wide peak boundaries**	chr10:11084680-11541076	chr11:70451058-72151160	chr12:93150-200238	chr13:11301803	chr14:104268612-1044268	chr15:97006929-97528929	chr16:54399891-54424733	chr17:34982340-35146598	chr19:35621806-35846011	chr20:10230049-10785711	chr20:60312573-61077463
**genes in wide peak**	CUGBP2	hsa-mir-139	SLC6A12	ATP4B	AKT1	IGF1R	CES1	ERBB2	ZNF536	JAG1	hsa-mir-133a-2
		PHOX2A	SLC6A13	GAS6	SIVA1	DMN		NEUROD2		SNAP25	COL9A3
		DHCR7	IQSEC3	LAMP1	ADSSL1	TTC23		PNMT		MKKS	LAMA5
		FOLR1		GRK1	KIAA0284	LOC145814		TCAP		C20orf94	NTSR1
		FOLR2		TFDP1				STARD3			RPS21
		FOLR3		CDC16				PPP1R1B			TCFL5
		INPPL1		RASA3				C17orf37			ADRM1
		KRTAP5-9		TMCO3				PERLD1			OGFR
		NUMA1		DCUN1D2							DIDO1
		PDE2A		UPF3A							SLCO4A1
		IL18BP		GRTP1							C20orf11
		STARD10		ADPRHL1							C20orf20
		C11orf51		ZNF828							C20orf59
		C11orf59		FAM70B							CABLES2
		NADSYN1									C20orf166
		FAM86C									GATA5
		RNF121									C20orf151
		KRTAP5-8									C20orf200
		CLPB									
		CENTD2									
		LRRC51									
		DEFB108B									
		KRTAP5-10									
		KRTAP5-7									
		KRTAP5-11									
