# Supplementary material for: Integrated Genomic and Epigenomic Analysis of Breast Cancer Brain Metastasis
Source: PLoS One. 2014 Jan 29;9(1):e85448. doi: 10.1371/journal.pone.0085448 (PMC3906004; doi:10.1371/journal.pone.0085448)
Supplement: File S1 — Supporting figures and tables. Figure S1: Combined Network for Upstream Analysis of FOXM1 and TBX2. The downstream genes connected to FOXM1 and TBX2 were illustrated as a network in IPA. The mRNA expression ratios are listed below the gene nodes. The legend within figure describes the node and edge color keys. Figure S2: Word Cloud Analysis of Cluster Enrichments. We have used word clouds to visually summarize the textual results from the enrichment analysis of each gene cluster as observed in Figure 3. The results were generated using www.wordle.net web resource. The larger the word, the more times it is mentioned in the enrichment categories. Supplementary Tables in File S1. Table S1a. Table S1b. Table S2. Table S3a. Table S3b. Table S4a. Figure S1. Table S4b. Table S5a–b. Table S6a–b. Table S7. Table S8a–f. Table S9a–f. Figure S2. Table S10. Table S11a–c. Table S11d. Table S12. Table S13. Table S14. (ZIP) [file pone.0085448.s001.zip › Supplementary Table S1b.pdf]

Supplementary Table 1b. List of deleted regions and associate genes as predicted by GISTIC
The q-value of the peak region are shown.
Residual q-values represent the q-value of the peak of the region after removing amplifications or deletions that overlap other more significant peak regions in the same chromosome.
Wide peak boundaries are most likely to contain the targeted genes.

**cytoband**	1p36.11	1p34.1	1p13.3	2p23.3	2q23.3	2q37.3	3p14.3	4p16.3	4p15.1	4q21.23	5q22.2
**q value**	0.094604	0.066727	0.091628	0.095768	0.085181	0.095768	0.094604	0.19174	0.081284	0.22272	0.05738
**residual q value**	0.10292	0.066727	0.095768	0.095768	0.085181	0.095768	0.094604	0.21869	0.081284	0.24728	0.05738
**wide peak boundaries**	chr1:27176936-28679201	chr1:45400022-46106422	chr1:109311065-109505627	chr2:23925363-24087622	chr2:151840464-213657373	chr2:241973862-242085433	chr3:57391545-57723346	chr4:29588-30731	chr4:31023997-34509894	chr4:86709687-88981446	chr5:112082644-112382383
**genes in wide peak**	EYA3	IPP	TAF13	ATAD2B	hsa-mir-561	FARP2	ARF4	[ZNF595]	[PCDH7]	DMP1	APC
	FGR	MUTYH	WDR47	UBXD4	hsa-mir-10b	STK25	SLMAP			DSPP	SRP19
	IFI6	NASP	C1orf119	LOC388931	hsa-mir-933		PDE12			IBSP	REEP5
	GPR3	PRDX1	KIAA1324		ACADL		FAM116A			AFF1	DCP2
	PPP1R8	AKR1A1			ACVR1		DNAH12L			MAPK10	
	PTAFR	TESK2			AOX1					PTPN13	
	RPA2	MAST2			ATP5G3					SPARCL1	
	SLC9A1	MMACHC			BMPR2					HSD17B11	
	FCN3	TMEM69			CACNB4					NUDT9	
	MAP3K6	ZSWIM5			CASP8					MEPE	
	C1orf38	GPBP1L1			CASP10					KLHL8	
	WASF2	HPDL			CD28					ARHGAP24	
	DNAJC8	TOE1			CHN1					C4orf36	
	WDTC1	LOC126661			CHRNA1					SLC10A6	
	STX12	CCDC17			CLK1					HSD17B13	
	AHDC1				COL3A1						
	SMPDL3B				COL5A2						
	MED18				CPS1						
	XKR8				CREB1						
	PHACTR4				ATF2						
	SESN2				CRYGA						
	C1orf160				CRYGB						
	SYTL1				CRYGC						
	ATPIF1				CRYGD						
	FAM46B				CTLA4						
	FAM76A				DLX1						
	LOC388610				DLX2						
	CD164L2				DYNC1I2						
					DPP4						
					EEF1B2						
					ERBB4						
					FAP						
					FRZB						
					GAD1						
					GALNT3						
					GCG						
					MSTN						
					GLS						
					GPD2						
					GPR1						
					GRB14						
					HOXD1						
					HOXD3						
					HOXD4						
					HOXD8						
					HOXD9						
					HOXD10						
					HOXD11						
					HOXD12						
					HOXD13						
					HSPD1						
					HSPE1						
					IDH1						
					INPP1						
					ITGA6						
					ITGA4						
					ITGAV						
					ITGB6						
					KCNJ3						
					LRP2						
					LY75						
					MAP2						
					MYO1B						
					MYL1						
					NAB1						
					NEB						
					NDUFB3						
					NDUFS1						
					NEUROD1						

NFE2L2
NR4A2
ORC2L
PDE1A
PDK1
PLCL1
PMS1
PTH2R
RBMS1
RPE
SCN1A
SCN2A
SCN3A
SCN7A
SCN9A
SP3
SSB
SSFA2
STAT1
STAT4
TFPI
TNFAIP6
TTN
SUMO1
WIPF1
FZD5
FZD7
SDPR
PKP4
HAT1
AGPS
PRKRA
SLC25A12
KLF7
ABCB11
B3GALT1
ADAM23
NRP2
CFLAR
NMI
STK17B
GTF3C3
PPIG
CIR
PSCDBP
BZW1
TLK1
CD302
TANK
ABI2
DHRS9
CALCRL
PSMD14
STAM2
LANCL1
KBTBD10
UBE2E3
MTX2
TBR1
NCKAP1
RAPGEF4
GALNT5
IKZF2
COBLL1
FASTKD2
PLA2R1
SATB2
SF3B1
TMEFF2
GCA
MOBKL3
GORASP2
LOC26010
ARL5A
HIBCH
STK39
METTL5

OLA1
ICOS
BAZ2B
SLC40A1
PDE11A
GULP1
NOP5/NOP58
FKBP7
ZAK
PPIL3
DNAJC10
ASNSD1
FLJ20160
FLJ20309
FIGN
RIF1
ALS2CR2
PRPF40A
WDR12
ZC3H15
DNAH7
RPRM
SLC39A10
SLC4A10
CYP20A1
SPC25
ERMN
HECW2
ALS2
ZDBF2
KIAA1604
G6PC2
MPP4
NIF3L1
IFIH1
OSGEPL1
March7
OBFC2A
RAPH1
PFTK2
ALS2CR4
NBEAL1
PLEKHA3
TRAK2
BOLL
C2orf47
SCRN3
FASTKD1
ALS2CR8
TTC21B
METTL8
CYBRD1
FAM130A2
PGAP1
C2orf37
COQ10B
KIAA1715
CDCA7
WDR75
MGC13057
TANC1
KCNH7
SESTD1
ANKRD44
ZNF804A
TTC30A
DAPL1
MARS2
ORMDL1
FMNL2
GALNT13
OSBPL6
NOSTRIN
DIRC1
PARD3B
NUP35
XIRP2

C2orf60
RBM45
BBS5
LOC129881
ICA1L
RFTN2
ACVR1C
UBR3
KCTD18
ALS2CR12
CPO
MDH1B
CCDC148
MYO3B
DUSP19
ANKAR
TTC30B
ALS2CR13
C2orf67
ZSWIM2
ZNF385B
ARL6IP6
FAM119A
CCNYL1
KLHL23
PPP1R1C
SGOL2
ALS2CR11
SLC38A11
WDSUB1
UPP2
GPR155
KIAA1946
PIP5K3
LOC200726
FLJ38973
HNRPA3
LASS6
MAP1D
FLJ39660
CCDC141
FAM126B
C2orf21
EVX2
CERKL
SP5
PLEKHM1L
LOC389073
DYTN
FLJ44048
C2orf66
PHOSPHO2
DFNB59
LOC644820

**cytoband**	6q23.2	7p22.3	7p22.1	7q34	8p22	8q12.1	8q22.2	9p21.3	10q21.3	10q23.1	10q25.2
**q value**	0.089318	0.052492	0.085181	0.12301	0.052492	0.094604	0.22272	0.094604	0.094604	0.0023202	3.27E-18
**residual q value**	0.089318	0.052492	0.085181	0.12301	0.052492	0.12696	0.22272	0.094604	0.10561	0.034333	3.27E-18
**wide peak boundaries**	chr6:134631702-13464507	chr7:167510-178407	chr7:6074162-6418400	chr7:142535937-14841664	chr8:18304067-19046553	chr8:48145850-58101645	chr8:99143259-101056786	chr9:20597135-233	chr10:69976224-70388837	chr10:82869427-82885456	chr10:114103548-114109302
**genes in wide peak**	[SGK1]	[FAM20C]	RAC1	CASP2	PSD3	CEBPD	hsa-mir-875	hsa-mir-31	DDX21	[SH2D4B]	[ACSL5]
			PSCD3	CLCN1		NPBWR1	COX6C	hsa-mir-491	CCAR1		
			USP42	EPHA1		LYN	KCNS2	CDKN2A	DDX50		
			MGC12966	EZH2		MCM4	STK3	CDKN2B	CXXC6		
			DAGLB	PIP		MOS	HRSP12	IFNA1	STOX1		
				ZYX		OPRK1	POP1	IFNA2			
				ARHGEF5		PENK	RGS22	IFNA4			
				CUL1		PLAG1	NPAL2	IFNA5			
				PDIA4		PRKDC	OSR2	IFNA6			
				FAM131B		RP1	VPS13B	IFNA7			
				FAM115A		RPS20	C8orf47	IFNA8			
				CNTNAP2		SNAI2		IFNA10			
				OR2F1		TCEA1		IFNA13			
				TPK1		UBE2V2		IFNA14			
				TMEM139		RGS20		IFNA16			
				NOBOX		ST18		IFNA17			
				OR2A14		RB1CC1		IFNA21			
				OR6B1		LYPLA1		IFNB1			

OR2F2	KIAA0146	IFNW1
ZNF786	MRPL15	MLLT3
C7orf33	ATP6V1H	MTAP
TAS2R39	SNTG1	KIAA1797
TAS2R40	IMPAD1	KLHL9
TAS2R41	SOX17	DMRTA1
FAM139A	CHCHD7	IFNE1
TAS2R60	EFCAB1	PTPLAD2
CTAGE6	TGS1	
OR2A12	XKR4	
OR2A1	PCMTD1	
GSTK1	TMEM68	
OR2A25	PXDNL	
OR2A5	RDHE2	
OR2A7	FAM150A	
OR2A42	C8orf22	
LOC402715		
LOC441294		
OR2A2		
FLJ43692		
LOC730647		

**cytoband**	11p15.4	11q24.1	12p13.2	12q13.13	12q23.1	13q12.11	14q12	14q31.3	14q32.33	15q15.3	16q23.1
**q value**	0.094604	0.052492	1.31E-08	0.14381	0.13171	0.052492	0.14381	0.077208	0.011915	0.0035682	0.10325
**residual q value**	0.094604	0.052492	1.31E-08	0.18568	0.13171	0.052492	0.17398	0.10292	0.011915	0.0035681	0.10325
**wide peak boundaries**	chr11:9192632-9513182	chr11:122457256-1225220	chr12:11121205-11147674	chr12:48984333-49233632	chr12:97345521-97624785	chr13:19194238-19494798	chr14:30486677-30941408	chr14:87879530-88	chr14:105872361-1058970	chr15:41676220-41817157	chr16:77065642-77375742
**genes in wide peak**	ZNF143	ASAM	PRH1	DIP2B	APAF1	ZMYM2	hsa-mir-624	PTPN21	[TMEM121]	CKMT1B	WWOX
	IPO7		PRR4	LARP4	SLC25A3	ZMYM5	AP4S1	SPATA7		CATSPER2	
	RAB6IP1		TAS2R43		TMPO	PSPC1	HECTD1	ZC3H14		STRC	
	TMEM41B				IKIP		HEATR5A	TTC8		CKMT1A	
							STRN3	EML5			

**cytoband**	17p12	18q21.2	19p13.3	19q13.42	21p13	22q13.2	Xp22.33	Xq24
**q value**	0.052492	0.081284	0.10266	0.23499	0.17586	0.12913	0.00063667	0.052492
**residual q value**	0.052492	0.081284	0.10266	0.23499	0.17586	0.12913	0.00063667	0.24568
**wide peak boundaries**	chr17:14069740-16056403	chr18:46555481-46968493	chr19:2314192-2372817	chr19:59440263-59543424	chr21:1-9975301	chr22:39761308-40497072	chrX:94063-108901	chrX:56594409-118579702
**genes in wide peak**	ADORA2B	SMAD4	TMPRSS9	LILRB2	TPTE	ACO2	[PLCXD1]	hsa-mir-448
	PMP22	ME2		LILRB5		EP300		hsa-mir-652
	NCOR1	MEX3C		LILRA3		XRCC6		hsa-mir-361
	HS3ST3B1	ELAC1		LILRA4		NHP2L1		hsa-mir-325
	TRIM16	MRO		LILRA5		PMM1		hsa-mir-374a
	TTC19					RANGAP1		hsa-mir-421
	ZNF286A					TEF		hsa-mir-223
	TEKT3					TOB2		ABCB7
	ZSWIM7					ZC3H7B		AGTR2
	CDRT15					CSDC2		SLC25A5
	FAM18B2					FAM152B		AR
	CDRT4					L3MBTL2		ARR3
	TBC1D26					PHF5A		ATP7A
	CDRT1					MEI1		ATRX
	FLJ45831					POLR3H		BTK
								CAPN6
								CDX4
								CHM
								COL4A5
								COL4A6
								COX7B
								CSTF2
								CYLC1
								DCX
								TIMM8A
								DIAPH2
								DLG3
								DRP2
								TSC22D3
								EDA
								EFNB1
								ACSL4
								CENPI
								GJB1
								GLA
								CXCR3
								GPR23
								GUCY2F
								HNRPH2
								HTR2C

IGBP1
IL2RG
IL13RA1
IL13RA2
FOXO4
CITED1
MSN
NAP1L2
NAP1L3
NONO
OPHN1
P2RY4
PAK3
PGK1
PHKA1
PIN4
PLP1
PLS3
POU3F4
PRPS1
PSMD10
RPL36A
RPS4X
SH3BGRL
SLC16A2
TAF1
SERPINA7
TSPAN6
TRPC5
ZNF711
ZXDA
IRS4
OGT
FGF16
CLDN2
ZMYM3
TCEAL1
ITM2A
MORF4L2
GPRASP1
STARD8
ARMCX2
HEPH
AMMECR1
MED12
CYSLTR1
PGRMC1
TMSL8
MID2
SLC6A14
VSIG4
ARHGEF9
KCNE1L
KIF4A
IL1RAPL2
ITGB1BP2
NGFRAP1
NOX1
TMEM28
SRPX2
PCDH11X
RPS6KA6
P2RY10
SNX12
RPA4
UBQLN2
TBX22
RNF12
WBP5
RAB9B
LUZP4
PDZD11
CXorf26
ARMCX1
ARMCX3
TAF9B
CPXCR1

CHIC1
NLGN3
TAF7L
SPIN2A
ARMCX6
WDR44
ERCC6L
NUP62CL
GDPD2
TBC1D8B
CXorf57
RBM41
MTMR8
ALG13
BEX1
HDAC8
KIAA1166
NXT2
NXF5
NXF3
NXF2
KLHL4
TEX13B
TEX13A
TEX11
BEX4
TCEAL7
KIAA1210
PCDH19
RGAG1
LRCH2
MAGEE1
TMEM35
EDA2R
CXorf56
DMRTC1
TNMD
PJA1
ARMCX5
NSBP1
RNF128
MORC4
LONRF3
TCEAL4
CXorf34
POF1B
ESX1
BHLHB9
LAS1L
MAGT1
TMEM164
ZMAT1
GPR174
BEX2
SLC7A3
TCEAL3
KLHL13
TGIF2LX
TCEAL8
CHRDL1
RIPPLY1
ACRC
SYTL4
GPRASP2
ATG4A
DACH2
CXorf41
MUM1L1
CXorf39
FAM123B
APOOL
HDX
OTUD6A
UPRT
MAGEE2
DOCK11
SPIN4

TCEAL2
PABPC5
RAB40A
ASB12
AMOT
FAAH2
ZXDB
LOC158830
DGAT2L3
DGAT2L4
ZDHHC15
TCEAL6
H2BFWT
FAM46D
SPIN3
ZCCHC12
CXorf61
SLC25A43
ZCCHC5
NRK
TMEM31
BRWD3
RAB40AL
YIPF6
FAM133A
MGC39900
RGAG4
NHSL2
PABPC1L2A
KIAA2022
BEX5
TCEAL5
VSIG1
ZC3H12B
SATL1
ZCCHC16
LHFPL1
DGAT2L6
RAB41
ZCCHC13
FLJ44635
ARL13A
MCART6
XKRX
FXYD8
GLRA4
PGAM4
SPIN2B
PABPC1L2B
NXF2B
LOC728656
