# Supplementary material for: Integrated Genomic and Epigenomic Analysis of Breast Cancer Brain Metastasis
Source: PLoS One. 2014 Jan 29;9(1):e85448. doi: 10.1371/journal.pone.0085448 (PMC3906004; doi:10.1371/journal.pone.0085448)
Supplement: File S1 — Supporting figures and tables. Figure S1: Combined Network for Upstream Analysis of FOXM1 and TBX2. The downstream genes connected to FOXM1 and TBX2 were illustrated as a network in IPA. The mRNA expression ratios are listed below the gene nodes. The legend within figure describes the node and edge color keys. Figure S2: Word Cloud Analysis of Cluster Enrichments. We have used word clouds to visually summarize the textual results from the enrichment analysis of each gene cluster as observed in Figure 3. The results were generated using www.wordle.net web resource. The larger the word, the more times it is mentioned in the enrichment categories. Supplementary Tables in File S1. Table S1a. Table S1b. Table S2. Table S3a. Table S3b. Table S4a. Figure S1. Table S4b. Table S5a–b. Table S6a–b. Table S7. Table S8a–f. Table S9a–f. Figure S2. Table S10. Table S11a–c. Table S11d. Table S12. Table S13. Table S14. (ZIP) [file pone.0085448.s001.zip › Supplementary Table S2.pdf]

Table 2. List of Differentially Expressed Genes (DEG) between BBM samples and NBn and NBr samples.

Downregulated Genes

Gene Symbol	GenBank Accession number	FC
ABCA8	NM_007168	-5.22
ABCC9	NM_005691///NM_020297///NM_020298	-3.32
ADAM29	NM_014269	-2.17
ADAM30	NM_021794	-2.40
ADAM5P	NR_001448	-5.94
ADAMTS8	NM_007037	-10.76
ADRA2A	NM_000681	-3.97
ADRB2	NM_000024	-4.70
AFAP1L2	NM_001001936///NM_032550	-3.47
AIF1L	NM_031426	-3.90
AKAP12	NM_144497///NM_005100	-5.43
ALDH1A1	NM_000689	-3.78
ANKRD29	NM_173505	-3.49
ANKRD33B	NM_001164440	-2.87
ANKRD35	NM_144698	-3.29
APBA1	NM_001163///BM511013	-4.19
APCDD1	NM_153000	-6.29
AQP6	NM_001652	-4.70
ARHGAP20	NM_020809	-3.28
ARHGAP6	NM_013427///NM_006125	-3.61
ARHGEF6	NM_004840	-2.50
ARMCX1	NM_016608	-3.14
ATOH8	NM_032827	-2.67
AXIN2	NM_004655	-2.69
BACH2	NM_021813	-2.77
BBOX1	NM_003986	-7.39
BCL11A	NM_022893///NM_018014	-4.38
BEND5	NM_024603	-3.34
BHMT2	NM_017614	-4.49
BIVM	NM_017693	-2.31
BMP6	NM_001718	-3.36
BMPER	AB075845///NM_133468	-2.96
C10orf54	NM_022153	-2.35
C10orf72	NM_144984///NM_001031746	-2.51
C12orf12	NM_152638	-3.44

C12orf64	XM_002343203	-5.38
C13orf18	NM_025113///BC032311	-2.14
C14orf105	NM_018168	-4.14
C15orf26	NM_173528	-2.60
C16orf89	NM_152459	-3.36
C18orf2	NR_023925///AF295728	-2.31
C1orf190	NM_001013615	-2.94
C21orf34	NR_027791///NR_027790	-3.54
C22orf23	AK097339///NM_032561	-2.91
C2orf88	NM_001042519	-4.96
C2orf89	NM_001080824	-3.04
C4orf12	NR_015359///AK124663	-3.39
C4orf49	NM_032623	-3.59
C5orf26	NR_015370	-3.16
C5orf27	NR_026936	-2.50
C5orf4	NM_032385	-6.89
C5orf41	NM_153607	-2.39
C6orf204	NM_206921///BC045657///NM_001042475	-2.56
C7orf57	NM_001100159	-4.33
C8orf79	NM_020844	-6.00
CA4	NM_000717	-15.40
CACNA1G	NM_018896///NM_198397	-4.05
CALB2	NM_001740	-6.83
CBFA2T3	NM_005187	-2.90
CCDC3	NM_031455	-2.75
CCND2	NM_001759	-2.92
CDC42EP2	NM_006779	-2.41
CDH23	NM_052836///NM_022124	-2.27
CDKN1C	NM_000076	-5.55
CEP68	NM_015147	-3.15
CGNL1	NM_032866	-2.95
CKMT2	NM_001825	-3.41
CLEC4G	NM_198492	-6.76
CLIP4	NM_024692	-3.75
CNTN1	NM_001843	-4.35
CNTN3	NM_020872	-11.88
CNTN4	NM_175607///NM_175613	-4.55
CNTNAP3	NM_033655	-3.30
COL16A1	NM_001856	-3.22
CRY2	NM_021117	-3.31
CSRP1	NM_004078	-3.12
CTSF	NM_003793	-2.89
CUGBP2	NM_001025077///NM_001025076	-3.97

CYGB	NM_134268	-2.62
CYP26A1	NM_057157	-4.00
CYP2E1	NM_000773	-2.67
DACT2	NM_214462	-6.40
DCDC1	NM_181807	-5.16
DCX	NM_000555	-19.45
DENND2A	NM_015689	-4.64
DKFZP586K1520	AL050153	-4.66
DLC1	NM_024767///NM_182643	-4.10
DLL1	NM_005618	-2.72
DMD	NM_004019///NM_004023///NM_004010	-4.64
DMRT2	NM_006557	-6.00
DNAJC6	NM_014787	-3.69
DPYS	NM_001385	-3.56
DST	NM_020388///NM_001723///AK025142///NM_015548	-3.74
DYNC2H1	NM_001080463	-2.81
EDN3	NM_207032	-24.78
EDNRB	NM_003991	-4.07
EEPD1	AF150438///NM_030636	-3.44
EFCAB1	NM_024593	-18.85
EFCAB6	NM_022785	-3.67
EFHB	NM_144715	-3.40
EID1	NM_014335	-2.41
EMX2	NM_004098	-6.69
EMX2OS	NR_002791	-4.56
EPHA4	NM_004438	-6.11
EPHB1	NM_004441	-8.52
ETV5	NM_004454	-2.75
F13B	NM_001994	-2.94
F3	NM_001993	-2.20
FAM13C	NM_001001971	-3.45
FAM184A	NM_024581	-2.95
FAM59A	NM_022751	-2.89
FBXL7	NM_012304	-3.26
FCRL1	NM_052938	-2.45
FGF2	NM_002006	-8.84
FGF7	NM_002009	-2.66
FHL1	NM_001449	-6.03
FIGN	NM_018086	-2.95
FLJ13197	NR_026804	-4.06
FLJ22536	AK094718///NR_015410	-2.77
FLJ30679	NR_026674	-3.69
FLNC	NM_001458	-2.07

FLRT2	NM_013231	-5.67
FOLH1	NM_001014986///NM_004476	-2.42
FOSB	NM_006732	-4.82
FOXP2	NM_148898///NM_014491	-3.68
FTO	NM_001080432	-2.58
GABRA6	NM_000811	-3.39
GAL3ST1	NM_004861	-2.83
GALNTL1	NM_020692	-10.82
GALNTL2	NM_054110	-3.40
GDF10	NM_004962	-7.90
GFRA2	NM_001495	-3.52
GGTA1	NR_003191	-5.23
GLIS1	NM_147193	-3.08
GLRA3	BG912760///NM_006529	-2.19
GMCL1L	NR_003281	-2.62
GPAM	NM_020918	-3.38
GPD1	NM_005276	-3.35
GPIHBP1	NM_178172	-4.56
GPR101	NM_054021	-3.17
GPR146	NM_138445	-2.50
GREB1	NM_014668///NM_148903	-2.76
GREM2	NM_022469	-12.20
GRK5	NM_005308	-2.23
GRP	NM_002091	-12.83
GSTM2	NM_000848	-3.88
GSTM5	NM_000851	-4.76
GSTT2	NM_000854	-2.58
GYPB	NM_002100	-2.56
HEPH	NM_014799	-2.42
HEY1	NM_001040708	-2.91
HIVEP3	BC080552///NM_024503	-2.42
HLF	NM_002126	-12.52
HSD11B1	NM_181755	-4.10
HSD17B11	NM_016245	-2.14
HSPB2	NM_001541	-3.23
ID4	NM_001546	-3.93
IFNW1	NM_002177	-2.99
IGDCC4	NM_020962	-2.87
IL33	NM_033439	-10.08
IL6	NM_000600	-2.97
IMPG1	NM_001563	-4.79
INPP5D	NM_001017915	-2.40
IRS2	NM_003749	-3.14

ITGA7	NM_002206	-2.76
ITM2A	NM_004867	-7.03
ITPR1	NM_002222	-2.42
JAM2	NM_021219///AK056079	-6.36
JAM3	NM_032801	-3.20
KANK1	NM_153186	-2.31
KANK3	NM_198471	-3.05
KCNJ2	NM_000891	-2.54
KCTD16	NM_020768	-6.27
KIAA1377	NM_020802	-2.39
KIAA1462	NM_020848	-2.98
KL	NM_004795	-16.66
KLF12	NM_007249	-2.55
KLF9	NM_001206	-2.71
KLHL29	NM_052920	-5.82
KLK7	NM_005046	-9.38
KRT1	NM_006121	-3.01
LCA5	NM_181714	-2.06
LDB2	NM_001290	-4.49
LGI4	NM_139284	-2.73
LHX5	NM_022363	-4.48
LHX6	NM_014368	-3.82
LIAS	NM_006859	-2.18
LIFR	NM_002310	-6.60
LIPE	NM_005357	-5.04
LOC100127983	XM_001722508	-3.20
LOC100128164	NR_027622	-7.09
LOC100131283	XM_001716948	-7.79
LOC100132733	XM_001715342	-3.55
LOC100240735	NR_026658	-2.32
LOC100292626	BC042038	-2.83
LOC100293390	XM_002346092	-3.08
LOC134466	NR_026867	-3.61
LOC283481	XM_001128325	-3.77
LOC283588	XR_041539	-2.20
LOC284232	NR_027995	-3.77
LOC339524	NR_026989///NR_026985	-6.60
LOC390595	NM_001163692	-2.67
LOC390940	XM_001129773	-2.46
LOC399959	NR_024430	-4.08
LOC400794	NR_026744	-2.76
LOC541473	NR_003602	-3.88
LOC55908	NM_018687	-2.56

LOC642406	AK024257	-2.45
LOC644538	NM_001163438	-2.89
LOC645277	XM_928321	-2.72
LOC646548	XR_019089	-3.63
LOC651721	BC026225	-3.54
LPAR4	NM_005296	-3.64
LPCAT2	NM_017839	-2.12
LST-3TM12	NM_001009562	-5.32
LZTS1	NM_021020	-3.50
MAOA	NM_000240	-2.62
MAOB	NM_000898	-3.95
MCTP1	NM_024717	-4.39
ME3	NM_001014811	-3.47
MEIS2	NM_170676///NM_170677	-3.75
METTL7A	NM_014033	-3.61
MGC4859	BC002644	-2.75
MGLL	NM_007283	-2.81
MMP16	AL136588///NM_022564///NM_005941	-6.08
MOBKL2B	NM_024761	-4.63
MPPED2	NM_001584	-2.54
MRAS	NM_012219	-2.61
MRVI1	NM_130385	-4.34
MSRA	NM_012331	-2.51
MT1E	NM_175617	-2.91
MUPCDH	NM_031264///NM_021924	-2.30
MYBPC1	NM_206819	-18.66
MYH3	NM_002470	-2.20
MYOM1	NM_003803	-2.98
N4BP2L1	NM_052818	-3.01
NBL1	NM_182744	-2.59
NCALD	NM_001040630	-8.76
NEUROG2	NM_024019	-2.92
NFIA	NM_005595///NM_001134673	-4.28
NFIX	NM_002501	-2.87
NPM2	NM_182795	-3.32
NPPC	NM_024409	-3.88
NPY1R	NM_000909	-6.25
NR2F1	NM_005654	-3.81
NR4A1	NM_002135	-2.22
NRG1	NM_013960///NM_013962///NM_013959///NM_004495	-3.43
NRG2	NM_013982///NM_004883	-4.49
NTN5	BC021210///NM_145807	-2.34
NUDT9P1	NR_002779	-2.77

ODZ2	NM_001122679	-27.43
P2RX6	AB002058	-2.62
P2RY14	NM_014879	-5.51
PALM2	NM_053016	-4.62
PAMR1	NM_015430	-4.65
PCDH19	NM_020766	-12.83
PDE11A	NM_001077358///NM_016953	-2.58
PDE4B	NM_001037339///NM_001037341	-3.91
PDGFA	NM_002607	-2.92
PDZD4	NM_032512	-2.94
PDZRN3	NM_015009	-4.13
PELI2	NM_021255	-3.26
PENK	NM_006211	-7.32
PER1	NM_002616	-2.73
PHYHD1	NM_174933	-2.34
PID1	NM_017933	-12.08
PIGZ	NM_025163	-2.91
PIK3R1	NM_181523	-5.30
PLAC1L	NM_173801	-3.21
PLCL2	NM_015184	-3.70
PLSCR4	NM_020353	-4.71
PNLDC1	NM_173516	-3.99
PNMT	NM_002686	-5.41
PODN	NM_153703	-3.93
PPAP2A	NM_176895	-2.46
PPAP2B	NM_003713	-6.36
PPP1R12B	NM_032105///NM_032103	-2.23
PPP1R1A	NM_006741	-11.58
PRKAR2B	NM_002736	-3.04
PRKCQ	NM_006257	-3.49
PRKD1	NM_002742	-3.09
PROK2	NM_021935	-3.67
PROX1	NM_002763	-3.41
PTCHD1	NM_173495	-6.89
PTPRB	NM_001109754///NM_002837	-2.28
PTPRE	NM_006504	-3.02
PTPRT	NM_133170	-4.67
PURA	NM_005859	-2.70
PYGO1	NM_015617///AL833463	-3.19
PZP	NM_002864	-3.52
RAB11FIP2	NM_014904	-2.48
RASD1	NM_016084	-4.75
RASL10A	NM_001007279	-14.83

RBMXL3	NM_001145346	-2.42
RBP4	NM_006744	-2.35
RCBTB2	NM_001268	-2.44
RELN	NM_005045	-11.88
RERGL	NM_024730	-7.35
RFX2	NM_000635	-3.58
RFX6	NM_173560	-2.43
RGL1	NM_015149	-4.26
RGMA	NM_020211	-3.06
RGS2	NM_002923	-3.03
RGS5	NM_003617	-2.78
RNASE11	NM_145250	-3.12
RNF113B	NM_178861	-3.41
RNF150	NM_020724	-3.59
ROBO3	NM_022370	-3.45
RORA	BC035094///NM_134260	-2.37
RP11-45B20.2	NM_001007537	-3.77
RPL23AP32	NR_002229	-2.17
RPS6KA5	NM_004755///NM_182398	-2.99
RPTN	NM_001122965	-2.46
RSPO3	NM_032784	-5.77
SARM1	NM_015077	-2.21
SASH1	NM_015278	-3.64
SATB1	NM_002971	-4.60
SCARA3	NM_182826///NM_016240	-3.06
SCN3B	NM_018400	-4.93
SCN4B	NM_174934	-8.06
SCN7A	BQ716254///NM_002976	-7.09
SEMA3D	NM_152754	-15.61
SEMA5A	NM_003966	-4.84
SEMA6A	NM_020796	-3.05
SERPINB9	NM_004155	-2.79
SETBP1	NM_015559	-3.17
SGCD	NM_172244///NM_000337	-4.01
SH3BGRL2	NM_031469	-3.71
SH3BP5	NM_004844	-2.51
SH3PXD2A	NM_014631	-2.90
SHROOM4	NM_020717	-3.21
SIDT2	NM_001040455	-2.23
SLC19A3	NM_025243	-5.42
SLC25A27	NM_004277	-3.84
SLC26A4	NM_000441	-6.78
SLC27A1	NM_198580	-2.55

SLC2A4	NM_001042	-2.98
SLC5A7	NM_021815	-4.40
SLC7A3	NM_032803	-2.43
SLC9A9	NM_173653	-3.30
SMAD9	NM_005905///BM802662	-3.12
SNCAIP	NM_005460	-2.78
SNTG2	NM_018968	-3.43
SORBS1	AK022468///NM_001034954	-4.59
SOX10	NM_006941	-4.13
SPHKAP	NM_030623	-20.97
SPON1	NM_006108	-6.35
SPRY2	NM_005842	-4.28
SSPN	NM_005086	-3.93
ST3GAL3	NM_174963	-3.14
STAC2	NM_198993	-9.22
STARD9	XM_001129290	-3.76
STAT4	NM_003151	-3.10
STATH	NM_003154	-4.11
STEAP2	NM_152999	-2.88
SULT1E1	NM_005420	-4.86
SYNM	NM_145728	-4.53
TAC1	NM_003182	-29.55
TACC1	NM_006283	-3.89
TCF4	NM_003199///NM_001083962	-4.09
TEK	NM_000459	-3.63
TESC	NM_017899	-5.56
THBS4	NM_003248	-6.45
THSD1	NM_018676	-2.82
TLN2	NM_015059	-2.81
TMCC2	NM_014858	-3.68
TMEM100	NM_018286	-4.22
TMEM132C	NM_001136103	-10.96
TMEM47	NM_031442	-4.52
TMEM88	NM_203411	-2.43
TMTC1	NM_175861	-5.27
TNFSF12	NM_003809	-2.96
TPPP2	NM_173846	-4.06
TRIL	NM_014817	-2.74
TSHZ3	NM_020856	-4.86
TSPAN11	NM_001080509	-6.86
TTN	NM_133379///NM_133378	-4.07
UST	NM_005715	-3.11
VWCE	NM_152718	-3.13

WIF1	NM_007191	-4.47
WNK2	NM_006648///AB051547	-2.40
ZBTB16	NM_006006	-5.37
ZBTB20	NM_015642	-3.01
ZC3H6	NM_198581	-2.59
ZC4H2	NM_018684	-2.41
ZCCHC24	NM_153367	-3.04
ZEB1	NM_030751	-2.54
ZFHX4	NM_024721	-6.64
ZFP2	NM_030613	-2.26
ZHX3	NM_015035	-2.98
ZNF132	NM_003433	-2.61
ZNF385D	NM_024697	-8.07
ZNF423	NM_015069	-2.35
ZNF521	NM_015461	-5.34
ZNF667	NM_022103	-4.30
ZNF91	NM_003430	-2.44
**Upregulated Genes**		
AAGAB	NM_024666	3.48
ABHD3	NM_138340	2.98
ACBD3	AB043587///NM_022735	2.27
ACPP	NM_001099	2.39
ADAM8	NM_001109	4.11
ADAM9	NM_003816	2.24
AIDA	NM_022831	2.19
AIM1L	NM_001039775	4.77
AKR1B10	NM_020299	6.27
AKT1	NM_005163	2.19
AMMECR1	NM_015365	3.04
ANKRD22	NM_144590	4.81
AP1S3	NM_001039569	3.70
APOOL	NM_198450	2.33
ARFGEF2	NM_006420	2.36
ARHGAP11A	NM_014783///NM_199357	6.88
ARPC1B	NM_005720	2.86
ARPC4	NM_005718	3.21
ASCL2	NM_005170	2.39
ATAD2	NM_014109	4.93
ATF7	NM_006856///BC042363	2.42
AURKA	NM_198433	2.66
AURKB	NM_004217	2.79

AVL9	NM_015060	2.99
B3GALNT2	NM_152490	3.43
B4GALT4	NM_212543	2.08
BRIP1	NM_032043	3.36
C11orf80	NM_024650	2.49
C12orf28	XM_001718058	3.83
C12orf49	NM_024738	2.51
C14orf142	NM_032490	2.34
C15orf23	NM_001142761	2.30
C15orf42	NM_152259	3.01
C16orf59	NM_025108	2.38
C19orf21	NM_173481	6.37
C1orf106	NM_018265	3.02
C1orf112	NM_018186	2.97
C1orf124	NM_032018///NM_001010984	2.68
C1orf211	BC030279	2.68
C1orf55	NM_152608	3.77
C1orf58	NM_144695	3.71
C1orf74	NM_152485	2.37
C1orf97	NR_026761	2.31
C20orf20	NM_018270	2.48
C20orf24	NM_018840	2.40
C21orf125	NR_026960	2.51
C21orf7	NM_020152	3.91
C2orf29	NM_017546	2.58
C4orf29	NM_001039717	2.20
C8orf33	NM_023080	2.30
C8orf38	AK074467///NM_152416	2.95
C8orf45	NM_001136160	2.67
C8orf51	NR_026785	4.73
C8orf73	NM_001100878	2.58
C8orf76	NM_032847	2.10
C9orf70	NR_026663	2.14
CAMP	NM_004345	14.38
CANT1	NM_138793	2.99
CATSPER1	NM_053054	3.47
CBX3	AL568696///NM_016587	2.54
CCDC109A	NM_138357	2.86
CCDC150	NM_001080539	3.04
CCDC58	NM_001017928	2.43
CCNB2	AK023404///NM_004701	3.45
CCNF	NM_001761	2.30
CCRN4L	NM_012118	2.53

CCT5	NM_012073	2.33
CD9	NM_001769	2.55
CDC20	NM_001255	3.37
CDC25A	NM_001789	3.01
CDC6	NM_001254	4.18
CDCA4	NM_017955	2.74
CDCA5	NM_080668	4.07
CDH15	NM_004933	3.19
CDKN2A	NM_058197	3.23
CDKN3	NM_005192	3.49
CDYL2	NM_152342	3.75
CEACAM3	NM_001815	5.16
CEACAM7	NM_006890	8.96
CEBPG	NM_001806	2.50
CENPE	NM_001813	3.43
CENPI	NM_006733	4.95
CENPL	AK056348///NM_033319	3.76
CEP76	NM_024899	2.26
CHAC2	NM_001008708	2.68
CHML	NM_001821	2.11
CKAP2	NM_018204	2.25
CLSPN	NM_022111	2.75
CNIH4	NM_014184	4.60
CNTD2	NM_024877	3.44
COIL	NM_004645	2.52
COPB2	NM_004766	2.56
COPG	NM_016128	2.56
CS	NM_004077	2.58
CSTB	NM_000100	2.17
CTSE	NM_001910	2.40
CTSL2	NM_001333	3.10
CTTN	NM_005231	2.82
CXADRP2	NR_024387	3.78
CXorf39	NM_207318	2.64
CXorf56	NM_022101	2.25
DAP3	NM_033657	2.01
DARS2	NM_018122	3.94
DCAF13	AF161549///NM_015420	3.54
DCAF17	NM_025000	2.88
DDX58	NM_014314	3.45
DENND1B	NM_144977	2.16
DEPDC1	NM_017779	3.84
DEPDC1B	NM_018369	9.05

DERL1	NM_024295	2.80
DHTKD1	NM_018706	2.37
DLEU2	AF264787	3.61
DNA2	NM_001080449	4.80
DNASE1	NM_005223	2.60
DNASE2	NM_001375	3.04
DNMT3B	NM_175850	3.08
DONSON	NM_017613	3.01
DOPEY2	NM_005128	2.71
DPH3B	NM_080750	2.47
DPM3	NM_018973	2.04
DPP3	NM_130443	2.39
DQX1	NM_133637	5.00
DUS4L	NM_181581	2.17
E2F5	NM_001951	2.47
ECT2	NM_018098	4.92
EIF2C2	NM_012154	2.77
EME1	NM_152463	3.95
ENTPD7	NM_020354	5.36
EPR1	NR_002219	2.98
EPSTI1	AL831953///NM_033255	2.41
ERMP1	NM_024896	3.08
ERO1L	AK024224///NM_014584	3.77
ESM1	NM_007036	5.79
EVPLL	NM_001145127	3.00
EXO1	NM_003686	3.56
EXOSC3	NM_016042	2.61
FAM105B	NM_138348	2.14
FAM136B	NM_001012983	2.42
FAM72D	NM_207418	3.16
FAM83D	NM_030919	11.21
FAM91A1	NM_144963	3.04
FBXL6	NM_012162	2.67
FBXO22	NM_147188///NM_012170	2.10
FKBPL	NM_022110	2.81
FLJ13744	AK023806	3.36
FLJ31813	AK056375	2.67
FLVCR1	NM_014053///AK090739	2.50
FLVCR2	NM_017791	5.25
FN1	NM_212482///NM_054034	4.86
FOXM1	NM_202002	6.20
FURIN	NM_002569	2.26
FUT2	NM_000511	2.62

G2E3	NM_017769	2.22
GABPB1	NM_002041///NM_005254	3.38
GABPB2	NM_144618	2.61
GAS2L3	NM_174942///BX649059	2.14
GCH1	NM_000161	2.83
GCNT3	NM_004751	6.80
GDAP2	NM_017686	2.82
GFPT1	NM_002056	2.85
GGCT	NM_024051	2.41
GGH	NM_003878	2.39
GJC1	NM_005497	2.56
GMPPA	NM_013335	2.12
GNPNAT1	NM_198066	2.33
GOLT1A	NM_198447	2.34
GPR39	NM_001508	2.30
GPR89B	NM_016334	2.60
GRHL3	NM_198173	3.40
GSPT1	NM_001130007///NM_002094	3.42
GTSE1	AY927424///NM_016426	2.36
GUCY1B2	NR_003923	5.59
H2AFV	NM_012412///NM_138635	4.13
H2AFZ	NM_002106	2.57
H3F3A	NM_002107	2.46
HDHD3	NM_031219	2.55
HHIPL2	NM_024746	7.31
HINT3	NM_138571	2.57
HIST1H1C	NM_005319	4.37
HIST1H1E	NM_005321	3.22
HIST1H2AA	NM_170745	2.85
HIST1H2AB	NM_003513	3.29
HIST1H2AC	BU680450///NM_003512	2.53
HIST1H2AD	NM_021065	4.71
HIST1H2AE	NM_021052	3.38
HIST1H2BC	NM_003526	5.11
HIST1H2BF	NM_003522	3.50
HIST1H2BG	NM_003518	5.37
HIST1H2BH	NM_003524	3.74
HIST1H2BI	NM_003525	3.28
HIST1H2BJ	NM_021058///BC014312	2.79
HIST1H2BL	NM_003519	3.41
HIST1H2BM	NM_003521	3.32
HIST1H2BN	NM_003520	4.41
HIST1H2BO	NM_003527	3.24

HIST1H3H	NM_003536	6.06
HIST1H4F	NM_003540	5.60
HIST1H4H	NM_003543	6.33
HIST1H4I	NM_003495	5.47
HIST1H4J	NM_021968	4.38
HIST1H4K	NM_003541	3.24
HIST2H2AA4	NM_001040874	5.05
HIST2H2AB	NM_175065	3.50
HIST2H2AC	NM_003517	3.94
HIST2H2BE	NM_003528	2.70
HIST2H4B	NM_001034077	5.16
HIST3H2A	NM_033445	4.08
HK2	NM_000189	3.69
HMGB3	NM_005342	2.66
HMGB3L1	NR_002165	7.99
HN1	NM_001002033	4.36
HNRNPA2B1	NM_002137	2.12
HNRNPAB	NM_004499	4.45
HOTAIR	NR_003716	8.13
HOXC10	NM_017409	4.42
HPSE	NM_006665///AF155510	2.58
HSPA6	NM_002155	2.83
HTATIP2	NM_006410///NM_001098523	2.68
HTRA4	NM_153692	5.70
HUS1	NM_004507	2.41
IBSP	NM_004967	9.78
IDH2	NM_002168	2.37
IFI30	NM_006332	3.55
IL1F9	NM_019618	2.56
IL29	NM_172140	2.07
INTS7	NM_015434	2.66
INTS8	NM_017864	2.73
ISG15	NM_005101	3.60
ITGB1BP2	NM_012278	2.14
IYD	NM_203395	4.84
JMJD6	NM_015167	2.17
KCNMB3	NM_171828	3.26
KIF15	NM_020242	4.61
KIF20A	NM_005733	5.18
KIF20B	NM_016195	4.29
KIF24	AK001795	3.47
KIF2C	NM_006845	3.60
KIF4A	NM_012310	3.10

KIFC1	NM_002263	2.95
KMO	NM_003679	2.84
KPNA2	NM_002266	3.08
KREMEN2	NM_172229	4.38
LACTB2	NM_016027	2.48
LAPTM4B	NM_018407	2.11
LASS2	NM_181746	2.28
LMNB1	NM_005573	3.09
LMX1B	NM_002316	2.52
LNX2	NM_153371	2.65
LOC100129478	XM_001719774	3.19
LOC100129566	XM_001718519	2.71
LOC100130938	XM_001720237	2.08
LOC100130967	XM_001716105	10.54
LOC100133153	XM_002343855	5.54
LOC100287615	XM_002343285	3.32
LOC100287852	XM_002342390	2.99
LOC100287902	XM_002342189	3.77
LOC100288842	XM_002342951	2.68
LOC100292646	XM_002345443	3.11
LOC149501	XR_018597	2.75
LOC158381	NR_003582	2.41
LOC283711	XR_040656	9.14
LOC284441	NR_003128	2.05
LOC284837	NR_026961	2.23
LOC285216	AK092228	6.40
LOC340888	XR_018726	5.12
LOC389842	XM_372200	2.51
LOC399881	XR_040376	3.06
LOC401127	NR_026854	3.30
LOC441016	XM_001714867	2.23
LOC441294	NM_001008747	3.43
LOC441722	XM_497450	2.14
LOC441795	XM_001715230	4.92
LOC728218	BC065757	2.19
LOC729595	XM_001130734	5.31
LOC730167	XM_001726158	3.10
LPAR2	NM_004720	2.78
LRRC59	NM_018509	2.87
LYPLA2	NM_007260	2.50
MAD2L1	NM_002358	5.31
MAL2	NM_052886	3.96
MARS2	NM_138395	2.32

MASTL	NM_032844	2.98
MBD5	BC014534	2.17
MCART2	NM_001034172	3.05
MCART3P	NR_026540	2.64
MCM4	NM_005914	2.76
METTL2A	NM_181725	3.01
MGAT4A	NM_012214	2.92
MINPP1	NM_004897	2.64
MNX1	NM_005515	4.13
MOCS3	NM_014484	2.37
MPHOSPH9	NM_022782	2.42
MRPL13	NM_014078	2.66
MTBP	NM_022045///AK022122	2.43
MTCP1	NM_001018025	2.82
MTERFD1	NM_015942	2.22
MTFR1	NM_014637	4.57
NAGA	NM_000262	2.60
NAT2	NM_000015	4.21
NCAPG2	NM_017760	3.27
NDUFA4L2	NM_020142	2.90
NEIL3	NM_018248	5.40
NEK2	NM_002497	9.18
NKX3-2	NM_001189	2.82
NLRP12	NM_033297	2.43
NOM1	NM_138400	3.35
NOX4	NM_016931	7.03
NOXO1	NM_144603	2.47
NP	NM_000270	2.21
NQO1	NM_000903	3.34
NR2F6	NM_005234	2.94
NUDT5	NM_014142	2.66
NUF2	NM_145697	9.80
OAS1	NM_002534	3.95
OAS2	NM_016817	4.74
OASL	NM_003733	4.53
OAZ3	NM_016178	4.12
OR2B6	NM_012367	2.35
OR51E1	NM_152430	2.99
P2RY6	NM_176798	2.76
P4HA1	NM_000917	3.61
PANX1	NM_015368	2.34
PARP12	NM_022750	2.94
PARP9	NM_031458	2.52

PDIA3	NM_005313	2.67
PDK3	NM_005391	2.45
PEX13	NM_002618	3.57
PGM3	NM_015599	2.22
PHEX	NM_000444	5.92
PIGM	NM_145167	2.18
PL-5283	NM_001130929	2.59
PLAC1	NM_021796	2.58
PLAUR	NM_001005377	2.77
PLEKHA8	NM_032639	3.01
PLK4	NM_014264	4.51
POLB	NM_002690	2.14
POP1	NM_001145860///NM_015029	2.39
PPIH	NM_006347	2.33
PPIL5	NM_203467///NM_152329	3.06
PPP1R15B	NM_032833	2.58
PPP4C	NM_002720	2.93
PRC1	NM_003981	2.79
PRKDC	NM_006904	2.14
PRPF3	NM_004698	2.34
PRPS1L1	NM_175886	3.21
PRRG4	NM_024081	2.40
PRUNE	NM_021222	2.51
PSENEN	NM_172341	2.54
PTTG1	NM_004219	2.65
PTTG2	NM_006607	3.51
PTTG3P	NR_002734	4.53
PUS10	NM_144709	2.70
RAB20	NM_017817	3.93
RACGAP1	NM_013277	3.09
RACGAP1P	NR_026583	3.44
RAD1	NM_002853	2.39
RAD54B	NM_012415	3.07
RAD54L	NM_003579	2.61
RAG1AP1	NM_018845	3.78
RAP2C	NM_021183	2.35
RASSF7	NM_003475	3.04
RBL1	NM_002895///NM_183404	3.52
RCE1	NM_005133	2.15
RDH10	NM_172037	3.72
RECQL4	NM_004260	2.28
REG1A	NM_002909	2.47
RP11-49G10.8	NR_026760	2.44

RPE	NM_006916	2.73
RPL39L	NM_052969	2.51
RTKN2	NM_145307	5.32
RUVBL1	NM_003707	2.54
S100A9	NM_002965	3.31
S100P	NM_005980	9.28
SDHC	NM_003001	3.76
SEC22B	NM_004892	2.19
SEC61G	NM_014302	2.70
SERPINE1	BU618641///NM_000602	4.11
SF3B4	NM_005850	2.63
SFXN1	NM_022754	2.52
SGOL1	NM_001012410///NM_001012409	3.41
SGOL2	NM_152524	4.78
SGPL1	NM_003901	2.72
SHCBP1	NM_024745	5.47
SIAH2	NM_005067	2.97
SIGLEC12	NM_053003	2.47
SIGLEC7	NM_014385	2.43
SIX2	NM_016932	2.89
SKA1	NM_001039535	4.98
SKIL	NM_005414	4.37
SLC16A6	NM_004694	2.18
SLC19A1	NM_194255	2.28
SLC25A16	NM_152707	2.96
SLC30A6	NM_017964	2.73
SLC35A2	NM_001042498///NM_005660	3.11
SLC35B3	NM_015948	3.08
SLC39A11	NM_139177	2.66
SLC4A2	NM_003040	2.12
SLC7A6	NM_001076785	2.61
SNRPA1	NM_003090	2.07
SOCS7	NM_014598	2.80
SORD	NM_003104	2.56
SPAG1	NM_003114	2.67
SPAG5	NM_006461	3.42
SPATA17	NM_138796	2.10
SPINK1	NM_003122	8.47
SPOCD1	NM_144569///AK097227	3.31
SRD5A2	NM_000348	4.68
SRD5A3	NM_024592	2.41
STAM2	NM_005843	2.14
STAT1	NM_007315///NM_139266	2.68

STIL	NM_001048166	4.17
STK38L	NM_015000	2.72
STX3	NM_004177	2.56
SULT2B1	NM_004605	3.33
SUV39H2	NM_024670	2.26
SYAP1	NM_032796	2.38
SYNGR2	NM_004710///BC105992	2.63
TARS	NM_152295	2.22
TEP1	NM_007110	2.54
TEX19	NM_207459	3.23
TFF2	NM_005423	2.92
THOC4	NM_005782	2.30
TMED7-TICAM2	NM_001164468	2.22
TMED9	NM_017510	3.22
TMEM49	NM_030938	3.24
TMEM92	NM_153229	3.10
TMPO	NM_001032283///NM_003276	4.53
TMPRSS4	NM_019894	4.55
TNIP2	NM_024309	3.00
TP53RK	NM_033550	2.39
TPX2	NM_012112	4.44
TRAPPC3	NM_014408	2.31
TREM1	NM_018643	6.75
TSPAN13	NM_014399	3.64
TSTA3	NM_003313	3.07
TTC26	NM_024926	2.85
TTC39B	NM_152574	3.21
TXNRD1	NM_003330	2.79
TYMS	NM_001071	2.82
UBE2NL	NM_001012989	2.81
UBE2T	NM_014176	3.63
UGGT1	NM_020120	4.96
UHRF1	NM_013282	6.24
UNC5CL	NM_173561	2.61
URB2	NM_014777	2.91
USP12	NM_182488	2.30
USP18	NM_017414	2.89
USP41	XM_937988	3.54
WBSCR28	NM_182504	2.37
WDHD1	NM_007086	3.14
WDR5	NM_017588	2.94
WDR51A	NM_015426	2.98
WISP1	NM_080838///NM_003882	3.28

XKRX	NM_212559	3.72
XPOT	NM_007235	2.12
YTHDF3	NM_152758	3.16
ZBED4	NM_014838	2.59
ZBTB9	NM_152735	2.26
ZDHHC13	NM_019028	2.30
ZNF146	NM_007145	2.20
ZNF274	NM_133502	2.04
ZNF443	NM_005815	2.60
ZNF623	NM_014789	3.29
ZNF695	NM_020394	5.05
ZNF707	NM_173831	2.27
ZNF75A	NM_153028	2.36
ZNRF2	NM_147128	2.65
