# Supplementary material for: Integrated Genomic and Epigenomic Analysis of Breast Cancer Brain Metastasis
Source: PLoS One. 2014 Jan 29;9(1):e85448. doi: 10.1371/journal.pone.0085448 (PMC3906004; doi:10.1371/journal.pone.0085448)
Supplement: File S1 — Supporting figures and tables. Figure S1: Combined Network for Upstream Analysis of FOXM1 and TBX2. The downstream genes connected to FOXM1 and TBX2 were illustrated as a network in IPA. The mRNA expression ratios are listed below the gene nodes. The legend within figure describes the node and edge color keys. Figure S2: Word Cloud Analysis of Cluster Enrichments. We have used word clouds to visually summarize the textual results from the enrichment analysis of each gene cluster as observed in Figure 3. The results were generated using www.wordle.net web resource. The larger the word, the more times it is mentioned in the enrichment categories. Supplementary Tables in File S1. Table S1a. Table S1b. Table S2. Table S3a. Table S3b. Table S4a. Figure S1. Table S4b. Table S5a–b. Table S6a–b. Table S7. Table S8a–f. Table S9a–f. Figure S2. Table S10. Table S11a–c. Table S11d. Table S12. Table S13. Table S14. (ZIP) [file pone.0085448.s001.zip › Supplementary Table S3b.pdf]

Supplementary Figure 3b. Functional Annotation of Downregulated Genes using Ingenuity® Systems Core Analysis Workflow

Category	Functions Annotation	p-Value	Predicted Activation State	Activatio	# Molecules	Molecules
Tissue Morphology	quantity of cells	2.35E-06	Decreased	-3.702	121	CDKN2A,IL6,KL,STAT1
Tissue Morphology	quantity of blood cells	5.53E-03	Decreased	-3.165	67	CDKN2A,IL6,KL,STAT1
Hematological System Development and Function	quantity of blood cells	5.53E-03	Decreased	-3.165	67	AKT1,CDKN1C,DMD,FLNC,HEY1,ITGA7,PIK3R1
Tissue Morphology	quantity of central nervous system cells	1.70E-04	Decreased	-2.548	16	AKT1,CDKN1C,DMD,FLNC,HEY1,ITGA7,PIK3R1
Nervous System Development and Function	quantity of central nervous system cells	1.70E-04	Decreased	-2.548	16	ADRB2,AKT1,CDKN1C,DMD,FLNC,FN1,HEY1,IL6,ITGA7
Cellular Development	differentiation of cells	1.30E-04	Decreased	-2.542	124	ADRB2,AKT1,CDKN1C,DMD,FLNC,FN1,HEY1,IL6,ITGA7
Cancer	hyperplasia of tissue	6.78E-03	Decreased	-2.379	8	
Tissue Morphology	quantity of brain cells	2.84E-04	Decreased	-2.361	14	
Nervous System Development and Function	quantity of brain cells	2.84E-04	Decreased	-2.361	14	AKT1,CD9,EME1,FGF2,FN1,RAD54L,SOX10,UHRF1
Cancer	adenocarcinoma	1.22E-08	Decreased	-2.236	84	AKT1,CD9,EME1,FGF2,FN1,RAD54L,SOX10,UHRF1
Cell Cycle	cell division of blood cells	1.30E-03	Decreased	-2.000	6	F3,FGF2,FN1,FOLH1,KRT1,NR4A1,PLAUR

7,PIK3R1,RGS2,SLC2A4
7,PIK3R1,RGS2,SLC2A4

A4,EXO1,F3,FAM72D,FAM83D,FGF2,FLRT2,FN1,FOLH1,FOXM1,GCH1,GGH,GREB1,GREM2,HIST1H3A (includes others),HIST1H4A (includes others),HOXC10,HPSE,HSD17B11,HTATIP2,IBSP,IFNW1,IL29,IL6,ISG1[illegible]
A4,EXO1,F3,FAM72D,FAM83D,FGF2,FLRT2,FN1,FOLH1,FOXM1,GCH1,GGH,GREB1,GREM2,HIST1H3A (includes others),HIST1H4A (includes others),HOXC10,HPSE,HSD17B11,HTATIP2,IBSP,IFNW1,IL29,IL6,ISG1[illegible]

ACPP,ADAM9,ADRA2A,AKR1B10,AKT1,ALYREF,[illegible]
ACPP,ADAM9,ADRA2A,AKR1B10,AKT1,ALYREF,[illegible]

AURKA,AXIN2
AURKA,AXIN2
