# Supplementary material for: Integrated Genomic and Epigenomic Analysis of Breast Cancer Brain Metastasis
Source: PLoS One. 2014 Jan 29;9(1):e85448. doi: 10.1371/journal.pone.0085448 (PMC3906004; doi:10.1371/journal.pone.0085448)
Supplement: File S1 — Supporting figures and tables. Figure S1: Combined Network for Upstream Analysis of FOXM1 and TBX2. The downstream genes connected to FOXM1 and TBX2 were illustrated as a network in IPA. The mRNA expression ratios are listed below the gene nodes. The legend within figure describes the node and edge color keys. Figure S2: Word Cloud Analysis of Cluster Enrichments. We have used word clouds to visually summarize the textual results from the enrichment analysis of each gene cluster as observed in Figure 3. The results were generated using www.wordle.net web resource. The larger the word, the more times it is mentioned in the enrichment categories. Supplementary Tables in File S1. Table S1a. Table S1b. Table S2. Table S3a. Table S3b. Table S4a. Figure S1. Table S4b. Table S5a–b. Table S6a–b. Table S7. Table S8a–f. Table S9a–f. Figure S2. Table S10. Table S11a–c. Table S11d. Table S12. Table S13. Table S14. (ZIP) [file pone.0085448.s001.zip › Supplementary Table S5a.pdf]

Supplementary Table 5a. Summary report of GSE analysis using the c5 gene set library (GSEA|MSigDB) containing 109 gene sets for upregulated genes in BBM

ES: enrichment score; NES: normalized enrichment score; FDR: false discovery rate; FWER: familywise error rate

Column descriptions

GENE SET: Gene set name. For MSigDB gene sets, the description is the gene set page on the GSEA web site.
SIZE: Number of genes in the gene set after filtering out those genes not in the expression dataset
ES: Enrichment score for the gene set; that is, the degree to which this gene set is overrepresented at the top or bottom of the ranked list of genes in the expression dataset.
NES: Normalized enrichment score; that is, the enrichment score for the gene set after it has been normalized across analyzed gene sets.
NOM p-value: Nominal p value; that is, the statistical significance of the enrichment score. The nominal p value is not adjusted for gene set size or multiple hypothesis testing; therefore, it is of limited use in comparing gene sets.
FDR q-value: False discovery rate; that is, the estimated probability that the normalized enrichment score represents a false positive finding.
FWER p-value: Familywise-error rate; that is, a more conservatively estimated probability that the normalized enrichment score represents a false positive finding. Because the goal of GSEA is to generate hypotheses, the GSEA team recommends focusing on the FDR statistic.
RANK AT MAX: The position in the ranked list at which the maximum enrichment score occurred. The more interesting gene sets achieve the maximum

Gene Set Name	SIZE	ES	NES	NOM p-val	FDR q-value	FWER p-value	RANK AT MAX	LEADING EDGE
REGULATION_OF_DNA_METABOLIC_PROCESS	26	0.63	1.85	0.001972387	1.00	0.427	2304	tags=58%, list=18%, signal=70%
RNA_PROCESSING	136	0.52	1.85	0.002040816	0.57	0.43	1647	tags=38%, list=13%, signal=43%
MICROTUBULE	21	0.60	1.85	0	0.39	0.436	2506	tags=43%, list=20%, signal=53%
RNA_SPLICING	75	0.56	1.82	0.00409836	0.41	0.523	1641	tags=44%, list=13%, signal=50%
ER_NUCLEAR_SIGNALING_PATHWAY	13	0.71	1.77	0	0.59	0.699	2430	tags=62%, list=19%, signal=76%
SECRETION_BY_CELL	72	0.49	1.77	0.001976285	0.50	0.706	2613	tags=42%, list=20%, signal=52%
ACETYLGLUCOSAMINYLTRANSFERASE_ACTIVITY	11	0.69	1.76	0.002053388	0.47	0.735	1467	tags=45%, list=11%, signal=51%
SPINDLE	22	0.64	1.76	0.011811024	0.42	0.738	2452	tags=55%, list=19%, signal=67%
CELL_CYCLE_PROCESS	128	0.49	1.75	0.004056795	0.40	0.762	2452	tags=41%, list=19%, signal=51%
CELL_CYCLE_PHASE	112	0.48	1.75	0.01	0.37	0.769	2419	tags=41%, list=19%, signal=50%
SECRETORY_PATHWAY	53	0.53	1.74	0.007952286	0.34	0.774	2308	tags=45%, list=18%, signal=55%
SPLICEOSOME	43	0.53	1.73	0.003944773	0.38	0.82	2030	tags=47%, list=16%, signal=55%
MRNA_METABOLIC_PROCESS	74	0.52	1.73	0.013972056	0.35	0.82	2674	tags=50%, list=21%, signal=63%
MRNA_PROCESSING_GO_0006397	64	0.55	1.72	0.01622718	0.33	0.826	2674	tags=52%, list=21%, signal=65%
REGULATION_OF_MITOSIS	21	0.61	1.72	0.010183299	0.31	0.827	3002	tags=57%, list=24%, signal=75%
NUCLEAR_ENVELOPE_ENDOPLASMIC_RETICULUM_NETWORK	66	0.52	1.72	0.00996016	0.30	0.83	2851	tags=50%, list=22%, signal=64%
GOLGI_VESICLE_TRANSPORT	40	0.56	1.72	0.013944224	0.28	0.831	2308	tags=50%, list=18%, signal=61%
M_PHASE	76	0.53	1.72	0.006224067	0.27	0.832	2547	tags=47%, list=20%, signal=59%
M_PHASE_OF_MITOTIC_CELL_CYCLE	57	0.53	1.72	0.008097166	0.26	0.836	2547	tags=49%, list=20%, signal=61%
RESPONSE_TO_DNA_DAMAGE_STIMULUS	124	0.47	1.71	0.006147541	0.26	0.843	3559	tags=52%, list=28%, signal=71%
MOTOR_ACTIVITY	17	0.59	1.71	0.003976143	0.25	0.844	2521	tags=53%, list=20%, signal=66%
PEPTIDYL_AMINO_ACID_MODIFICATION	49	0.45	1.71	0.003891051	0.25	0.857	2318	tags=37%, list=18%, signal=45%
DI___TRI_VALENT_INORGANIC_CATION_TRANSPORT	14	0.62	1.70	0.00952381	0.26	0.871	1313	tags=36%, list=10%, signal=40%
DNA_REPAIR	101	0.48	1.70	0.008163265	0.25	0.873	3819	tags=54%, list=30%, signal=77%
ENDOMEMBRANE_SYSTEM	150	0.45	1.70	0.010141988	0.25	0.882	2884	tags=42%, list=23%, signal=54%
UNFOLDED_PROTEIN_BINDING	30	0.56	1.69	0.01968504	0.25	0.887	3592	tags=57%, list=28%, signal=79%
MITOSIS	55	0.53	1.69	0.016460905	0.24	0.887	2547	tags=49%, list=20%, signal=61%
TRANSFERASE_ACTIVITY__TRANSFERRING_HEXOSYL_GROUPS	48	0.48	1.69	0.014056225	0.23	0.889	1681	tags=33%, list=13%, signal=38%
ENDOPLASMIC_RETICULUM_MEMBRANE	59	0.53	1.69	0.015968064	0.23	0.889	2851	tags=51%, list=22%, signal=65%
MEMBRANE_COAT	14	0.70	1.69	0.018292682	0.22	0.893	1817	tags=57%, list=14%, signal=67%
COATED_MEMBRANE	14	0.70	1.69	0.018292682	0.21	0.893	1817	tags=57%, list=14%, signal=67%
G1_S_TRANSITION_OF_MITOTIC_CELL_CYCLE	18	0.61	1.69	0.007858546	0.21	0.893	3391	tags=67%, list=27%, signal=91%
ENDOPLASMIC_RETICULUM_PART	64	0.52	1.69	0.013944224	0.20	0.893	3132	tags=53%, list=25%, signal=70%
CHROMATIN	25	0.57	1.68	0.015810277	0.21	0.908	3200	tags=52%, list=25%, signal=69%
ENDONUCLEASE_ACTIVITY	22	0.58	1.67	0.007766991	0.22	0.917	2600	tags=55%, list=20%, signal=68%
CYTOPLASMIC_VESICLE_MEMBRANE	17	0.66	1.67	0.016632017	0.23	0.932	4289	tags=88%, list=34%, signal=133%
CYTOPLASMIC_VESICLE_PART	17	0.66	1.67	0.016632017	0.22	0.932	4289	tags=88%, list=34%, signal=133%
INTRA_GOLGI_VESICLE_MEDIATED_TRANSPORT	10	0.67	1.66	0.016842104	0.22	0.933	1286	tags=50%, list=10%, signal=56%
MITOTIC_CELL_CYCLE	99	0.46	1.66	0.01629328	0.21	0.935	2419	tags=40%, list=19%, signal=49%
RIBONUCLEOPROTEIN_COMPLEX	112	0.48	1.66	0.017681729	0.22	0.941	2600	tags=44%, list=20%, signal=54%
TRNA_METABOLIC_PROCESS	14	0.64	1.66	0.007936508	0.21	0.942	1569	tags=43%, list=12%, signal=49%
RESPONSE_TO_ENDOGENOUS_STIMULUS	148	0.41	1.66	0.002040816	0.21	0.944	3276	tags=43%, list=26%, signal=57%
CELL_CYCLE_CHECKPOINT_GO_0000075	28	0.51	1.65	0.014285714	0.21	0.947	2577	tags=46%, list=20%, signal=58%
NUCLEOLAR_PART	14	0.68	1.65	0.02385686	0.21	0.95	2600	tags=57%, list=20%, signal=72%
MITOTIC_CELL_CYCLE_CHECKPOINT	11	0.64	1.65	0.010438413	0.21	0.953	2547	tags=64%, list=20%, signal=79%
VESICLE_COAT	13	0.70	1.64	0.020283977	0.22	0.961	1817	tags=62%, list=14%, signal=72%
COATED_VESICLE_MEMBRANE	13	0.70	1.64	0.020283977	0.21	0.961	1817	tags=62%, list=14%, signal=72%
TRANSFERASE_ACTIVITY__TRANSFERRING_ACYL_GROUPS	42	0.46	1.64	0.010706638	0.22	0.964	2763	tags=38%, list=22%, signal=48%
RNA_SPLICING__VIA_TRANSESTERIFICATION_REACTIONS	31	0.52	1.63	0.034	0.22	0.966	1464	tags=42%, list=11%, signal=47%
GTP_BINDING	27	0.51	1.63	0.02414487	0.22	0.967	1317	tags=33%, list=10%, signal=37%
GUANYL_NUCLEOTIDE_BINDING	27	0.51	1.63	0.02414487	0.22	0.967	1317	tags=33%, list=10%, signal=37%
BIOPOLYMER_CATABOLIC_PROCESS	90	0.42	1.62	0.014344262	0.23	0.974	2269	tags=37%, list=18%, signal=44%
INTRINSIC_TO_ORGANELLE_MEMBRANE	37	0.51	1.62	0.034412954	0.23	0.976	1194	tags=30%, list=9%, signal=33%
NUCLEOTIDYLTRANSFERASE_ACTIVITY	36	0.50	1.62	0.01992032	0.23	0.977	2680	tags=44%, list=21%, signal=56%
ENDORIBONUCLEASE_ACTIVITY	13	0.64	1.61	0.02745098	0.23	0.977	2600	tags=69%, list=20%, signal=87%
CHROMOSOMAL_PART	67	0.47	1.61	0.028	0.23	0.977	3001	tags=45%, list=24%, signal=58%
PROTEASOME_COMPLEX	21	0.70	1.61	0.034615386	0.23	0.977	3245	tags=81%, list=25%, signal=108%
ENDONUCLEASE_ACTIVITY_GO_0016893	11	0.71	1.61	0.022357723	0.23	0.979	2600	tags=82%, list=20%, signal=103%
CYTOSKELETAL_PART	141	0.37	1.61	0.005825243	0.22	0.979	2564	tags=33%, list=20%, signal=40%
INTEGRAL_TO_ORGANELLE_MEMBRANE	36	0.52	1.61	0.034764826	0.22	0.98	1194	tags=31%, list=9%, signal=34%
DNA_RECOMBINATION	35	0.50	1.61	0.021442495	0.22	0.98	2392	tags=37%, list=19%, signal=46%
CHROMOSOME	84	0.46	1.61	0.028	0.22	0.98	3001	tags=45%, list=24%, signal=59%
PROTEOLYSIS	125	0.40	1.60	0.01010101	0.22	0.981	2652	tags=38%, list=21%, signal=47%
NUCLEASE_ACTIVITY	41	0.49	1.60	0.028397566	0.22	0.982	2715	tags=41%, list=21%, signal=52%
SECRETION	99	0.39	1.60	0.004032258	0.22	0.982	2613	tags=34%, list=20%, signal=43%
NUCLEAR_BODY	25	0.53	1.59	0.040169135	0.23	0.983	1703	tags=40%, list=13%, signal=46%
VESICLE_MEMBRANE	19	0.58	1.59	0.03285421	0.23	0.985	4289	tags=79%, list=34%, signal=119%
ORGANELLE_LOCALIZATION	15	0.58	1.59	0.019723866	0.23	0.985	3260	tags=60%, list=26%, signal=80%
ATPASE_ACTIVITY	82	0.42	1.58	0.015873017	0.23	0.986	3245	tags=43%, list=25%, signal=57%
NUCLEOLUS	93	0.46	1.58	0.043209877	0.23	0.986	3251	tags=45%, list=25%, signal=60%
CERAMIDE_METABOLIC_PROCESS	10	0.64	1.58	0.016260162	0.23	0.986	3132	tags=50%, list=25%, signal=66%
MACROMOLECULE_CATABOLIC_PROCESS	102	0.41	1.58	0.014112903	0.23	0.986	3233	tags=44%, list=25%, signal=59%
KINETOCHORE	16	0.57	1.58	0.041257367	0.22	0.986	2229	tags=44%, list=17%, signal=53%
SPHINGOID_METABOLIC_PROCESS	11	0.62	1.58	0.023952097	0.22	0.986	3132	tags=45%, list=25%, signal=60%
ESTABLISHMENT_OF_PROTEIN_LOCALIZATION	132	0.36	1.58	0.012195122	0.23	0.987	2613	tags=33%, list=20%, signal=41%
GOLGI_ASSOCIATED_VESICLE	23	0.56	1.57	0.04411765	0.23	0.987	1817	tags=43%, list=14%, signal=51%
CHROMOSOME__PERICENTRIC_REGION	18	0.58	1.57	0.039337475	0.22	0.987	4273	tags=72%, list=33%, signal=108%
CELLULAR_MACROMOLECULE_CATABOLIC_PROCESS	80	0.43	1.57	0.022222223	0.22	0.988	3233	tags=49%, list=25%, signal=65%

RIBONUCLEOPROTEIN_COMPLEX_BIOGENESIS_AND_ASSEMBLY	62	0.42	1.57	0.027083334	0.23	0.99	1621	tags=29%, list=13%, signal=33%
CELLULAR_PROTEIN_CATABOLIC_PROCESS	47	0.46	1.57	0.03929273	0.23	0.99	2428	tags=45%, list=19%, signal=55%
PROTEIN_TRANSPORT	114	0.37	1.56	0.01871102	0.23	0.99	2417	tags=33%, list=19%, signal=41%
SPINDLE_MICROTUBULE	10	0.64	1.56	0.055343512	0.23	0.99	2452	tags=50%, list=19%, signal=62%
S_ADENOSYLMETHIONINE_DEPENDENT_METHYLTRANSFERASE	17	0.56	1.56	0.05263158	0.23	0.99	3247	tags=65%, list=25%, signal=87%
INTRACELLULAR_PROTEIN_TRANSPORT	107	0.38	1.56	0.018255578	0.23	0.99	2417	tags=34%, list=19%, signal=41%
DNA_POLYMERASE_ACTIVITY	13	0.61	1.56	0.059615385	0.23	0.99	3179	tags=62%, list=25%, signal=82%
SMALL_NUCLEAR_RIBONUCLEOPROTEIN_COMPLEX	18	0.59	1.55	0.058708414	0.24	0.99	2030	tags=56%, list=16%, signal=66%
TRANSFERASE_ACTIVITY__TRANSFERRING_GROUPS_OTHER_TH	37	0.45	1.55	0.02258727	0.24	0.99	3705	tags=46%, list=29%, signal=65%
GLYCOPROTEIN_METABOLIC_PROCESS	59	0.46	1.55	0.026639344	0.24	0.991	4273	tags=59%, list=33%, signal=89%
INTRINSIC_TO_ENDOPLASMIC_RETICULUM_MEMBRANE	18	0.61	1.55	0.04024145	0.24	0.992	2824	tags=61%, list=22%, signal=78%
INTEGRAL_TO_ENDOPLASMIC_RETICULUM_MEMBRANE	18	0.61	1.55	0.04024145	0.24	0.992	2824	tags=61%, list=22%, signal=78%
RNA_HELICASE_ACTIVITY	21	0.53	1.55	0.04032258	0.23	0.993	1639	tags=38%, list=13%, signal=44%
DNA_DAMAGE_RESPONSE__SIGNAL_TRANSDUCTION	23	0.53	1.54	0.045454547	0.24	0.994	2577	tags=52%, list=20%, signal=65%
PROTEIN_CATABOLIC_PROCESS	55	0.44	1.54	0.035785288	0.24	0.994	2428	tags=42%, list=19%, signal=51%
UBIQUITIN_PROTEIN_LIGASE_ACTIVITY	35	0.49	1.54	0.050200805	0.24	0.995	2269	tags=43%, list=18%, signal=52%
RIBOSOME_BIOGENESIS_AND_ASSEMBLY	10	0.69	1.54	0.044444446	0.24	0.996	918	tags=40%, list=7%, signal=43%
SMALL_PROTEIN_CONJUGATING_ENZYME_ACTIVITY	37	0.49	1.53	0.05	0.24	0.996	2269	tags=43%, list=18%, signal=52%
PYROPHOSPHATASE_ACTIVITY	150	0.37	1.52	0.014256619	0.25	0.997	3256	tags=37%, list=26%, signal=50%
PROTEIN_FOLDING	39	0.43	1.52	0.044088177	0.25	0.997	2672	tags=36%, list=21%, signal=45%
METHYLTRANSFERASE_ACTIVITY	25	0.48	1.52	0.049115915	0.25	0.997	2782	tags=48%, list=22%, signal=61%
TRANSFERASE_ACTIVITY__TRANSFERRING_ONE_CARBON_GRO	25	0.48	1.52	0.049115915	0.25	0.997	2782	tags=48%, list=22%, signal=61%
NEGATIVE_REGULATION_OF_TRANSLATION	12	0.57	1.52	0.06395349	0.25	0.997	1980	tags=42%, list=16%, signal=49%
PROTEIN_LOCALIZATION	146	0.34	1.51	0.018218623	0.26	0.997	2613	tags=31%, list=20%, signal=38%
GLYCOPROTEIN_BIOSYNTHETIC_PROCESS	50	0.43	1.51	0.037623763	0.26	0.997	3728	tags=50%, list=29%, signal=70%
NUCLEOSIDE_TRIPHOSPHATASE_ACTIVITY	143	0.37	1.51	0.02	0.26	0.997	3256	tags=38%, list=26%, signal=50%
SMALL_CONJUGATING_PROTEIN_LIGASE_ACTIVITY	37	0.48	1.51	0.065868266	0.26	0.998	2522	tags=46%, list=20%, signal=57%
NUCLEOBASE__NUCLEOSIDE__NUCLEOTIDE_KINASE_ACTIVITY	12	0.62	1.50	0.06029106	0.27	0.998	3105	tags=75%, list=24%, signal=99%
NUCLEOBASE__NUCLEOSIDE_AND_NUCLEOTIDE_METABOLIC_I	32	0.46	1.50	0.042168673	0.27	0.998	4560	tags=53%, list=36%, signal=82%
PROTEIN_AMINO_ACID_O_LINKED_GLYCOSYLATION	13	0.53	1.50	0.054393306	0.27	0.998	2602	tags=54%, list=20%, signal=68%
ER_TO_GOLGI_VESICLE_MEDIATED_TRANSPORT	16	0.59	1.50	0.07098121	0.27	0.998	2308	tags=63%, list=18%, signal=76%
DEOXYRIBONUCLEASE_ACTIVITY	19	0.50	1.49	0.04296875	0.27	0.998	3200	tags=47%, list=25%, signal=63%
MICROTUBULE_CYTOSKELETON	100	0.37	1.49	0.04511278	0.28	0.998	2564	tags=29%, list=20%, signal=36%
DNA_INTEGRITY_CHECKPOINT	14	0.57	1.49	0.067226894	0.28	0.998	1757	tags=50%, list=14%, signal=58%
CYSTEINE_TYPE_PEPTIDASE_ACTIVITY	38	0.41	1.49	0.04225352	0.27	0.998	2962	tags=39%, list=23%, signal=51%
ENDODEOXYRIBONUCLEASE_ACTIVITY	10	0.61	1.49	0.05882353	0.27	0.998	2059	tags=50%, list=16%, signal=60%
REGULATION_OF_GENE_EXPRESSION__EPIGENETIC	25	0.47	1.49	0.049281314	0.28	0.998	1810	tags=32%, list=14%, signal=37%
DOUBLE_STRAND_BREAK_REPAIR	16	0.59	1.48	0.06225681	0.28	0.998	2392	tags=56%, list=19%, signal=69%
ONE_CARBON_COMPOUND_METABOLIC_PROCESS	21	0.48	1.48	0.05338809	0.28	0.998	2102	tags=38%, list=16%, signal=46%
SIGNAL_SEQUENCE_BINDING	12	0.59	1.48	0.06560636	0.28	0.998	286	tags=25%, list=2%, signal=26%
NEGATIVE_REGULATION_OF_CELLULAR_COMPONENT_ORGAN	14	0.51	1.48	0.0546875	0.28	0.998	2393	tags=43%, list=19%, signal=53%
CARBOHYDRATE_TRANSMEMBRANE_TRANSPORTER_ACTIVITY	10	0.57	1.48	0.06827309	0.28	0.998	518	tags=30%, list=4%, signal=31%
NEGATIVE_REGULATION_OF_CELLULAR_PROTEIN_METABOLIC_	22	0.47	1.47	0.04255319	0.28	0.998	2288	tags=41%, list=18%, signal=50%
NEGATIVE_REGULATION_OF_APOPTOSIS	92	0.37	1.47	0.030487806	0.28	0.999	2251	tags=32%, list=18%, signal=38%
NEGATIVE_REGULATION_OF_PROGRAMMED_CELL_DEATH	92	0.37	1.47	0.030487806	0.28	0.999	2251	tags=32%, list=18%, signal=38%
TRANSFERASE_ACTIVITY__TRANSFERRING_GLYCOSYL_GROUPS	65	0.38	1.47	0.030927835	0.28	0.999	2318	tags=32%, list=18%, signal=39%
EXOCYTOSIS	11	0.60	1.47	0.07755102	0.27	0.999	1520	tags=36%, list=12%, signal=41%
CELLULAR_CATABOLIC_PROCESS	138	0.33	1.46	0.022044089	0.29	0.999	2269	tags=28%, list=18%, signal=33%
PRIMARY_ACTIVE_TRANSMEMBRANE_TRANSPORTER_ACTIVITY	24	0.47	1.46	0.08366534	0.29	0.999	2918	tags=46%, list=23%, signal=59%
RIBONUCLEASE_ACTIVITY	21	0.53	1.46	0.1030303	0.30	0.999	2600	tags=48%, list=20%, signal=60%
HYDROLASE_ACTIVITY__ACTING_ON_CARBON_NITROGEN__BU	10	0.55	1.45	0.054474708	0.30	0.999	2894	tags=40%, list=23%, signal=52%
SPLICEOSOME_ASSEMBLY	17	0.50	1.45	0.06896552	0.31	1	1464	tags=47%, list=11%, signal=53%
CHROMATIN_BINDING	24	0.48	1.45	0.076771654	0.30	1	3214	tags=50%, list=25%, signal=67%
ATPASE_ACTIVITY__COUPLED	67	0.40	1.45	0.05940594	0.30	1	3214	tags=40%, list=25%, signal=54%
APOPTOTIC_PROGRAM	38	0.41	1.45	0.04483431	0.30	1	2236	tags=32%, list=18%, signal=38%
ACID_AMINO_ACID_LIGASE_ACTIVITY	41	0.46	1.45	0.085487075	0.30	1	4253	tags=63%, list=33%, signal=95%
DNA_PACKAGING	26	0.46	1.44	0.06313646	0.31	1	3802	tags=50%, list=30%, signal=71%
PROTEIN_SERINE_THREONINE_KINASE_ACTIVITY	130	0.33	1.44	0.023904383	0.30	1	2294	tags=25%, list=18%, signal=31%
DNA_DAMAGE_CHECKPOINT	13	0.54	1.44	0.08595388	0.31	1	1757	tags=46%, list=14%, signal=53%
LIGASE_ACTIVITY__FORMING_CARBON_NITROGEN_BONDS	49	0.43	1.44	0.06275304	0.31	1	3581	tags=51%, list=28%, signal=71%
GOLGI_MEMBRANE	29	0.51	1.44	0.12655602	0.31	1	1521	tags=34%, list=12%, signal=39%
MICROSOME	24	0.48	1.44	0.085192695	0.31	1	1106	tags=29%, list=9%, signal=32%
NUCLEOTIDE_BIOSYNTHETIC_PROCESS	11	0.57	1.43	0.07450981	0.31	1	1428	tags=36%, list=11%, signal=41%
NITROGEN_COMPOUND_BIOSYNTHETIC_PROCESS	15	0.50	1.43	0.09016393	0.31	1	1368	tags=47%, list=11%, signal=52%
NEGATIVE_REGULATION_OF_CELLULAR_BIOSYNTHETIC_PROCE	15	0.51	1.43	0.06986028	0.31	1	1980	tags=33%, list=16%, signal=39%
NEGATIVE_REGULATION_OF_BIOSYNTHETIC_PROCESS	15	0.51	1.43	0.06986028	0.31	1	1980	tags=33%, list=16%, signal=39%
CONDENSED_CHROMOSOME	24	0.55	1.43	0.12676056	0.31	1	3735	tags=71%, list=29%, signal=100%
TRANSCRIPTION_ELONGATION_REGULATOR_ACTIVITY	10	0.56	1.43	0.08979592	0.31	1	3971	tags=60%, list=31%, signal=87%
DNA_DIRECTED_RNA_POLYMERASE_II__HOLOENZYME	64	0.35	1.43	0.03877551	0.31	1	2752	tags=30%, list=22%, signal=38%
INTEGRATOR_COMPLEX	13	0.54	1.42	0.07214429	0.31	1	2386	tags=38%, list=19%, signal=47%
CATABOLIC_PROCESS	148	0.32	1.42	0.030487806	0.31	1	2269	tags=27%, list=18%, signal=32%
EXONUCLEASE_ACTIVITY	13	0.56	1.42	0.09014675	0.31	1	4582	tags=69%, list=36%, signal=108%
ER_GOLGI_INTERMEDIATE_COMPARTMENT	13	0.57	1.42	0.09453782	0.31	1	3260	tags=69%, list=26%, signal=93%
ALCOHOL_METABOLIC_PROCESS	55	0.37	1.42	0.042990655	0.31	1	3233	tags=42%, list=25%, signal=56%
NUCLEOTIDE_METABOLIC_PROCESS	27	0.45	1.42	0.06508876	0.31	1	3401	tags=41%, list=27%, signal=55%
NUCLEAR_EXPORT	26	0.47	1.42	0.09255533	0.31	1	3366	tags=54%, list=26%, signal=73%
HELICASE_ACTIVITY	43	0.41	1.42	0.08722109	0.31	1	1717	tags=28%, list=13%, signal=32%
CHROMOSOME_SEGREGATION	22	0.52	1.41	0.12753037	0.32	1	2229	tags=45%, list=17%, signal=55%
CHROMATIN_ASSEMBLY_OR_DISASSEMBLY	21	0.47	1.41	0.09657948	0.32	1	3414	tags=48%, list=27%, signal=65%
VESICLE_MEDIATED_TRANSPORT	126	0.38	1.41	0.10159363	0.32	1	2667	tags=35%, list=21%, signal=44%
GOLGI_APPARATUS_PART	78	0.40	1.41	0.11293635	0.32	1	4077	tags=50%, list=32%, signal=73%
LIPID_BIOSYNTHETIC_PROCESS	69	0.36	1.41	0.05490196	0.31	1	4195	tags=49%, list=33%, signal=73%
VESICULAR_FRACTION	26	0.45	1.41	0.08553971	0.31	1	1106	tags=27%, list=9%, signal=29%
UDP_GLYCOSYLTRANSFERASE_ACTIVITY	21	0.46	1.41	0.07450981	0.31	1	1467	tags=29%, list=11%, signal=32%
NUCLEOCYTOPLASMIC_TRANSPORT	65	0.38	1.41	0.06313646	0.31	1	3427	tags=45%, list=27%, signal=61%
DNA_DIRECTED_DNA_POLYMERASE_ACTIVITY	11	0.57	1.41	0.10852713	0.31	1	3179	tags=55%, list=25%, signal=73%
ATP_DEPENDENT_RNA_HELICASE_ACTIVITY	14	0.54	1.41	0.107421875	0.31	1	1472	tags=36%, list=12%, signal=40%
PROTEIN_TRANSPORTER_ACTIVITY	12	0.57	1.40	0.111801244	0.31	1	1989	tags=42%, list=16%, signal=49%
COATED_VESICLE	33	0.44	1.40	0.11320755	0.32	1	3039	tags=48%, list=24%, signal=63%
NUCLEAR_TRANSPORT	66	0.37	1.40	0.061601643	0.32	1	3427	tags=44%, list=27%, signal=60%
CELL_STRUCTURE_DISASSEMBLY_DURING_APOPTOSIS	10	0.56	1.40	0.08806262	0.32	1	1833	tags=40%, list=14%, signal=47%
HISTONE_DEACETYLASE_COMPLEX	13	0.47	1.39	0.08080808	0.32	1	2894	tags=46%, list=23%, signal=60%
CYSTEINE_TYPE_ENDOPEPTIDASE_ACTIVITY	28	0.41	1.39	0.06967213	0.32	1	2962	tags=39%, list=23%, signal=51%
NEGATIVE_REGULATION_OF_PROTEIN_METABOLIC_PROCESS	25	0.43	1.39	0.08239701	0.32	1	2288	tags=36%, list=18%, signal=44%
DI___TRI_VALENT_INORGANIC_CATION_TRANSMEMBRANE_TF	11	0.52	1.39	0.103515625	0.32	1	1313	tags=36%, list=10%, signal=40%

ATPASE_ACTIVITY__COUPLED_TO_MOVEMENT_OF_SUBSTANC	24	0.44	1.39	0.12723657	0.32	1	2918	tags=42%, list=23%, signal=54%
SPHINGOLIPID_METABOLIC_PROCESS	21	0.46	1.39	0.10557769	0.32	1	3595	tags=38%, list=28%, signal=53%
MEIOTIC_CELL_CYCLE	23	0.48	1.39	0.119521916	0.32	1	3949	tags=61%, list=31%, signal=88%
RESPONSE_TO_BIOTIC_STIMULUS	67	0.42	1.38	0.10663984	0.34	1	3572	tags=43%, list=28%, signal=60%
DNA_HELICASE_ACTIVITY	20	0.47	1.37	0.12955466	0.34	1	367	tags=20%, list=3%, signal=21%
PROTEIN_RNA_COMPLEX_ASSEMBLY	50	0.36	1.37	0.07770961	0.35	1	2674	tags=34%, list=21%, signal=43%
ELECTRON_CARRIER_ACTIVITY	57	0.37	1.37	0.12015504	0.35	1	3040	tags=37%, list=24%, signal=48%
TRANSLATION_INITIATION_FACTOR_ACTIVITY	17	0.53	1.37	0.13836478	0.35	1	4702	tags=65%, list=37%, signal=102%
REGULATION_OF_TRANSLATION	56	0.35	1.37	0.07854406	0.35	1	1980	tags=21%, list=16%, signal=25%
REGULATION_OF_CELL_CYCLE	104	0.31	1.36	0.051282052	0.35	1	2577	tags=29%, list=20%, signal=36%
CHROMOSOME_ORGANIZATION_AND_BIOGENESIS	88	0.36	1.36	0.09448819	0.35	1	3843	tags=48%, list=30%, signal=68%
CELL_CORTEX	21	0.44	1.36	0.10311284	0.35	1	1505	tags=33%, list=12%, signal=38%
CYTOKINESIS	10	0.57	1.36	0.1411043	0.35	1	1291	tags=30%, list=10%, signal=33%
HYDROLASE_ACTIVITY__ACTING_ON_ACID_ANHYDRIDES__CAT	23	0.44	1.36	0.14087301	0.35	1	2918	tags=43%, list=23%, signal=56%
REGULATION_OF_DNA_REPLICATION	10	0.58	1.36	0.14688128	0.35	1	2018	tags=50%, list=16%, signal=59%
REGULATION_OF_TRANSCRIPTION_FACTOR_ACTIVITY	30	0.40	1.35	0.093812376	0.36	1	4981	tags=67%, list=39%, signal=109%
PEPTIDASE_ACTIVITY	102	0.33	1.35	0.062378168	0.36	1	2962	tags=32%, list=23%, signal=42%
TRANSITION_METAL_ION_TRANSPORT	10	0.59	1.35	0.16898608	0.36	1	1313	tags=40%, list=10%, signal=45%
LIGASE_ACTIVITY	71	0.38	1.35	0.1252485	0.36	1	2522	tags=37%, list=20%, signal=45%
APOPTOTIC_NUCLEAR_CHANGES	11	0.50	1.34	0.121031746	0.36	1	958	tags=27%, list=8%, signal=29%
PROTEIN_TARGETING	79	0.33	1.34	0.084848486	0.36	1	2417	tags=29%, list=19%, signal=36%
REGULATION_OF_DNA_BINDING	36	0.39	1.34	0.0951417	0.36	1	4981	tags=64%, list=39%, signal=104%
PROTEIN_AUTOPROCESSING	20	0.43	1.34	0.13967611	0.37	1	2378	tags=35%, list=19%, signal=43%
PROTEIN_AMINO_ACID_AUTOPHOSPHORYLATION	20	0.43	1.34	0.13967611	0.37	1	2378	tags=35%, list=19%, signal=43%
TRANSCRIPTION_FACTOR_COMPLEX	73	0.34	1.33	0.099609375	0.38	1	2440	tags=26%, list=19%, signal=32%
PROTEIN_PROCESSING	32	0.37	1.33	0.11	0.38	1	2988	tags=34%, list=23%, signal=45%
RNA_DEPENDENT_ATPASE_ACTIVITY	15	0.50	1.33	0.1611002	0.38	1	1472	tags=33%, list=12%, signal=38%
NUCLEOBASE__NUCLEOSIDE__NUCLEOTIDE_AND_NUCLEIC_AC	24	0.46	1.32	0.15560167	0.39	1	2124	tags=42%, list=17%, signal=50%
RNA_CATABOLIC_PROCESS	18	0.47	1.32	0.17446809	0.39	1	2188	tags=44%, list=17%, signal=54%
CELL_DIVISION	11	0.55	1.32	0.1691023	0.39	1	1291	tags=27%, list=10%, signal=30%
DIGESTION	24	0.43	1.31	0.13872832	0.40	1	5090	tags=67%, list=40%, signal=111%
CELLULAR_RESPONSE_TO_STIMULUS	13	0.52	1.31	0.17729084	0.40	1	2484	tags=38%, list=19%, signal=48%
DNA_REPLICATION	66	0.38	1.31	0.1733871	0.41	1	3819	tags=47%, list=30%, signal=67%
MEIOSIS_I	13	0.51	1.30	0.18924303	0.41	1	2392	tags=46%, list=19%, signal=57%
I_KAPPAB_KINASE_NF_KAPPAB_CASCADE	76	0.38	1.30	0.17738791	0.42	1	4586	tags=50%, list=36%, signal=78%
BASE_EXCISION_REPAIR	13	0.51	1.30	0.1875	0.42	1	3819	tags=54%, list=30%, signal=77%
DNA_DEPENDENT_DNA_REPLICATION	34	0.42	1.30	0.18181819	0.42	1	2141	tags=35%, list=17%, signal=42%
N_ACYLTRANSFERASE_ACTIVITY	18	0.46	1.29	0.18145162	0.42	1	3705	tags=44%, list=29%, signal=63%
NEGATIVE_REGULATION_OF_BINDING	15	0.45	1.29	0.1626506	0.42	1	2865	tags=47%, list=22%, signal=60%
NEGATIVE_REGULATION_OF_CELL_ADHESION	12	0.47	1.29	0.15662651	0.42	1	1174	tags=25%, list=9%, signal=28%
POSITIVE_REGULATION_OF_BINDING	20	0.45	1.29	0.15461847	0.42	1	4977	tags=65%, list=39%, signal=106%
POSITIVE_REGULATION_OF_I_KAPPAB_KINASE_NF_KAPPAB_C/	57	0.40	1.29	0.20396039	0.42	1	4583	tags=54%, list=36%, signal=84%
ORGANELLAR_RIBOSOME	21	0.50	1.29	0.22352941	0.42	1	5411	tags=81%, list=42%, signal=140%
MITOCHONDRIAL_RIBOSOME	21	0.50	1.29	0.22352941	0.42	1	5411	tags=81%, list=42%, signal=140%
REGULATION_OF_CYCLIN_DEPENDENT_PROTEIN_KINASE_ACTI	29	0.37	1.29	0.12701613	0.42	1	1174	tags=24%, list=9%, signal=27%
CARBOHYDRATE_BIOSYNTHETIC_PROCESS	24	0.40	1.29	0.15132925	0.42	1	1613	tags=29%, list=13%, signal=33%
PROTEIN_DNA_COMPLEX_ASSEMBLY	40	0.38	1.28	0.15697674	0.42	1	3668	tags=45%, list=29%, signal=63%
NUCLEOTIDE_BINDING	137	0.30	1.28	0.1003937	0.42	1	3460	tags=35%, list=27%, signal=48%
RIBONUCLEOPROTEIN_BINDING	11	0.53	1.28	0.17227723	0.43	1	4616	tags=73%, list=36%, signal=114%
MEIOTIC_RECOMBINATION	10	0.52	1.27	0.18585859	0.44	1	2392	tags=50%, list=19%, signal=61%
3__5__EXONUCLEASE_ACTIVITY	10	0.51	1.27	0.19502075	0.44	1	4582	tags=60%, list=36%, signal=94%
ATP_DEPENDENT_HELICASE_ACTIVITY	21	0.46	1.27	0.19411765	0.44	1	1717	tags=29%, list=13%, signal=33%
PROTEIN_HETERODIMERIZATION_ACTIVITY	52	0.32	1.27	0.105675146	0.44	1	2430	tags=29%, list=19%, signal=35%
ESTABLISHMENT_AND_OR_MAINTENANCE_OF_CHROMATIN_A	55	0.34	1.26	0.14893617	0.45	1	3414	tags=42%, list=27%, signal=57%
ANTI_APOPTOSIS	70	0.33	1.26	0.14910537	0.45	1	2660	tags=31%, list=21%, signal=39%
ENDOPEPTIDASE_ACTIVITY	68	0.32	1.26	0.14092664	0.45	1	2962	tags=32%, list=23%, signal=42%
PROTEIN_AMINO_ACID_N_LINKED_GLYCOSYLATION	20	0.43	1.26	0.2045889	0.45	1	3175	tags=50%, list=25%, signal=66%
PHOSPHOLIPID_BIOSYNTHETIC_PROCESS	33	0.38	1.26	0.18962076	0.45	1	3902	tags=48%, list=31%, signal=70%
ORGANELLAR_SMALL_RIBOSOMAL_SUBUNIT	11	0.55	1.26	0.23762377	0.45	1	4423	tags=64%, list=35%, signal=97%
MITOCHONDRIAL_SMALL_RIBOSOMAL_SUBUNIT	11	0.55	1.26	0.23762377	0.45	1	4423	tags=64%, list=35%, signal=97%
SMALL_RIBOSOMAL_SUBUNIT	11	0.55	1.26	0.23762377	0.45	1	4423	tags=64%, list=35%, signal=97%
NUCLEAR_IMPORT	35	0.34	1.26	0.13398059	0.45	1	2165	tags=29%, list=17%, signal=34%
PROTEIN_METHYLTRANSFERASE_ACTIVITY	10	0.48	1.26	0.17684211	0.45	1	2782	tags=40%, list=22%, signal=51%
POSITIVE_REGULATION_OF_DNA_BINDING	19	0.43	1.25	0.202	0.45	1	4977	tags=63%, list=39%, signal=103%
PHOSPHOINOSITIDE_BIOSYNTHETIC_PROCESS	22	0.43	1.25	0.21807465	0.45	1	4473	tags=68%, list=35%, signal=105%
MICROTUBULE_BASED_PROCESS	50	0.34	1.25	0.16007905	0.45	1	2452	tags=32%, list=19%, signal=39%
CHROMATIN_MODIFICATION	37	0.37	1.25	0.18128654	0.46	1	2894	tags=38%, list=23%, signal=49%
NEGATIVE_REGULATION_OF_DEVELOPMENTAL_PROCESS	110	0.29	1.25	0.1184466	0.45	1	2460	tags=28%, list=19%, signal=35%
SEQUENCE_SPECIFIC_DNA_BINDING	35	0.37	1.25	0.16061185	0.45	1	3526	tags=40%, list=28%, signal=55%
REGULATION_OF_I_KAPPAB_KINASE_NF_KAPPAB_CASCADE	62	0.38	1.25	0.23874755	0.45	1	4311	tags=50%, list=34%, signal=75%
CELLULAR_CARBOHYDRATE_METABOLIC_PROCESS	76	0.31	1.25	0.15430862	0.45	1	3233	tags=38%, list=25%, signal=51%
CALCIUM_ION_TRANSPORT	10	0.48	1.25	0.20576923	0.45	1	1284	tags=20%, list=10%, signal=22%
OXIDOREDUCTASE_ACTIVITY__ACTING_ON_CH_OH_GROUP_O	44	0.34	1.25	0.18042226	0.45	1	3174	tags=39%, list=25%, signal=51%
TRANSLATION_REGULATOR_ACTIVITY	26	0.42	1.24	0.19198312	0.45	1	4929	tags=58%, list=39%, signal=94%
TRANSLATION_FACTOR_ACTIVITY__NUCLEIC_ACID_BINDING	26	0.42	1.24	0.19198312	0.45	1	4929	tags=58%, list=39%, signal=94%
MEMBRANE_LIPID_BIOSYNTHETIC_PROCESS	38	0.36	1.24	0.2058212	0.45	1	3902	tags=45%, list=31%, signal=64%
CYTOPLASMIC_VESICLE	72	0.32	1.24	0.18825911	0.45	1	3545	tags=46%, list=28%, signal=63%
CYTOPLASMIC_MEMBRANE_BOUND_VESICLE	72	0.32	1.24	0.18825911	0.45	1	3545	tags=46%, list=28%, signal=63%
RNA_SPLICING_FACTOR_ACTIVITY__TRANSESTERIFICATION_MI	15	0.42	1.24	0.19018404	0.45	1	2585	tags=40%, list=20%, signal=50%
PURINE_RIBONUCLEOTIDE_BINDING	124	0.29	1.24	0.13926499	0.45	1	3460	tags=34%, list=27%, signal=46%
MEMBRANE_LIPID_METABOLIC_PROCESS	75	0.31	1.23	0.16435644	0.46	1	3902	tags=37%, list=31%, signal=53%
REGULATION_OF_TRANSLATIONAL_INITIATION	21	0.38	1.23	0.19009902	0.46	1	1958	tags=24%, list=15%, signal=28%
CELLULAR_COMPONENT_DISASSEMBLY	24	0.38	1.23	0.19809523	0.46	1	1833	tags=25%, list=14%, signal=29%
NUCLEAR_MEMBRANE_PART	32	0.39	1.23	0.20987654	0.46	1	5109	tags=69%, list=40%, signal=114%
PURINE_NUCLEOTIDE_BINDING	127	0.29	1.23	0.15789473	0.46	1	3460	tags=34%, list=27%, signal=46%
DNA_DEPENDENT_ATPASE_ACTIVITY	17	0.47	1.23	0.234	0.45	1	1717	tags=29%, list=13%, signal=34%
DNA_CATABOLIC_PROCESS	15	0.42	1.23	0.20472442	0.45	1	936	tags=20%, list=7%, signal=22%
POSITIVE_REGULATION_OF_TRANSCRIPTION_FACTOR_ACTIVIT	17	0.44	1.23	0.21242484	0.45	1	4977	tags=65%, list=39%, signal=106%
PROTEIN_AMINO_ACID_LIPIDATION	22	0.43	1.23	0.25	0.45	1	4473	tags=59%, list=35%, signal=91%
INTERPHASE	46	0.35	1.23	0.21011673	0.46	1	2327	tags=30%, list=18%, signal=37%
THYROID_HORMONE_RECEPTOR_BINDING	17	0.44	1.22	0.21604939	0.46	1	3240	tags=47%, list=25%, signal=63%
SENSORY_PERCEPTION_OF_CHEMICAL_STIMULUS	13	0.47	1.22	0.23469388	0.46	1	3957	tags=38%, list=31%, signal=56%
INTRAMOLECULAR_OXIDOREDUCTASE_ACTIVITY	11	0.47	1.22	0.24089068	0.45	1	2593	tags=45%, list=20%, signal=57%
HYDROLASE_ACTIVITY__ACTING_ON_GLYCOSYL_BONDS	29	0.39	1.22	0.24898785	0.46	1	2982	tags=41%, list=23%, signal=54%
PROTEIN_MODIFICATION_BY_SMALL_PROTEIN_CONJUGATION	30	0.35	1.22	0.2038835	0.46	1	2304	tags=37%, list=18%, signal=45%

ADENYL_RIBONUCLEOTIDE_BINDING	102	0.30	1.22	0.19335938	0.46	1	3214	tags=32%, list=25%, signal=43%
DNA_DIRECTED_RNA_POLYMERASE_COMPLEX	15	0.41	1.22	0.22560975	0.46	1	2680	tags=47%, list=21%, signal=59%
RNA_POLYMERASE_COMPLEX	15	0.41	1.22	0.22560975	0.46	1	2680	tags=47%, list=21%, signal=59%
NUCLEAR_DNA_DIRECTED_RNA_POLYMERASE_COMPLEX	15	0.41	1.22	0.22560975	0.46	1	2680	tags=47%, list=21%, signal=59%
NEGATIVE_REGULATION_OF_TRANSCRIPTION_FACTOR_ACTIVIT	12	0.45	1.21	0.22874494	0.46	1	1295	tags=33%, list=10%, signal=37%
DOUBLE_STRANDED_RNA_BINDING	13	0.44	1.21	0.24752475	0.46	1	3200	tags=46%, list=25%, signal=62%
DAMAGED_DNA_BINDING	18	0.43	1.21	0.23839663	0.47	1	3819	tags=50%, list=30%, signal=71%
VESICLE	77	0.31	1.21	0.21472393	0.47	1	3545	tags=44%, list=28%, signal=61%
RESPONSE_TO_DRUG	11	0.46	1.21	0.26061776	0.47	1	1568	tags=36%, list=12%, signal=41%
PHOSPHOLIPID_METABOLIC_PROCESS	54	0.31	1.21	0.19465649	0.47	1	3902	tags=43%, list=31%, signal=61%
CARBOHYDRATE_METABOLIC_PROCESS	105	0.28	1.21	0.16532259	0.47	1	3233	tags=34%, list=25%, signal=46%
HOMEOSTASIS_OF_NUMBER_OF_CELLS	11	0.48	1.20	0.24796748	0.47	1	905	tags=18%, list=7%, signal=20%
ADENYL_NUCLEOTIDE_BINDING	105	0.29	1.20	0.20792079	0.47	1	3214	tags=32%, list=25%, signal=43%
RIBOSOMAL_SUBUNIT	19	0.45	1.20	0.28543308	0.47	1	5411	tags=74%, list=42%, signal=128%
MEMBRANE_BOUND_VESICLE	74	0.30	1.19	0.23838384	0.48	1	3545	tags=45%, list=28%, signal=61%
PROTEIN_UBIQUITINATION	27	0.36	1.19	0.22986248	0.48	1	2304	tags=37%, list=18%, signal=45%
HYDROLASE_ACTIVITY__ACTING_ON_CARBON_NITROGEN__BU	23	0.39	1.19	0.24329501	0.48	1	4171	tags=48%, list=33%, signal=71%
OXIDOREDUCTASE_ACTIVITY_GO_0016616	40	0.33	1.19	0.2263056	0.48	1	3174	tags=38%, list=25%, signal=50%
RIBOSOME	35	0.40	1.19	0.27572817	0.48	1	5188	tags=60%, list=41%, signal=101%
NEGATIVE_REGULATION_OF_DNA_BINDING	14	0.41	1.19	0.252505	0.48	1	2865	tags=43%, list=22%, signal=55%
MITOCHONDRIAL_TRANSPORT	15	0.49	1.19	0.29051384	0.48	1	4667	tags=80%, list=37%, signal=126%
N_ACETYLTRANSFERASE_ACTIVITY	17	0.42	1.19	0.2689243	0.48	1	3705	tags=41%, list=29%, signal=58%
ATPASE_ACTIVITY__COUPLED_TO_TRANSMEMBRANE_MOVEM	12	0.45	1.19	0.26734695	0.48	1	5007	tags=58%, list=39%, signal=96%
REGULATION_OF_BINDING	44	0.32	1.18	0.22709164	0.50	1	4981	tags=64%, list=39%, signal=104%
REGULATION_OF_ORGANELLE_ORGANIZATION_AND_BIOGENE	24	0.35	1.17	0.23135756	0.50	1	2288	tags=33%, list=18%, signal=41%
INDUCTION_OF_APOPTOSIS_BY_EXTRACELLULAR_SIGNALS	20	0.37	1.17	0.26907632	0.50	1	4072	tags=45%, list=32%, signal=66%
SPINDLE_POLE	11	0.44	1.17	0.25948104	0.50	1	2452	tags=36%, list=19%, signal=45%
NUCLEAR_CHROMOSOME	39	0.36	1.17	0.27095518	0.50	1	3755	tags=49%, list=29%, signal=69%
NUCLEAR_ENVELOPE	50	0.33	1.17	0.26104417	0.50	1	4106	tags=46%, list=32%, signal=68%
ELECTRON_TRANSPORT_GO_0006118	35	0.35	1.17	0.25562373	0.50	1	2160	tags=29%, list=17%, signal=34%
NUCLEAR_PORE	23	0.42	1.17	0.28247422	0.50	1	5810	tags=83%, list=46%, signal=151%
INDUCTION_OF_APOPTOSIS_BY_INTRACELLULAR_SIGNALS	14	0.40	1.17	0.2601156	0.50	1	2327	tags=36%, list=18%, signal=44%
DETECTION_OF_STIMULUS	21	0.40	1.17	0.2615694	0.50	1	5783	tags=57%, list=45%, signal=104%
LIPOPROTEIN_BIOSYNTHETIC_PROCESS	24	0.37	1.16	0.27606177	0.51	1	4473	tags=54%, list=35%, signal=83%
DNA_REPLICATION_INITIATION	11	0.42	1.16	0.26959848	0.51	1	1581	tags=36%, list=12%, signal=41%
COENZYME_METABOLIC_PROCESS	19	0.42	1.16	0.30452675	0.51	1	4915	tags=53%, list=38%, signal=85%
CELLULAR_CARBOHYDRATE_CATABOLIC_PROCESS	15	0.44	1.16	0.27504912	0.51	1	3233	tags=47%, list=25%, signal=62%
CARBOHYDRATE_CATABOLIC_PROCESS	15	0.44	1.16	0.27504912	0.51	1	3233	tags=47%, list=25%, signal=62%
INTERPHASE_OF_MITOTIC_CELL_CYCLE	43	0.35	1.16	0.29960316	0.51	1	2327	tags=30%, list=18%, signal=37%
NEGATIVE_REGULATION_OF_DNA_METABOLIC_PROCESS	11	0.46	1.16	0.2947977	0.51	1	2018	tags=36%, list=16%, signal=43%
MEDIATOR_COMPLEX	17	0.39	1.15	0.272	0.51	1	3647	tags=47%, list=29%, signal=66%
REGULATION_OF_CELLULAR_COMPONENT_ORGANIZATION_AI	76	0.27	1.15	0.22243346	0.52	1	2393	tags=24%, list=19%, signal=29%
PROTEIN_IMPORT_INTO_NUCLEUS	33	0.32	1.15	0.23320158	0.52	1	2165	tags=27%, list=17%, signal=33%
RECEPTOR_SIGNALING_PROTEIN_ACTIVITY	43	0.35	1.15	0.29718876	0.52	1	3615	tags=33%, list=28%, signal=45%
ATP_BINDING	98	0.28	1.14	0.25494072	0.53	1	3214	tags=31%, list=25%, signal=41%
MITOCHONDRION_ORGANIZATION_AND_BIOGENESIS	35	0.37	1.14	0.32874015	0.53	1	2534	tags=40%, list=20%, signal=50%
UBIQUITIN_CYCLE	33	0.34	1.14	0.30078125	0.53	1	2522	tags=36%, list=20%, signal=45%
GTPASE_ACTIVITY	61	0.34	1.14	0.3177388	0.53	1	1542	tags=21%, list=12%, signal=24%
DNA_DIRECTED_RNA_POLYMERASE_II__CORE_COMPLEX	11	0.41	1.14	0.29622266	0.53	1	2622	tags=45%, list=21%, signal=57%
MAGNESIUM_ION_BINDING	38	0.33	1.13	0.31075698	0.54	1	2141	tags=24%, list=17%, signal=28%
CENTROSOME	46	0.33	1.13	0.27237353	0.54	1	1328	tags=17%, list=10%, signal=19%
NUCLEAR_MEMBRANE	38	0.34	1.13	0.2915811	0.54	1	5109	tags=66%, list=40%, signal=109%
ANTIOXIDANT_ACTIVITY	11	0.46	1.13	0.32515338	0.54	1	2539	tags=36%, list=20%, signal=45%
RESPONSE_TO_OXIDATIVE_STRESS	30	0.34	1.13	0.31889763	0.54	1	3355	tags=50%, list=26%, signal=68%
REGULATION_OF_CELLULAR_PROTEIN_METABOLIC_PROCESS	98	0.27	1.12	0.27961165	0.55	1	1980	tags=20%, list=16%, signal=24%
INTRINSIC_TO_GOLGI_MEMBRANE	10	0.45	1.12	0.33200794	0.56	1	712	tags=20%, list=6%, signal=21%
PROTEIN_SECRETION	19	0.37	1.12	0.3174905	0.55	1	2613	tags=32%, list=20%, signal=40%
RNA_POLYMERASE_II_TRANSCRIPTION_FACTOR_ACTIVITY	132	0.25	1.11	0.2749004	0.56	1	1375	tags=16%, list=11%, signal=18%
LIPOPROTEIN_METABOLIC_PROCESS	30	0.34	1.11	0.32427186	0.56	1	3121	tags=37%, list=24%, signal=48%
ACTIVATION_OF_NF_KAPPAB_TRANSCRIPTION_FACTOR	11	0.44	1.11	0.3493724	0.56	1	2590	tags=36%, list=20%, signal=46%
GOLGI_STACK	12	0.44	1.11	0.3806706	0.57	1	4454	tags=50%, list=35%, signal=77%
GLYCEROPHOSPHOLIPID_BIOSYNTHETIC_PROCESS	26	0.35	1.11	0.3458498	0.57	1	4473	tags=58%, list=35%, signal=89%
TRANSCRIPTION_INITIATION	30	0.35	1.11	0.32244897	0.57	1	3668	tags=43%, list=29%, signal=61%
MITOCHONDRIAL_LUMEN	40	0.36	1.10	0.37295082	0.57	1	3771	tags=48%, list=30%, signal=67%
MITOCHONDRIAL_MATRIX	40	0.36	1.10	0.37295082	0.56	1	3771	tags=48%, list=30%, signal=67%
RESPONSE_TO_ABIOTIC_STIMULUS	52	0.28	1.10	0.2889734	0.56	1	3573	tags=35%, list=28%, signal=48%
PORE_COMPLEX	26	0.38	1.10	0.3319672	0.56	1	5109	tags=65%, list=40%, signal=109%
RNA_EXPORT_FROM_NUCLEUS	17	0.41	1.10	0.34210527	0.56	1	4106	tags=59%, list=32%, signal=87%
JAK_STAT_CASCADE	20	0.39	1.10	0.34607646	0.56	1	3533	tags=45%, list=28%, signal=62%
STEROID_BIOSYNTHETIC_PROCESS	17	0.36	1.10	0.34824902	0.56	1	2847	tags=41%, list=22%, signal=53%
CENTROSOME_ORGANIZATION_AND_BIOGENESIS	13	0.41	1.10	0.34189722	0.56	1	4687	tags=54%, list=37%, signal=85%
MICROTUBULE_ORGANIZING_CENTER_ORGANIZATION_AND_B	13	0.41	1.10	0.34189722	0.56	1	4687	tags=54%, list=37%, signal=85%
HORMONE_RECEPTOR_BINDING	26	0.34	1.10	0.3305785	0.56	1	3240	tags=38%, list=25%, signal=51%
GAMETE_GENERATION	68	0.27	1.10	0.29821074	0.56	1	1737	tags=18%, list=14%, signal=20%
REGULATION_OF_PROTEIN_METABOLIC_PROCESS	109	0.26	1.10	0.3043478	0.56	1	2069	tags=20%, list=16%, signal=24%
ISOMERASE_ACTIVITY	21	0.35	1.10	0.344	0.56	1	1012	tags=24%, list=8%, signal=26%
ACTIVATION_OF_JNK_ACTIVITY	10	0.44	1.10	0.35211268	0.56	1	3580	tags=60%, list=28%, signal=83%
THIOLESTER_HYDROLASE_ACTIVITY	10	0.43	1.10	0.37166324	0.56	1	4852	tags=60%, list=38%, signal=97%
RESPONSE_TO_TEMPERATURE_STIMULUS	10	0.45	1.09	0.35	0.56	1	2484	tags=40%, list=19%, signal=50%
ACETYLTRANSFERASE_ACTIVITY	21	0.35	1.09	0.36565655	0.56	1	2398	tags=29%, list=19%, signal=35%
EXTRINSIC_TO_MEMBRANE	16	0.36	1.09	0.33271375	0.56	1	1379	tags=25%, list=11%, signal=28%
SEXUAL_REPRODUCTION	84	0.26	1.09	0.29069766	0.56	1	2136	tags=19%, list=17%, signal=23%
AMINO_ACID_METABOLIC_PROCESS	39	0.29	1.09	0.3211382	0.56	1	4868	tags=54%, list=38%, signal=87%
CARBOHYDRATE_TRANSPORT	11	0.39	1.09	0.34	0.56	1	508	tags=18%, list=4%, signal=19%
PHOSPHOINOSITIDE_METABOLIC_PROCESS	25	0.35	1.09	0.354	0.56	1	4473	tags=64%, list=35%, signal=98%
CHROMATIN_ASSEMBLY	11	0.42	1.09	0.34511435	0.56	1	3346	tags=45%, list=26%, signal=62%
ENVELOPE	121	0.27	1.08	0.33333334	0.57	1	4106	tags=40%, list=32%, signal=59%
ORGANELLE_ENVELOPE	121	0.27	1.08	0.33333334	0.57	1	4106	tags=40%, list=32%, signal=59%
PROTEIN_SERINE_THREONINE_PHOSPHATASE_ACTIVITY	15	0.38	1.08	0.34980237	0.57	1	494	tags=13%, list=4%, signal=14%
CONDENSED_NUCLEAR_CHROMOSOME	13	0.43	1.08	0.39173228	0.57	1	3735	tags=69%, list=29%, signal=98%
CYTOSKELETON_DEPENDENT_INTRACELLULAR_TRANSPORT	16	0.35	1.08	0.37084872	0.57	1	2521	tags=38%, list=20%, signal=47%
PEPTIDE_BINDING	48	0.27	1.08	0.33657587	0.57	1	318	tags=8%, list=2%, signal=9%
CASPASE_ACTIVATION	18	0.36	1.07	0.37240076	0.58	1	2236	tags=33%, list=18%, signal=40%
ACTIN_CYTOSKELETON	75	0.27	1.07	0.3398058	0.58	1	2524	tags=28%, list=20%, signal=35%
VIRAL_GENOME_REPLICATION	10	0.40	1.07	0.37708333	0.58	1	3366	tags=50%, list=26%, signal=68%

SISTER_CHROMATID_SEGREGATION	13	0.44	1.07	0.41247484	0.58	1	4088	tags=62%, list=32%, signal=90%
MITOTIC_SISTER_CHROMATID_SEGREGATION	12	0.46	1.07	0.40695298	0.58	1	4088	tags=67%, list=32%, signal=98%
ANTIGEN_BINDING	13	0.40	1.06	0.37816763	0.59	1	4128	tags=31%, list=32%, signal=45%
GENERAL_RNA_POLYMERASE_II_TRANSCRIPTION_FACTOR_ACT	29	0.31	1.06	0.3627451	0.60	1	1375	tags=17%, list=11%, signal=19%
NEGATIVE_REGULATION_OF_TRANSPORT	15	0.38	1.06	0.42168674	0.60	1	1114	tags=27%, list=9%, signal=29%
ORGANIC_ACID_TRANSMEMBRANE_TRANSPORTER_ACTIVITY	24	0.32	1.05	0.39513108	0.60	1	3379	tags=38%, list=26%, signal=51%
TRANSLATIONAL_INITIATION	27	0.32	1.05	0.3578732	0.60	1	4702	tags=44%, list=37%, signal=70%
GLUCOSE_METABOLIC_PROCESS	18	0.35	1.05	0.39250493	0.60	1	3233	tags=44%, list=25%, signal=59%
CARBOXYLIC_ACID_TRANSMEMBRANE_TRANSPORTER_ACTIVIT	23	0.32	1.05	0.39926064	0.60	1	3379	tags=39%, list=26%, signal=53%
VACUOLAR_TRANSPORT	10	0.42	1.05	0.40433925	0.61	1	2193	tags=50%, list=17%, signal=60%
INORGANIC_CATION_TRANSMEMBRANE_TRANSPORTER_ACTIV	29	0.32	1.05	0.38709676	0.61	1	4903	tags=48%, list=38%, signal=78%
AMINO_ACID_TRANSMEMBRANE_TRANSPORTER_ACTIVITY	11	0.38	1.04	0.39096266	0.61	1	1941	tags=27%, list=15%, signal=32%
TRANSCRIPTION_INITIATION_FROM_RNA_POLYMERASE_II_PRO	25	0.34	1.04	0.39805827	0.61	1	1375	tags=24%, list=11%, signal=27%
TRANSLATION	121	0.26	1.04	0.38430583	0.62	1	4581	tags=40%, list=36%, signal=61%
RNA_POLYMERASE_ACTIVITY	13	0.39	1.04	0.38342968	0.62	1	2680	tags=38%, list=21%, signal=49%
PROTEIN_IMPORT	46	0.27	1.04	0.4008016	0.62	1	2417	tags=24%, list=19%, signal=29%
POSITIVE_REGULATION_OF_CASPASE_ACTIVITY	19	0.34	1.03	0.41984734	0.62	1	2236	tags=32%, list=18%, signal=38%
NUCLEAR_HORMONE_RECEPTOR_BINDING	25	0.32	1.03	0.42411643	0.63	1	3240	tags=36%, list=25%, signal=48%
CALCIUM_INDEPENDENT_CELL_CELL_ADHESION	10	0.43	1.03	0.42883548	0.63	1	4842	tags=70%, list=38%, signal=113%
CHROMATIN_REMODELING	17	0.36	1.02	0.43004116	0.64	1	2340	tags=29%, list=18%, signal=36%
ENDOSOME_TRANSPORT	20	0.36	1.02	0.40079364	0.63	1	1993	tags=30%, list=16%, signal=35%
COVALENT_CHROMATIN_MODIFICATION	16	0.37	1.02	0.4228457	0.63	1	2782	tags=38%, list=22%, signal=48%
HISTONE_MODIFICATION	16	0.37	1.02	0.4228457	0.63	1	2782	tags=38%, list=22%, signal=48%
COFACTOR_METABOLIC_PROCESS	28	0.34	1.02	0.42975205	0.63	1	5483	tags=57%, list=43%, signal=100%
GLYCEROPHOSPHOLIPID_METABOLIC_PROCESS	33	0.30	1.02	0.42714572	0.63	1	3902	tags=48%, list=31%, signal=70%
MICROTUBULE_CYTOSKELETON_ORGANIZATION_AND_BIOGEN	21	0.34	1.02	0.43564355	0.63	1	1563	tags=24%, list=12%, signal=27%
ATPASE_ACTIVITY__COUPLED_TO_TRANSMEMBRANE_MOVEM	11	0.40	1.02	0.42505133	0.63	1	5007	tags=55%, list=39%, signal=90%
FEMALE_GAMETE_GENERATION	12	0.35	1.02	0.43083003	0.63	1	2636	tags=25%, list=21%, signal=31%
INTERMEDIATE_FILAMENT_CYTOSKELETON	10	0.39	1.02	0.42610365	0.63	1	2687	tags=30%, list=21%, signal=38%
INTERMEDIATE_FILAMENT	10	0.39	1.02	0.42610365	0.63	1	2687	tags=30%, list=21%, signal=38%
MICROTUBULE_BINDING	21	0.31	1.02	0.4224806	0.63	1	2291	tags=33%, list=18%, signal=41%
STRUCTURE_SPECIFIC_DNA_BINDING	40	0.29	1.01	0.43991855	0.63	1	3905	tags=45%, list=31%, signal=65%
TRANSPORT_VESICLE	24	0.32	1.01	0.41883767	0.63	1	2934	tags=46%, list=23%, signal=59%
CELL_CELL_ADHESION	36	0.29	1.01	0.4232143	0.63	1	5514	tags=64%, list=43%, signal=112%
CYTOKINE_AND_CHEMOKINE_MEDIATED_SIGNALING_PATHWA	12	0.37	1.01	0.43505156	0.63	1	2443	tags=33%, list=19%, signal=41%
POSITIVE_REGULATION_OF_SIGNAL_TRANSDUCTION	83	0.28	1.01	0.43366337	0.63	1	3167	tags=30%, list=25%, signal=40%
CATION_TRANSPORT	55	0.26	1.01	0.4448743	0.64	1	1412	tags=18%, list=11%, signal=20%
HYDROLASE_ACTIVITY__HYDROLYZING_O_GLYCOSYL_COMPOU	20	0.35	1.01	0.4473161	0.64	1	3428	tags=45%, list=27%, signal=61%
ANDROGEN_RECEPTOR_SIGNALING_PATHWAY	12	0.40	1.01	0.43639922	0.64	1	3240	tags=42%, list=25%, signal=56%
POST_GOLGI_VESICLE_MEDIATED_TRANSPORT	10	0.39	1.00	0.44268775	0.64	1	3151	tags=40%, list=25%, signal=53%
NUCLEAR_ORGANIZATION_AND_BIOGENESIS	20	0.33	1.00	0.46414343	0.65	1	958	tags=15%, list=8%, signal=16%
SUGAR_BINDING	18	0.36	1.00	0.48393196	0.65	1	5490	tags=72%, list=43%, signal=127%
ACTIVE_TRANSMEMBRANE_TRANSPORTER_ACTIVITY	71	0.26	1.00	0.45736435	0.65	1	2548	tags=24%, list=20%, signal=30%
POSITIVE_REGULATION_OF_JNK_ACTIVITY	11	0.40	1.00	0.48023716	0.65	1	3580	tags=55%, list=28%, signal=76%
RESPONSE_TO_RADIATION	39	0.26	1.00	0.45109782	0.65	1	3573	tags=36%, list=28%, signal=50%
ORGANIC_ACID_TRANSPORT	22	0.30	0.99	0.4598131	0.65	1	3429	tags=41%, list=27%, signal=56%
ACTIN_FILAMENT_BINDING	12	0.36	0.99	0.4413519	0.65	1	2324	tags=42%, list=18%, signal=51%
ENDOSOME	48	0.28	0.99	0.4490196	0.66	1	3818	tags=35%, list=30%, signal=50%
CHROMATIN_REMODELING_COMPLEX	12	0.36	0.99	0.44186047	0.66	1	2948	tags=42%, list=23%, signal=54%
NITROGEN_COMPOUND_METABOLIC_PROCESS	88	0.23	0.99	0.49306932	0.66	1	4459	tags=47%, list=35%, signal=71%
RNA_POLYMERASE_II_TRANSCRIPTION_MEDIATOR_ACTIVITY	12	0.39	0.98	0.49387756	0.66	1	3647	tags=42%, list=29%, signal=58%
CARBOXYLIC_ACID_TRANSPORT	21	0.30	0.98	0.4803002	0.67	1	3429	tags=43%, list=27%, signal=58%
TRANSCRIPTION_FACTOR_TFIID_COMPLEX	14	0.34	0.97	0.4940476	0.67	1	1042	tags=14%, list=8%, signal=16%
REGULATION_OF_NUCLEOCYTOPLASMIC_TRANSPORT	15	0.33	0.97	0.49425286	0.68	1	3427	tags=47%, list=27%, signal=64%
POSITIVE_REGULATION_OF_HYDROLASE_ACTIVITY	29	0.28	0.97	0.49250937	0.68	1	4245	tags=41%, list=33%, signal=62%
HOMEOSTATIC_PROCESS	109	0.24	0.97	0.48501873	0.69	1	4161	tags=31%, list=33%, signal=46%
CELL_CORTEX_PART	11	0.36	0.96	0.507874	0.69	1	1505	tags=27%, list=12%, signal=31%
REGULATION_OF_TRANSPORT	35	0.26	0.96	0.52884614	0.70	1	2865	tags=34%, list=22%, signal=44%
NUCLEAR_CHROMOSOME_PART	26	0.31	0.96	0.5046211	0.70	1	3755	tags=42%, list=29%, signal=60%
SOLUBLE_FRACTION	100	0.23	0.95	0.5389105	0.72	1	1801	tags=17%, list=14%, signal=20%
METAL_ION_TRANSPORT	43	0.25	0.95	0.53154874	0.72	1	1313	tags=16%, list=10%, signal=18%
N_METHYLTRANSFERASE_ACTIVITY	11	0.37	0.94	0.5146771	0.72	1	3247	tags=45%, list=25%, signal=61%
MICROBODY	38	0.30	0.94	0.5031185	0.72	1	4275	tags=45%, list=33%, signal=67%
PEROXISOME	38	0.30	0.94	0.5031185	0.72	1	4275	tags=45%, list=33%, signal=67%
TRANS_GOLGI_NETWORK_TRANSPORT_VESICLE	10	0.38	0.94	0.5253165	0.72	1	4004	tags=60%, list=31%, signal=87%
MICROTUBULE_ORGANIZING_CENTER	53	0.26	0.94	0.5293006	0.72	1	1328	tags=15%, list=10%, signal=17%
REGULATION_OF_RESPONSE_TO_STIMULUS	34	0.29	0.94	0.5510204	0.72	1	2742	tags=26%, list=21%, signal=34%
DEVELOPMENT_OF_PRIMARY_SEXUAL_CHARACTERISTICS	17	0.31	0.93	0.56041664	0.73	1	4154	tags=47%, list=33%, signal=70%
DETECTION_OF_EXTERNAL_STIMULUS	12	0.35	0.93	0.5781893	0.73	1	5783	tags=58%, list=45%, signal=107%
ION_TRANSPORT	71	0.23	0.93	0.56	0.73	1	1465	tags=17%, list=11%, signal=19%
CALCIUM_ION_BINDING	51	0.26	0.93	0.5536723	0.73	1	2613	tags=27%, list=20%, signal=34%
HISTONE_ACETYLTRANSFERASE_ACTIVITY	14	0.34	0.92	0.56435645	0.74	1	3705	tags=36%, list=29%, signal=50%
EPIDERMIS_DEVELOPMENT	37	0.25	0.92	0.5785582	0.74	1	3738	tags=38%, list=29%, signal=53%
ION_HOMEOSTASIS	65	0.25	0.92	0.56561923	0.74	1	5007	tags=42%, list=39%, signal=68%
CYTOSKELETON_ORGANIZATION_AND_BIOGENESIS	123	0.21	0.92	0.6149312	0.75	1	2521	tags=22%, list=20%, signal=27%
SINGLE_STRANDED_DNA_BINDING	26	0.28	0.92	0.5705996	0.75	1	3819	tags=42%, list=30%, signal=60%
CHEMICAL_HOMEOSTASIS	81	0.24	0.91	0.5785582	0.75	1	5007	tags=42%, list=39%, signal=69%
REGULATION_OF_PROTEIN_STABILITY	16	0.31	0.91	0.61354584	0.75	1	4463	tags=44%, list=35%, signal=67%
TUBULIN_BINDING	28	0.28	0.91	0.5882353	0.76	1	2430	tags=32%, list=19%, signal=40%
REGULATION_OF_SIGNAL_TRANSDUCTION	136	0.22	0.90	0.5859375	0.76	1	3059	tags=25%, list=24%, signal=33%
REPLICATION_FORK	15	0.34	0.90	0.57593685	0.76	1	2137	tags=33%, list=17%, signal=40%
REGULATION_OF_PROTEIN_SECRETION	13	0.32	0.90	0.58285713	0.76	1	2590	tags=31%, list=20%, signal=39%
OXIDOREDUCTASE_ACTIVITY__ACTING_ON_NADH_OR_NADPH	19	0.30	0.90	0.5839695	0.76	1	2708	tags=42%, list=21%, signal=53%
RESPONSE_TO_VIRUS	35	0.29	0.89	0.60474306	0.78	1	3204	tags=34%, list=25%, signal=46%
NEGATIVE_REGULATION_OF_TRANSCRIPTION_FROM_RNA_PO	65	0.21	0.89	0.6574803	0.78	1	3268	tags=32%, list=26%, signal=43%
MITOCHONDRIAL_PART	111	0.25	0.89	0.5767717	0.78	1	4062	tags=40%, list=32%, signal=58%
TIGHT_JUNCTION	14	0.32	0.89	0.60733944	0.78	1	5124	tags=57%, list=40%, signal=95%
LIGASE_ACTIVITY__FORMING_CARBON_OXYGEN_BONDS	12	0.36	0.89	0.5894539	0.78	1	3714	tags=42%, list=29%, signal=59%
POSITIVE_REGULATION_OF_DEVELOPMENTAL_PROCESS	125	0.20	0.89	0.66729677	0.78	1	2791	tags=22%, list=22%, signal=27%
PROTEIN_COMPLEX_ASSEMBLY	108	0.20	0.89	0.6817289	0.78	1	3903	tags=33%, list=31%, signal=48%
SH3_DOMAIN_BINDING	12	0.32	0.89	0.5992063	0.78	1	2011	tags=33%, list=16%, signal=40%
UBIQUITIN_LIGASE_COMPLEX	21	0.28	0.88	0.6386719	0.78	1	957	tags=19%, list=7%, signal=21%
ZINC_ION_BINDING	57	0.22	0.88	0.67896676	0.78	1	4171	tags=46%, list=33%, signal=67%
REGULATION_OF_JNK_ACTIVITY	12	0.34	0.88	0.62795275	0.78	1	3580	tags=50%, list=28%, signal=69%
TRANSFERASE_ACTIVITY__TRANSFERRING_PENTOSYL_GROUPS	12	0.31	0.88	0.6490486	0.78	1	4560	tags=58%, list=36%, signal=91%

HEART_DEVELOPMENT	16	0.30	0.88	0.65275145	0.78	1	3419	tags=31%, list=27%, signal=43%
AMINO_ACID_TRANSPORT	12	0.31	0.87	0.62352943	0.79	1	3429	tags=42%, list=27%, signal=57%
ORGANELLE_OUTER_MEMBRANE	16	0.32	0.87	0.6401591	0.79	1	4106	tags=56%, list=32%, signal=83%
OUTER_MEMBRANE	16	0.32	0.87	0.6401591	0.79	1	4106	tags=56%, list=32%, signal=83%
PROTEIN_COMPLEX_DISASSEMBLY	13	0.32	0.87	0.6219512	0.79	1	4929	tags=54%, list=39%, signal=88%
RESPONSE_TO_CARBOHYDRATE_STIMULUS	10	0.34	0.87	0.60305345	0.79	1	2484	tags=30%, list=19%, signal=37%
TRANS_GOLGI_NETWORK	18	0.29	0.87	0.62204725	0.79	1	5007	tags=67%, list=39%, signal=110%
POSITIVE_REGULATION_OF_RESPONSE_TO_STIMULUS	24	0.27	0.87	0.63188976	0.79	1	2258	tags=21%, list=18%, signal=25%
CATION_BINDING	119	0.20	0.86	0.75140184	0.80	1	2466	tags=24%, list=19%, signal=29%
STEROID_BINDING	14	0.32	0.86	0.6086106	0.80	1	3852	tags=50%, list=30%, signal=72%
METALLOENDOPEPTIDASE_ACTIVITY	18	0.29	0.86	0.66542053	0.80	1	1455	tags=22%, list=11%, signal=25%
COFACTOR_BIOSYNTHETIC_PROCESS	11	0.34	0.86	0.64908725	0.80	1	5799	tags=73%, list=45%, signal=133%
RUFFLE	22	0.29	0.86	0.6499069	0.81	1	4217	tags=50%, list=33%, signal=75%
CELLULAR_PROTEIN_COMPLEX_DISASSEMBLY	12	0.34	0.85	0.6486486	0.81	1	4929	tags=58%, list=39%, signal=95%
TRANSCRIPTION_FROM_RNA_POLYMERASE_III_PROMOTER	17	0.27	0.85	0.6826923	0.81	1	4376	tags=47%, list=34%, signal=72%
RESPONSE_TO_OTHER_ORGANISM	51	0.27	0.85	0.65483236	0.81	1	3572	tags=35%, list=28%, signal=49%
CELLULAR_HOMEOSTASIS	74	0.22	0.85	0.7059925	0.81	1	3718	tags=27%, list=29%, signal=38%
PEROXISOMAL_MEMBRANE	11	0.35	0.84	0.6699801	0.82	1	1344	tags=27%, list=11%, signal=30%
PEROXISOMAL_PART	11	0.35	0.84	0.6699801	0.82	1	1344	tags=27%, list=11%, signal=30%
MICROBODY_MEMBRANE	11	0.35	0.84	0.6699801	0.82	1	1344	tags=27%, list=11%, signal=30%
MICROBODY_PART	11	0.35	0.84	0.6699801	0.82	1	1344	tags=27%, list=11%, signal=30%
VACUOLE	40	0.25	0.84	0.6764706	0.81	1	2368	tags=25%, list=19%, signal=31%
MAINTENANCE_OF_CELLULAR_LOCALIZATION	11	0.32	0.84	0.6633065	0.82	1	1114	tags=18%, list=9%, signal=20%
INTERACTION_WITH_HOST	13	0.31	0.84	0.69153225	0.82	1	1	tags=8%, list=0%, signal=8%
ANION_CATION_SYMPORTER_ACTIVITY	10	0.33	0.84	0.65055764	0.82	1	2342	tags=30%, list=18%, signal=37%
S_PHASE	10	0.36	0.84	0.6396917	0.82	1	4088	tags=50%, list=32%, signal=73%
REGULATION_OF_PROTEIN_MODIFICATION_PROCESS	30	0.25	0.84	0.67045456	0.81	1	1980	tags=27%, list=16%, signal=31%
POSITIVE_REGULATION_OF_CELL_PROLIFERATION	77	0.22	0.83	0.68577075	0.82	1	2509	tags=25%, list=20%, signal=31%
EARLY_ENDOSOME	16	0.30	0.83	0.65773195	0.82	1	4401	tags=44%, list=34%, signal=67%
MAINTENANCE_OF_CELLULAR_PROTEIN_LOCALIZATION	10	0.32	0.83	0.692	0.82	1	1114	tags=20%, list=9%, signal=22%
PROTEIN_DIMERIZATION_ACTIVITY	110	0.19	0.83	0.8201581	0.83	1	2430	tags=20%, list=19%, signal=24%
NEGATIVE_REGULATION_OF_TRANSFERASE_ACTIVITY	24	0.25	0.82	0.69754255	0.83	1	4391	tags=42%, list=34%, signal=63%
REGULATION_OF_INTRACELLULAR_TRANSPORT	17	0.27	0.82	0.7159309	0.83	1	2061	tags=29%, list=16%, signal=35%
GENERATION_OF_NEURONS	24	0.24	0.82	0.7470817	0.83	1	628	tags=8%, list=5%, signal=9%
REGULATION_OF_BLOOD_PRESSURE	11	0.32	0.82	0.68880457	0.83	1	5515	tags=82%, list=43%, signal=144%
EXCITATORY_EXTRACELLULAR_LIGAND_GATED_ION_CHANNEL_	11	0.33	0.82	0.6891089	0.83	1	1465	tags=18%, list=11%, signal=21%
EXTRACELLULAR_LIGAND_GATED_ION_CHANNEL_ACTIVITY	11	0.33	0.82	0.6891089	0.83	1	1465	tags=18%, list=11%, signal=21%
CLATHRIN_COATED_VESICLE	24	0.26	0.81	0.72912425	0.85	1	4202	tags=54%, list=33%, signal=81%
MACROMOLECULAR_COMPLEX_DISASSEMBLY	14	0.29	0.81	0.72210956	0.85	1	4929	tags=50%, list=39%, signal=81%
REGIONALIZATION	12	0.29	0.81	0.75243664	0.85	1	3905	tags=42%, list=31%, signal=60%
MRNA_BINDING	17	0.27	0.80	0.74413645	0.85	1	3859	tags=47%, list=30%, signal=67%
VIRAL_REPRODUCTIVE_PROCESS	20	0.24	0.79	0.7826962	0.86	1	2669	tags=25%, list=21%, signal=32%
POSITIVE_REGULATION_OF_PEPTIDYL_TYROSINE_PHOSPHORYI	12	0.30	0.79	0.742115	0.86	1	2742	tags=33%, list=21%, signal=42%
MEMBRANE_ORGANIZATION_AND_BIOGENESIS	81	0.22	0.79	0.7514231	0.87	1	4502	tags=44%, list=35%, signal=68%
MAINTENANCE_OF_LOCALIZATION	18	0.25	0.79	0.74653465	0.87	1	3427	tags=28%, list=27%, signal=38%
LIGAND_GATED_CHANNEL_ACTIVITY	18	0.27	0.79	0.7221135	0.87	1	2833	tags=28%, list=22%, signal=36%
REGULATION_OF_CELL_ADHESION	18	0.25	0.78	0.7861507	0.87	1	1174	tags=17%, list=9%, signal=18%
ECTODERM_DEVELOPMENT	42	0.21	0.78	0.81784385	0.88	1	3738	tags=36%, list=29%, signal=50%
RESPONSE_TO_BACTERIUM	14	0.30	0.78	0.7247525	0.87	1	5783	tags=64%, list=45%, signal=117%
DEFENSE_RESPONSE_TO_BACTERIUM	14	0.30	0.78	0.7247525	0.87	1	5783	tags=64%, list=45%, signal=117%
METALLOPEPTIDASE_ACTIVITY	30	0.23	0.78	0.83914727	0.87	1	2652	tags=27%, list=21%, signal=34%
MICROTUBULE_BASED_MOVEMENT	10	0.28	0.78	0.808	0.87	1	2158	tags=30%, list=17%, signal=36%
STEROID_HORMONE_RECEPTOR_SIGNALING_PATHWAY	18	0.27	0.78	0.76099426	0.87	1	3240	tags=33%, list=25%, signal=45%
POSITIVE_REGULATION_OF_CELL_DIFFERENTIATION	11	0.30	0.78	0.78352493	0.87	1	3856	tags=45%, list=30%, signal=65%
PROTEIN_POLYMERIZATION	10	0.30	0.78	0.7410359	0.87	1	1418	tags=20%, list=11%, signal=22%
PROTEASE_INHIBITOR_ACTIVITY	20	0.28	0.77	0.75095785	0.87	1	3472	tags=30%, list=27%, signal=41%
MAINTENANCE_OF_PROTEIN_LOCALIZATION	12	0.28	0.77	0.7654321	0.87	1	1114	tags=17%, list=9%, signal=18%
GENERATION_OF_PRECURSOR_METABOLITES_AND_ENERGY	85	0.19	0.77	0.851272	0.87	1	2160	tags=19%, list=17%, signal=23%
POTASSIUM_CHANNEL_ACTIVITY	11	0.30	0.77	0.7585513	0.87	1	1996	tags=18%, list=16%, signal=22%
INTRACELLULAR_RECEPTOR_MEDIATED_SIGNALING_PATHWAY	19	0.27	0.77	0.7601547	0.87	1	3240	tags=32%, list=25%, signal=42%
REGULATION_OF_HYDROLASE_ACTIVITY	46	0.21	0.77	0.83820665	0.87	1	3166	tags=26%, list=25%, signal=35%
CARBOHYDRATE_BINDING	34	0.23	0.77	0.7873563	0.87	1	4440	tags=44%, list=35%, signal=67%
RESPONSE_TO_NUTRIENT_LEVELS	12	0.29	0.77	0.788	0.87	1	6139	tags=67%, list=48%, signal=128%
STEROID_METABOLIC_PROCESS	42	0.22	0.77	0.81070745	0.87	1	4064	tags=38%, list=32%, signal=56%
REGULATION_OF_TRANSFERASE_ACTIVITY	95	0.18	0.77	0.87356323	0.87	1	3028	tags=23%, list=24%, signal=30%
MEMBRANE_FUSION	20	0.25	0.76	0.76704544	0.87	1	2187	tags=20%, list=17%, signal=24%
REGULATION_OF_SECRETION	19	0.25	0.76	0.80761904	0.87	1	4610	tags=47%, list=36%, signal=74%
PROTEIN_N_TERMINUS_BINDING	27	0.21	0.75	0.8674464	0.89	1	3366	tags=33%, list=26%, signal=45%
ORGANELLE_INNER_MEMBRANE	60	0.23	0.75	0.77045906	0.89	1	2708	tags=25%, list=21%, signal=32%
SERINE_TYPE_PEPTIDASE_ACTIVITY	19	0.26	0.74	0.8073218	0.89	1	4013	tags=32%, list=31%, signal=46%
CATION_HOMEOSTASIS	53	0.20	0.74	0.8517824	0.89	1	5007	tags=42%, list=39%, signal=68%
SERINE_HYDROLASE_ACTIVITY	20	0.26	0.74	0.8151751	0.90	1	4013	tags=30%, list=31%, signal=44%
RESPONSE_TO_ORGANIC_SUBSTANCE	19	0.24	0.74	0.83953035	0.90	1	2484	tags=26%, list=19%, signal=33%
EXOPEPTIDASE_ACTIVITY	15	0.26	0.74	0.8134172	0.90	1	2486	tags=27%, list=19%, signal=33%
REGULATION_OF_KINASE_ACTIVITY	93	0.17	0.73	0.92761904	0.90	1	3028	tags=23%, list=24%, signal=29%
DETECTION_OF_STIMULUS_INVOLVED_IN_SENSORY_PERCEPTI	12	0.28	0.73	0.81707317	0.90	1	4949	tags=33%, list=39%, signal=54%
FEEDING_BEHAVIOR	12	0.26	0.73	0.8228347	0.90	1	2686	tags=25%, list=21%, signal=32%
GLYCOLIPID_METABOLIC_PROCESS	11	0.28	0.73	0.79113925	0.90	1	5372	tags=55%, list=42%, signal=94%
G_PROTEIN_COUPLED_RECEPTOR_BINDING	24	0.25	0.73	0.80078894	0.90	1	2829	tags=29%, list=22%, signal=37%
CELLULAR_CATION_HOMEOSTASIS	51	0.20	0.72	0.87642586	0.91	1	5007	tags=41%, list=39%, signal=67%
PEPTIDYL_TYROSINE_PHOSPHORYLATION	21	0.25	0.72	0.82608694	0.91	1	1776	tags=19%, list=14%, signal=22%
NEUROGENESIS	31	0.21	0.72	0.9041916	0.91	1	1716	tags=13%, list=13%, signal=15%
B_CELL_ACTIVATION	11	0.27	0.71	0.8151951	0.91	1	1734	tags=18%, list=14%, signal=21%
MITOCHONDRIAL_MEMBRANE	66	0.22	0.71	0.8121331	0.91	1	4062	tags=36%, list=32%, signal=53%
REGULATION_OF_RHO_PROTEIN_SIGNAL_TRANSDUCTION	12	0.26	0.71	0.84555984	0.91	1	3059	tags=25%, list=24%, signal=33%
OXIDOREDUCTASE_ACTIVITY__ACTING_ON_THE_CH_CH_GROU	18	0.26	0.71	0.8358209	0.91	1	2668	tags=22%, list=21%, signal=28%
POSITIVE_REGULATION_OF_CYTOKINE_PRODUCTION	12	0.27	0.71	0.8352941	0.91	1	4966	tags=67%, list=39%, signal=109%
MITOCHONDRIAL_INNER_MEMBRANE	54	0.23	0.70	0.82669324	0.92	1	2708	tags=24%, list=21%, signal=30%
REGULATION_OF_RAS_PROTEIN_SIGNAL_TRANSDUCTION	13	0.26	0.70	0.8368932	0.92	1	3059	tags=23%, list=24%, signal=30%
INOSITOL_OR_PHOSPHATIDYLINOSITOL_KINASE_ACTIVITY	10	0.27	0.70	0.871308	0.92	1	4580	tags=60%, list=36%, signal=93%
MITOCHONDRIAL_ENVELOPE	72	0.20	0.70	0.84765625	0.92	1	4062	tags=36%, list=32%, signal=53%
POSITIVE_REGULATION_OF_CATALYTIC_ACTIVITY	89	0.17	0.69	0.9570094	0.92	1	2988	tags=24%, list=23%, signal=31%
RESPONSE_TO_EXTRACELLULAR_STIMULUS	14	0.26	0.69	0.85119045	0.92	1	6437	tags=64%, list=50%, signal=130%
REPRODUCTIVE_PROCESS	92	0.16	0.69	0.9706458	0.93	1	2669	tags=17%, list=21%, signal=22%
PEROXISOME_ORGANIZATION_AND_BIOGENESIS	15	0.26	0.68	0.8467909	0.93	1	1194	tags=13%, list=9%, signal=15%

REGULATION_OF_IMMUNE_EFFECTOR_PROCESS	12	0.27	0.68	0.8416834	0.93	1	4586	tags=33%, list=36%, signal=52%
G2_M_TRANSITION_OF_MITOTIC_CELL_CYCLE	10	0.27	0.67	0.870021	0.93	1	1787	tags=20%, list=14%, signal=23%
REGULATION_OF_CYTOKINE_SECRETION	10	0.27	0.67	0.85880077	0.94	1	2590	tags=30%, list=20%, signal=38%
POSITIVE_REGULATION_OF_TRANSLATION	20	0.23	0.66	0.8888889	0.95	1	1621	tags=15%, list=13%, signal=17%
PROTEIN_OLIGOMERIZATION	20	0.21	0.66	0.91698116	0.95	1	3853	tags=40%, list=30%, signal=57%
MITOCHONDRIAL_OUTER_MEMBRANE	12	0.25	0.65	0.8968254	0.95	1	4057	tags=50%, list=32%, signal=73%
POLYSACCHARIDE_METABOLIC_PROCESS	10	0.25	0.65	0.9140625	0.95	1	3428	tags=40%, list=27%, signal=55%
MONOVALENT_INORGANIC_CATION_TRANSMEMBRANE_TRAN	17	0.24	0.65	0.8646465	0.95	1	4893	tags=41%, list=38%, signal=67%
LIPID_TRANSPORTER_ACTIVITY	17	0.23	0.65	0.8704062	0.95	1	3911	tags=41%, list=31%, signal=59%
SERINE_TYPE_ENDOPEPTIDASE_ACTIVITY	17	0.23	0.65	0.91468257	0.95	1	1222	tags=12%, list=10%, signal=13%
JNK_CASCADE	26	0.18	0.62	0.9412916	0.97	1	3999	tags=35%, list=31%, signal=50%
VIRAL_INFECTIOUS_CYCLE	16	0.20	0.62	0.94093686	0.97	1	3366	tags=31%, list=26%, signal=42%
NUCLEOTIDE_EXCISION_REPAIR	16	0.20	0.62	0.95472443	0.97	1	4152	tags=38%, list=33%, signal=56%
REGULATION_OF_BODY_FLUID_LEVELS	31	0.19	0.61	0.9410569	0.97	1	977	tags=10%, list=8%, signal=10%
OXIDOREDUCTASE_ACTIVITY_GO_0016705	20	0.21	0.60	0.9186047	0.97	1	914	tags=10%, list=7%, signal=11%
CELL_SURFACE	40	0.16	0.60	0.9753788	0.97	1	2338	tags=18%, list=18%, signal=21%
CYTOKINE_SECRETION	12	0.24	0.60	0.91102517	0.97	1	2590	tags=25%, list=20%, signal=31%
EXTRACELLULAR_STRUCTURE_ORGANIZATION_AND_BIOGENES	13	0.24	0.60	0.9032258	0.97	1	4807	tags=46%, list=38%, signal=74%
REGULATION_OF_CATABOLIC_PROCESS	13	0.21	0.60	0.9522863	0.97	1	3107	tags=23%, list=24%, signal=30%
STRESS_ACTIVATED_PROTEIN_KINASE_SIGNALING_PATHWAY	27	0.17	0.59	0.9634615	0.97	1	3999	tags=33%, list=31%, signal=48%
PROTEIN_BINDING__BRIDGING	35	0.18	0.59	0.9765166	0.97	1	2873	tags=20%, list=23%, signal=26%
AMINE_TRANSPORT	20	0.20	0.58	0.9496982	0.97	1	3429	tags=30%, list=27%, signal=41%
KINASE_BINDING	37	0.16	0.58	0.9902724	0.97	1	3317	tags=24%, list=26%, signal=33%
LOCOMOTORY_BEHAVIOR	41	0.19	0.57	0.93711966	0.98	1	2499	tags=20%, list=20%, signal=24%
TASTE_RECEPTOR_ACTIVITY	14	0.20	0.54	0.97109824	0.99	1	3880	tags=21%, list=30%, signal=31%
HYDROGEN_ION_TRANSMEMBRANE_TRANSPORTER_ACTIVITY	16	0.20	0.54	0.9720559	0.99	1	4893	tags=38%, list=38%, signal=61%
POSITIVE_REGULATION_OF_PROTEIN_MODIFICATION_PROCES	22	0.18	0.53	0.98087955	0.99	1	1776	tags=18%, list=14%, signal=21%
PROTEIN_PHOSPHATASE_TYPE_2A_REGULATOR_ACTIVITY	11	0.19	0.50	0.988	1.00	1	4050	tags=27%, list=32%, signal=40%
NEGATIVE_REGULATION_OF_MULTICELLULAR_ORGANISMAL_F	17	0.18	0.50	0.9864865	1.00	1	2434	tags=18%, list=19%, signal=22%
LYSOSOME_ORGANIZATION_AND_BIOGENESIS	11	0.20	0.49	0.9617591	0.99	1	3132	tags=27%, list=25%, signal=36%
VACUOLE_ORGANIZATION_AND_BIOGENESIS	11	0.20	0.49	0.9617591	0.99	1	3132	tags=27%, list=25%, signal=36%
STRUCTURAL_CONSTITUENT_OF_RIBOSOME	64	0.18	0.48	0.94572026	0.99	1	5130	tags=42%, list=40%, signal=70%
REGULATION_OF_CYTOKINE_BIOSYNTHETIC_PROCESS	20	0.17	0.47	0.9846154	0.99	1	273	tags=5%, list=2%, signal=5%
CELLULAR_MORPHOGENESIS_DURING_DIFFERENTIATION	12	0.17	0.45	0.9943925	0.99	1	5124	tags=42%, list=40%, signal=70%
