# Supplementary material for: Integrated Genomic and Epigenomic Analysis of Breast Cancer Brain Metastasis
Source: PLoS One. 2014 Jan 29;9(1):e85448. doi: 10.1371/journal.pone.0085448 (PMC3906004; doi:10.1371/journal.pone.0085448)
Supplement: File S1 — Supporting figures and tables. Figure S1: Combined Network for Upstream Analysis of FOXM1 and TBX2. The downstream genes connected to FOXM1 and TBX2 were illustrated as a network in IPA. The mRNA expression ratios are listed below the gene nodes. The legend within figure describes the node and edge color keys. Figure S2: Word Cloud Analysis of Cluster Enrichments. We have used word clouds to visually summarize the textual results from the enrichment analysis of each gene cluster as observed in Figure 3. The results were generated using www.wordle.net web resource. The larger the word, the more times it is mentioned in the enrichment categories. Supplementary Tables in File S1. Table S1a. Table S1b. Table S2. Table S3a. Table S3b. Table S4a. Figure S1. Table S4b. Table S5a–b. Table S6a–b. Table S7. Table S8a–f. Table S9a–f. Figure S2. Table S10. Table S11a–c. Table S11d. Table S12. Table S13. Table S14. (ZIP) [file pone.0085448.s001.zip › Supplementary Table S5b.pdf]

Supplementary Table 5b. GSE analysis using the c5 gene set library (GSEA|MSigDB) containing one gene set for downregulated genes in BBM samples

ES: enrichment score; NES: normalized enrichment score; FDR: false discovery rate; FWER: familywise error rate

Column descriptions

GENE SET: Gene set name. For MSigDB gene sets, the description is the gene set page on the GSEA web site.
SIZE: Number of genes in the gene set after filtering out those genes not in the expression dataset
ES: Enrichment score for the gene set; that is, the degree to which this gene set is overrepresented at the top or bottom of the ranked list of genes in the expression dataset.
NES: Normalized enrichment score; that is, the enrichment score for the gene set after it has been normalized across analyzed gene sets.
NOM p-value: Nominal p value; that is, the statistical significance of the enrichment score. The nominal p value is not adjusted for gene set size or multiple hypothesis testing; therefore, it is of limited use in comparing gene sets.
FDR q-value: False discovery rate; that is, the estimated probability that the normalized enrichment score represents a false positive finding.
FWER p-value: Familywise-error rate; that is, a more conservatively estimated probability that the normalized enrichment score represents a false positive finding. Because the goal of GSEA is to generate hypotheses, the GSEA team recommends focusing on the FDR statistic.
RANK AT MAX: The position in the ranked list at which the maximum enrichment score occurred. The more interesting gene sets achieve the maximum

Gene Set name	SIZE	ES	NES	NOM p-val	FDR q-val	FWER p-val	RANK AT MAX	LEADING EDGE
EXTRACELLULAR_MATRIX	39	-0.55	-1.73	0.01	0.43277043	0.834	2310	tags=44%, list=18%, signal=53%
PROTEINACEOUS_EXTRACELLULAR_MATRIX	39	-0.55	-1.73	0.01	0.51932454	0.834	2310	tags=44%, list=18%, signal=53%
TRANSMEMBRANE_RECEPTOR_PROTEIN_TYROSINE_KINASE_SIGNALING_PATHWAY	45	-0.48	-1.61	0.03	0.530348	0.978	1088	tags=24%, list=9%, signal=27%
GTPASE_ACTIVATOR_ACTIVITY	30	-0.52	-1.62	0.02	0.54063404	0.975	841	tags=30%, list=7%, signal=32%
RNA_POLYMERASE_II_TRANSCRIPTION_FACTOR_ACTIVITY__ENHANCER_BINDING	11	-0.65	-1.63	0.03	0.5437872	0.968	391	tags=27%, list=3%, signal=28%
ADHERENS_JUNCTION	10	-0.74	-1.63	0.02	0.56255335	0.967	2198	tags=70%, list=17%, signal=84%
CARBOXYLESTERASE_ACTIVITY	19	-0.57	-1.66	0.02	0.5935924	0.938	3035	tags=47%, list=24%, signal=62%
CONTRACTILE_FIBER	12	-0.61	-1.63	0.01	0.6058745	0.965	585	tags=42%, list=5%, signal=44%
KINASE_REGULATOR_ACTIVITY	29	-0.47	-1.57	0.02	0.60871065	0.993	779	tags=24%, list=6%, signal=26%
REGULATION_OF_G_PROTEIN_COUPLED_RECEPTOR_PROTEIN_SIGNALING_PATHWAY	10	-0.72	-1.64	0.02	0.6166015	0.953	1549	tags=50%, list=12%, signal=57%
CENTRAL_NERVOUS_SYSTEM_DEVELOPMENT	62	-0.41	-1.56	0.00	0.61765575	0.996	2470	tags=34%, list=19%, signal=42%
STRIATED_MUSCLE_CONTRACTION_GO_0006941	10	-0.68	-1.67	0.01	0.62686574	0.929	594	tags=30%, list=5%, signal=31%
BASAL_LAMINA	10	-0.79	-1.73	0.00	0.6370621	0.831	759	tags=50%, list=6%, signal=53%
TRANSMEMBRANE_RECEPTOR_PROTEIN_PHOSPHATASE_ACTIVITY	10	-0.67	-1.58	0.03	0.6371529	0.991	844	tags=40%, list=7%, signal=43%
ENZYME_LINKED_RECEPTOR_PROTEIN_SIGNALING_PATHWAY	80	-0.41	-1.53	0.02	0.6391122	0.998	1088	tags=20%, list=9%, signal=22%
EPIDERMAL_GROWTH_FACTOR_RECEPTOR_SIGNALING_PATHWAY	11	-0.68	-1.57	0.05	0.6232552	0.992	2198	tags=55%, list=17%, signal=66%
CAMP_MEDIATED_SIGNALING	30	-0.48	-1.52	0.04	0.6520349	0.999	1192	tags=27%, list=9%, signal=29%
NEGATIVE_REGULATION_OF_CELL_DIFFERENTIATION	13	-0.56	-1.53	0.05	0.6655811	0.997	1996	tags=46%, list=16%, signal=55%
VOLTAGE_GATED_CATION_CHANNEL_ACTIVITY	19	-0.57	-1.55	0.05	0.6080266	0.996	1145	tags=26%, list=9%, signal=29%
GTPASE_REGULATOR_ACTIVITY	71	-0.38	-1.47	0.04	0.73224485	1	1094	tags=21%, list=9%, signal=23%
CONTRACTILE_FIBER_PART	10	-0.57	-1.49	0.05	0.7630492	1	585	tags=40%, list=5%, signal=42%
PROTEIN_KINASE_REGULATOR_ACTIVITY	25	-0.46	-1.48	0.04	0.7780941	1	779	tags=24%, list=6%, signal=26%
STRUCTURAL_CONSTITUENT_OF_MUSCLE	18	-0.63	-1.73	0.00	0.83588415	0.821	1190	tags=39%, list=9%, signal=43%
VOLTAGE_GATED_CHANNEL_ACTIVITY	24	-0.52	-1.48	0.06	0.7812467	1	1145	tags=21%, list=9%, signal=23%
EXTRACELLULAR_MATRIX_PART	20	-0.68	-1.77	0.00	0.8558732	0.723	2310	tags=60%, list=18%, signal=73%
CALCIUM_CHANNEL_ACTIVITY	14	-0.54	-1.47	0.07	0.7810272	1	1145	tags=29%, list=9%, signal=31%
GLUTATHIONE_TRANSFERASE_ACTIVITY	12	-0.66	-1.47	0.08	0.7575875	1	2730	tags=58%, list=21%, signal=74%
BASEMENT_MEMBRANE	17	-0.72	-1.78	0.00	1	0.666	1765	tags=53%, list=14%, signal=61%
BASOLATERAL_PLASMA_MEMBRANE	19	-0.56	-1.47	0.10	0.70779395	1	2278	tags=47%, list=18%, signal=58%
PROTEIN_COMPLEX_BINDING	38	-0.42	-1.47	0.05	0.69626814	1	380	tags=16%, list=3%, signal=16%
OXIDOREDUCTASE_ACTIVITY__ACTING_ON_THE_ALDEHYDE_OR_OXO_GROUP_OF_DONORS	12	-0.54	-1.45	0.08	0.74294823	1	2931	tags=50%, list=23%, signal=65%
G_PROTEIN_SIGNALING__COUPLED_TO_CAMP_NUCLEOTIDE_SECOND_MESSENGER	29	-0.45	-1.43	0.08	0.829003	1	1192	tags=24%, list=9%, signal=27%
SYNAPTIC_TRANSMISSION	72	-0.36	-1.43	0.06	0.80496967	1	2121	tags=32%, list=17%, signal=38%
PHAGOCYTOSIS	10	-0.63	-1.42	0.09	0.80396295	1	749	tags=40%, list=6%, signal=42%
INTEGRIN_BINDING	18	-0.53	-1.41	0.11	0.8255811	1	2192	tags=44%, list=17%, signal=54%
SPECIFIC_RNA_POLYMERASE_II_TRANSCRIPTION_FACTOR_ACTIVITY	25	-0.42	-1.41	0.07	0.817344	1	969	tags=24%, list=8%, signal=26%
CELL_JUNCTION	36	-0.43	-1.40	0.10	0.83593476	1	2271	tags=36%, list=18%, signal=44%
MUSCLE_DEVELOPMENT	42	-0.41	-1.39	0.10	0.84534603	1	2313	tags=33%, list=18%, signal=41%
REGULATION_OF_CELL_SHAPE	10	-0.58	-1.38	0.13	0.86309254	1	2179	tags=40%, list=17%, signal=48%
PROTEIN_KINASE_INHIBITOR_ACTIVITY	15	-0.49	-1.38	0.12	0.8458863	1	1909	tags=40%, list=15%, signal=47%
KINASE_INHIBITOR_ACTIVITY	15	-0.49	-1.38	0.12	0.8252549	1	1909	tags=40%, list=15%, signal=47%
CYTOSOLIC_PART	14	-0.54	-1.38	0.16	0.80850947	1	1810	tags=50%, list=14%, signal=58%
CALMODULIN_BINDING	15	-0.49	-1.37	0.13	0.8367341	1	665	tags=27%, list=5%, signal=28%
REGULATION_OF_MUSCLE_CONTRACTION	10	-0.52	-1.36	0.11	0.8524759	1	2559	tags=40%, list=20%, signal=50%
OXYGEN_BINDING	14	-0.52	-1.36	0.14	0.8505888	1	3685	tags=64%, list=29%, signal=90%
ENZYME_ACTIVATOR_ACTIVITY	73	-0.35	-1.35	0.08	0.84392005	1	2144	tags=33%, list=17%, signal=39%
REGULATION_OF_ANATOMICAL_STRUCTURE_MORPHOGENESIS	12	-0.56	-1.35	0.16	0.8349203	1	2179	tags=42%, list=17%, signal=50%
CELL_PROJECTION_BIOGENESIS	16	-0.47	-1.35	0.12	0.8374836	1	3241	tags=44%, list=25%, signal=59%
BRAIN_DEVELOPMENT	29	-0.42	-1.34	0.12	0.83965176	1	891	tags=24%, list=7%, signal=26%
AMINO_ACID_DERIVATIVE_METABOLIC_PROCESS	12	-0.50	-1.34	0.14	0.83038896	1	1259	tags=50%, list=10%, signal=55%
FEMALE_PREGNANCY	26	-0.46	-1.33	0.16	0.86886615	1	1060	tags=19%, list=8%, signal=21%
HORMONE_METABOLIC_PROCESS	19	-0.45	-1.33	0.15	0.85718817	1	1259	tags=32%, list=10%, signal=35%
SODIUM_ION_TRANSPORT	10	-0.51	-1.31	0.13	0.8878755	1	2654	tags=50%, list=21%, signal=63%
CELL_MATRIX_ADHESION	20	-0.44	-1.31	0.12	0.9006277	1	1707	tags=35%, list=13%, signal=40%
TRANSFERASE_ACTIVITY__TRANSFERRING_SULFUR_CONTAINING_GROUPS	20	-0.47	-1.29	0.19	0.9782232	1	2200	tags=40%, list=17%, signal=48%
AMINE_RECEPTOR_ACTIVITY	19	-0.46	-1.28	0.18	0.9697406	1	3519	tags=42%, list=28%, signal=58%
SULFUR_METABOLIC_PROCESS	21	-0.46	-1.27	0.19	0.9905573	1	2285	tags=43%, list=18%, signal=52%
REGULATION_OF_ANGIOGENESIS	12	-0.50	-1.27	0.20	0.99594325	1	2189	tags=50%, list=17%, signal=60%
SULFOTRANSFERASE_ACTIVITY	17	-0.49	-1.27	0.21	0.99342877	1	2200	tags=47%, list=17%, signal=57%
CELL_SUBSTRATE_ADHESION	21	-0.42	-1.26	0.17	0.9837131	1	2192	tags=38%, list=17%, signal=46%
CYTOSKELETAL_PROTEIN_BINDING	82	-0.31	-1.26	0.12	0.96846116	1	1526	tags=22%, list=12%, signal=25%
REGULATION_OF_CELL_MORPHOGENESIS	11	-0.53	-1.26	0.22	0.9769528	1	2179	tags=36%, list=17%, signal=44%
CHLORIDE_CHANNEL_ACTIVITY	10	-0.49	-1.26	0.19	0.9700942	1	838	tags=20%, list=7%, signal=21%
BIOGENIC_AMINE_METABOLIC_PROCESS	10	-0.48	-1.24	0.20	1	1	1259	tags=50%, list=10%, signal=55%
PHOSPHATASE_REGULATOR_ACTIVITY	18	-0.42	-1.22	0.24	1	1	1269	tags=22%, list=10%, signal=25%
COFACTOR_BINDING	11	-0.48	-1.22	0.24	1	1	1025	tags=36%, list=8%, signal=40%
INSULIN_RECEPTOR_SIGNALING_PATHWAY	11	-0.53	-1.22	0.25	1	1	3255	tags=64%, list=25%, signal=85%
ACTIN_CYTOSKELETON_ORGANIZATION_AND_BIOGENESIS	63	-0.33	-1.22	0.20	1	1	1912	tags=29%, list=15%, signal=33%
SUBSTRATE_SPECIFIC_CHANNEL_ACTIVITY	62	-0.32	-1.21	0.20	1	1	1425	tags=19%, list=11%, signal=22%
ACTIN_BINDING	36	-0.35	-1.21	0.22	1	1	501	tags=17%, list=4%, signal=17%
INORGANIC_ANION_TRANSMEMBRANE_TRANSPORTER_ACTIVITY	12	-0.45	-1.21	0.24	1	1	282	tags=17%, list=2%, signal=17%
TRANSMISSION_OF_NERVE_IMPULSE	81	-0.28	-1.20	0.18	1	1	2121	tags=28%, list=17%, signal=34%
FATTY_ACID_BETA_OXIDATION	11	-0.48	-1.20	0.26	1	1	3785	tags=73%, list=30%, signal=103%
LIPID_CATABOLIC_PROCESS	26	-0.37	-1.19	0.22	1	1	2291	tags=31%, list=18%, signal=37%
ORGAN_MORPHOGENESIS	71	-0.31	-1.19	0.22	1	1	2351	tags=37%, list=18%, signal=45%
RAS_GTPASE_ACTIVATOR_ACTIVITY	14	-0.46	-1.18	0.26	1	1	841	tags=21%, list=7%, signal=23%
TRANSFORMING_GROWTH_FACTOR_BETA_RECEPTOR_SIGNALING_PATHWAY	21	-0.38	-1.18	0.24	1	1	1031	tags=19%, list=8%, signal=21%
VASCULATURE_DEVELOPMENT	24	-0.37	-1.18	0.28	1	1	2189	tags=42%, list=17%, signal=50%

ACTIN_FILAMENT_ORGANIZATION	13	-0.43	-1.18	0.27	1	1	2198	tags=46%, list=17%, signal=56%
NEUROTRANSMITTER_BINDING	29	-0.35	-1.17	0.24	1	1	2939	tags=34%, list=23%, signal=45%
LIPASE_ACTIVITY	29	-0.36	-1.17	0.26	1	1	3035	tags=41%, list=24%, signal=54%
ENERGY_DERIVATION_BY_OXIDATION_OF_ORGANIC_COMPOUNDS	23	-0.39	-1.16	0.27	1	1	2454	tags=39%, list=19%, signal=48%
NEGATIVE_REGULATION_OF_TRANSCRIPTION__DNA_DEPENDENT	99	-0.26	-1.16	0.17	1	1	633	tags=14%, list=5%, signal=15%
NEGATIVE_REGULATION_OF_RNA_METABOLIC_PROCESS	99	-0.26	-1.16	0.17	1	1	633	tags=14%, list=5%, signal=15%
STRUCTURAL_CONSTITUENT_OF_CYTOSKELETON	28	-0.35	-1.16	0.26	1	1	1923	tags=29%, list=15%, signal=34%
CELLULAR_RESPIRATION	11	-0.53	-1.16	0.35	1	1	2905	tags=45%, list=23%, signal=59%
PROTEIN_TYROSINE_PHOSPHATASE_ACTIVITY	33	-0.33	-1.15	0.26	1	1	868	tags=21%, list=7%, signal=23%
HEMATOPOIETIN_INTERFERON_CLASS__D200_DOMAIN__CYTOKINE_RECEPTOR_BINDING	17	-0.43	-1.14	0.31	1	1	3133	tags=35%, list=25%, signal=47%
CYCLIC_NUCLEOTIDE_MEDIATED_SIGNALING	55	-0.31	-1.14	0.29	1	1	1192	tags=15%, list=9%, signal=16%
REGULATION_OF_CELL_DIFFERENTIATION	26	-0.36	-1.14	0.30	1	1	1996	tags=31%, list=16%, signal=36%
SH3_SH2_ADAPTOR_ACTIVITY	26	-0.36	-1.13	0.31	1	1	3792	tags=46%, list=30%, signal=66%
GUANYL_NUCLEOTIDE_EXCHANGE_FACTOR_ACTIVITY	26	-0.33	-1.13	0.29	1	1	980	tags=15%, list=8%, signal=17%
ION_CHANNEL_ACTIVITY	58	-0.30	-1.12	0.27	1	1	1425	tags=17%, list=11%, signal=19%
BONE_REMODELING	12	-0.41	-1.12	0.34	1	1	749	tags=17%, list=6%, signal=18%
TISSUE_REMODELING	12	-0.41	-1.12	0.34	1	1	749	tags=17%, list=6%, signal=18%
POSITIVE_REGULATION_OF_TRANSCRIPTION	93	-0.26	-1.11	0.28	1	1	922	tags=15%, list=7%, signal=16%
TRANSMEMBRANE_RECEPTOR_PROTEIN_SERINE_THREONINE_KINASE_SIGNALING_PATHWA	29	-0.34	-1.11	0.33	1	1	1031	tags=17%, list=8%, signal=19%
CELLULAR_LIPID_CATABOLIC_PROCESS	25	-0.34	-1.10	0.30	1	1	2291	tags=28%, list=18%, signal=34%
PROTEIN_HOMODIMERIZATION_ACTIVITY	69	-0.27	-1.10	0.32	1	1	2303	tags=29%, list=18%, signal=35%
TRANSCRIPTION_REPRESSOR_ACTIVITY	108	-0.25	-1.10	0.28	1	1	2664	tags=32%, list=21%, signal=41%
EXCRETION	16	-0.40	-1.10	0.35	1	1	1149	tags=25%, list=9%, signal=27%
CELL_MIGRATION	36	-0.32	-1.10	0.35	1	1	2305	tags=31%, list=18%, signal=37%
ACTIN_FILAMENT_BASED_PROCESS	70	-0.29	-1.09	0.33	1	1	1912	tags=26%, list=15%, signal=30%
MICROTUBULE_ASSOCIATED_COMPLEX	24	-0.37	-1.09	0.36	1	1	1892	tags=38%, list=15%, signal=44%
ENZYME_INHIBITOR_ACTIVITY	67	-0.28	-1.09	0.31	1	1	1948	tags=22%, list=15%, signal=26%
CATION_CHANNEL_ACTIVITY	43	-0.30	-1.08	0.34	1	1	1425	tags=19%, list=11%, signal=21%
ACTIVATION_OF_MAPK_ACTIVITY	20	-0.34	-1.08	0.35	1	1	565	tags=15%, list=4%, signal=16%
ANGIOGENESIS	19	-0.37	-1.08	0.36	1	1	2189	tags=42%, list=17%, signal=51%
POTASSIUM_ION_TRANSPORT	14	-0.38	-1.07	0.38	1	1	1875	tags=29%, list=15%, signal=33%
TRANSFERASE_ACTIVITY__TRANSFERRING_ALKYL_OR_ARYL__OTHER_THAN_METHYL__GROU	24	-0.33	-1.07	0.36	1	1	2134	tags=29%, list=17%, signal=35%
NEGATIVE_REGULATION_OF_TRANSCRIPTION	138	-0.24	-1.06	0.31	1	1	633	tags=13%, list=5%, signal=14%
SYNAPSE	11	-0.40	-1.05	0.40	1	1	4052	tags=45%, list=32%, signal=67%
G_PROTEIN_SIGNALING__COUPLED_TO_CYCLIC_NUCLEOTIDE_SECOND_MESSENGER	53	-0.29	-1.05	0.38	1	1	3566	tags=38%, list=28%, signal=52%
PHOSPHORIC_MONOESTER_HYDROLASE_ACTIVITY	65	-0.25	-1.05	0.35	1	1	868	tags=15%, list=7%, signal=16%
LAMELLIPODIUM	20	-0.35	-1.05	0.41	1	1	1912	tags=30%, list=15%, signal=35%
HYDRO_LYASE_ACTIVITY	16	-0.35	-1.05	0.40	1	1	512	tags=13%, list=4%, signal=13%
MOLECULAR_ADAPTOR_ACTIVITY	28	-0.32	-1.04	0.39	1	1	3792	tags=46%, list=30%, signal=66%
DEPHOSPHORYLATION	46	-0.28	-1.04	0.40	1	1	868	tags=17%, list=7%, signal=19%
MRNA_SPLICE_SITE_SELECTION	11	-0.39	-1.04	0.42	1	1	1003	tags=45%, list=8%, signal=49%
ANION_CHANNEL_ACTIVITY	11	-0.41	-1.04	0.41	1	1	838	tags=18%, list=7%, signal=19%
STRIATED_MUSCLE_DEVELOPMENT	14	-0.35	-1.04	0.40	1	1	1709	tags=21%, list=13%, signal=25%
ANION_TRANSMEMBRANE_TRANSPORTER_ACTIVITY	36	-0.31	-1.04	0.39	1	1	2646	tags=31%, list=21%, signal=38%
NEGATIVE_REGULATION_OF_CELL_PROLIFERATION	80	-0.23	-1.03	0.37	1	1	950	tags=13%, list=7%, signal=13%
TRANSMEMBRANE_RECEPTOR_PROTEIN_TYROSINE_KINASE_ACTIVITY	17	-0.38	-1.03	0.41	1	1	1991	tags=35%, list=16%, signal=42%
POSITIVE_REGULATION_OF_TRANSFERASE_ACTIVITY	44	-0.30	-1.03	0.40	1	1	1903	tags=20%, list=15%, signal=24%
REGULATION_OF_CYTOSKELETON_ORGANIZATION_AND_BIOGENESIS	17	-0.35	-1.03	0.41	1	1	1824	tags=35%, list=14%, signal=41%
MONOVALENT_INORGANIC_CATION_TRANSPORT	28	-0.30	-1.03	0.42	1	1	3216	tags=43%, list=25%, signal=57%
POSITIVE_REGULATION_OF_MAP_KINASE_ACTIVITY	22	-0.32	-1.03	0.42	1	1	565	tags=14%, list=4%, signal=14%
POSITIVE_REGULATION_OF_CELLULAR_COMPONENT_ORGANIZATION_AND_BIOGENESIS	24	-0.32	-1.03	0.42	1	1	2179	tags=38%, list=17%, signal=45%
PHOSPHOPROTEIN_PHOSPHATASE_ACTIVITY	48	-0.27	-1.03	0.42	1	1	868	tags=17%, list=7%, signal=18%
PHOSPHORIC_DIESTER_HYDROLASE_ACTIVITY	20	-0.34	-1.01	0.44	1	1	2901	tags=45%, list=23%, signal=58%
POSITIVE_REGULATION_OF_CELLULAR_METABOLIC_PROCESS	146	-0.22	-1.01	0.42	1	1	1118	tags=14%, list=9%, signal=16%
SMALL_GTPASE_BINDING	26	-0.30	-1.01	0.42	1	1	731	tags=15%, list=6%, signal=16%
POSITIVE_REGULATION_OF_NUCLEOBASE__NUCLEOSIDE__NUCLEOTIDE_AND_NUCLEIC_ACII	99	-0.23	-1.01	0.45	1	1	922	tags=14%, list=7%, signal=15%
HEMATOPOIETIN_INTERFERON_CLASS__D200_DOMAIN__CYTOKINE_RECEPTOR_ACTIVITY	17	-0.40	-1.01	0.48	1	1	1026	tags=24%, list=8%, signal=26%
POSITIVE_REGULATION_OF_TRANSCRIPTION__DNA_DEPENDENT	80	-0.24	-1.00	0.46	1	1	891	tags=14%, list=7%, signal=15%
MICROTUBULE_ORGANIZING_CENTER_PART	15	-0.36	-1.00	0.45	1	1	1427	tags=27%, list=11%, signal=30%
PHOSPHORIC_ESTER_HYDROLASE_ACTIVITY	85	-0.23	-1.00	0.47	1	1	880	tags=14%, list=7%, signal=15%
HEMOPOIESIS	42	-0.30	-1.00	0.46	1	1	3435	tags=43%, list=27%, signal=58%
GTPASE_BINDING	27	-0.30	-1.00	0.44	1	1	731	tags=15%, list=6%, signal=16%
INTERCELLULAR_JUNCTION	28	-0.31	-1.00	0.44	1	1	1479	tags=21%, list=12%, signal=24%
CELLULAR_PROTEIN_COMPLEX_ASSEMBLY	25	-0.28	-0.99	0.44	1	1	1031	tags=20%, list=8%, signal=22%
RHYTHMIC_PROCESS	14	-0.34	-0.99	0.47	1	1	318	tags=14%, list=2%, signal=15%
LIGAND_DEPENDENT_NUCLEAR_RECEPTOR_ACTIVITY	13	-0.34	-0.99	0.48	1	1	2118	tags=38%, list=17%, signal=46%
REGULATION_OF_GTPASE_ACTIVITY	11	-0.37	-0.99	0.46	1	1	950	tags=18%, list=7%, signal=20%
LYMPHOCYTE_DIFFERENTIATION	14	-0.37	-0.99	0.50	1	1	3078	tags=57%, list=24%, signal=75%
REGULATION_OF_MAPKKK_CASCADE	10	-0.38	-0.98	0.47	1	1	39	tags=10%, list=0%, signal=10%
SECOND_MESSENGER_MEDIATED_SIGNALING	77	-0.25	-0.98	0.47	1	1	870	tags=10%, list=7%, signal=11%
HEMOPOIETIC_OR_LYMPHOID_ORGAN_DEVELOPMENT	43	-0.29	-0.98	0.49	1	1	3435	tags=42%, list=27%, signal=57%
AMINE_BINDING	11	-0.35	-0.98	0.48	1	1	2575	tags=36%, list=20%, signal=46%
PROTEIN_TYROSINE_KINASE_ACTIVITY	24	-0.32	-0.97	0.46	1	1	1364	tags=25%, list=11%, signal=28%
G_PROTEIN_SIGNALING__COUPLED_TO_IP3_SECOND_MESSENGER__PHOSPHOLIPASE_C_AC	21	-0.31	-0.97	0.49	1	1	215	tags=10%, list=2%, signal=10%
AMINO_ACID_AND_DERIVATIVE_METABOLIC_PROCESS	51	-0.24	-0.97	0.51	1	1	485	tags=14%, list=4%, signal=14%
GATED_CHANNEL_ACTIVITY	45	-0.26	-0.96	0.52	1	1	1425	tags=16%, list=11%, signal=17%
SMALL_GTPASE_MEDIATED_SIGNAL_TRANSDUCTION	58	-0.27	-0.96	0.50	1	1	1576	tags=17%, list=12%, signal=20%
REGULATION_OF_PROTEIN_AMINO_ACID_PHOSPHORYLATION	19	-0.34	-0.96	0.51	1	1	2183	tags=37%, list=17%, signal=44%
REGULATION_OF_MULTICELLULAR_ORGANISMAL_PROCESS	78	-0.26	-0.96	0.50	1	1	3423	tags=35%, list=27%, signal=47%
POSITIVE_REGULATION_OF_RNA_METABOLIC_PROCESS	82	-0.22	-0.95	0.53	1	1	891	tags=13%, list=7%, signal=14%
AROMATIC_COMPOUND_METABOLIC_PROCESS	13	-0.34	-0.95	0.50	1	1	2285	tags=38%, list=18%, signal=47%
PHOSPHOINOSITIDE_MEDIATED_SIGNALING	22	-0.31	-0.95	0.53	1	1	215	tags=9%, list=2%, signal=9%
REGULATION_OF_MAP_KINASE_ACTIVITY	35	-0.29	-0.95	0.51	1	1	2427	tags=29%, list=19%, signal=35%
CARBON_OXYGEN_LYASE_ACTIVITY	19	-0.31	-0.94	0.55	1	1	512	tags=11%, list=4%, signal=11%
RESPONSE_TO_HYPOXIA	12	-0.33	-0.94	0.54	1	1	564	tags=17%, list=4%, signal=17%
REGULATION_OF_MYELOID_CELL_DIFFERENTIATION	10	-0.35	-0.94	0.54	1	1	85	tags=10%, list=1%, signal=10%
TRANSMEMBRANE_RECEPTOR_PROTEIN_KINASE_ACTIVITY	22	-0.32	-0.94	0.52	1	1	2997	tags=41%, list=23%, signal=53%
NADH_DEHYDROGENASE_COMPLEX	13	-0.42	-0.94	0.54	1	1	559	tags=23%, list=4%, signal=24%
MITOCHONDRIAL_RESPIRATORY_CHAIN_COMPLEX_I	13	-0.42	-0.94	0.54	1	1	559	tags=23%, list=4%, signal=24%
RESPIRATORY_CHAIN_COMPLEX_I	13	-0.42	-0.94	0.54	1	1	559	tags=23%, list=4%, signal=24%
TRANSCRIPTION_COREPRESSOR_ACTIVITY	73	-0.22	-0.94	0.55	1	1	2551	tags=30%, list=20%, signal=37%
NEGATIVE_REGULATION_OF_MAP_KINASE_ACTIVITY	11	-0.37	-0.93	0.51	1	1	2427	tags=45%, list=19%, signal=56%
EMBRYONIC_DEVELOPMENT	32	-0.27	-0.93	0.55	1	1	2929	tags=41%, list=23%, signal=53%
G_PROTEIN_SIGNALING__ADENYLATE_CYCLASE_ACTIVATING_PATHWAY	12	-0.32	-0.93	0.57	1	1	39	tags=8%, list=0%, signal=8%
SKELETAL_MUSCLE_DEVELOPMENT	11	-0.34	-0.92	0.59	1	1	1709	tags=18%, list=13%, signal=21%

G1_PHASE	10	-0.35	-0.92	0.53	1	1	847	tags=20%, list=7%, signal=21%
TRANSCRIPTION_ACTIVATOR_ACTIVITY	124	-0.21	-0.91	0.62	1	1	633	tags=11%, list=5%, signal=12%
DOUBLE_STRANDED_DNA_BINDING	21	-0.29	-0.91	0.61	1	1	374	tags=14%, list=3%, signal=15%
SENSORY_PERCEPTION	110	-0.21	-0.91	0.62	1	1	1253	tags=14%, list=10%, signal=15%
CARBON_CARBON_LYASE_ACTIVITY	10	-0.36	-0.91	0.56	1	1	2334	tags=30%, list=18%, signal=37%
NEGATIVE_REGULATION_OF_CATALYTIC_ACTIVITY	42	-0.24	-0.90	0.62	1	1	2427	tags=31%, list=19%, signal=38%
PERINUCLEAR_REGION_OF_CYTOPLASM	40	-0.26	-0.90	0.60	1	1	2189	tags=25%, list=17%, signal=30%
FATTY_ACID_OXIDATION	15	-0.33	-0.90	0.59	1	1	3785	tags=67%, list=30%, signal=95%
NEGATIVE_REGULATION_OF_CELL_CYCLE	44	-0.22	-0.90	0.66	1	1	1909	tags=25%, list=15%, signal=29%
TRANSCRIPTION_COACTIVATOR_ACTIVITY	95	-0.20	-0.90	0.69	1	1	994	tags=14%, list=8%, signal=15%
PROTEOGLYCAN_METABOLIC_PROCESS	10	-0.35	-0.89	0.60	1	1	1614	tags=40%, list=13%, signal=46%
PROTEIN_AMINO_ACID_DEPHOSPHORYLATION	40	-0.25	-0.89	0.62	1	1	868	tags=15%, list=7%, signal=16%
NEGATIVE_REGULATION_OF_GROWTH	24	-0.26	-0.88	0.63	1	1	627	tags=13%, list=5%, signal=13%
RESPONSE_TO_UV	20	-0.28	-0.88	0.63	1	1	947	tags=15%, list=7%, signal=16%
REGULATION_OF_DEFENSE_RESPONSE	12	-0.34	-0.88	0.58	1	1	749	tags=17%, list=6%, signal=18%
REGULATION_OF_GROWTH	34	-0.25	-0.87	0.68	1	1	2478	tags=26%, list=19%, signal=33%
ANATOMICAL_STRUCTURE_FORMATION	23	-0.28	-0.87	0.65	1	1	2189	tags=35%, list=17%, signal=42%
NEUROTRANSMITTER_RECEPTOR_ACTIVITY	27	-0.26	-0.87	0.65	1	1	2852	tags=30%, list=22%, signal=38%
POSITIVE_REGULATION_OF_MULTICELLULAR_ORGANISMAL_PROCESS	40	-0.27	-0.87	0.63	1	1	2198	tags=25%, list=17%, signal=30%
STRUCTURAL_MOLECULE_ACTIVITY	139	-0.23	-0.87	0.61	1	1	1190	tags=13%, list=9%, signal=14%
ACTIN_FILAMENT	12	-0.32	-0.87	0.61	1	1	950	tags=25%, list=7%, signal=27%
REGULATION_OF_PROTEIN_IMPORT_INTO_NUCLEUS	10	-0.34	-0.86	0.61	1	1	564	tags=20%, list=4%, signal=21%
INNATE_IMMUNE_RESPONSE	13	-0.32	-0.86	0.62	1	1	925	tags=23%, list=7%, signal=25%
CELL_CYCLE_ARREST_GO_0007050	32	-0.24	-0.86	0.70	1	1	938	tags=19%, list=7%, signal=20%
SMALL_GTPASE_REGULATOR_ACTIVITY	36	-0.26	-0.86	0.67	1	1	841	tags=11%, list=7%, signal=12%
GROWTH_FACTOR_ACTIVITY	32	-0.25	-0.86	0.65	1	1	428	tags=9%, list=3%, signal=10%
AMINE_METABOLIC_PROCESS	78	-0.20	-0.86	0.72	1	1	1614	tags=19%, list=13%, signal=22%
POSITIVE_REGULATION_OF_T_CELL_ACTIVATION	10	-0.39	-0.86	0.62	1	1	2940	tags=50%, list=23%, signal=65%
NEGATIVE_REGULATION_OF_SIGNAL_TRANSDUCTION	22	-0.27	-0.85	0.69	1	1	950	tags=14%, list=7%, signal=15%
ANION_TRANSPORT	14	-0.29	-0.85	0.67	1	1	2288	tags=36%, list=18%, signal=43%
GLYCOSAMINOGLYCAN_BINDING	15	-0.28	-0.85	0.68	1	1	877	tags=20%, list=7%, signal=21%
METAL_ION_TRANSMEMBRANE_TRANSPORTER_ACTIVITY	56	-0.22	-0.84	0.72	1	1	1425	tags=16%, list=11%, signal=18%
RAS_GTPASE_BINDING	18	-0.28	-0.84	0.67	1	1	703	tags=17%, list=6%, signal=18%
POSITIVE_REGULATION_OF_PROTEIN_AMINO_ACID_PHOSPHORYLATION	15	-0.31	-0.84	0.66	1	1	3071	tags=47%, list=24%, signal=61%
POSITIVE_REGULATION_OF_LYMPHOCYTE_ACTIVATION	13	-0.35	-0.84	0.65	1	1	2940	tags=46%, list=23%, signal=60%
ENZYME_BINDING	114	-0.19	-0.84	0.79	1	1	1112	tags=12%, list=9%, signal=13%
INTERLEUKIN_BINDING	14	-0.35	-0.84	0.64	1	1	3686	tags=57%, list=29%, signal=80%
PATTERN_SPECIFICATION_PROCESS	23	-0.26	-0.83	0.73	1	1	2920	tags=43%, list=23%, signal=56%
CELL_PROJECTION_PART	12	-0.32	-0.83	0.68	1	1	4536	tags=50%, list=36%, signal=77%
PHOSPHOLIPASE_ACTIVITY	23	-0.26	-0.83	0.73	1	1	2890	tags=35%, list=23%, signal=45%
MONOCARBOXYLIC_ACID_METABOLIC_PROCESS	47	-0.23	-0.82	0.71	1	1	700	tags=13%, list=5%, signal=13%
LYASE_ACTIVITY	41	-0.23	-0.82	0.76	1	1	2502	tags=24%, list=20%, signal=30%
POSITIVE_REGULATION_OF_TRANSPORT	14	-0.29	-0.82	0.70	1	1	749	tags=21%, list=6%, signal=23%
MAPKKK_CASCADE_GO_0000165	52	-0.22	-0.82	0.71	1	1	2894	tags=29%, list=23%, signal=37%
HORMONE_ACTIVITY	22	-0.27	-0.81	0.73	1	1	1174	tags=14%, list=9%, signal=15%
POSITIVE_REGULATION_OF_PHOSPHORYLATION	16	-0.28	-0.81	0.71	1	1	3071	tags=44%, list=24%, signal=58%
LIPOPROTEIN_BINDING	11	-0.35	-0.81	0.69	1	1	3965	tags=64%, list=31%, signal=92%
PIGMENT_METABOLIC_PROCESS	10	-0.32	-0.81	0.71	1	1	2196	tags=40%, list=17%, signal=48%
APICAL_JUNCTION_COMPLEX	17	-0.27	-0.81	0.73	1	1	1479	tags=18%, list=12%, signal=20%
APICOLATERAL_PLASMA_MEMBRANE	17	-0.27	-0.81	0.73	1	1	1479	tags=18%, list=12%, signal=20%
ION_TRANSMEMBRANE_TRANSPORTER_ACTIVITY	136	-0.19	-0.81	0.79	1	1	2454	tags=21%, list=19%, signal=25%
REGULATION_OF_MITOTIC_CELL_CYCLE	11	-0.30	-0.80	0.70	1	1	564	tags=18%, list=4%, signal=19%
CELL_ACTIVATION	37	-0.26	-0.80	0.73	1	1	3309	tags=41%, list=26%, signal=55%
PROTEIN_DOMAIN_SPECIFIC_BINDING	43	-0.22	-0.80	0.82	1	1	1912	tags=21%, list=15%, signal=25%
RAS_PROTEIN_SIGNAL_TRANSDUCTION	41	-0.25	-0.80	0.72	1	1	1016	tags=12%, list=8%, signal=13%
T_CELL_ACTIVATION	20	-0.30	-0.80	0.69	1	1	3309	tags=40%, list=26%, signal=54%
POLYSACCHARIDE_BINDING	16	-0.26	-0.79	0.76	1	1	877	tags=19%, list=7%, signal=20%
INACTIVATION_OF_MAPK_ACTIVITY	10	-0.33	-0.79	0.72	1	1	2427	tags=40%, list=19%, signal=49%
TRANSITION_METAL_ION_BINDING	69	-0.19	-0.79	0.86	1	1	1274	tags=16%, list=10%, signal=18%
PEPTIDYL_TYROSINE_MODIFICATION	23	-0.28	-0.79	0.71	1	1	2183	tags=35%, list=17%, signal=42%
EXTRACELLULAR_SPACE	132	-0.21	-0.78	0.74	1	1	3660	tags=36%, list=29%, signal=50%
REGULATION_OF_CYTOKINE_PRODUCTION	18	-0.25	-0.78	0.76	1	1	564	tags=11%, list=4%, signal=12%
RHODOPSIN_LIKE_RECEPTOR_ACTIVITY	75	-0.20	-0.78	0.79	1	1	3629	tags=29%, list=28%, signal=41%
REGULATION_OF_T_CELL_ACTIVATION	12	-0.33	-0.78	0.69	1	1	3309	tags=50%, list=26%, signal=67%
NUCLEAR_SPECK	10	-0.29	-0.78	0.76	1	1	783	tags=20%, list=6%, signal=21%
RESPONSE_TO_HORMONE_STIMULUS	20	-0.24	-0.78	0.80	1	1	991	tags=15%, list=8%, signal=16%
CARBOXYLIC_ACID_METABOLIC_PROCESS	93	-0.18	-0.77	0.87	1	1	700	tags=11%, list=5%, signal=11%
MITOCHONDRIAL_RESPIRATORY_CHAIN	20	-0.31	-0.77	0.71	1	1	559	tags=15%, list=4%, signal=16%
POSITIVE_REGULATION_OF_TRANSCRIPTION_FROM_RNA_POLYMERASE_II_PROMOTER	44	-0.20	-0.77	0.84	1	1	686	tags=11%, list=5%, signal=12%
ORGANIC_ACID_METABOLIC_PROCESS	95	-0.18	-0.77	0.87	1	1	700	tags=11%, list=5%, signal=11%
RECEPTOR_MEDIATED_ENDOCYTOSIS	20	-0.27	-0.77	0.75	1	1	1917	tags=30%, list=15%, signal=35%
REGULATION_OF_PHOSPHORYLATION	28	-0.23	-0.77	0.81	1	1	2183	tags=29%, list=17%, signal=34%
RESPONSE_TO_LIGHT_STIMULUS	30	-0.21	-0.76	0.86	1	1	947	tags=10%, list=7%, signal=11%
IMMUNE_SYSTEM_DEVELOPMENT	47	-0.21	-0.76	0.82	1	1	3435	tags=38%, list=27%, signal=52%
CYTOSOL	132	-0.18	-0.75	0.90	1	1	1423	tags=15%, list=11%, signal=17%
RHO_PROTEIN_SIGNAL_TRANSDUCTION	27	-0.24	-0.75	0.77	1	1	1016	tags=15%, list=8%, signal=16%
GROWTH	40	-0.21	-0.75	0.83	1	1	2478	tags=23%, list=19%, signal=28%
LEUKOCYTE_DIFFERENTIATION	20	-0.25	-0.75	0.78	1	1	3241	tags=45%, list=25%, signal=60%
POSITIVE_REGULATION_OF_PHOSPHATE_METABOLIC_PROCESS	18	-0.25	-0.74	0.81	1	1	2183	tags=28%, list=17%, signal=33%
MYELOID_CELL_DIFFERENTIATION	22	-0.24	-0.74	0.82	1	1	4217	tags=59%, list=33%, signal=88%
LEADING_EDGE	32	-0.23	-0.74	0.82	1	1	2198	tags=28%, list=17%, signal=34%
REGULATION_OF_LYMPHOCYTE_ACTIVATION	16	-0.29	-0.74	0.75	1	1	3309	tags=44%, list=26%, signal=59%
LEUKOCYTE_ACTIVATION	31	-0.24	-0.73	0.78	1	1	3309	tags=39%, list=26%, signal=52%
REGULATION_OF_SMALL_GTPASE_MEDIATED_SIGNAL_TRANSDUCTION	16	-0.26	-0.73	0.82	1	1	1426	tags=19%, list=11%, signal=21%
AMINO_SUGAR_METABOLIC_PROCESS	14	-0.26	-0.73	0.84	1	1	296	tags=14%, list=2%, signal=15%
GROWTH_FACTOR_BINDING	20	-0.24	-0.72	0.84	1	1	3435	tags=45%, list=27%, signal=61%
REGULATION_OF_IMMUNE_RESPONSE	23	-0.24	-0.72	0.82	1	1	2198	tags=26%, list=17%, signal=31%
CELL_PROJECTION	69	-0.19	-0.72	0.89	1	1	2305	tags=22%, list=18%, signal=26%
G_PROTEIN_COUPLED_RECEPTOR_ACTIVITY	98	-0.17	-0.72	0.91	1	1	3519	tags=30%, list=28%, signal=41%
PROTEIN_C_TERMINUS_BINDING	43	-0.18	-0.71	0.92	1	1	2699	tags=28%, list=21%, signal=35%
INFLAMMATORY_RESPONSE	69	-0.20	-0.71	0.84	1	1	2200	tags=20%, list=17%, signal=24%
PROTEIN_HOMOOLIGOMERIZATION	10	-0.27	-0.71	0.83	1	1	2801	tags=50%, list=22%, signal=64%
MULTI_ORGANISM_PROCESS	96	-0.19	-0.71	0.85	1	1	1082	tags=13%, list=8%, signal=14%
ACTIN_POLYMERIZATION_AND_OR_DEPOLYMERIZATION	15	-0.24	-0.71	0.86	1	1	2957	tags=33%, list=23%, signal=43%
POSITIVE_REGULATION_OF_CELLULAR_PROTEIN_METABOLIC_PROCESS	48	-0.20	-0.71	0.86	1	1	2183	tags=25%, list=17%, signal=30%

POSITIVE_REGULATION_OF_PROTEIN_METABOLIC_PROCESS	50	-0.20	-0.70	0.86	1	1	2183	tags=24%, list=17%, signal=29%
TISSUE_DEVELOPMENT	73	-0.18	-0.69	0.93	1	1	1405	tags=14%, list=11%, signal=15%
SECONDARY_ACTIVE_TRANSMEMBRANE_TRANSPORTER_ACTIVITY	31	-0.22	-0.69	0.87	1	1	2733	tags=32%, list=21%, signal=41%
REGULATION_OF_PROTEIN_KINASE_ACTIVITY	91	-0.17	-0.69	0.94	1	1	565	tags=8%, list=4%, signal=8%
PHOSPHOLIPID_BINDING	23	-0.21	-0.69	0.88	1	1	841	tags=13%, list=7%, signal=14%
LYTIC_VACUOLE	37	-0.21	-0.69	0.88	1	1	1815	tags=22%, list=14%, signal=25%
LYSOSOME	37	-0.21	-0.69	0.88	1	1	1815	tags=22%, list=14%, signal=25%
MALE_GONAD_DEVELOPMENT	11	-0.25	-0.68	0.92	1	1	114	tags=9%, list=1%, signal=9%
LIPID_BINDING	46	-0.19	-0.68	0.90	1	1	2931	tags=30%, list=23%, signal=39%
CHEMOKINE_ACTIVITY	19	-0.25	-0.68	0.85	1	1	3416	tags=42%, list=27%, signal=57%
CHEMOKINE_RECEPTOR_BINDING	19	-0.25	-0.68	0.85	1	1	3416	tags=42%, list=27%, signal=57%
SKELETAL_DEVELOPMENT	45	-0.20	-0.68	0.90	1	1	1916	tags=18%, list=15%, signal=21%
VIRAL_REPRODUCTION	23	-0.20	-0.68	0.90	1	1	2607	tags=35%, list=20%, signal=44%
RESPONSE_TO_WOUNDING	99	-0.19	-0.68	0.89	1	1	2200	tags=19%, list=17%, signal=23%
PEPTIDE_RECEPTOR_ACTIVITY	25	-0.21	-0.67	0.92	1	1	10082	tags=100%, list=79%, signal=475%
SERINE_TYPE_ENDOPEPTIDASE_INHIBITOR_ACTIVITY	12	-0.26	-0.67	0.88	1	1	280	tags=8%, list=2%, signal=9%
AMINE_TRANSMEMBRANE_TRANSPORTER_ACTIVITY	19	-0.21	-0.66	0.94	1	1	1627	tags=21%, list=13%, signal=24%
CYTOKINE_ACTIVITY	57	-0.18	-0.66	0.90	1	1	3257	tags=30%, list=26%, signal=40%
REGULATION_OF_PEPTIDYL_TYROSINE_PHOSPHORYLATION	14	-0.25	-0.66	0.86	1	1	3071	tags=50%, list=24%, signal=66%
PATTERN_BINDING	22	-0.21	-0.65	0.92	1	1	877	tags=14%, list=7%, signal=15%
CATION_TRANSMEMBRANE_TRANSPORTER_ACTIVITY	99	-0.15	-0.65	0.97	1	1	2454	tags=20%, list=19%, signal=25%
SECONDARY_METABOLIC_PROCESS	11	-0.25	-0.64	0.91	1	1	2196	tags=36%, list=17%, signal=44%
LYMPHOCYTE_ACTIVATION	29	-0.21	-0.64	0.88	1	1	3309	tags=38%, list=26%, signal=51%
POSITIVE_REGULATION_OF_IMMUNE_SYSTEM_PROCESS	31	-0.21	-0.64	0.91	1	1	2198	tags=26%, list=17%, signal=31%
CARBOHYDRATE_KINASE_ACTIVITY	10	-0.24	-0.64	0.93	1	1	2567	tags=40%, list=20%, signal=50%
CYTOCHROME_C_OXIDASE_ACTIVITY	11	-0.27	-0.63	0.87	1	1	3584	tags=45%, list=28%, signal=63%
LIPID_RAFT	15	-0.24	-0.63	0.89	1	1	67	tags=7%, list=1%, signal=7%
FATTY_ACID_METABOLIC_PROCESS	37	-0.19	-0.63	0.95	1	1	2898	tags=30%, list=23%, signal=38%
POSITIVE_REGULATION_OF_IMMUNE_RESPONSE	20	-0.21	-0.63	0.94	1	1	2198	tags=25%, list=17%, signal=30%
NEUROPEPTIDE_BINDING	13	-0.22	-0.62	0.93	1	1	8057	tags=92%, list=63%, signal=250%
ACTIVATION_OF_PROTEIN_KINASE_ACTIVITY	16	-0.21	-0.62	0.93	1	1	2575	tags=25%, list=20%, signal=31%
POSITIVE_REGULATION_OF_CYTOKINE_BIOSYNTHETIC_PROCESS	14	-0.25	-0.62	0.88	1	1	1948	tags=21%, list=15%, signal=25%
CELLULAR_DEFENSE_RESPONSE	33	-0.21	-0.61	0.90	1	1	9257	tags=97%, list=73%, signal=352%
REGULATION_OF_CELL_MIGRATION	13	-0.23	-0.61	0.94	1	1	3234	tags=38%, list=25%, signal=51%
PROTEIN_KINASE_BINDING	33	-0.17	-0.61	0.97	1	1	250	tags=6%, list=2%, signal=6%
NEURITE_DEVELOPMENT	14	-0.20	-0.61	0.96	1	1	2993	tags=36%, list=23%, signal=47%
REGULATION_OF_CELL_GROWTH	29	-0.18	-0.60	0.95	1	1	3483	tags=34%, list=27%, signal=47%
NEUROPEPTIDE_RECEPTOR_ACTIVITY	12	-0.21	-0.60	0.94	1	1	10082	tags=100%, list=79%, signal=475%
DEFENSE_RESPONSE	148	-0.17	-0.59	0.92	1	1	2200	tags=18%, list=17%, signal=21%
IMMUNE_EFFECTOR_PROCESS	30	-0.20	-0.59	0.93	1	1	2403	tags=23%, list=19%, signal=29%
CYTOKINE_BINDING	25	-0.22	-0.58	0.90	1	1	3339	tags=44%, list=26%, signal=59%
CDC42_PROTEIN_SIGNAL_TRANSDUCTION	10	-0.23	-0.58	0.94	1	1	2403	tags=20%, list=19%, signal=25%
LIPID_TRANSPORT	19	-0.20	-0.57	0.95	1	1	3242	tags=42%, list=25%, signal=56%
HETEROCYCLE_METABOLIC_PROCESS	12	-0.21	-0.57	0.97	1	1	2285	tags=25%, list=18%, signal=30%
NITROGEN_COMPOUND_CATABOLIC_PROCESS	11	-0.23	-0.56	0.94	1	1	403	tags=9%, list=3%, signal=9%
BEHAVIOR	67	-0.15	-0.55	1.00	1	1	1192	tags=10%, list=9%, signal=11%
MITOCHONDRIAL_MEMBRANE_PART	42	-0.19	-0.55	0.94	1	1	559	tags=10%, list=4%, signal=10%
CYTOKINE_BIOSYNTHETIC_PROCESS	21	-0.19	-0.53	0.97	1	1	1996	tags=19%, list=16%, signal=23%
HEMOSTASIS	26	-0.17	-0.53	0.98	1	1	3228	tags=35%, list=25%, signal=46%
SOLUTE_SODIUM_SYMPORTER_ACTIVITY	10	-0.20	-0.52	0.98	1	1	472	tags=10%, list=4%, signal=10%
CYTOKINE_METABOLIC_PROCESS	22	-0.18	-0.52	0.98	1	1	1996	tags=18%, list=16%, signal=22%
REGULATION_OF_IMMUNE_SYSTEM_PROCESS	38	-0.16	-0.52	0.98	1	1	2198	tags=21%, list=17%, signal=25%
MONOVALENT_INORGANIC_CATION_HOMEOSTASIS	10	-0.22	-0.52	0.97	1	1	600	tags=10%, list=5%, signal=10%
REGULATION_OF_PH	10	-0.22	-0.52	0.97	1	1	600	tags=10%, list=5%, signal=10%
ADAPTIVE_IMMUNE_RESPONSE_GO_0002460	19	-0.21	-0.52	0.92	1	1	8515	tags=95%, list=67%, signal=284%
ADAPTIVE_IMMUNE_RESPONSE	19	-0.21	-0.52	0.92	1	1	8515	tags=95%, list=67%, signal=284%
BLOOD_COAGULATION	25	-0.18	-0.52	0.98	1	1	3228	tags=36%, list=25%, signal=48%
COAGULATION	25	-0.18	-0.52	0.98	1	1	3228	tags=36%, list=25%, signal=48%
SYMPORTER_ACTIVITY	21	-0.17	-0.52	0.98	1	1	2733	tags=29%, list=21%, signal=36%
HUMORAL_IMMUNE_RESPONSE	15	-0.19	-0.52	0.97	1	1	3341	tags=40%, list=26%, signal=54%
NEURON_DIFFERENTIATION	21	-0.15	-0.51	1.00	1	1	2993	tags=33%, list=23%, signal=43%
RECEPTOR_COMPLEX	33	-0.18	-0.51	0.97	1	1	2217	tags=18%, list=17%, signal=22%
IMMUNE_RESPONSE	130	-0.15	-0.51	0.98	1	1	3541	tags=32%, list=28%, signal=43%
NEURON_DEVELOPMENT	18	-0.16	-0.51	0.99	1	1	2993	tags=33%, list=23%, signal=43%
CYTOKINE_PRODUCTION	42	-0.16	-0.50	0.99	1	1	917	tags=10%, list=7%, signal=10%
SYNAPSE_ORGANIZATION_AND_BIOGENESIS	11	-0.21	-0.49	0.99	1	1	2552	tags=27%, list=20%, signal=34%
SULFURIC_ESTER_HYDROLASE_ACTIVITY	10	-0.19	-0.47	0.99	1	1	2724	tags=40%, list=21%, signal=51%
MONOOXYGENASE_ACTIVITY	17	-0.17	-0.46	0.98	1	1	2583	tags=24%, list=20%, signal=29%
WOUND_HEALING	29	-0.14	-0.45	0.99	1	1	3228	tags=31%, list=25%, signal=41%
ACTIVATION_OF_IMMUNE_RESPONSE	13	-0.17	-0.45	0.99	1	1	2198	tags=23%, list=17%, signal=28%
PRODUCTION_OF_MOLECULAR_MEDIATOR_OF_IMMUNE_RESPONSE	12	-0.18	-0.44	0.99	1	1	10510	tags=100%, list=82%, signal=565%
LATE_ENDOSOME	11	-0.17	-0.42	0.99	1	1	10638	tags=100%, list=83%, signal=599%
MESODERM_DEVELOPMENT	13	-0.17	-0.42	0.99	1	1	2368	tags=23%, list=19%, signal=28%
RECEPTOR_SIGNALING_PROTEIN_SERINE_THREONINE_KINASE_ACTIVITY	15	-0.16	-0.42	1.00	0.99786013	1	4435	tags=60%, list=35%, signal=92%
