# Supplementary material for: Integrated Genomic and Epigenomic Analysis of Breast Cancer Brain Metastasis
Source: PLoS One. 2014 Jan 29;9(1):e85448. doi: 10.1371/journal.pone.0085448 (PMC3906004; doi:10.1371/journal.pone.0085448)
Supplement: File S1 — Supporting figures and tables. Figure S1: Combined Network for Upstream Analysis of FOXM1 and TBX2. The downstream genes connected to FOXM1 and TBX2 were illustrated as a network in IPA. The mRNA expression ratios are listed below the gene nodes. The legend within figure describes the node and edge color keys. Figure S2: Word Cloud Analysis of Cluster Enrichments. We have used word clouds to visually summarize the textual results from the enrichment analysis of each gene cluster as observed in Figure 3. The results were generated using www.wordle.net web resource. The larger the word, the more times it is mentioned in the enrichment categories. Supplementary Tables in File S1. Table S1a. Table S1b. Table S2. Table S3a. Table S3b. Table S4a. Figure S1. Table S4b. Table S5a–b. Table S6a–b. Table S7. Table S8a–f. Table S9a–f. Figure S2. Table S10. Table S11a–c. Table S11d. Table S12. Table S13. Table S14. (ZIP) [file pone.0085448.s001.zip › Supplementary Table S6a.pdf]

Supplementary Table 6a. Differentially Expressed Genes between Luminal B and basal-like tumors

Gene Symbol	GenBank Accession	fold change	ProbeName
**Upregulated Genes**			
ABCC11	NM_033151	8.89	A_23_P141076
ABCG1	NM_207627	3.50	A_23_P166297
ACADSB	NM_001609	3.40	A_23_P158570
ACADSB	NM_001609	6.35	A_32_P31945
ACSM1	NM_052956	5.68	A_23_P106933
ADSSL1	NM_199165	3.24	A_23_P76823
AFF3	NM_002285	7.66	A_23_P373464
AFF4	NM_014423	2.17	A_24_P394408
AGGF1	NM_018046	2.08	A_23_P250554
AGR2	NM_006408	362.70	A_23_P31407
AGR3	NM_176813	203.99	A_23_P42811
ANKRA2	NM_023039	2.51	A_24_P337397
ANKRA2	NM_023039	2.24	A_23_P41634
ANKRA2	NM_023039	2.39	A_23_P159012
ANKRD30A	NM_052997	41.56	A_23_P12533
ANKRD42	NM_182603	4.50	A_24_P357572
ANKRD42	NM_182603	4.23	A_32_P69166
ANXA9	NM_003568	13.49	A_23_P103617
APBB2	NM_004307	2.66	A_24_P234701
APBB2	NM_173075	2.24	A_23_P10701
APPL2	NM_018171	2.11	A_23_P105747
AR	NM_000044	36.48	A_23_P113111
ARFIP1	NM_001025595	1.54	A_24_P166094
ARFIP2	NM_012402	2.15	A_23_P139228
ARHGEF16	NM_014448	2.32	A_23_P114670
ARMC9	AB058771	2.42	A_24_P945408
ARRDC1	NM_152285	1.59	A_23_P391607
ATG16L1	NM_030803	2.53	A_32_P113508
ATP6AP1	NM_001183	2.13	A_23_P250462
ATP8B1	NM_005603	3.18	A_23_P107597
BAG3	NM_004281	1.93	A_23_P47077
BBS1	NM_024649	2.34	A_24_P184305
BBS4	NM_033028	2.38	A_23_P99967

BCAS4	NM_001010974	4.81	A_24_P143492
BCL2	NM_000633	3.46	A_23_P352266
C11orf52	NM_080659	1.89	A_23_P1722
C12orf72	NM_173802	2.27	A_23_P404120
C14orf25	BC038110	18.00	A_24_P393596
C14orf45	NM_025057	2.64	A_23_P76983
C14orf79	NM_174891	3.61	A_23_P412707
C14orf79	NM_174891	2.62	A_23_P420981
C14orf79	NM_174891	3.25	A_23_P376870
C16orf71	NM_139170	3.94	A_23_P414281
C17orf28	NM_030630	5.35	A_23_P118412
C19orf21	NM_173481	9.23	A_23_P390068
C19orf51	NM_178837	3.18	A_23_P335486
C1QTNF3	NM_181435	4.72	A_23_P122067
C1QTNF3	NM_181435	3.71	A_23_P122068
C20orf112	AK097804	3.57	A_23_P303548
C20orf114	NM_033197	9.34	A_23_P154784
C20orf26	NM_015585	8.41	A_32_P4262
C2orf55	NM_207362	2.93	A_24_P272313
C2orf81	NM_001145054	2.51	A_23_P131449
C3orf62	NM_198562	2.34	A_32_P831725
C4orf34	NM_174921	4.03	A_23_P112634
C5orf44	NM_024941	2.21	A_23_P121825
C6orf1	NM_178508	2.21	A_23_P411379
C6orf1	NM_178508	2.49	A_23_P81993
C6orf97	NM_025059	11.71	A_23_P93514
C9orf116	NM_001048265	2.43	A_23_P422115
C9orf75	NM_173691	2.43	A_23_P323836
C9orf98	NM_152572	3.29	A_23_P83200
CA12	NM_001218	24.98	A_24_P330518
CA12	AK000158	8.08	A_23_P163336
CA12	AK000158	5.36	A_23_P163338
CA12	NM_001218	20.15	A_23_P372234
CACNA1D	NM_000720	5.95	A_23_P365767
CACNA2D2	NM_001005505	9.87	A_23_P346900
CADPS2	NM_017954	3.95	A_24_P246710
CALCOCO2	NM_005831	2.39	A_23_P4223
CAPN13	NM_144575	16.56	A_23_P101972
CAPN13	NM_144575	7.83	A_24_P336983
CAPN13	AK074418	5.94	A_23_P345068
CAPN8	NM_001143962	9.25	A_23_P83381
CASC4	NM_138423	2.41	A_23_P140469
CCDC111	NM_152683	1.91	A_23_P358470

CCDC125	NM_176816	3.33	A_23_P432591
CCDC159	NM_001080503	2.26	A_24_P75920
CCDC24	NM_152499	2.36	A_24_P310864
CCDC30	AY639646	2.83	A_24_P28430
CCDC48	NM_024768	8.15	A_23_P166566
CCDC74B	NM_207310	4.19	A_23_P401718
CCDC74B	NM_207310	3.98	A_23_P381102
CCDC87	NM_018219	4.75	A_23_P127484
CCDC96	NM_153376	3.29	A_32_P69930
CCND1	NM_053056	5.08	A_24_P124550
CCND1	NM_053056	8.05	A_23_P202837
CCND1	NM_053056	4.54	A_24_P193011
CDK17	NM_002595	2.71	A_23_P33376
CDYL2	NM_152342	5.05	A_23_P371865
CEACAM6	BC005008	62.81	A_23_P421483
CEACAM7	NM_006890	15.74	A_24_P228302
CES8	NM_173815	4.81	A_23_P361544
CHRD	NM_003741	4.77	A_23_P502047
CHST15	NM_015892	3.89	A_23_P383986
CIRBP	NR_023312	2.40	A_23_P312652
CIRBP	NR_023312	2.92	A_23_P413456
CIRBP	NM_001280	2.99	A_23_P142322
CIRBP	NR_023312	3.42	A_23_P377616
CMBL	NM_138809	6.66	A_23_P144668
COG3	NM_031431	1.55	A_24_P296070
COL24A1	NM_152890	4.33	A_23_P74701
COL4A3BP	NM_001130105	2.40	A_24_P29277
COL4A3BP	AF136450	2.06	A_23_P61748
COMMD10	NM_016144	2.12	A_23_P252403
CPEB2	NM_182485	4.36	A_32_P225355
CPEB2	NM_182485	2.99	A_24_P340390
CPEB3	NM_014912	2.41	A_23_P46813
CST3	NM_000099	2.62	A_24_P216294
CST5	NM_001900	2.79	A_23_P170453
CTAGE1	NM_172241	1.85	A_24_P305223
CTSO	NM_001334	3.17	A_23_P110175
CXXC5	NM_016463	3.40	A_24_P930062
CXXC5	NM_016463	3.91	A_23_P399001
CYB5R1	NM_016243	2.31	A_23_P52101
CYP3A7	NM_000765	5.74	A_23_P358917
CYP4F8	NM_007253	4.89	A_23_P131060
DACH1	NM_080759	69.83	A_23_P32577
DAK	NM_015533	2.30	A_23_P36129

DALRD3	NM_018114	2.46	A_23_P135611
DCLK1	NM_004734	4.93	A_24_P928627
DCLK1	NM_004734	7.40	A_32_P108889
DCLK1	NM_004734	7.42	A_23_P369994
DNAH7	NM_018897	5.76	A_23_P33583
DNAJC12	NM_021800	8.50	A_23_P127220
DNAJC30	NM_032317	1.49	A_23_P157170
DNALI1	NM_003462	8.47	A_23_P160377
DPP7	NM_013379	2.24	A_23_P32975
DYNLRB2	NM_130897	18.64	A_23_P94840
EFCAB6	NM_022785	4.82	A_23_P68978
EHMT1	NM_024757	2.20	A_23_P20894
ELF1	NM_172373	2.41	A_23_P2801
ELF1	NM_172373	3.05	A_24_P78590
ELMOD2	NM_153702	2.00	A_23_P305692
EME2	AK074080	3.12	A_23_P366125
EPB41L5	NM_020909	2.48	A_24_P944640
EPB41L5	BC032822	2.31	A_23_P209298
EPOR	NM_000121	2.25	A_23_P381954
EPOR	NM_000121	2.27	A_23_P367899
EPS8L1	NM_133180	2.73	A_23_P208779
ERBB3	NM_001982	2.42	A_23_P349416
ERGIC1	NM_001031711	4.53	A_23_P333218
ERGIC1	NM_001031711	4.42	A_24_P89257
ERGIC1	NM_001031711	4.13	A_24_P97770
ERGIC1	NM_001031711	3.94	A_23_P404871
ESR1	NM_000125	34.21	A_23_P309739
EVL	NM_016337	3.44	A_23_P129038
EVL	NM_016337	3.96	A_23_P140427
EXD3	NM_017820	2.88	A_24_P49183
EXOC6	NM_019053	2.41	A_23_P169576
FAM120AOS	AK093641	2.50	A_32_P21646
FAM134B	NM_001034850	6.09	A_24_P266048
FAM134B	NM_001034850	4.99	A_23_P167599
FAM174A	NM_198507	2.64	A_23_P30283
FAM174B	NM_207446	4.81	A_23_P100001
FAM176B	NM_018166	3.03	A_23_P62831
FAM179B	NM_015091	3.00	A_23_P3102
FAM47E	NM_001136570	6.12	A_32_P83811
FAM5B	NM_021165	9.76	A_23_P35277
FAM84B	NM_174911	2.44	A_24_P329487
FBP1	NM_000507	9.39	A_23_P257111
FBXL5	NM_033535	2.21	A_23_P213247

FECH	NM_001012515	2.75	A_32_P151933
FFAR2	NM_005306	3.19	A_23_P397391
FGD3	NM_033086	3.40	A_24_P153840
FLJ30901	AK056490	4.28	A_23_P343104
FLJ38379	AK095698	7.81	A_24_P194661
FLJ40194	AK097513	3.22	A_24_P418152
FLJ40504	NR_028334	3.93	A_23_P373708
FLT3	NM_004119	9.79	A_23_P99442
FOXA1	NM_004496	72.76	A_23_P37127
FOXA1	NM_004496	72.07	A_24_P347431
FOXP1	NM_032682	3.67	A_24_P362737
FRMD6	NM_001042481	2.29	A_24_P330303
FSIP1	NM_152597	33.26	A_23_P353125
FSTL4	NM_015082	5.10	A_24_P266131
FUCA1	NM_000147	2.15	A_23_P11543
FUT8	NM_178154	5.49	A_23_P313632
FYCO1	NM_024513	2.12	A_23_P212339
GALNT10	NM_198321	3.78	A_23_P7706
GALNT10	AK021777	4.17	A_23_P19102
GALNT10	NM_198321	4.64	A_24_P910923
GALNT6	NM_007210	8.32	A_23_P204133
GAMT	NM_000156	3.53	A_23_P108143
GAMT	NM_138924	2.19	A_24_P19228
GATA2	NM_032638	3.70	A_24_P165998
GATA2	NM_032638	5.35	A_23_P110022
GATA3	NM_001002295	10.36	A_23_P75056
GDF15	NM_004864	13.06	A_23_P16523
GLI3	NM_000168	2.92	A_23_P111531
GLOD5	NM_001080489	5.55	A_23_P95619
GNA14	NM_004297	9.58	A_23_P169479
GP2	NM_001007240	17.73	A_24_P190007
GPC1	NM_002081	2.12	A_23_P209904
GPD1L	NM_015141	2.75	A_23_P318284
GPR160	NM_014373	6.92	A_23_P167005
GPR68	NM_003485	6.46	A_24_P931443
GPRC5C	AK000249	3.34	A_23_P346670
GPRC5C	NM_022036	4.05	A_23_P38167
GPRC5C	AK000249	5.74	A_23_P346673
GPRC5C	NM_022036	4.81	A_32_P109029
GRPEL1	AF070525	2.44	A_24_P166045
GSR	BC035691	3.43	A_32_P31618
GUSBP1	NR_027028	2.57	A_24_P84822
HAGHL	NM_032304	2.06	A_24_P356373

HNMT	NM_006895	2.34	A_23_P56734
HOXB3	NM_002146	8.81	A_24_P399220
HPX	NM_000613	8.25	A_23_P161998
HSPA1L	NM_005527	3.04	A_23_P70547
HSPB1	NM_001540	3.03	A_32_P76247
HSPB1	NM_001540	2.58	A_23_P257704
HSPB1	NM_001540	2.64	A_24_P86537
HTR7	NM_019859	3.38	A_23_P500381
HTR7P	NR_002774	3.51	A_32_P174572
IER3	NM_003897	3.47	A_23_P42257
IFT88	NM_175605	2.09	A_23_P48339
IL13RA1	NM_001560	2.19	A_24_P280113
IL6ST	CR621148	2.81	A_32_P140656
INPP4B	BC005273	7.36	A_24_P915492
IQCD	NM_138451	3.15	A_24_P390060
ITGB5	NM_002213	3.02	A_23_P166633
ITPR1	NM_002222	3.18	A_23_P92042
KAZALD1	AK172864	3.65	A_24_P192727
KBTBD4	NM_016506	2.16	A_23_P12950
KCNJ11	NM_000525	2.97	A_23_P1973
KIAA0232	NM_014743	2.12	A_23_P327069
KIAA0556	NM_015202	2.12	A_23_P381203
KIAA0564	NM_015058	2.08	A_23_P432077
KIAA1244	NM_020340	6.67	A_32_P188186
KIAA1370	NM_019600	3.66	A_23_P99853
KIAA1370	NM_019600	3.77	A_24_P357576
KIAA1407	NM_020817	2.66	A_23_P419213
KIAA1683	NM_025249	2.51	A_23_P130974
KIF12	NM_138424	8.41	A_23_P386356
KIF16B	NM_024704	2.67	A_23_P17503
KIF9	NM_022342	4.30	A_24_P225878
KITLG	NM_000899	6.75	A_23_P204654
KLHDC1	NM_172193	2.19	A_23_P422766
KLHDC9	NM_001007255	3.50	A_23_P86100
KRR1	NM_007043	2.25	A_32_P326819
KRT18	NM_000224	3.76	A_23_P99320
KRT18	NM_000224	3.30	A_24_P42136
KRT18	L32537	2.94	A_24_P924957
KRT18	NM_000224	3.72	A_32_P151544
LASP1	NM_006148	2.49	A_23_P89187
LASS2	NM_181746	2.03	A_23_P63010
LASS6	NM_203463	2.82	A_24_P289366
LCA5L	NM_152505	3.89	A_32_P48466

LCMT2	NM_014793	2.24	A_23_P106505
LEO1	NM_138792	2.28	A_23_P314222
LFNG	NM_001040167	11.91	A_23_P8452
LIMA1	NM_016357	3.31	A_23_P151267
LOC100129034	NR_027406	2.49	A_32_P91042
LOC100133050	NR_027503	2.54	A_32_P39003
LOC145837	NR_026979	34.10	A_32_P46594
LOC149134	AK022825	4.14	A_24_P803885
LOC220429	NR_003268	1.87	A_24_P358054
LOC254057	AK024653	4.99	A_24_P450092
LOC375295	BC013438	24.56	A_23_P302787
LOC375295	BC013438	15.37	A_32_P16204
LOC440335	NR_029454	5.88	A_24_P229884
LOC442249	XR_019231	3.68	A_24_P256063
LOC645431	NR_024334	13.58	A_23_P327156
LOC645431	NR_024334	2.49	A_24_P109766
LOC646976	AK096082	4.26	A_24_P350546
LOC90246	NR_026954	2.81	A_24_P532180
LPPR2	NM_022737	2.62	A_23_P153461
LRBA	NM_006726	2.77	A_24_P360078
LRFN2	NM_020737	9.29	A_32_P82111
LRRC27	NM_030626	2.83	A_32_P186157
LRRC46	NM_033413	2.84	A_23_P152949
LRRC48	NM_031294	4.43	A_23_P255701
LRRC4C	NM_020929	7.11	A_23_P24457
LRRC6	NM_012472	3.93	A_23_P112004
MAGED2	NM_201222	3.74	A_23_P33894
MAGED2	NM_201222	3.36	A_24_P160263
MALAT1	NR_002819	2.66	A_24_P497244
MALAT1	NR_002819	2.57	A_24_P873659
MAN2B2	NM_015274	2.26	A_23_P250380
MAN2B2	NM_015274	2.42	A_23_P250379
MARVELD2	AK055094	2.54	A_23_P401675
MAST4	NM_198828	5.44	A_24_P84340
MAST4	NM_001164664	2.77	A_23_P110571
MBOAT7	NM_024298	2.92	A_23_P208516
MCCC2	NM_022132	2.55	A_23_P18887
MCF2L	AK022184	3.03	A_23_P99496
MED13L	NM_015335	2.85	A_24_P911508
MEGF9	NM_001080497	2.72	A_32_P129894
MEIS3	NM_001009813	2.51	A_24_P207503
MEIS3	NM_001009813	3.25	A_23_P78795
MLPH	NM_024101	19.80	A_23_P165778

MLPH	NM_024101	14.69	A_23_P165783
MLPH	NM_001042467	11.70	A_23_P154400
MMEL1	NM_033467	2.35	A_23_P138294
MOAP1	NM_022151	2.36	A_23_P205389
MSL1	BC039449	4.92	A_24_P332837
MSX2	NM_002449	14.94	A_24_P132006
MTHFR	NM_005957	1.86	A_23_P400078
N4BP2L2	NM_033111	2.59	A_23_P65262
NAIP	NM_004536	3.44	A_24_P72139
NAT1	NM_000662	14.71	A_23_P95594
NAT2	NM_000015	11.72	A_23_P31798
NAV1	NM_020443	3.02	A_24_P102880
NBEA	NM_015678	4.29	A_23_P65278
NDFIP1	NM_030571	2.02	A_23_P81247
NEK11	NM_024800	2.51	A_23_P211973
NEK11	NM_145910	1.89	A_23_P155301
NEK9	NM_033116	2.99	A_23_P3131
NME3	NM_002513	3.08	A_23_P152115
NOL3	NM_003946	2.39	A_23_P206371
NPC1L1	NM_013389	3.77	A_23_P20075
NPDC1	NM_015392	4.45	A_23_P146572
NR1D1	NM_021724	3.66	A_24_P250227
NR1D1	NM_021724	2.58	A_23_P420873
NR2E3	NM_014249	6.84	A_23_P205867
NUCB2	AK097398	4.37	A_24_P595460
NUCB2	NM_005013	3.24	A_23_P13364
NUDT16	NM_152395	1.82	A_23_P310560
NUDT4	NM_199040	2.40	A_24_P335263
NUDT4	NM_199040	2.26	A_24_P50753
NUDT4	NM_199040	2.25	A_32_P117723
NUDT4	NM_199040	2.92	A_24_P67946
NXPH3	NM_007225	2.67	A_24_P940086
OBFC1	NM_024928	2.45	A_24_P759674
OVGP1	NM_002557	2.05	A_23_P103756
P2RX4	NM_002560	2.99	A_23_P53623
P4HTM	NM_177938	3.03	A_23_P113317
P4HTM	NM_177938	2.43	A_23_P113311
PAAF1	NM_025155	1.91	A_23_P139339
PAFAH2	NM_000437	2.04	A_24_P71153
PARP9	NM_031458	2.35	A_23_P69383
PAX9	NM_006194	5.33	A_32_P70818
PBLD	NM_022129	2.66	A_23_P149998
PBLD	NM_022129	3.31	A_24_P112395

PCDH1	NM_032420	2.85	A_24_P234838
PCSK4	NM_017573	3.77	A_23_P16648
PGAP3	NM_033419	5.84	A_24_P275828
PGPEP1	NM_017712	1.73	A_23_P218531
PIP	NM_002652	46.69	A_23_P8702
PLCD4	NM_032726	6.61	A_23_P385105
PLEKHF2	NM_024613	4.41	A_23_P20275
PLIN5	NM_001013706	2.29	A_24_P272222
PNPLA4	NM_004650	8.67	A_24_P943815
POLD4	NM_021173	2.10	A_23_P360215
POLD4	NM_021173	2.41	A_23_P127367
POLK	NM_016218	2.27	A_23_P386450
POLK	NM_016218	2.38	A_24_P303160
POTEC	NM_001137671	17.04	A_23_P56855
PPP1R3C	NM_005398	6.50	A_23_P35414
PRDM6	NM_001136239	4.30	A_32_P222684
PRR13	NM_001005354	2.05	A_24_P349466
PRR15	NM_175887	30.93	A_32_P154911
PRR15	NM_175887	6.82	A_23_P431346
PRRT2	NM_145239	3.27	A_23_P66017
RAB27B	NM_004163	5.46	A_23_P107612
RABEP1	NM_004703	4.05	A_24_P945147
RABEP1	NM_004703	4.61	A_24_P399174
RALGPS2	NM_152663	5.11	A_24_P173746
RAP2C	NM_021183	1.85	A_23_P147826
RAPGEF3	NM_006105	2.94	A_23_P151307
RASL11B	NM_023940	3.49	A_23_P69738
RBM47	NM_019027	2.55	A_24_P226108
RBM47	NM_019027	3.68	A_23_P132910
REPS2	NM_004726	3.28	A_32_P100109
RET	NM_020630	8.40	A_24_P343695
RET	NM_020975	29.57	A_23_P202245
RGS11	AK294448	4.64	A_24_P325118
RGS11	NM_003834	5.04	A_23_P118122
RHBG	NM_020407	2.35	A_23_P51690
RHOC	NM_175744	2.17	A_23_P12514
RHOH	NM_004310	5.42	A_23_P58132
RNASEL	NM_021133	3.68	A_23_P390172
RND1	NM_014470	2.77	A_23_P53370
RNF103	NM_005667	2.19	A_23_P56709
RSPH1	NM_080860	5.29	A_23_P102950
RUNDC1	NM_173079	2.05	A_24_P395621
RXRA	AK090416	2.32	A_24_P930985

SAR1B	NM_001033503	2.64	A_24_P916845
SDSL	NM_138432	2.55	A_23_P53439
SEC16A	NM_014866	3.03	A_23_P251303
SELENBP1	NM_003944	4.03	A_23_P74619
SEPHS2	NM_012248	2.78	A_23_P146798
SERF2	NM_001018108	2.02	A_24_P313334
SETD1B	NM_015048	2.32	A_23_P319895
SFXN5	NM_144579	2.61	A_23_P108819
SFXN5	NM_144579	2.14	A_24_P63030
SH3BP4	NM_014521	3.08	A_23_P79259
SIDT1	NM_017699	24.04	A_23_P132515
SLC22A18	NM_183233	2.69	A_23_P139260
SLC22A5	NM_003060	2.51	A_24_P174755
SLC27A2	NM_003645	5.16	A_23_P140450
SLC2A10	NM_030777	6.09	A_24_P271323
SLC39A6	NM_012319	6.74	A_24_P156049
SLC39A6	NM_012319	6.39	A_23_P50167
SLC40A1	NM_014585	4.57	A_23_P102391
SLC44A4	NM_025257	48.36	A_23_P93349
SLC44A4	NM_025257	32.79	A_24_P684183
SLC4A8	NM_004858	3.72	A_23_P72912
SLC4A8	NM_004858	3.91	A_24_P75680
SLC7A8	NM_182728	3.30	A_23_P205489
SMPD3	NM_018667	8.97	A_23_P163567
SNAP29	NM_004782	2.19	A_24_P48862
SNX25	NM_031953	1.91	A_24_P303097
SNX9	NM_016224	2.27	A_24_P179044
SORD	NM_003104	3.03	A_32_P127153
SORD	NM_003104	3.08	A_32_P89691
SPAG17	NM_206996	4.33	A_23_P319783
SPATA20	NM_022827	3.24	A_23_P118633
SPDEF	NM_012391	11.34	A_23_P111194
SPEF2	NM_024867	4.93	A_32_P179396
SPG11	NM_025137	2.22	A_23_P65699
SPOP	NM_001007226	3.35	A_24_P270890
SPOP	NM_001007226	3.13	A_23_P107257
SPRED2	NM_181784	2.67	A_32_P225854
SSBP2		6.32	A_32_P107219
SSH3	NM_017857	2.67	A_24_P266734
SSH3	NM_017857	2.52	A_23_P150147
ST3GAL5	NM_003896	2.63	A_23_P136573
ST3GAL5	NM_003896	2.90	A_23_P311869
STARD10	NM_006645	4.81	A_23_P36345

STRN3	NM_014574	2.64	A_23_P392076
STRN3	NM_014574	2.81	A_23_P65410
SUOX	NM_000456	2.10	A_23_P150857
SYT9	NM_175733	3.96	A_24_P255471
SYTL2	NM_032943	5.41	A_24_P85085
TADA2B	NM_152293	3.10	A_24_P687582
TADA2B	NM_152293	1.93	A_23_P396541
TANC2	NM_025185	4.33	A_24_P942068
TANC2	AK021886	3.46	A_23_P402908
TBC1D9	NM_015130	28.37	A_23_P41487
TCEAL1	NM_001006640	3.39	A_23_P73801
TCEAL3	NM_001006933	2.48	A_23_P434442
TCEAL4	NM_024863	2.27	A_23_P259166
TCEAL6	NM_001006938	2.44	A_32_P192545
TCTN1	NM_024549	2.29	A_23_P76402
TCTN1	BC030993	3.02	A_24_P282274
TFF1	NM_003225	16.83	A_23_P68759
TFF1	NM_003225	44.98	A_24_P322771
TFF3	NM_003226	109.01	A_24_P245778
TFF3	NM_003226	20.99	A_24_P289208
TFF3	NM_003226	197.30	A_23_P393099
TFF3	NM_003226	148.76	A_23_P257296
THPO	NM_000460	2.37	A_23_P121459
THSD4	NM_024817	14.35	A_23_P148249
TLE3	NM_005078	2.06	A_23_P342934
TM7SF2	NM_003273	3.03	A_23_P116037
TMBIM6	NM_003217	2.51	A_24_P355876
TMC4	NM_144686	3.45	A_23_P330461
TMC5	NM_024780	18.21	A_23_P15101
TMEM121	NM_025268	2.55	A_23_P61987
TMEM141	NM_032928	1.86	A_23_P94591
TMEM192	NM_001100389	1.99	A_24_P20524
TMEM192	NM_001100389	2.03	A_23_P253677
TMEM45B	NM_138788	7.18	A_23_P1682
TMEM50B	NM_006134	2.45	A_24_P305623
TMEM62	NM_024956	2.92	A_23_P49041
TMEM86A	NM_153347	3.46	A_32_P66035
TMEM87B	NM_032824	2.18	A_23_P91076
TMEM87B	NM_032824	3.31	A_23_P303155
TP53I11	NM_001076787	2.84	A_24_P160969
TPCN1	NM_001143819	2.14	A_24_P244575
TPCN1	NM_001143819	2.43	A_23_P218086
TRAF3IP1	NM_015650	1.72	A_23_P5359

TSC22D1	NM_183422	2.11	A_23_P162739
TSC22D3	NM_004089	2.16	A_23_P217688
TSNAXIP1	NM_018430	3.58	A_23_P129425
TSPAN1	NM_005727	9.16	A_23_P160167
TTC12	NM_017868	1.94	A_24_P73075
TTC8	NM_144596	2.54	A_32_P169735
UBR3	NM_172070	1.75	A_23_P5405
UBXN10	BX648631	4.56	A_32_P205053
UEVLD	NM_001040697	2.39	A_23_P203445
ULK1	NM_003565	2.01	A_24_P73370
UPRT	NM_145052	2.25	A_23_P159865
VEZT	NM_017599	2.12	A_23_P204609
WDR52	NM_001164496	2.13	A_23_P110090
WFS1	NM_006005	2.27	A_23_P121499
WWP1	NM_007013	3.46	A_23_P146990
XBP1	NM_005080	10.42	A_23_P120845
XBP1	NM_001079539	9.92	A_24_P100228
YPEL2	NM_001005404	2.49	A_24_P787947
ZMAT1	NM_032441	3.48	A_24_P11100
ZMYND10	NM_015896	3.79	A_23_P29663
ZNF136	NM_003437	2.12	A_23_P147121
ZNF217	NM_006526	2.29	A_23_P210608
ZNF281	NM_012482	2.13	A_23_P311087
ZNF304	NM_020657	1.79	A_24_P228266
ZNF467	BC038972	3.69	A_23_P59470
ZNF597	NM_152457	4.69	A_23_P3753
ZSWIM5	NM_020883	2.80	A_23_P383118
**Downregulated Genes**			
ACSS2	NM_018677	-2.73	A_24_P156295
ADAMTS3	NM_014243	-8.58	A_24_P140405
ADAT2	NM_182503	-3.04	A_23_P134014
ADAT2	NM_182503	-2.31	A_24_P186204
ADORA2B	NM_000676	-4.83	A_23_P55477
AGBL5	NM_001035507	-2.60	A_23_P154466
AKR1E2	AB040820	-4.13	A_23_P1322
AMOTL1	NM_130847	-2.37	A_23_P138796
AMOTL1	NM_130847	-2.96	A_24_P329815
ANKRD36	NM_001164315	-2.90	A_24_P336931
ANKRD36BP1	NR_026844	-3.21	A_23_P97123
ANKS6	NM_173551	-3.22	A_23_P362183
ANP32E	NM_030920	-3.70	A_23_P160934
ANP32E	NM_030920	-4.02	A_24_P225468

ANXA8L2	BC008813	-11.59	A_23_P395054
ANXA8L2	NM_001630	-19.18	A_32_P105549
ARHGAP11A	NM_014783	-2.50	A_23_P136805
ART3	NM_001179	-23.92	A_23_P80918
ASPM	NM_018136	-2.62	A_23_P52017
ASPM	NM_018136	-2.75	A_24_P911179
ASS1	NM_000050	-3.28	A_23_P31921
ATL2	NM_022374	-2.52	A_23_P209619
ATP11C	NM_173694	-2.34	A_24_P399942
ATP11C	NM_001010986	-2.86	A_23_P251730
ATP6V1C2	NM_001039362	-14.52	A_23_P250914
AURKB	NM_004217	-2.32	A_23_P130182
B3GNT5	NM_032047	-9.47	A_23_P18372
BCL11A	NM_022893	-10.41	A_23_P218584
BCL11A	NM_018014	-19.53	A_24_P402588
BCL11A	NM_022893	-37.96	A_24_P411186
BOP1	NM_015201	-2.75	A_23_P43800
BPI	NM_001725	-3.29	A_23_P131789
BTG3	BC028229	-4.90	A_32_P33723
BTG3	NM_006806	-5.50	A_23_P80068
BYSL	NM_004053	-2.44	A_23_P145197
C11orf75	NM_020179	-2.53	A_23_P75430
C13orf38	NM_001144982	-5.56	A_23_P14216
C15orf23	NM_001142761	-2.30	A_23_P140705
C15orf41	NM_032499	-2.44	A_23_P77201
C15orf42	NM_152259	-2.74	A_23_P345707
C15orf42	NM_152259	-2.48	A_32_P109296
C1orf163	NM_023077	-2.21	A_23_P347508
C1orf198	NM_032800	-2.72	A_32_P42574
C21orf91	NM_017447	-3.26	A_23_P211015
C22orf23	AK097339	-4.23	A_24_P941407
C2orf3	EF158467	-2.09	A_24_P51037
C3orf26	NM_032359	-2.28	A_23_P132874
C4orf7	NM_152997	-14.82	A_23_P362694
C5orf23	NM_024563	-11.24	A_23_P58676
C5orf46	NM_206966	-10.35	A_23_P19176
C6orf15	NM_014070	-9.79	A_24_P72364
C6orf173	NM_001012507	-4.72	A_32_P143245
C6orf173	NM_001012507	-4.49	A_24_P462899
C6orf218	NR_027793	-5.74	A_23_P302595
C8orf85	NM_001025357	-6.71	A_32_P181527
C9orf40	NM_017998	-2.06	A_23_P43425
C9orf40	NM_017998	-4.29	A_24_P43876

CAPN6	NM_014289	-6.09	A_23_P217570
CASC5	NM_170589	-2.17	A_24_P378331
CCDC67	NM_181645	-6.92	A_32_P153954
CCNA2	NM_001237	-2.82	A_23_P58321
CCNB2	NM_004701	-2.18	A_23_P65757
CDC20	NM_001255	-2.60	A_23_P149200
CDC45L	NM_003504	-2.54	A_23_P57379
CDCA2	NM_152562	-3.67	A_23_P385861
CDCA3	NM_031299	-2.37	A_23_P162476
CDCA5	NM_080668	-2.38	A_23_P104651
CDCA7	NM_031942	-8.98	A_24_P171549
CDCA7	NM_031942	-7.75	A_23_P251421
CDCA7L	NM_018719	-2.90	A_24_P274795
CDCA8	NM_018101	-3.11	A_23_P375
CDH3	NM_001793	-5.35	A_23_P49155
CDK6	NM_001259	-7.02	A_24_P166663
CDKN2C	NM_078626	-2.13	A_23_P85460
CENPA	NM_001809	-2.81	A_24_P413884
CENPF	NM_016343	-2.49	A_23_P401
CENPN	NM_018455	-2.30	A_23_P88740
CERK	NM_022766	-3.42	A_23_P211659
CHAC2	NM_001008708	-3.29	A_32_P194264
CHEK1	NM_001274	-2.88	A_23_P116123
CHODL	NM_024944	-10.31	A_23_P68669
CHRM3		-4.34	A_23_P200843
CHST3	NM_004273	-2.77	A_24_P269779
CIRH1A	NM_032830	-1.81	A_24_P25346
CKS1B	NM_001826	-2.55	A_32_P192430
CKS1B	NM_001826	-2.04	A_32_P206698
CKS1B	NM_001826	-2.39	A_23_P45917
CKS2	NM_001827	-2.29	A_23_P71727
CLDN8	NM_199328	-14.35	A_23_P427014
CLIC4	NM_013943	-1.97	A_23_P259189
CLIP4	NM_024692	-3.68	A_23_P209232
CNGA1	NM_000087	-5.22	A_24_P256722
CNTNAP3	NM_033655	-7.61	A_23_P9135
CRABP1	NM_004378	-3.51	A_23_P117882
CREB3L2	BC063666	-2.75	A_24_P180654
CSDA	NM_003651	-2.90	A_23_P25224
CSDA	NM_003651	-2.93	A_24_P625382
CTPS	NM_001905	-2.58	A_23_P21706
CTSL2	NM_001333	-4.03	A_23_P146456
CYB5R2	NM_016229	-3.08	A_23_P2181

CYP39A1	NM_016593	-6.88	A_23_P133712
DEK	NM_003472	-2.61	A_23_P254702
DIAPH3	NM_030932	-2.68	A_23_P419254
DIAPH3	NM_001042517	-2.88	A_32_P150891
DLGAP5	NM_014750	-1.82	A_23_P88331
DMD	NM_004019	-3.00	A_24_P342388
DNAH14	NM_144989	-2.69	A_23_P333951
DNAH14	NM_001145154	-2.66	A_32_P87531
DSC2	NM_024422	-6.60	A_23_P4494
DSC3	NM_024423	-33.48	A_24_P344416
DSC3	NM_024423	-9.92	A_23_P208029
E2F2	NM_004091	-1.88	A_23_P408955
ECE2	NM_032331	-2.58	A_23_P92261
EIF2C2	NM_012154	-2.15	A_23_P112159
EIF5A2	NM_020390	-2.86	A_24_P385739
ELF5	NM_198381	-14.74	A_24_P227141
ELF5	NM_198381	-13.58	A_23_P13465
EN1	NM_001426	-46.95	A_23_P56404
ETV6	NM_001987	-2.15	A_23_P105264
EXPH5	NM_015065	-2.44	A_24_P937691
EXPH5	NM_015065	-2.06	A_23_P403335
FAF1	NM_007051	-1.62	A_23_P96853
FAM123B	NM_152424	-2.35	A_23_P308150
FAM126A	NM_032581	-5.09	A_23_P8582
FAM171A1	NM_001010924	-4.67	A_23_P44964
FAM27E3	BC119675	-3.23	A_23_P348979
FAM36A	NM_198076	-2.13	A_32_P106646
FAM64A	NM_019013	-2.92	A_23_P49878
FAM83B	NM_001010872	-4.24	A_24_P66780
FANCE	NM_021922	-2.75	A_23_P42335
FANCI	NM_018193	-2.56	A_24_P902509
FBL	NM_001436	-2.38	A_23_P78892
FBL	NM_001436	-2.15	A_23_P78888
FBXO31	AK026130	-2.13	A_23_P89030
FGFBP1	NM_005130	-35.10	A_23_P30126
FKBP1A	NM_054014	-3.24	A_23_P397238
FLJ25694	AK127969	-2.70	A_23_P342751
FLJ40330	NR_015424	-2.33	A_24_P341089
FNDC3B	BC012204	-2.97	A_24_P919304
FOXC1	NM_001453	-7.16	A_23_P390504
FOXC1	NM_001453	-6.06	A_32_P205110
FOXL1	NM_005250	-2.74	A_23_P365081
FOXM1	NM_202002	-3.35	A_23_P151150

FSCN1	NM_003088	-2.04	A_23_P168531
FZD7	NM_003507	-6.18	A_23_P209449
FZD9	NM_003508	-2.14	A_23_P59613
GABRP	NM_014211	-19.60	A_23_P328545
GCNT2	NM_001491	-5.19	A_24_P397489
GCSH	NM_004483	-2.28	A_23_P117933
GEMIN4	NM_015721	-2.08	A_23_P66872
GEMIN4	NM_015721	-2.18	A_23_P66867
GMPS	NM_003875	-2.23	A_23_P21033
GNB4	NM_021629	-2.61	A_32_P184916
GPR161	NM_153832	-2.69	A_23_P354314
GPRIN2	AB011086	-7.19	A_23_P343382
GPSM2	NM_013296	-2.20	A_23_P63402
GSDMC	NM_031415	-6.83	A_23_P60120
GSG2	AK056691	-2.56	A_24_P76521
GTPBP4	NM_012341	-2.20	A_23_P12874
HEATR1	NM_018072	-2.10	A_23_P103628
HIF3A	NM_022462	-8.51	A_23_P374339
HIST1H1A	NM_005325	-23.79	A_23_P70448
HLA-DOB	NM_002120	-3.57	A_23_P30736
HORMAD1	NM_032132	-53.00	A_32_P199884
HPDL	NM_032756	-4.38	A_23_P74449
HSPC159	NM_014181	-3.16	A_23_P430818
IGF2BP3	NM_006547	-8.85	A_23_P19987
IL12RB2	NM_001559	-9.34	A_23_P72077
IL17RD	NM_017563	-5.53	A_32_P188860
ILF2	NM_004515	-2.26	A_23_P257956
ITGB8	NM_002214	-5.89	A_24_P759477
ITGB8	NM_002214	-5.54	A_24_P273599
ITGB8	NM_002214	-3.95	A_23_P123060
JRKL	NM_003772	-3.24	A_23_P202737
KHDC1	NM_030568	-2.46	A_24_P280762
KIF18B	BC048263	-2.59	A_24_P680947
KIF1B	NM_183416	-3.67	A_24_P649624
KIF1B	NM_183416	-2.98	A_24_P145066
KIF1C	BC040307	-2.03	A_32_P149288
KIF20A	NM_005733	-1.80	A_23_P256956
KIF2C	NM_006845	-2.42	A_23_P34788
KIFC1	NM_002263	-2.45	A_23_P133956
KIT	NM_000222	-18.57	A_23_P110253
KLHL18	BC015962	-3.23	A_24_P916686
KRT14	NM_000526	-10.80	A_24_P265346
KRT14	NM_000526	-2.68	A_23_P4335

KRT16	NM_005557	-22.48	A_23_P38537
KRT16	NM_005557	-23.50	A_24_P392991
KRT16P2	NR_029392	-12.95	A_32_P62963
KRT16P3	NR_029393	-8.29	A_32_P168973
KRT17	NM_000422	-12.58	A_23_P96158
KRT34	NM_021013	-2.84	A_23_P101054
KRT5	NM_000424	-14.70	A_23_P218047
KRT6A	NM_005554	-7.06	A_23_P87653
KRT6B	NM_005555	-43.56	A_23_P76249
KRT6C	NM_173086	-8.62	A_23_P366936
KRTAP6-3	NM_181605	-1.87	A_24_P391604
L3MBTL4	NM_173464	-13.41	A_23_P326474
LAD1	NM_005558	-3.57	A_23_P415510
LBR	NM_002296	-3.17	A_23_P200493
LEMD1	NM_001001552	-49.19	A_24_P696761
LGALS7	NM_002307	-5.87	A_24_P238250
LGALS7B	NM_001042507	-11.59	A_23_P108062
LGALS7B	NM_001042507	-5.50	A_24_P348118
LIN9	NM_173083	-2.00	A_23_P301995
LIN9	NM_173083	-2.12	A_32_P233304
LMNB2	NM_032737	-2.00	A_23_P67725
LMO4	NM_006769	-2.97	A_23_P380181
LOC100128355	XM_002343797	-2.15	A_24_P204474
LOC100293193	XR_079078	-3.09	A_24_P235520
LOC100293193	XR_079078	-4.31	A_32_P112623
LOC149351	BC036441	-4.01	A_24_P520767
LOC401317	BC087859	-3.07	A_32_P219135
LOC643650	BC033221	-4.83	A_23_P359214
LOC645195	AK123450	-2.23	A_32_P224234
LOC729088	CR602569	-2.28	A_32_P36143
LOC729683	CR594811	-1.85	A_32_P145010
LRP12	NM_013437	-3.35	A_23_P8906
LRP12	NM_013437	-3.71	A_24_P415012
LRRC42	NM_052940	-2.05	A_23_P51278
LY6K	NM_017527	-8.41	A_23_P397285
MAGOH	NM_002370	-3.03	A_23_P200216
MAP7D3	NM_024597	-1.86	A_24_P177631
MCM10	NM_182751	-2.80	A_23_P161474
MCM10	NM_182751	-2.96	A_24_P412088
MED30	NM_080651	-2.71	A_23_P31866
MIA	NM_006533	-25.66	A_23_P4714
MICALL1	NM_033386	-3.24	A_23_P68922
MLLT4	BC014505	-3.20	A_23_P344694

MPP6	NM_016447	-8.19	A_23_P71053
MPZL2	NM_144765	-5.86	A_23_P150379
MRPL14	NM_032111	-2.28	A_23_P124035
MRPL2	NM_015950	-2.55	A_23_P7941
MRPL37	NM_016491	-2.14	A_23_P135474
MSH2	NM_000251	-2.00	A_23_P102471
MTHFD1L	AY374131	-2.12	A_23_P214908
MTHFD1L	NM_015440	-2.40	A_23_P214907
MTSS1L	NM_138383	-2.07	A_32_P84084
MYO10	NM_012334	-2.32	A_24_P46357
MYO19	NM_001033580	-2.02	A_23_P100868
NANOGP1	AY455283	-3.87	A_24_P68068
NCAPD2	NM_014865	-2.33	A_23_P25293
NDC80	NM_006101	-2.60	A_23_P50108
NDC80	NM_006101	-3.17	A_24_P14156
NFE2L3	NM_004289	-5.58	A_24_P136653
NFE2L3	NM_004289	-4.87	A_23_P42718
NFIL3	NM_005384	-2.64	A_23_P32253
NMT2	NM_004808	-2.29	A_23_P138686
NPR3	NM_000908	-11.55	A_23_P327451
NPR3	NM_000908	-10.20	A_23_P253536
NRG2	NM_004883	-3.56	A_23_P349857
NRG2	NM_013982	-6.55	A_23_P213699
NRTN	NM_004558	-4.93	A_23_P90359
NT5DC2	NM_022908	-3.09	A_23_P44836
NTN1	NM_004822	-3.08	A_32_P53524
NUDCD1	NM_032869	-2.14	A_23_P123343
NUDT5	NM_014142	-2.26	A_23_P1199
OBSCN	NM_052843	-2.36	A_24_P119685
OGFRL1	NM_024576	-4.26	A_23_P7791
OIP5	NM_007280	-2.36	A_23_P379614
OLFM4	NM_006418	-33.04	A_24_P181254
OSBPL3	NM_015550	-5.28	A_24_P599340
OSBPL3	NM_015550	-5.55	A_24_P377499
OSBPL3	NM_015550	-3.55	A_23_P215525
OSR1	NM_145260	-4.03	A_23_P323272
PDE7A	NM_002603	-2.40	A_23_P123478
PELI1	NM_020651	-2.56	A_23_P120345
PGM1	NM_002633	-2.17	A_23_P52031
PHGDH	NM_006623	-4.50	A_23_P85783
PIM1	NM_002648	-2.73	A_23_P345118
PLEKHG4B	NM_052909	-7.49	A_32_P129269
PLEKHG4B	NM_052909	-3.18	A_23_P81640

PM20D2	NM_001010853	-9.13	A_32_P86118
POLH	NM_006502	-2.89	A_32_P69492
POLR2F	NM_021974	-2.24	A_23_P80321
POU4F1	NM_006237	-15.70	A_23_P205164
PPP1CB	NM_002709	-1.61	A_24_P396720
PPP2R3A	NM_002718	-2.93	A_23_P10401
PPP2R3A	NM_002718	-2.45	A_24_P388433
PPP2R5D	NM_180976	-1.90	A_24_P294931
PPPDE1	BC020640	-4.15	A_24_P922808
PPPDE1	NM_016076	-2.23	A_23_P201445
PRDM13	NM_021620	-20.82	A_23_P256581
PRICKLE1	NM_153026	-4.23	A_23_P408285
PROM1	NM_006017	-52.99	A_23_P258463
PRPF38A	NM_032864	-1.61	A_24_P97001
PSAT1	NM_058179	-3.56	A_23_P259692
PSMG1	NM_003720	-2.12	A_23_P68717
PTK7	NM_002821	-2.92	A_24_P320545
PTPLA	NM_014241	-4.25	A_23_P161352
PTPN14	NM_005401	-3.25	A_24_P2648
PTPN14	NM_005401	-5.17	A_23_P149111
PTTG1	NM_004219	-2.17	A_23_P7636
QKI	NM_006775	-3.17	A_24_P941322
RAD51AP1	NM_006479	-2.61	A_23_P99292
RAD54L	NM_003579	-2.45	A_23_P74115
RARRES1	NM_002888	-47.57	A_23_P18078
RASD2	NM_014310	-3.64	A_24_P357100
RDH10	NM_172037	-5.01	A_24_P261032
RDH10	NM_172037	-5.36	A_32_P25050
REXO2	NM_015523	-2.03	A_24_P316364
REXO2	NM_015523	-2.10	A_23_P150365
RGMA	NM_020211	-5.35	A_23_P372308
RIOK1	NM_153005	-2.14	A_24_P8088
ROPN1	NM_017578	-153.87	A_32_P184464
ROPN1B	NM_001012337	-120.96	A_24_P417407
RPS27A	NM_002954	-2.18	A_32_P24581
SAA1	NM_000331	-12.98	A_24_P335092
SAA2	NM_030754	-7.07	A_23_P306203
SEH1L	NM_031216	-2.34	A_24_P407930
SEH1L	NM_031216	-2.75	A_23_P78311
SEPHS1	BC064610	-1.81	A_24_P90022
SERBP1	NM_001018067	-2.08	A_23_P359111
SFRP1	NM_003012	-12.12	A_23_P10127
SFRP1	NM_003012	-22.68	A_23_P10121

SFRS13B	NM_080743	-13.09	A_23_P110903
SH2D2A	NM_003975	-2.78	A_23_P160618
SIN3B	BC025026	-3.33	A_24_P93901
SKA3	BC013418	-2.36	A_23_P340909
SKP2	NM_032637	-2.31	A_23_P156310
SLC19A3	NM_025243	-5.90	A_23_P39871
SLC25A37	AF495725	-3.81	A_24_P64100
SLC25A37	AF113696	-3.05	A_23_P216004
SLC27A6	NM_001017372	-10.99	A_23_P41789
SLC34A2	NM_006424	-13.84	A_23_P133036
SLC6A2	NM_001172504	-3.07	A_24_P910660
SLC6A2	NM_001043	-4.06	A_23_P358345
SLPI	NM_003064	-8.58	A_23_P91230
SLPI	NM_003064	-11.13	A_24_P190472
SOHLH2	NM_017826	-5.92	A_23_P25615
SOSTDC1	NM_015464	-52.70	A_23_P145841
SOX10	NM_006941	-3.16	A_23_P143694
SPC25	NM_020675	-2.11	A_23_P51085
SPEG	AK055387	-2.69	A_23_P338919
SPSB1	NM_025106	-2.17	A_24_P96961
SPSB1	NM_025106	-2.61	A_23_P200096
SRF	NM_003131	-2.00	A_24_P346277
SRPK1	NM_003137	-2.45	A_23_P19543
STAC	NM_003149	-14.12	A_23_P121061
STAC	NM_003149	-28.88	A_24_P234415
STMN1	NM_203401	-1.84	A_23_P200866
STRA8	NM_182489	-4.77	A_24_P203308
SUV39H2	NM_024670	-2.72	A_23_P202392
TAGAP	NM_138810	-3.48	A_23_P339588
TBPL1	NM_004865	-2.07	A_23_P168276
TBX19	NM_005149	-2.52	A_23_P137705
TCF7L1	NM_031283	-7.88	A_23_P142872
TEAD4	NM_003213	-2.43	A_23_P94795
TEX10	NM_017746	-2.12	A_23_P112412
TFCP2L1	NM_014553	-9.15	A_23_P5301
TIFA	NM_052864	-2.11	A_24_P350686
TMEM123	NM_052932	-2.45	A_23_P202964
TMEM123	NM_052932	-2.62	A_24_P309415
TMEM38A	NM_024074	-2.68	A_23_P101392
TMEM65	NM_194291	-2.84	A_24_P256583
TMEM74	NM_153015	-6.14	A_23_P390139
TMSB15B	NM_194324	-4.67	A_23_P96599
TNNI2	NM_003282	-3.25	A_23_P24784

TOP1MT	NM_052963	-2.78	A_24_P248053
TPX2	NM_012112	-2.21	A_23_P68610
TRDMT1	NM_004412	-4.27	A_24_P252705
TRDMT1	NM_004412	-4.60	A_23_P115636
TRIM29	NM_012101	-12.66	A_23_P203267
TSLP	NM_033035	-7.18	A_23_P121987
TTK	NM_003318	-3.37	A_23_P259586
TTLL4	NM_014640	-3.46	A_23_P142697
TYMS	NM_001071	-2.72	A_23_P50096
UBASH3B	NM_032873	-4.62	A_24_P192933
UBE2E3	NM_006357	-3.35	A_24_P148450
UBE2E3	NM_006357	-4.78	A_23_P11192
UCK2	NM_012474	-2.30	A_23_P487
UGT8	NM_003360	-11.08	A_24_P103264
UGT8	U62899	-11.20	A_23_P72747
UGT8	AL137342	-16.35	A_24_P942589
URB2	NM_014777	-2.34	A_23_P74914
USP1	NM_003368	-2.12	A_23_P11652
USP31	NM_020718	-1.91	A_24_P390583
USP6NL	NM_014688	-2.53	A_23_P138426
VGLL1	NM_016267	-13.75	A_23_P253123
VRK2	NM_006296	-2.17	A_23_P119992
WNT11	NM_004626	-3.91	A_24_P253003
WNT6	NM_006522	-12.42	A_23_P119916
WWTR1	NM_015472	-4.35	A_23_P29769
WWTR1	NM_015472	-6.42	A_32_P6868
XPO5	NM_020750	-2.36	A_23_P256855
YBX1	NM_004559	-2.03	A_24_P375002
YBX1	NM_004559	-2.22	A_23_P34767
YBX1	NM_004559	-2.23	A_32_P218989
YBX1	NM_004559	-2.03	A_24_P101391
ZCCHC11	NM_001009881	-2.91	A_23_P34433
ZNF232	NM_014519	-1.82	A_23_P4294
ZNF238	NM_006352	-3.16	A_24_P299663
ZNF238	NM_006352	-3.60	A_23_P200512
ZNF286A	AF086305	-4.78	A_24_P910833
ZNF318	NM_014345	-2.18	A_23_P145175
ZNF462	NM_021224	-4.67	A_23_P60499
ZNF507	NM_014910	-2.11	A_23_P208812
ZW10	NM_004724	-2.11	A_23_P64204
