# Supplementary material for: Integrated Genomic and Epigenomic Analysis of Breast Cancer Brain Metastasis
Source: PLoS One. 2014 Jan 29;9(1):e85448. doi: 10.1371/journal.pone.0085448 (PMC3906004; doi:10.1371/journal.pone.0085448)
Supplement: File S1 — Supporting figures and tables. Figure S1: Combined Network for Upstream Analysis of FOXM1 and TBX2. The downstream genes connected to FOXM1 and TBX2 were illustrated as a network in IPA. The mRNA expression ratios are listed below the gene nodes. The legend within figure describes the node and edge color keys. Figure S2: Word Cloud Analysis of Cluster Enrichments. We have used word clouds to visually summarize the textual results from the enrichment analysis of each gene cluster as observed in Figure 3. The results were generated using www.wordle.net web resource. The larger the word, the more times it is mentioned in the enrichment categories. Supplementary Tables in File S1. Table S1a. Table S1b. Table S2. Table S3a. Table S3b. Table S4a. Figure S1. Table S4b. Table S5a–b. Table S6a–b. Table S7. Table S8a–f. Table S9a–f. Figure S2. Table S10. Table S11a–c. Table S11d. Table S12. Table S13. Table S14. (ZIP) [file pone.0085448.s001.zip › Supplementary Table S6b.pdf]

Supplementary Table 6b. Differentially expressed genes between Basal-like and Her2+/ER- BBM tumors

Gene Symbol	GenBank Accession	fold change	ProbeName
**Upregulated Genes**			
ANP32E	NM_030920	4.24	A_23_P160934
ANP32E	NM_030920	5.03	A_24_P225468
ANXA8L2	BC008813	13.83	A_23_P395054
ANXA8L2	NM_001630	23.78	A_32_P105549
ARHGAP11A	NM_014783	3.00	A_23_P136805
ART3	NM_001179	21.77	A_23_P80918
ASPM	NM_018136	2.56	A_23_P52017
ASPM	NM_018136	2.68	A_24_P911179
B3GNT5	NM_032047	3.80	A_23_P18372
BCL11A	NM_022893	4.04	A_23_P218584
BCL11A	NM_018014	6.39	A_24_P402588
BCL11A	NM_022893	9.68	A_24_P411186
BPI	NM_001725	2.81	A_23_P131789
C1orf163	NM_023077	2.23	A_23_P347508
C1orf198	NM_032800	2.37	A_32_P42574
C21orf91	NM_017447	2.48	A_23_P211015
C22orf23	AK097339	2.61	A_24_P941407
C4orf7	NM_152997	9.47	A_23_P362694
C5orf23	NM_024563	18.93	A_23_P58676
C5orf46	NM_206966	19.79	A_23_P19176
C6orf15	NM_014070	8.28	A_24_P72364
C6orf173	NM_001012507	3.98	A_32_P143245
C6orf173	NM_001012507	3.87	A_24_P462899
C6orf218	NR_027793	4.80	A_23_P302595
C8orf85	NM_001025357	14.02	A_32_P181527
C9orf40	NM_017998	2.71	A_24_P43876
CAPN6	NM_014289	5.13	A_23_P217570
CCNA2	NM_001237	2.06	A_23_P58321
CCNB2	NM_004701	2.47	A_23_P65757
CDC20	NM_001255	2.72	A_23_P149200
CDCA2	NM_152562	3.30	A_23_P385861
CDCA7	NM_031942	5.46	A_24_P171549
CDCA7	NM_031942	4.28	A_23_P251421
CDCA8	NM_018101	2.72	A_23_P375

CDK6	NM_001259	7.34	A_24_P166663
CDKN2C	NM_078626	3.02	A_23_P85460
CENPA	NM_001809	2.12	A_24_P413884
CENPF	NM_016343	2.72	A_23_P401
CENPN	NM_018455	2.70	A_23_P88740
CHODL	NM_024944	4.03	A_23_P68669
CHRM3		10.31	A_23_P200843
CIRH1A	NM_032830	2.02	A_24_P25346
CKS1B	NM_001826	2.63	A_32_P192430
CKS1B	NM_001826	2.55	A_23_P45917
CKS2	NM_001827	2.51	A_23_P71727
CLIC4	NM_013943	2.71	A_23_P259189
CLIC4	NM_013943	2.60	A_23_P135494
CNTNAP3	NM_033655	4.70	A_23_P9135
COL27A1	AK021957	5.13	A_23_P158096
CRABP1	NM_004378	3.47	A_23_P117882
CTSL2	NM_001333	2.87	A_23_P146456
CYB5R2	NM_016229	4.73	A_23_P2181
CYP39A1	NM_016593	5.94	A_23_P133712
DCAF4	NM_181340	2.41	A_24_P241318
DCAF4	NM_181340	2.34	A_23_P370035
DEK	NM_003472	2.34	A_23_P254702
DIAPH3	NM_030932	2.79	A_23_P419254
DIAPH3	NM_001042517	3.25	A_32_P150891
DLGAP5	NM_014750	2.30	A_23_P88331
DNAH14	NM_144989	2.19	A_23_P333951
DNAH14	NM_001145154	2.32	A_32_P87531
DSC2	NM_024422	4.24	A_23_P4494
DSC3	NM_024423	27.91	A_24_P344416
DSC3	NM_024423	8.53	A_23_P208029
E2F2	NM_004091	2.10	A_23_P408955
E2F7	NM_203394	2.77	A_32_P210202
EIF5A2	NM_020390	2.25	A_24_P385739
ELF5	NM_198381	23.21	A_24_P227141
ELF5	NM_198381	24.74	A_23_P13465
EN1	NM_001426	57.45	A_23_P56404
FAF1	NM_007051	2.07	A_23_P96853
FAM171A1	NM_001010924	2.90	A_23_P44964
FAM64A	NM_019013	3.47	A_23_P49878
FAM83B	NM_001010872	6.36	A_24_P66780
FANCE	NM_021922	2.72	A_23_P42335
FANCI	NM_018193	2.09	A_24_P902509
FBL	NM_001436	2.13	A_23_P78892

FBXO31	AK026130	2.31	A_23_P89030
FGFBP1	NM_005130	8.19	A_23_P30126
FLJ22536	NR_015410	6.63	A_24_P838448
FLJ25694	AK127969	2.77	A_23_P342751
FOXC1	NM_001453	9.81	A_23_P390504
FOXC1	NM_001453	8.60	A_32_P205110
FOXL1	NM_005250	3.28	A_23_P365081
FSCN1	NM_003088	2.35	A_23_P168531
FZD7	NM_003507	7.36	A_23_P209449
FZD9	NM_003508	2.57	A_23_P59613
GABRP	NM_014211	17.08	A_23_P328545
GCNT2	NM_001491	2.81	A_24_P397489
GCSH	NM_004483	2.08	A_23_P117933
GEMIN4	NM_015721	2.30	A_23_P66872
GEMIN4	NM_015721	2.06	A_23_P66867
GLI3	NM_000168	3.06	A_23_P111531
GNB4	NM_021629	2.71	A_32_P184916
GPR161	NM_153832	2.42	A_23_P354314
GPRIN2	AB011086	4.51	A_23_P343382
GSG2	AK056691	2.03	A_24_P76521
H2BFXP	NR_003238	4.50	A_32_P85591
HAB1	X83412	3.71	A_24_P541489
HIST1H1A	NM_005325	19.86	A_23_P70448
HLA-DOB	NM_002120	2.41	A_23_P30736
HORMAD1	NM_032132	20.94	A_32_P199884
HPDL	NM_032756	2.63	A_23_P74449
IGF2BP3	NM_006547	3.69	A_23_P19987
IL12RB2	NM_001559	9.61	A_23_P72077
ITGB8	NM_002214	4.58	A_24_P759477
ITGB8	NM_002214	3.06	A_24_P273599
ITGB8	NM_002214	4.26	A_23_P123060
KHDC1	NM_030568	3.00	A_24_P280762
KIF18B	BC048263	2.18	A_24_P680947
KIF1B	NM_183416	2.81	A_24_P649624
KIF1B	NM_183416	2.26	A_24_P145066
KIF20A	NM_005733	2.32	A_23_P256956
KIF2C	NM_006845	2.49	A_23_P34788
KIFC1	NM_002263	3.02	A_23_P133956
KIT	NM_000222	27.08	A_23_P110253
KLHL18	BC015962	2.44	A_24_P916686
KRT14	NM_000526	4.59	A_24_P265346
KRT14	NM_000526	3.54	A_23_P4335
KRT16	NM_005557	18.87	A_23_P38537

KRT16	NM_005557	14.49	A_24_P392991
KRT16P2	NR_029392	10.04	A_32_P62963
KRT17	NM_000422	13.11	A_23_P96158
KRT34	NM_021013	3.42	A_23_P101054
KRT5	NM_000424	11.63	A_23_P218047
KRT6A	NM_005554	4.13	A_23_P87653
KRT6B	NM_005555	80.36	A_23_P76249
KRT6C	NM_173086	8.76	A_23_P366936
KRTAP6-3	NM_181605	2.27	A_24_P391604
L3MBTL4	NM_173464	4.69	A_23_P326474
LBR	NM_002296	3.07	A_23_P200493
LGALS7	NM_002307	5.71	A_24_P238250
LGALS7B	NM_001042507	7.99	A_23_P108062
LGALS7B	NM_001042507	5.50	A_24_P348118
LIN9	NM_173083	2.32	A_32_P233304
LMO4	NM_006769	7.00	A_23_P380181
LOC149351	BC036441	2.61	A_24_P520767
LOC399491	AK023376	2.12	A_24_P238046
LOC401317	BC087859	3.96	A_32_P219135
LOC643650	BC033221	5.20	A_23_P359214
LOC645195	AK123450	2.32	A_32_P224234
LOC729088	CR602569	3.07	A_32_P36143
LOC729683	CR594811	2.96	A_32_P145010
LY6K	NM_017527	9.41	A_23_P397285
MAGOH	NM_002370	2.99	A_23_P200216
MAP7D3	NM_024597	2.02	A_24_P177631
MCM10	NM_182751	2.19	A_23_P161474
MCM10	NM_182751	2.61	A_24_P412088
MIA	NM_006533	32.33	A_23_P4714
MLLT4	BC014505	2.13	A_23_P344694
MTSS1L	NM_138383	2.11	A_32_P84084
NANOGP1	AY455283	4.36	A_24_P68068
NCAPD2	NM_014865	2.05	A_23_P25293
NDC80	NM_006101	2.71	A_23_P50108
NDC80	NM_006101	3.37	A_24_P14156
NFE2L3	NM_004289	3.25	A_23_P42718
NFIL3	NM_005384	2.79	A_23_P32253
NMT2	NM_004808	3.09	A_23_P138686
NPR3	NM_000908	12.31	A_23_P327451
NPR3	NM_000908	11.27	A_23_P253536
NRG2	NM_004883	2.86	A_23_P349857
NRG2	NM_013982	8.75	A_23_P213699
NRTN	NM_004558	4.85	A_23_P90359

NT5DC2	NM_022908	2.52	A_23_P44836
NTRK3	NM_002530	2.48	A_23_P88538
OBSCN	NM_052843	2.77	A_24_P119685
OGFRL1	NM_024576	3.32	A_23_P7791
OIP5	NM_007280	2.52	A_23_P379614
OLFM4	NM_006418	33.54	A_24_P181254
OSBPL3	NM_015550	3.01	A_24_P377499
OSBPL3	NM_015550	2.13	A_23_P215525
PCDHB9	NM_019119	2.89	A_23_P18798
PCYOX1L	NM_024028	2.17	A_23_P30275
PDE7A	NM_002603	2.63	A_23_P123478
PHF19	NM_015651	2.32	A_23_P21436
PHGDH	NM_006623	4.69	A_23_P85783
PIM1	NM_002648	2.68	A_23_P345118
PLEKHG4B	NM_052909	6.27	A_32_P129269
PLEKHG4B	NM_052909	3.72	A_23_P81640
PM20D2	NM_001010853	3.69	A_32_P86118
POLH	NM_006502	2.22	A_32_P69492
POU4F1	NM_006237	6.77	A_23_P205164
PPP1CB	NM_002709	2.02	A_24_P396720
PPP2R5D	NM_180976	2.02	A_24_P294931
PRDM13	NM_021620	23.26	A_23_P256581
PRICKLE1	NM_153026	4.19	A_23_P408285
PROM1	NM_006017	11.97	A_23_P258463
PRPF38A	NM_032864	2.06	A_24_P97001
PTPLA	NM_014241	5.78	A_23_P161352
PTPN14	NM_005401	2.95	A_24_P2648
PTTG1	NM_004219	2.07	A_23_P7636
QKI	NM_006775	2.14	A_24_P941322
RAD54L	NM_003579	2.18	A_23_P74115
RARRES1	NM_002888	8.51	A_23_P18078
RASD2	NM_014310	3.10	A_24_P357100
RAVER2	NM_018211	4.33	A_23_P328323
RGMA	NM_020211	3.96	A_23_P372308
RIOK1	NM_153005	2.03	A_24_P8088
ROPN1	NM_017578	105.93	A_32_P184464
ROPN1B	NM_001012337	72.38	A_24_P417407
SEPHS1	BC064610	2.51	A_24_P90022
SFRP1	NM_003012	11.68	A_23_P10127
SFRP1	NM_003012	20.02	A_23_P10121
SH2D2A	NM_003975	2.06	A_23_P160618
SKP2	NM_032637	2.16	A_23_P156310
SLC19A3	NM_025243	4.40	A_23_P39871

SLC25A37	AF495725	2.23	A_24_P64100
SLC25A37	AF113696	2.45	A_23_P216004
SLC27A6	NM_001017372	5.51	A_23_P41789
SLC6A2	NM_001172504	2.45	A_24_P910660
SLC6A2	NM_001043	5.57	A_23_P358345
SOSTDC1	NM_015464	23.12	A_23_P145841
SOX10	NM_006941	4.36	A_23_P143694
SPC25	NM_020675	2.37	A_23_P51085
SPEG	AK055387	4.35	A_23_P338919
STAC	NM_003149	8.27	A_23_P121061
STAC	NM_003149	12.00	A_24_P234415
STMN1	NM_203401	3.19	A_23_P200866
STRA8	NM_182489	3.78	A_24_P203308
TAGAP	NM_138810	4.07	A_23_P339588
TBX19	NM_005149	2.20	A_23_P137705
TCF7L1	NM_031283	11.54	A_23_P142872
TDP1	NM_018319	2.21	A_23_P117623
TFCP2L1	NM_014553	4.43	A_23_P5301
TMEM74	NM_153015	3.14	A_23_P390139
TMSB15B	NM_194324	14.78	A_23_P96599
TNNI2	NM_003282	4.28	A_23_P24784
TPX2	NM_012112	2.25	A_23_P68610
TRDMT1	NM_004412	3.21	A_24_P252705
TRDMT1	NM_004412	4.32	A_23_P115636
TRIM29	NM_012101	5.14	A_23_P203267
TSLP	NM_033035	6.90	A_23_P121987
TTLL4	NM_014640	2.38	A_23_P142697
TYMS	NM_001071	2.90	A_23_P50096
UBASH3B	NM_032873	2.32	A_24_P192933
UBE2E3	NM_006357	2.18	A_24_P148450
UBE2E3	NM_006357	2.42	A_23_P11192
UCK2	NM_012474	2.41	A_23_P487
UGT8	NM_003360	8.36	A_24_P103264
UGT8	U62899	7.67	A_23_P72747
UGT8	AL137342	8.21	A_24_P942589
USP1	NM_003368	2.78	A_23_P11652
USP31	NM_020718	2.19	A_24_P390583
VGLL1	NM_016267	3.53	A_23_P253123
WNT11	NM_004626	7.66	A_24_P253003
WNT6	NM_006522	9.06	A_23_P119916
XPO5	NM_020750	2.55	A_23_P256855
YBX1	NM_004559	2.18	A_23_P34767
YBX1	NM_004559	2.13	A_32_P218989

YBX1	NM_004559	2.08	A_24_P101391
ZCCHC11	NM_001009881	2.84	A_23_P34433
ZCCHC18	NM_001143978	3.51	A_32_P116989
ZFP28	NM_020828	2.33	A_23_P107673
ZNF232	NM_014519	2.11	A_23_P4294
ZNF286A	AF086305	4.00	A_24_P910833
ZNF318	NM_014345	2.36	A_23_P145175
ZNF462	NM_021224	5.64	A_23_P60499
ZNF644	NM_201269	2.14	A_23_P200772
**GeneSymbol**	**GenbankAcce:**	**FC**	**ProbeName**
**Downregulated Genes**			
ABCA12	NM_173076	-25.43	A_23_P56369
ABCC11	NM_033151	-7.95	A_23_P141076
ABCG1	NM_207627	-3.43	A_23_P166297
ACADSB	NM_001609	-3.25	A_23_P158570
ACADSB	NM_001609	-5.48	A_32_P31945
ACOX2	NM_003500	-17.55	A_23_P10182
ACSM1	NM_052956	-7.65	A_23_P106933
ACSM3	NM_202000	-2.26	A_23_P317756
AGR2	NM_006408	-60.78	A_23_P31407
AGR3	NM_176813	-7.58	A_23_P42811
ALCAM	NM_001627	-4.64	A_32_P74643
ANKRA2	NM_023039	-2.54	A_24_P337397
ANKRA2	NM_023039	-2.08	A_23_P159012
ANKRD30A	NM_052997	-8.90	A_23_P12533
ANKRD42	NM_182603	-5.05	A_24_P357572
ANKRD42	NM_182603	-5.51	A_32_P69166
ANXA11	NM_145869	-2.38	A_24_P107142
ANXA9	NM_003568	-4.52	A_23_P103617
AR	NM_000044	-8.61	A_23_P113111
ARFIP1	NM_001025595	-2.90	A_23_P95050
ARFIP1	NM_001025595	-2.23	A_24_P166094
ARHGEF16	NM_014448	-2.41	A_23_P114670
ARRDC1	NM_152285	-2.07	A_23_P391607
ATE1	NM_001001976	-2.48	A_23_P138574
ATG16L1	NM_030803	-2.21	A_32_P113508
ATP8A1	NM_006095	-4.68	A_23_P30075
ATP8B1	NM_005603	-2.61	A_23_P107597
BAG3	NM_004281	-2.32	A_23_P47077
BBS1	NM_024649	-2.25	A_24_P184305
BCAS4	NM_001010974	-4.37	A_24_P143492
C11orf52	NM_080659	-3.20	A_23_P1722

C11orf54	NM_014039	-2.34	A_23_P202750
C11orf54	NM_014039	-2.92	A_24_P201404
C11orf65	NM_152587	-2.96	A_23_P418485
C12orf72	NM_173802	-2.72	A_23_P404120
C14orf25	BC038110	-8.35	A_24_P393596
C14orf79	NM_174891	-2.74	A_23_P412707
C14orf79	NM_174891	-2.36	A_23_P420981
C14orf79	NM_174891	-2.60	A_23_P376870
C16orf71	NM_139170	-3.39	A_23_P414281
C17orf28	NM_030630	-5.39	A_23_P118412
C19orf21	NM_173481	-12.16	A_23_P390068
C20orf112	AK097804	-4.91	A_23_P303548
C2CD2L	NM_014807	-2.24	A_23_P353056
C2orf55	NM_207362	-2.74	A_24_P272313
C4orf33	NM_173487	-2.44	A_23_P433152
C4orf34	NM_174921	-3.92	A_23_P112634
C6orf97	NM_025059	-3.19	A_23_P93514
C9orf116	NM_001048265	-2.37	A_23_P422115
C9orf98	NM_152572	-2.17	A_23_P83200
CA12	NM_001218	-4.32	A_24_P330518
CA12	NM_001218	-3.74	A_23_P372234
CAB39	NM_016289	-2.44	A_23_P79426
CACNA1D	NM_000720	-5.99	A_23_P365767
CADPS2	NM_017954	-4.78	A_24_P246710
CAPN13	NM_144575	-19.66	A_23_P101972
CAPN13	NM_144575	-11.13	A_24_P336983
CAPN13	AK074418	-7.02	A_23_P345068
CAPN8	NM_001143962	-8.64	A_23_P83381
CASC1	NM_018272	-8.40	A_23_P95231
CASP10	NM_032974	-2.96	A_24_P3045
CASP8	NM_033355	-5.57	A_24_P157087
CCDC111	NM_152683	-2.06	A_23_P358470
CCDC125	NM_176816	-2.11	A_23_P432591
CCDC159	NM_001080503	-2.18	A_24_P75920
CCDC87	NM_018219	-3.37	A_23_P127484
CCDC96	NM_153376	-2.63	A_32_P69930
CDK17	NM_002595	-3.32	A_23_P33376
CEACAM6	BC005008	-23.36	A_23_P421483
CEACAM7	NM_006890	-9.54	A_24_P228302
CHST15	NM_015892	-3.54	A_23_P383986
CIRBP	NM_001280	-2.41	A_23_P142322
COG3	NM_031431	-2.75	A_23_P2692
COG3	NM_031431	-2.01	A_24_P296070

COL4A3BP	NM_001130105	-2.66	A_24_P29277
COL4A3BP	AF136450	-2.42	A_23_P61748
COX15	NM_078470	-2.30	A_24_P115700
CPD	NM_001304	-3.46	A_23_P207837
CPEB2	NM_182485	-2.40	A_32_P225355
CPEB3	NM_014912	-3.35	A_23_P46813
CTAGE1	NM_172241	-2.72	A_24_P305223
CTSO	NM_001334	-5.63	A_23_P110175
CYB5R1	NM_016243	-2.30	A_23_P52101
CYP3A7	NM_000765	-9.66	A_23_P358917
CYP4F8	NM_007253	-15.89	A_23_P131060
DACH1	NM_080759	-11.21	A_23_P32577
DAK	NM_015533	-2.62	A_23_P36129
DALRD3	NM_018114	-2.08	A_23_P135611
DCAF8	NR_028105	-2.54	A_24_P76725
DNAH7	NM_018897	-6.59	A_23_P33583
DNAJC30	NM_032317	-2.07	A_23_P157170
DUSP5	NM_004419	-4.31	A_23_P150018
DYNLRB2	NM_130897	-8.21	A_23_P94840
EDEM1	NM_014674	-2.54	A_24_P285768
EFCAB6	NM_022785	-3.93	A_23_P68978
ELF1	NM_172373	-2.15	A_23_P2801
ELF1	NM_172373	-3.08	A_24_P78590
ELMOD2	NM_153702	-2.95	A_23_P305692
EME2	AK074080	-2.88	A_23_P366125
ENPP3	NM_005021	-10.23	A_23_P404536
EPB41L5	BC032822	-3.55	A_23_P209298
EPS8L1	NM_133180	-4.50	A_23_P208779
ERGIC1	NM_001031711	-2.95	A_23_P333218
ERGIC1	NM_001031711	-3.36	A_24_P89257
ERGIC1	NM_001031711	-3.95	A_24_P97770
ERGIC1	NM_001031711	-2.88	A_23_P404871
ETNK1	NM_018638	-2.71	A_23_P419239
EXD3	NM_017820	-2.56	A_24_P49183
FAM174B	NM_207446	-4.03	A_23_P100001
FAM179B	NM_015091	-4.01	A_23_P3102
FAM47E	NM_001136570	-4.15	A_32_P83811
FAM84B	NM_174911	-4.35	A_24_P329487
FBP1	NM_000507	-3.37	A_23_P257111
FDXACB1	NM_138378	-2.70	A_23_P362261
FFAR2	NM_005306	-2.43	A_23_P397391
FLJ38379	AK095698	-6.66	A_24_P194661
FLJ40504	NR_028334	-2.93	A_23_P373708

FOXA1	NM_004496	-22.39	A_23_P37127
FOXA1	NM_004496	-21.00	A_24_P347431
FSIP1	NM_152597	-10.52	A_23_P353125
FUCA1	NM_000147	-2.50	A_23_P11543
FUT8	NM_178154	-2.01	A_23_P313632
GALNT10	NM_198321	-3.91	A_23_P7706
GALNT10	AK021777	-3.05	A_23_P19102
GALNT10	NM_198321	-4.50	A_24_P910923
GALNT6	NM_007210	-4.60	A_23_P204133
GATA2	NM_032638	-2.42	A_24_P165998
GATA2	NM_032638	-3.98	A_23_P110022
GDF15	NM_004864	-6.20	A_23_P16523
GGT1	NM_005265	-2.62	A_24_P76267
GGT1	NM_005265	-2.42	A_23_P120809
GGT1	NM_005265	-2.72	A_23_P154986
GGT1	NM_005265	-2.30	A_24_P178175
GGTLC1	NM_178311	-3.42	A_23_P57199
GGTLC2	NM_199127	-3.02	A_24_P59062
GGTLC2	NM_199127	-2.52	A_32_P319200
GLOD5	NM_001080489	-6.08	A_23_P95619
GNA14	NM_004297	-8.32	A_23_P169479
GPD1L	NM_015141	-2.68	A_23_P318284
GPR160	NM_014373	-5.30	A_23_P167005
GPRC5C	AK000249	-5.01	A_23_P346670
GPRC5C	NM_022036	-4.88	A_23_P38167
GPRC5C	AK000249	-12.33	A_23_P346673
GPRC5C	NM_022036	-5.45	A_32_P109029
GRPEL1	AF070525	-3.66	A_24_P166045
GSR	BC035691	-5.80	A_32_P31618
HNF1B	NM_000458	-3.58	A_23_P409287
HOXB3	NM_002146	-13.06	A_24_P399220
HPX	NM_000613	-3.77	A_23_P161998
INPP4B	BC005273	-4.34	A_24_P915492
IQCD	NM_138451	-2.23	A_24_P390060
ITGB5	NM_002213	-2.69	A_23_P166633
ITPR1	NM_002222	-3.25	A_23_P92042
KBTBD3	NM_198439	-4.04	A_23_P127557
KBTBD3	NM_198439	-2.63	A_23_P127553
KIAA0232	NM_014743	-2.12	A_23_P327069
KIAA0556	NM_015202	-2.08	A_23_P381203
KIAA0564	NM_015058	-2.31	A_23_P432077
KIAA1244	NM_020340	-6.28	A_32_P188186
KIAA1370	NM_019600	-3.27	A_23_P99853

KIAA1370	NM_019600	-3.66	A_24_P357576
KIAA1407	NM_020817	-2.34	A_23_P419213
KIAA1826	NM_032424	-2.02	A_23_P116168
KIF9	NM_022342	-3.41	A_24_P225878
KITLG	NM_000899	-9.12	A_23_P204654
KLHDC9	NM_001007255	-2.97	A_23_P86100
KLHL8	NM_020803	-2.72	A_23_P328729
KRR1	NM_007043	-2.20	A_32_P326819
KRT18	NM_000224	-2.84	A_23_P99320
KRT18	NM_000224	-2.50	A_24_P42136
KRT18	L32537	-2.35	A_24_P924957
KRT18	NM_000224	-2.87	A_32_P151544
LASP1	NM_006148	-2.24	A_23_P89187
LASS6	NM_203463	-2.07	A_24_P289366
LCA5L	NM_152505	-4.51	A_32_P48466
LFNG	NM_001040167	-3.54	A_23_P8452
LIMA1	NM_016357	-2.06	A_23_P151267
LOC100129034	NR_027406	-3.61	A_32_P91042
LOC145837	NR_026979	-9.12	A_32_P46594
LOC220429	NR_003268	-2.60	A_24_P358054
LOC254057	AK024653	-3.19	A_24_P450092
LOC375295	BC013438	-4.82	A_23_P302787
LOC375295	BC013438	-3.80	A_32_P16204
LOC440335	NR_029454	-6.76	A_24_P229884
LOC442249	XR_019231	-2.73	A_24_P256063
LOC643783	XM_931798	-2.47	A_32_P12327
LOC645431	NR_024334	-3.50	A_23_P327156
LOC90246	NR_026954	-2.30	A_24_P532180
LPPR2	NM_022737	-2.29	A_23_P153461
LRBA	NM_006726	-2.70	A_24_P360078
LRFN2	NM_020737	-5.20	A_32_P82111
LRP10	NM_014045	-2.49	A_23_P205499
LRRC27	NM_030626	-2.51	A_32_P186157
LRRC6	NM_012472	-3.76	A_23_P112004
LYSMD3	NM_198273	-2.11	A_24_P15658
MAN2B2	NM_015274	-2.01	A_23_P250380
MAN2B2	NM_015274	-2.01	A_23_P250379
MCCC2	NM_022132	-2.46	A_23_P18887
MLPH	NM_024101	-12.51	A_23_P165778
MLPH	NM_024101	-8.63	A_23_P165783
MLPH	NM_001042467	-6.51	A_23_P154400
MMEL1	NM_033467	-2.63	A_23_P138294
MPZL2	NM_005797	-3.52	A_24_P278552

MSX2	NM_002449	-7.96	A_24_P132006
MTHFR	NM_005957	-2.16	A_23_P400078
N4BP2L2	NM_033111	-2.34	A_23_P65262
NAIP	NM_004536	-2.51	A_24_P72139
NAT1	NM_000662	-4.38	A_23_P95594
NAT2	NM_000015	-5.26	A_23_P31798
NEK11	NM_024800	-2.88	A_23_P211973
NEK11	NM_145910	-2.30	A_23_P155301
NEK6	NM_014397	-2.83	A_23_P216920
NEK9	NM_033116	-2.39	A_23_P3131
NME3	NM_002513	-2.32	A_23_P152115
NPDC1	NM_015392	-2.28	A_23_P146572
NUDT16	NM_152395	-2.05	A_23_P310560
NUDT4	NM_199040	-2.14	A_24_P335263
NUDT4	NM_199040	-2.10	A_24_P50753
NUDT4	NM_199040	-2.23	A_32_P117723
NUDT4	NM_199040	-2.99	A_24_P67946
OVGP1	NM_002557	-4.28	A_23_P103756
P4HTM	NM_177938	-2.15	A_23_P113317
PAAF1	NM_025155	-2.05	A_23_P139339
PAFAH2	NM_000437	-2.42	A_24_P71153
PARP9	NM_031458	-2.94	A_23_P69383
PAX9	NM_006194	-12.96	A_32_P70818
PBLD	NM_022129	-2.07	A_23_P149998
PBLD	NM_022129	-2.68	A_24_P112395
PCDH1	NM_032420	-2.07	A_24_P234838
PGPEP1	NM_017712	-2.08	A_23_P218531
PHF8	BC042108	-4.36	A_23_P9744
PIP	NM_002652	-38.77	A_23_P8702
PNPLA4	NM_004650	-4.34	A_24_P943815
POLD4	NM_021173	-2.27	A_23_P127367
PRMT10	NM_138364	-2.16	A_23_P41541
PRR13	NM_001005354	-2.33	A_24_P349466
PRR15	NM_175887	-11.19	A_32_P154911
PRR15	NM_175887	-3.42	A_23_P431346
RAB27B	NM_004163	-9.45	A_23_P107612
RAB43	NM_198490	-2.87	A_23_P77552
RAB43	NM_198490	-2.19	A_32_P86318
RAB43	NM_198490	-2.12	A_32_P205859
RALGPS2	NM_152663	-4.65	A_24_P173746
RAP2C	NM_021183	-2.35	A_23_P147826
RAPGEF3	NM_006105	-3.41	A_23_P151307
RBM47	NM_019027	-3.82	A_24_P226108

RBM47	NM_019027	-5.45	A_23_P132910
RERE	NM_012102	-2.52	A_23_P85414
RET	NM_020975	-8.60	A_23_P202245
RNASEL	NM_021133	-3.05	A_23_P390172
RND1	NM_014470	-3.65	A_23_P53370
RNF103	NM_005667	-2.11	A_23_P56709
RNF148	NM_198085	-12.10	A_24_P170234
RSPH1	NM_080860	-3.09	A_23_P102950
RUNDC1	NM_173079	-2.82	A_24_P395621
SCOC	NM_032547	-3.31	A_23_P167293
SCOC	NM_032547	-2.08	A_24_P46334
SDSL	NM_138432	-2.46	A_23_P53439
SEC16A	NM_014866	-3.25	A_23_P251303
SEC23IP	NM_007190	-2.67	A_24_P227017
SELENBP1	NM_003944	-4.44	A_23_P74619
SEPHS2	NM_012248	-2.03	A_23_P146798
SH3BP4	NM_014521	-2.86	A_23_P79259
SIDT1	NM_017699	-13.63	A_23_P132515
SLC10A7	NM_001029998	-2.72	A_23_P213085
SLC22A18	NM_183233	-2.01	A_23_P139260
SLC25A23	NM_024103	-2.69	A_23_P130598
SLC2A10	NM_030777	-4.31	A_24_P271323
SLC39A11	NM_139177	-3.51	A_24_P57528
SLC40A1	NM_014585	-7.13	A_23_P102391
SLC44A4	NM_025257	-46.28	A_23_P93349
SLC44A4	NM_025257	-30.30	A_24_P684183
SMPDL3A	NM_006714	-5.81	A_32_P223859
SNAP29	NM_004782	-2.83	A_24_P48862
SNX25	NM_031953	-2.14	A_24_P303097
SPAG17	NM_206996	-4.88	A_23_P319783
SPDEF	NM_012391	-4.30	A_23_P111194
SPEF2	NM_024867	-4.91	A_32_P179396
SPRED2	NM_181784	-4.73	A_32_P225854
SSH3	NM_017857	-2.57	A_24_P266734
SSH3	NM_017857	-2.61	A_23_P150147
ST3GAL5	NM_003896	-4.30	A_23_P136573
ST3GAL5	NM_003896	-5.74	A_23_P311869
STRN3	NM_014574	-3.54	A_23_P392076
STRN3	NM_014574	-3.00	A_23_P65410
SULT1C2	NM_176825	-7.76	A_23_P51002
SUOX	NM_000456	-2.52	A_23_P150857
TADA2B	NM_152293	-2.85	A_24_P687582
TADA2B	NM_152293	-2.03	A_23_P396541

TANC2	NM_025185	-2.31	A_24_P942068
TBC1D9	NM_015130	-5.25	A_23_P41487
TFF3	NM_003226	-12.12	A_24_P245778
TFF3	NM_003226	-8.39	A_24_P289208
TFF3	NM_003226	-26.21	A_23_P393099
TFF3	NM_003226	-21.51	A_23_P257296
THSD4	NM_024817	-4.66	A_23_P148249
TM7SF2	NM_003273	-3.58	A_23_P116037
TMC4	NM_144686	-2.60	A_23_P330461
TMC5	NM_024780	-52.22	A_23_P15101
TMEM135	NM_022918	-3.80	A_24_P181101
TMEM135	NM_022918	-2.61	A_24_P127075
TMEM135	NM_022918	-3.72	A_23_P203751
TMEM141	NM_032928	-2.19	A_23_P94591
TMEM192	NM_001100389	-3.51	A_24_P20524
TMEM192	NM_001100389	-3.46	A_23_P253677
TMEM45B	NM_138788	-34.42	A_23_P1682
TMEM62	NM_024956	-3.11	A_23_P49041
TMEM86A	NM_153347	-5.11	A_32_P66035
TMEM87B	NM_032824	-2.01	A_23_P91076
TMEM87B	NM_032824	-3.50	A_23_P303155
TNFSF13	NM_172088	-3.07	A_23_P152620
TP53I11	NM_001076787	-4.24	A_24_P160969
TPCN1	NM_001143819	-2.19	A_24_P244575
TPCN1	NM_001143819	-2.46	A_23_P218086
TRAF3IP1	NM_015650	-2.63	A_23_P5359
TSC22D1	NM_183422	-2.69	A_23_P162739
TSC22D3	NM_004089	-2.46	A_23_P217688
TSPAN1	NM_005727	-3.95	A_23_P160167
TTC12	NM_017868	-2.85	A_24_P73075
UBR3	NM_172070	-2.26	A_23_P5405
UBXN10	NM_152376	-9.52	A_23_P85664
UBXN10	BX648631	-6.35	A_32_P205053
UPRT	NM_145052	-2.24	A_23_P159865
USP38	NM_032557	-3.01	A_23_P44734
WDR52	NM_001164496	-2.92	A_23_P110090
WFS1	NM_006005	-2.13	A_23_P121499
WWP1	NM_007013	-2.15	A_23_P146990
XBP1	NM_001079539	-4.89	A_24_P100228
ZNF136	NM_003437	-2.05	A_23_P147121
ZNF304	NM_020657	-2.35	A_24_P228266
ZNF467	BC038972	-3.94	A_23_P59470
ZNF597	NM_152457	-5.89	A_23_P3753
