# Supplementary material for: Integrated Genomic and Epigenomic Analysis of Breast Cancer Brain Metastasis
Source: PLoS One. 2014 Jan 29;9(1):e85448. doi: 10.1371/journal.pone.0085448 (PMC3906004; doi:10.1371/journal.pone.0085448)
Supplement: File S1 — Supporting figures and tables. Figure S1: Combined Network for Upstream Analysis of FOXM1 and TBX2. The downstream genes connected to FOXM1 and TBX2 were illustrated as a network in IPA. The mRNA expression ratios are listed below the gene nodes. The legend within figure describes the node and edge color keys. Figure S2: Word Cloud Analysis of Cluster Enrichments. We have used word clouds to visually summarize the textual results from the enrichment analysis of each gene cluster as observed in Figure 3. The results were generated using www.wordle.net web resource. The larger the word, the more times it is mentioned in the enrichment categories. Supplementary Tables in File S1. Table S1a. Table S1b. Table S2. Table S3a. Table S3b. Table S4a. Figure S1. Table S4b. Table S5a–b. Table S6a–b. Table S7. Table S8a–f. Table S9a–f. Figure S2. Table S10. Table S11a–c. Table S11d. Table S12. Table S13. Table S14. (ZIP) [file pone.0085448.s001.zip › Supplementary Table S7.pdf]

Table 7.Union of genes differentially expressed gene list in BBM of different intrinsic subtypes. Values represent Log2 normalized ratios.

Gene Symbol	GenBank Accession	Basal-like	Her2+/ER-	Luminal B
ABCC11	NM_033151	-0.33	2.66	2.82
ABCG1	NM_207627	0.32	2.09	2.13
ACADSB	NM_001609	-1.59	0.11	0.18
ACADSB	NM_001609	-2.23	0.22	0.44
ACSM1	NM_052956	-1.14	1.80	1.37
ACSS2	NM_018677	-0.68	-0.96	-2.12
ADAMTS3	NM_014243	-0.77	-1.51	-3.87
ADAT2	NM_182503	-0.88	-1.65	-2.48
ADAT2	NM_182503	-0.72	-1.01	-1.93
ADORA2B	NM_000676	-0.38	-1.64	-2.66
ADSSL1	NM_199165	-1.79	-1.00	-0.10
AFF3	NM_002285	-1.04	-1.48	1.89
AFF4	NM_014423	-0.90	-0.34	0.22
AGBL5	NM_001035507	-0.01	-1.00	-1.39
AGGF1	NM_018046	-0.82	-0.33	0.24
AGR2	NM_006408	-4.68	1.25	3.83
AGR3	NM_176813	-3.09	-0.17	4.58
AKR1E2	AB040820	2.15	1.01	0.10
AMOTL1	NM_130847	-0.93	-1.01	-2.18
AMOTL1	NM_130847	-0.83	-0.89	-2.40
ANKRA2	NM_023039	-0.90	0.45	0.43
ANKRA2	NM_023039	-0.10	0.85	1.06
ANKRA2	NM_023039	-0.45	0.61	0.81
ANKRD30A	NM_052997	0.52	3.68	5.90
ANKRD36	NM_001164315	-0.26	-1.10	-1.79
ANKRD36BP1	NR_026844	1.39	0.75	-0.29
ANKRD42	NM_182603	-0.89	1.44	1.28
ANKRD42	NM_182603	-0.78	1.68	1.30
ANKS6	NM_173551	-0.11	-0.96	-1.80
ANP32E	NM_030920	-0.02	-2.10	-1.91
ANP32E	NM_030920	0.31	-2.02	-1.70
ANXA8L2	BC008813	3.52	-0.27	-0.02
ANXA8L2	NM_001630	2.40	-2.17	-1.86
ANXA9	NM_003568	-2.08	0.10	1.67
APBB2	NM_004307	0.59	0.41	2.00
APBB2	NM_173075	0.68	0.20	1.84
APPL2	NM_018171	-0.48	0.15	0.59

AR	NM_000044	-1.58	1.52	3.60
ARFIP1	NM_001025595	0.42	1.57	1.04
ARFIP2	NM_012402	-0.21	0.67	0.90
ARHGAP11A	NM_014783	-0.79	-2.38	-2.11
ARHGEF16	NM_014448	-0.17	1.10	1.05
ARMC9	AB058771	-0.96	-0.28	0.31
ARRDC1	NM_152285	-0.11	0.94	0.56
ART3	NM_001179	2.94	-1.50	-1.64
ASPM	NM_018136	-0.54	-1.90	-1.93
ASPM	NM_018136	-0.81	-2.23	-2.27
ASS1	NM_000050	-0.46	0.23	-2.17
ATG16L1	NM_030803	0.35	1.50	1.69
ATL2	NM_022374	1.03	0.09	-0.30
ATP11C	NM_173694	0.57	-0.14	-0.66
ATP11C	NM_001010986	0.66	-0.24	-0.86
ATP6AP1	NM_001183	0.49	0.50	1.57
ATP6V1C2	NM_001039362	3.31	1.86	-0.55
ATP8B1	NM_005603	0.00	1.39	1.67
AURKB	NM_004217	-0.62	-1.57	-1.83
B3GNT5	NM_032047	2.44	0.52	-0.80
BAG3	NM_004281	0.37	1.58	1.32
BBS1	NM_024649	-0.43	0.74	0.80
BBS4	NM_033028	-0.64	-0.38	0.60
BCAS4	NM_001010974	-0.80	1.33	1.47
BCL11A	NM_022893	1.34	-0.67	-2.04
BCL11A	NM_018014	2.23	-0.44	-2.05
BCL11A	NM_022893	1.51	-1.77	-3.74
BCL2	NM_000633	0.11	-1.16	1.90
BOP1	NM_015201	0.20	-0.68	-1.26
BPI	NM_001725	1.48	-0.01	-0.24
BTG3	BC028229	0.13	-1.16	-2.16
BTG3	NM_006806	0.65	-0.18	-1.80
BYSL	NM_004053	0.85	-0.11	-0.44
C11orf52	NM_080659	1.51	3.19	2.43
C11orf75	NM_020179	0.87	1.88	-0.46
C12orf72	NM_173802	-0.20	1.25	0.99
C13orf38	NM_001144982	-0.55	-1.19	-3.03
C14orf25	BC038110	-1.04	2.03	3.13
C14orf45	NM_025057	0.36	1.20	1.76
C14orf79	NM_174891	-1.23	0.22	0.62
C14orf79	NM_174891	-0.49	0.75	0.90
C14orf79	NM_174891	-1.06	0.32	0.64
C15orf23	NM_001142761	-0.03	-1.01	-1.23

C15orf41	NM_032499	0.68	-0.24	-0.61
C15orf42	NM_152259	-0.43	-1.40	-1.88
C15orf42	NM_152259	-0.63	-1.53	-1.95
C16orf71	NM_139170	0.16	1.92	2.14
C17orf28	NM_030630	0.57	3.00	2.99
C19orf21	NM_173481	-3.36	0.24	-0.15
C19orf51	NM_178837	-1.86	-0.92	-0.19
C1orf163	NM_023077	-0.04	-1.20	-1.18
C1orf198	NM_032800	2.24	0.99	0.79
C1QTNF3	NM_181435	-0.92	0.07	1.32
C1QTNF3	NM_181435	-0.85	-0.12	1.04
C20orf112	AK097804	-1.47	0.83	0.37
C20orf114	NM_033197	0.77	0.83	3.99
C20orf26	NM_015585	1.27	2.47	4.34
C21orf91	NM_017447	0.78	-0.53	-0.92
C22orf23	AK097339	1.58	0.20	-0.50
C2orf3	EF158467	-0.46	-1.09	-1.52
C2orf55	NM_207362	2.34	3.79	3.88
C2orf81	NM_001145054	-0.57	-0.12	0.75
C3orf26	NM_032359	-0.42	-1.41	-1.61
C3orf62	NM_198562	-0.95	-0.24	0.27
C4orf34	NM_174921	-0.50	1.47	1.51
C4orf7	NM_152997	2.87	-0.37	-1.02
C5orf23	NM_024563	2.78	-1.46	-0.71
C5orf44	NM_024941	-0.27	0.47	0.88
C5orf46	NM_206966	4.19	-0.11	0.82
C6orf1	NM_178508	0.15	0.68	1.29
C6orf1	NM_178508	-0.15	0.38	1.17
C6orf15	NM_014070	3.47	0.42	0.18
C6orf173	NM_001012507	0.47	-1.52	-1.77
C6orf173	NM_001012507	0.57	-1.38	-1.59
C6orf218	NR_027793	-1.12	-3.39	-3.64
C6orf97	NM_025059	-2.59	-0.92	0.96
C8orf85	NM_001025357	4.74	0.93	1.99
C9orf116	NM_001048265	-0.57	0.67	0.71
C9orf40	NM_017998	-0.10	-0.97	-1.15
C9orf40	NM_017998	-0.72	-2.16	-2.82
C9orf75	NM_173691	-0.83	0.07	0.45
C9orf98	NM_152572	0.15	1.27	1.87
CA12	NM_001218	-2.07	0.04	2.57
CA12	AK000158	0.08	0.67	3.09
CA12	AK000158	0.20	1.01	2.62
CA12	NM_001218	-2.11	-0.20	2.23

CACNA1D	NM_000720	-0.86	1.73	1.72
CACNA2D2	NM_001005505	-2.36	-0.82	0.95
CADPS2	NM_017954	-0.42	1.83	1.56
CALCOCO2	NM_005831	0.08	0.34	1.33
CAPN13	NM_144575	-0.02	4.27	4.03
CAPN13	NM_144575	0.78	4.25	3.75
CAPN13	AK074418	-0.72	2.09	1.85
CAPN6	NM_014289	0.57	-1.78	-2.03
CAPN8	NM_001143962	-0.94	2.17	2.27
CASC4	NM_138423	-0.30	0.60	0.97
CASC5	NM_170589	-2.06	-2.45	-3.18
CCDC111	NM_152683	-0.57	0.48	0.37
CCDC125	NM_176816	-0.29	0.79	1.44
CCDC159	NM_001080503	-0.10	1.02	1.07
CCDC24	NM_152499	0.05	0.73	1.29
CCDC30	AY639646	-0.48	0.17	1.02
CCDC48	NM_024768	1.64	3.32	4.67
CCDC67	NM_181645	1.40	0.43	-1.39
CCDC74B	NM_207310	-1.35	-0.89	0.71
CCDC74B	NM_207310	-1.20	-0.80	0.79
CCDC87	NM_018219	-0.37	1.38	1.88
CCDC96	NM_153376	0.20	1.60	1.92
CCNA2	NM_001237	-0.75	-1.79	-2.24
CCNB2	NM_004701	-0.44	-1.74	-1.56
CCND1	NM_053056	-1.07	-0.67	1.28
CCND1	NM_053056	-1.75	-1.21	1.26
CCND1	NM_053056	-1.80	-1.68	0.38
CDC20	NM_001255	-0.38	-1.82	-1.76
CDC45L	NM_003504	-1.42	-2.23	-2.77
CDCA2	NM_152562	-0.32	-2.04	-2.19
CDCA3	NM_031299	0.08	-0.87	-1.16
CDCA5	NM_080668	-0.68	-1.41	-1.93
CDCA7	NM_031942	-0.55	-3.00	-3.72
CDCA7	NM_031942	-0.43	-2.53	-3.38
CDCA7L	NM_018719	-0.11	-0.42	-1.65
CDCA8	NM_018101	-0.31	-1.75	-1.94
CDH3	NM_001793	2.87	1.89	0.45
CDK17	NM_002595	-0.13	1.60	1.31
CDK6	NM_001259	-0.90	-3.77	-3.71
CDKN2C	NM_078626	-0.49	-2.08	-1.58
CDYL2	NM_152342	-1.92	-0.41	0.41
CEACAM6	BC005008	-0.20	4.35	5.77
CEACAM7	NM_006890	0.98	4.23	4.95

CENPA	NM_001809	-0.02	-1.11	-1.51
CENPF	NM_016343	-0.03	-1.47	-1.34
CENPN	NM_018455	-0.93	-2.37	-2.14
CERK	NM_022766	0.61	0.05	-1.16
CES8	NM_173815	0.75	1.60	3.02
CHAC2	NM_001008708	0.27	-0.32	-1.44
CHEK1	NM_001274	-1.07	-1.89	-2.60
CHODL	NM_024944	2.91	0.90	-0.45
CHRD	NM_003741	-0.82	-0.61	1.43
CHRM3		-0.20	-3.57	-2.32
CHST15	NM_015892	-0.58	1.24	1.38
CHST3	NM_004273	1.06	0.07	-0.41
CIRBP	NR_023312	-0.90	-0.52	0.36
CIRBP	NR_023312	-0.39	0.12	1.16
CIRBP	NM_001280	-0.20	1.06	1.37
CIRBP	NR_023312	-0.69	-0.12	1.08
CIRH1A	NM_032830	-0.21	-1.23	-1.07
CKS1B	NM_001826	0.40	-0.99	-0.95
CKS1B	NM_001826	-0.03	-1.00	-1.06
CKS1B	NM_001826	0.33	-1.02	-0.92
CKS2	NM_001827	-0.58	-1.91	-1.78
CLDN8	NM_199328	4.91	5.18	1.07
CLIC4	NM_013943	0.80	-0.63	-0.18
CLIP4	NM_024692	0.64	-0.14	-1.24
CMBL	NM_138809	-2.12	-0.95	0.62
CNGA1	NM_000087	2.93	1.62	0.55
CNTNAP3	NM_033655	0.38	-1.86	-2.55
COG3	NM_031431	0.36	1.37	1.00
COL24A1	NM_152890	-1.03	-0.46	1.09
COL4A3BP	NM_001130105	-0.71	0.69	0.55
COL4A3BP	AF136450	-0.74	0.54	0.31
COMMD10	NM_016144	-0.46	-0.43	0.62
CPEB2	NM_182485	-1.80	-0.54	0.32
CPEB2	NM_182485	-1.66	-0.90	-0.08
CPEB3	NM_014912	-0.11	1.63	1.16
CRABP1	NM_004378	0.21	-1.58	-1.60
CREB3L2	BC063666	0.42	0.15	-1.04
CSDA	NM_003651	0.43	-0.20	-1.10
CSDA	NM_003651	0.15	-0.46	-1.40
CST3	NM_000099	-0.36	-0.25	1.03
CST5	NM_001900	-0.66	-0.50	0.82
CTAGE1	NM_172241	-0.30	1.14	0.59
CTPS	NM_001905	-0.41	-1.36	-1.78

CTSL2	NM_001333	0.08	-1.45	-1.94
CTSO	NM_001334	1.77	4.26	3.43
CXXC5	NM_016463	-0.79	-0.34	0.98
CXXC5	NM_016463	-1.00	-0.59	0.96
CYB5R1	NM_016243	0.50	1.70	1.71
CYB5R2	NM_016229	1.98	-0.26	0.36
CYP39A1	NM_016593	2.31	-0.26	-0.47
CYP3A7	NM_000765	-2.78	0.49	-0.26
CYP4F8	NM_007253	-2.20	1.79	0.09
DACH1	NM_080759	0.61	4.10	6.74
DAK	NM_015533	-1.77	-0.38	-0.57
DALRD3	NM_018114	0.00	1.06	1.30
DCLK1	NM_004734	-1.01	-0.81	1.29
DCLK1	NM_004734	-1.80	-2.11	1.09
DCLK1	NM_004734	-1.52	-1.62	1.38
DEK	NM_003472	-0.04	-1.27	-1.42
DIAPH3	NM_030932	-0.64	-2.12	-2.07
DIAPH3	NM_001042517	-0.57	-2.27	-2.10
DLGAP5	NM_014750	-0.77	-1.97	-1.63
DMD	NM_004019	0.15	-0.79	-1.44
DNAH14	NM_144989	-0.31	-1.44	-1.74
DNAH14	NM_001145154	-0.15	-1.37	-1.56
DNAH7	NM_018897	-0.10	2.62	2.43
DNAJC12	NM_021800	-1.60	-0.59	1.49
DNAJC30	NM_032317	0.04	1.09	0.62
DNALI1	NM_003462	0.73	2.69	3.81
DPP7	NM_013379	-1.21	-0.41	-0.04
DSC2	NM_024422	3.89	1.80	1.16
DSC3	NM_024423	5.40	0.60	0.34
DSC3	NM_024423	3.57	0.48	0.26
DYNLRB2	NM_130897	-1.03	2.01	3.19
E2F2	NM_004091	-0.11	-1.18	-1.02
ECE2	NM_032331	1.00	0.51	-0.36
EFCAB6	NM_022785	0.80	2.77	3.07
EHMT1	NM_024757	-1.13	-0.19	0.01
EIF2C2	NM_012154	0.30	-0.30	-0.81
EIF5A2	NM_020390	-0.25	-1.41	-1.76
ELF1	NM_172373	-0.28	0.82	0.99
ELF1	NM_172373	-0.70	0.92	0.91
ELF5	NM_198381	5.86	1.33	1.98
ELF5	NM_198381	5.00	0.38	1.24
ELMOD2	NM_153702	0.07	1.64	1.07
EME2	AK074080	-1.49	0.03	0.15

EN1	NM_001426	4.66	-1.18	-0.89
EPB41L5	NM_020909	-1.24	-0.38	0.08
EPB41L5	BC032822	0.94	2.77	2.15
EPOR	NM_000121	-1.34	-0.70	-0.17
EPOR	NM_000121	-1.21	-0.49	-0.03
EPS8L1	NM_133180	0.11	2.28	1.56
ERBB3	NM_001982	0.00	0.60	1.27
ERGIC1	NM_001031711	-0.02	1.54	2.16
ERGIC1	NM_001031711	-0.37	1.38	1.77
ERGIC1	NM_001031711	-0.90	1.08	1.14
ERGIC1	NM_001031711	-0.31	1.22	1.67
ESR1	NM_000125	-0.68	1.30	4.41
ETV6	NM_001987	1.56	1.28	0.45
EVL	NM_016337	-0.97	-1.33	0.81
EVL	NM_016337	-0.27	-0.73	1.72
EXD3	NM_017820	-0.58	0.78	0.94
EXOC6	NM_019053	-0.56	0.19	0.71
EXPH5	NM_015065	2.21	2.83	0.92
EXPH5	NM_015065	2.31	3.19	1.27
FAF1	NM_007051	0.35	-0.70	-0.34
FAM120AOS	AK093641	-0.94	-0.13	0.38
FAM123B	NM_152424	0.06	-0.87	-1.18
FAM126A	NM_032581	-0.90	-1.69	-3.25
FAM134B	NM_001034850	0.65	0.98	3.25
FAM134B	NM_001034850	0.81	0.88	3.13
FAM171A1	NM_001010924	1.21	-0.32	-1.01
FAM174A	NM_198507	-0.11	0.41	1.29
FAM174B	NM_207446	-0.44	1.57	1.82
FAM176B	NM_018166	0.17	0.35	1.76
FAM179B	NM_015091	-0.13	1.87	1.45
FAM27E3	BC119675	-0.45	-0.48	-2.14
FAM36A	NM_198076	0.75	-0.01	-0.34
FAM47E	NM_001136570	0.88	2.94	3.49
FAM5B	NM_021165	0.24	1.04	3.53
FAM64A	NM_019013	0.02	-1.77	-1.53
FAM83B	NM_001010872	1.32	-1.35	-0.77
FAM84B	NM_174911	1.17	3.29	2.46
FANCE	NM_021922	-0.04	-1.49	-1.50
FANCI	NM_018193	-0.66	-1.73	-2.02
FBL	NM_001436	-0.04	-1.13	-1.29
FBL	NM_001436	0.08	-0.90	-1.02
FBP1	NM_000507	-1.26	0.49	1.97
FBXL5	NM_033535	0.17	0.88	1.31

FBXO31	AK026130	-0.08	-1.29	-1.17
FECH	NM_001012515	-0.06	0.47	1.40
FFAR2	NM_005306	-0.10	1.18	1.57
FGD3	NM_033086	-0.28	-0.81	1.49
FGFBP1	NM_005130	1.90	-1.14	-3.24
FKBP1A	NM_054014	0.00	-0.41	-1.69
FLJ25694	AK127969	0.78	-0.69	-0.66
FLJ30901	AK056490	-1.54	-1.41	0.56
FLJ38379	AK095698	-0.19	2.55	2.78
FLJ40194	AK097513	1.25	0.51	2.94
FLJ40330	NR_015424	0.58	-0.19	-0.64
FLJ40504	NR_028334	-1.58	-0.03	0.40
FLT3	NM_004119	0.93	0.91	4.22
FNDC3B	BC012204	0.27	0.92	-1.30
FOXA1	NM_004496	-2.78	1.71	3.41
FOXA1	NM_004496	-2.59	1.80	3.58
FOXC1	NM_001453	1.88	-1.41	-0.96
FOXC1	NM_001453	2.03	-1.08	-0.57
FOXL1	NM_005250	1.64	-0.07	0.19
FOXM1	NM_202002	-0.03	-0.95	-1.77
FOXP1	NM_032682	-0.60	0.54	1.28
FRMD6	NM_001042481	-1.27	-1.95	-0.08
FSCN1	NM_003088	-1.21	-2.44	-2.24
FSIP1	NM_152597	-2.06	1.33	2.99
FSTL4	NM_015082	-1.51	-0.94	0.84
FUCA1	NM_000147	0.05	1.37	1.15
FUT8	NM_178154	-0.80	0.20	1.65
FYCO1	NM_024513	-0.08	0.70	1.00
FZD7	NM_003507	1.52	-1.36	-1.11
FZD9	NM_003508	0.04	-1.32	-1.06
GABRP	NM_014211	4.63	0.54	0.34
GALNT10	NM_198321	-1.15	0.82	0.77
GALNT10	AK021777	-0.27	1.34	1.79
GALNT10	NM_198321	-1.62	0.55	0.59
GALNT6	NM_007210	0.23	2.43	3.28
GAMT	NM_000156	-2.23	-1.54	-0.41
GAMT	NM_138924	-1.15	-0.82	-0.01
GATA2	NM_032638	-2.51	-1.24	-0.63
GATA2	NM_032638	-3.10	-1.11	-0.68
GATA3	NM_001002295	-1.54	-1.21	1.83
GCNT2	NM_001491	1.28	-0.22	-1.10
GCSH	NM_004483	-0.36	-1.41	-1.54
GDF15	NM_004864	-4.90	-2.26	-1.19

GEMIN4	NM_015721	0.01	-1.19	-1.05
GEMIN4	NM_015721	-0.11	-1.15	-1.23
GLI3	NM_000168	-1.28	-2.90	0.26
GLOD5	NM_001080489	-1.44	1.16	1.03
GMPS	NM_003875	-0.07	-0.42	-1.23
GNA14	NM_004297	-1.82	1.24	1.44
GNB4	NM_021629	0.36	-1.08	-1.02
GP2	NM_001007240	-0.29	1.26	3.86
GPC1	NM_002081	-0.45	-0.50	0.64
GPD1L	NM_015141	-0.35	1.07	1.11
GPR160	NM_014373	-0.82	1.58	1.97
GPR161	NM_153832	0.34	-0.94	-1.09
GPR68	NM_003485	-2.14	-2.28	0.55
GPRC5C	AK000249	-0.09	2.23	1.65
GPRC5C	NM_022036	-1.53	0.76	0.49
GPRC5C	AK000249	-1.08	2.55	1.44
GPRC5C	NM_022036	-1.52	0.93	0.74
GPRIN2	AB011086	3.96	1.78	1.11
GPSM2	NM_013296	0.65	-0.29	-0.48
GRPEL1	AF070525	-0.87	1.00	0.42
GSDMC	NM_031415	4.14	3.36	1.37
GSG2	AK056691	-0.71	-1.73	-2.06
GSR	BC035691	-2.62	-0.09	-0.84
GTPBP4	NM_012341	0.36	-0.50	-0.78
GUSBP1	NR_027028	-0.73	-0.26	0.63
HAGHL	NM_032304	-0.50	-0.68	0.54
HEATR1	NM_018072	-0.01	-0.95	-1.08
HIF3A	NM_022462	1.12	0.05	-1.97
HIST1H1A	NM_005325	3.70	-0.62	-0.88
HLA-DOB	NM_002120	1.44	0.17	-0.39
HNMT	NM_006895	-0.09	0.36	1.13
HORMAD1	NM_032132	2.75	-1.64	-2.98
HOXB3	NM_002146	-2.65	1.06	0.49
HPDL	NM_032756	-0.70	-2.10	-2.83
HPX	NM_000613	-0.42	1.49	2.62
HSPA1L	NM_005527	-0.37	0.26	1.23
HSPB1	NM_001540	-1.14	-0.43	0.46
HSPB1	NM_001540	-0.87	-0.36	0.50
HSPB1	NM_001540	-0.92	-0.39	0.48
HSPC159	NM_014181	0.48	-0.18	-1.18
HTR7	NM_019859	-1.36	-0.93	0.39
HTR7P	NR_002774	-0.61	-0.19	1.20
IER3	NM_003897	-1.31	-0.55	0.49

IFT88	NM_175605	-0.41	0.20	0.65
IGF2BP3	NM_006547	-2.08	-3.96	-5.22
IL12RB2	NM_001559	3.26	-0.01	0.04
IL13RA1	NM_001560	0.37	0.90	1.50
IL17RD	NM_017563	1.03	-0.34	-1.44
IL6ST	CR621148	-0.87	-0.23	0.63
ILF2	NM_004515	0.86	-0.02	-0.31
INPP4B	BC005273	-0.72	1.40	2.16
IQCD	NM_138451	-0.13	1.02	1.52
ITGB5	NM_002213	-0.74	0.69	0.86
ITGB8	NM_002214	1.51	-0.69	-1.05
ITGB8	NM_002214	1.00	-0.62	-1.48
ITGB8	NM_002214	2.01	-0.08	0.03
ITPR1	NM_002222	-0.93	0.77	0.74
JRKL	NM_003772	0.01	0.48	-1.68
KAZALD1	AK172864	-1.33	-0.89	0.54
KBTBD4	NM_016506	0.62	1.39	1.72
KCNJ11	NM_000525	-0.77	0.05	0.80
KHDC1	NM_030568	1.47	-0.12	0.17
KIAA0232	NM_014743	0.26	1.35	1.35
KIAA0556	NM_015202	-0.18	0.88	0.91
KIAA0564	NM_015058	-0.59	0.61	0.46
KIAA1244	NM_020340	-0.07	2.58	2.67
KIAA1370	NM_019600	-0.33	1.38	1.55
KIAA1370	NM_019600	-0.24	1.63	1.68
KIAA1407	NM_020817	0.54	1.77	1.95
KIAA1683	NM_025249	-1.60	-1.47	-0.28
KIF12	NM_138424	0.12	1.35	3.19
KIF16B	NM_024704	0.49	0.31	1.91
KIF18B	BC048263	-0.88	-2.01	-2.26
KIF1B	NM_183416	0.69	-0.80	-1.19
KIF1B	NM_183416	0.17	-1.01	-1.40
KIF1C	BC040307	0.28	-0.58	-0.75
KIF20A	NM_005733	-0.24	-1.46	-1.09
KIF2C	NM_006845	-0.48	-1.79	-1.75
KIF9	NM_022342	-0.91	0.86	1.19
KIFC1	NM_002263	-0.30	-1.89	-1.59
KIT	NM_000222	2.94	-1.82	-1.28
KITLG	NM_000899	-0.47	2.72	2.28
KLHDC1	NM_172193	-0.13	0.66	1.00
KLHDC9	NM_001007255	0.91	2.49	2.72
KLHL18	BC015962	0.75	-0.54	-0.94
KRR1	NM_007043	-0.70	0.44	0.47

KRT14	NM_000526	1.57	-0.63	-1.87
KRT14	NM_000526	2.93	1.11	1.51
KRT16	NM_005557	3.50	-0.74	-0.99
KRT16	NM_005557	2.02	-1.84	-2.53
KRT16P2	NR_029392	2.25	-1.08	-1.45
KRT16P3	NR_029393	0.69	-0.69	-2.36
KRT17	NM_000422	0.93	-2.79	-2.73
KRT18	NM_000224	-1.46	0.04	0.45
KRT18	NM_000224	-1.31	0.01	0.41
KRT18	L32537	-1.01	0.22	0.54
KRT18	NM_000224	-1.25	0.27	0.64
KRT34	NM_021013	-1.63	-3.40	-3.14
KRT5	NM_000424	4.35	0.81	0.47
KRT6A	NM_005554	4.69	2.64	1.87
KRT6B	NM_005555	6.02	-0.31	0.58
KRT6C	NM_173086	4.45	1.31	1.34
KRTAP6-3	NM_181605	2.12	0.93	1.21
L3MBTL4	NM_173464	2.20	-0.03	-1.54
LAD1	NM_005558	2.63	2.56	0.79
LASP1	NM_006148	0.05	1.21	1.36
LASS2	NM_181746	0.35	0.65	1.38
LASS6	NM_203463	0.23	1.28	1.73
LBR	NM_002296	0.01	-1.60	-1.65
LCA5L	NM_152505	-0.98	1.19	0.98
LCMT2	NM_014793	-0.02	0.32	1.14
LEMD1	NM_001001552	1.63	-0.54	-3.99
LEO1	NM_138792	-0.63	0.06	0.56
LFNG	NM_001040167	-0.62	1.21	2.96
LGALS7	NM_002307	3.04	0.53	0.49
LGALS7B	NM_001042507	3.63	0.63	0.10
LGALS7B	NM_001042507	3.14	0.68	0.68
LIMA1	NM_016357	-1.01	0.03	0.71
LIN9	NM_173083	0.45	-0.46	-0.55
LIN9	NM_173083	0.73	-0.48	-0.35
LMNB2	NM_032737	-0.44	-1.16	-1.44
LMO4	NM_006769	2.49	-0.32	0.92
LOC100128355	XM_002343797	-0.26	-1.06	-1.37
LOC100129034	NR_027406	-0.19	1.66	1.12
LOC100133050	NR_027503	-0.68	-0.20	0.67
LOC100293193	XR_079078	-1.00	-0.99	-2.63
LOC100293193	XR_079078	-1.02	-1.08	-3.12
LOC145837	NR_026979	0.08	3.27	5.18
LOC149134	AK022825	-0.15	-0.19	1.90

LOC149351	BC036441	-0.97	-2.36	-2.98
LOC220429	NR_003268	-0.68	0.70	0.22
LOC254057	AK024653	-2.35	-0.68	-0.03
LOC375295	BC013438	-3.58	-1.31	1.04
LOC375295	BC013438	-2.57	-0.64	1.37
LOC401317	BC087859	0.62	-1.37	-1.00
LOC440335	NR_029454	1.13	3.89	3.69
LOC442249	XR_019231	-1.63	-0.18	0.24
LOC643650	BC033221	1.08	-1.29	-1.19
LOC645195	AK123450	-0.08	-1.30	-1.24
LOC645431	NR_024334	-1.88	-0.07	1.88
LOC645431	NR_024334	0.17	0.43	1.49
LOC646976	AK096082	-1.24	0.00	0.85
LOC729088	CR602569	0.59	-1.03	-0.60
LOC729683	CR594811	0.16	-1.40	-0.72
LOC90246	NR_026954	0.34	1.54	1.83
LPPR2	NM_022737	-1.39	-0.20	0.00
LRBA	NM_006726	-0.04	1.40	1.44
LRFN2	NM_020737	-0.15	2.23	3.07
LRP12	NM_013437	1.03	-0.06	-0.71
LRP12	NM_013437	0.74	-0.09	-1.15
LRRC27	NM_030626	0.25	1.58	1.75
LRRC42	NM_052940	0.09	-0.64	-0.95
LRRC46	NM_033413	-0.01	0.94	1.50
LRRC48	NM_031294	-0.30	0.81	1.85
LRRC4C	NM_020929	0.87	1.53	3.70
LRRC6	NM_012472	0.90	2.81	2.87
LY6K	NM_017527	1.15	-2.08	-1.92
MAGED2	NM_201222	0.74	1.15	2.64
MAGED2	NM_201222	0.86	1.25	2.60
MAGOH	NM_002370	-0.25	-1.83	-1.85
MALAT1	NR_002819	-3.37	-2.92	-1.96
MALAT1	NR_002819	-2.32	-1.72	-0.96
MAN2B2	NM_015274	0.19	1.20	1.37
MAN2B2	NM_015274	0.28	1.30	1.56
MAP7D3	NM_024597	-0.30	-1.31	-1.19
MARVELD2	AK055094	0.98	1.61	2.32
MAST4	NM_198828	-0.04	0.50	2.40
MAST4	NM_001164664	-0.09	0.69	1.38
MBOAT7	NM_024298	-0.81	-0.12	0.73
MCCC2	NM_022132	-0.76	0.54	0.60
MCF2L	AK022184	0.37	0.33	1.97
MCM10	NM_182751	-0.29	-1.42	-1.77

MCM10	NM_182751	-0.22	-1.60	-1.78
MED13L	NM_015335	-0.77	-0.96	0.74
MED30	NM_080651	0.85	0.13	-0.59
MEGF9	NM_001080497	0.87	1.77	2.31
MEIS3	NM_001009813	-1.36	-1.19	-0.03
MEIS3	NM_001009813	-1.74	-1.39	-0.04
MIA	NM_006533	0.66	-4.35	-4.02
MICALL1	NM_033386	0.65	0.13	-1.05
MLLT4	BC014505	1.66	0.57	-0.01
MLPH	NM_024101	-2.19	1.46	2.12
MLPH	NM_024101	-1.63	1.47	2.24
MLPH	NM_001042467	-1.73	0.97	1.82
MMEL1	NM_033467	1.00	2.40	2.23
MOAP1	NM_022151	-0.36	-0.22	0.88
MPP6	NM_016447	-0.94	-1.63	-3.97
MPZL2	NM_144765	1.23	0.84	-1.32
MRPL14	NM_032111	0.86	0.01	-0.33
MRPL2	NM_015950	-0.22	-0.83	-1.58
MRPL37	NM_016491	0.08	-0.83	-1.02
MSH2	NM_000251	-0.18	-0.88	-1.18
MSL1	BC039449	-0.64	0.28	1.66
MSX2	NM_002449	-3.05	-0.06	0.85
MTHFD1L	AY374131	-0.47	-1.45	-1.55
MTHFD1L	NM_015440	-0.83	-1.66	-2.09
MTHFR	NM_005957	-1.12	-0.01	-0.22
MTSS1L	NM_138383	-0.07	-1.14	-1.12
MYO10	NM_012334	-0.02	-0.36	-1.24
MYO19	NM_001033580	-0.87	-0.94	-1.89
N4BP2L2	NM_033111	0.34	1.56	1.71
NAIP	NM_004536	-0.87	0.45	0.91
NANOGP1	AY455283	-1.95	-4.07	-3.90
NAT1	NM_000662	-0.75	1.38	3.13
NAT2	NM_000015	-0.35	2.04	3.20
NAV1	NM_020443	-1.26	-0.58	0.34
NBEA	NM_015678	-1.39	-1.45	0.71
NCAPD2	NM_014865	-0.46	-1.50	-1.68
NDC80	NM_006101	0.04	-1.40	-1.34
NDC80	NM_006101	0.34	-1.42	-1.32
NDFIP1	NM_030571	0.24	0.90	1.26
NEK11	NM_024800	1.03	2.55	2.36
NEK11	NM_145910	1.02	2.22	1.94
NEK9	NM_033116	-0.83	0.42	0.75
NFE2L3	NM_004289	-0.15	-1.46	-2.63

NFE2L3	NM_004289	0.20	-1.50	-2.08
NFIL3	NM_005384	0.63	-0.85	-0.77
NME3	NM_002513	-0.30	0.92	1.33
NMT2	NM_004808	-0.05	-1.68	-1.25
NOL3	NM_003946	-1.24	-1.10	0.02
NPC1L1	NM_013389	-2.00	-1.97	-0.08
NPDC1	NM_015392	-1.05	0.14	1.11
NPR3	NM_000908	1.77	-1.85	-1.76
NPR3	NM_000908	1.65	-1.85	-1.70
NR1D1	NM_021724	-1.39	-0.54	0.48
NR1D1	NM_021724	-1.15	-0.60	0.22
NR2E3	NM_014249	-0.89	0.09	1.89
NRG2	NM_004883	1.23	-0.29	-0.60
NRG2	NM_013982	1.54	-1.59	-1.17
NRTN	NM_004558	0.60	-1.68	-1.70
NT5DC2	NM_022908	-0.76	-2.09	-2.39
NTN1	NM_004822	2.29	1.90	0.67
NUCB2	AK097398	-1.12	-0.33	1.00
NUCB2	NM_005013	-0.57	0.07	1.13
NUDCD1	NM_032869	0.71	0.07	-0.39
NUDT16	NM_152395	0.15	1.19	1.01
NUDT4	NM_199040	-0.42	0.68	0.85
NUDT4	NM_199040	-0.40	0.67	0.78
NUDT4	NM_199040	-0.39	0.77	0.78
NUDT4	NM_199040	-0.88	0.70	0.66
NUDT5	NM_014142	0.56	-0.35	-0.62
NXPH3	NM_007225	0.72	0.23	2.14
OBFC1	NM_024928	-0.25	0.42	1.04
OBSCN	NM_052843	0.54	-0.93	-0.70
OGFRL1	NM_024576	2.15	0.42	0.06
OIP5	NM_007280	-0.86	-2.20	-2.10
OLFM4	NM_006418	6.96	1.89	1.91
OSBPL3	NM_015550	0.21	-0.63	-2.19
OSBPL3	NM_015550	0.08	-1.51	-2.39
OSBPL3	NM_015550	0.36	-0.73	-1.47
OSR1	NM_145260	0.88	-0.22	-1.13
OVGP1	NM_002557	-0.40	1.70	0.64
P2RX4	NM_002560	0.29	1.14	1.87
P4HTM	NM_177938	-0.18	0.92	1.42
P4HTM	NM_177938	-0.15	0.73	1.13
PAAF1	NM_025155	-0.90	0.13	0.03
PAFAH2	NM_000437	0.06	1.34	1.09
PARP9	NM_031458	2.01	3.57	3.24

PAX9	NM_006194	-1.88	1.81	0.53
PBLD	NM_022129	0.04	1.09	1.45
PBLD	NM_022129	-0.16	1.26	1.56
PCDH1	NM_032420	0.28	1.33	1.79
PCSK4	NM_017573	-1.76	-0.91	0.15
PDE7A	NM_002603	0.52	-0.87	-0.74
PELI1	NM_020651	1.60	1.20	0.25
PGAP3	NM_033419	0.14	1.63	2.68
PGM1	NM_002633	0.18	-0.64	-0.94
PGPEP1	NM_017712	-0.49	0.56	0.30
PHGDH	NM_006623	0.52	-1.71	-1.66
PIM1	NM_002648	-0.92	-2.35	-2.37
PIP	NM_002652	1.96	7.24	7.50
PLCD4	NM_032726	-2.82	-1.94	-0.10
PLEKHF2	NM_024613	-0.07	1.10	2.07
PLEKHG4B	NM_052909	2.14	-0.51	-0.77
PLEKHG4B	NM_052909	1.51	-0.39	-0.16
PLIN5	NM_001013706	-0.23	-0.60	0.96
PM20D2	NM_001010853	0.55	-1.33	-2.64
PNPLA4	NM_004650	-1.07	1.04	2.04
POLD4	NM_021173	0.30	1.12	1.36
POLD4	NM_021173	-0.17	1.01	1.10
POLH	NM_006502	0.72	-0.43	-0.81
POLK	NM_016218	-1.85	-1.28	-0.67
POLK	NM_016218	-1.63	-0.84	-0.38
POLR2F	NM_021974	0.24	-0.11	-0.92
POTEC	NM_001137671	-1.83	-0.64	2.26
POU4F1	NM_006237	0.32	-2.44	-3.65
PPP1CB	NM_002709	1.11	0.10	0.42
PPP1R3C	NM_005398	-2.40	-2.18	0.30
PPP2R3A	NM_002718	1.13	1.52	-0.42
PPP2R3A	NM_002718	0.93	1.27	-0.36
PPP2R5D	NM_180976	0.28	-0.73	-0.64
PPPDE1	BC020640	-0.54	-1.43	-2.60
PPPDE1	NM_016076	1.07	0.24	-0.09
PRDM13	NM_021620	0.29	-4.25	-4.09
PRDM6	NM_001136239	-0.30	-0.40	1.81
PRICKLE1	NM_153026	1.84	-0.23	-0.24
PROM1	NM_006017	2.80	-0.78	-2.92
PRPF38A	NM_032864	-0.13	-1.17	-0.81
PRR13	NM_001005354	-0.55	0.67	0.49
PRR15	NM_175887	-0.90	2.59	4.06
PRR15	NM_175887	-0.46	1.32	2.31

PRRT2	NM_145239	-1.38	-2.14	0.33
PSAT1	NM_058179	-1.00	-1.31	-2.84
PSMG1	NM_003720	-0.30	-1.21	-1.38
PTK7	NM_002821	0.78	0.36	-0.76
PTPLA	NM_014241	-0.25	-2.78	-2.34
PTPN14	NM_005401	0.61	-0.94	-1.08
PTPN14	NM_005401	1.28	0.22	-1.09
PTTG1	NM_004219	-0.42	-1.47	-1.54
QKI	NM_006775	-0.04	-1.14	-1.71
RAB27B	NM_004163	-0.62	2.62	1.83
RABEP1	NM_004703	-0.33	0.22	1.68
RABEP1	NM_004703	-0.59	0.36	1.61
RAD51AP1	NM_006479	-0.28	-0.44	-1.67
RAD54L	NM_003579	-0.82	-1.95	-2.11
RALGPS2	NM_152663	-1.66	0.56	0.70
RAP2C	NM_021183	0.44	1.68	1.33
RAPGEF3	NM_006105	-0.87	0.90	0.68
RARRES1	NM_002888	5.86	2.77	0.29
RASD2	NM_014310	1.65	0.02	-0.21
RASL11B	NM_023940	-0.23	-0.80	1.57
RBM47	NM_019027	0.27	2.20	1.62
RBM47	NM_019027	0.91	3.36	2.79
RDH10	NM_172037	2.71	2.15	0.38
RDH10	NM_172037	2.22	1.49	-0.21
REPS2	NM_004726	0.56	0.84	2.28
RET	NM_020630	-0.93	0.48	2.14
RET	NM_020975	-2.17	0.94	2.72
REXO2	NM_015523	-0.54	-0.67	-1.57
REXO2	NM_015523	-0.30	-0.44	-1.37
RGMA	NM_020211	2.45	0.47	0.03
RGS11	AK294448	-1.50	-1.10	0.72
RGS11	NM_003834	-0.58	-0.20	1.76
RHBG	NM_020407	-2.50	-2.27	-1.27
RHOC	NM_175744	-0.29	0.53	0.83
RHOH	NM_004310	-2.37	-0.94	0.07
RIOK1	NM_153005	0.65	-0.37	-0.44
RNASEL	NM_021133	-0.21	1.40	1.67
RND1	NM_014470	-1.32	0.54	0.15
RNF103	NM_005667	0.93	2.00	2.06
ROPN1	NM_017578	2.33	-4.40	-4.94
ROPN1B	NM_001012337	2.22	-3.96	-4.70
RPS27A	NM_002954	0.16	-0.58	-0.97
RSPH1	NM_080860	0.98	2.61	3.38

RUNDC1	NM_173079	0.23	1.73	1.26
RXRA	AK090416	-0.77	-0.28	0.45
SAA1	NM_000331	3.85	1.75	0.16
SAA2	NM_030754	3.50	1.93	0.68
SAR1B	NM_001033503	-0.49	0.05	0.91
SDSL	NM_138432	-0.22	1.08	1.13
SEC16A	NM_014866	-0.04	1.66	1.56
SEH1L	NM_031216	0.49	-0.36	-0.74
SEH1L	NM_031216	0.10	-0.54	-1.36
SELENBP1	NM_003944	-0.38	1.77	1.63
SEPHS1	BC064610	0.32	-1.01	-0.54
SEPHS2	NM_012248	-0.66	0.36	0.81
SERBP1	NM_001018067	-0.13	-1.02	-1.19
SERF2	NM_001018108	0.36	0.87	1.37
SETD1B	NM_015048	-0.30	0.34	0.91
SFRP1	NM_003012	-0.37	-3.91	-3.97
SFRP1	NM_003012	0.10	-4.23	-4.41
SFRS13B	NM_080743	1.20	-0.59	-2.51
SFXN5	NM_144579	-0.87	-0.47	0.52
SFXN5	NM_144579	-0.31	0.04	0.79
SH2D2A	NM_003975	-0.29	-1.33	-1.76
SH3BP4	NM_014521	-0.13	1.38	1.49
SIDT1	NM_017699	-2.01	1.76	2.58
SIN3B	BC025026	0.84	-0.06	-0.89
SKA3	BC013418	-0.38	-1.30	-1.62
SKP2	NM_032637	-1.32	-2.43	-2.53
SLC19A3	NM_025243	0.31	-1.82	-2.25
SLC22A18	NM_183233	-1.27	-0.26	0.15
SLC22A5	NM_003060	-0.04	0.44	1.28
SLC25A37	AF495725	0.37	-0.79	-1.56
SLC25A37	AF113696	-1.18	-2.47	-2.78
SLC27A2	NM_003645	-3.43	-2.16	-1.06
SLC27A6	NM_001017372	0.51	-1.96	-2.95
SLC2A10	NM_030777	0.04	2.15	2.65
SLC34A2	NM_006424	4.30	4.00	0.51
SLC39A6	NM_012319	-1.15	-0.86	1.61
SLC39A6	NM_012319	-1.01	-0.88	1.66
SLC40A1	NM_014585	0.17	3.01	2.37
SLC44A4	NM_025257	-1.24	4.30	4.36
SLC44A4	NM_025257	-1.16	3.76	3.87
SLC4A8	NM_004858	0.44	1.22	2.34
SLC4A8	NM_004858	-1.19	-0.38	0.78
SLC6A2	NM_001172504	1.77	0.48	0.15

SLC6A2	NM_001043	2.02	-0.46	0.00
SLC7A8	NM_182728	0.18	1.18	1.90
SLPI	NM_003064	1.53	2.52	-1.57
SLPI	NM_003064	1.51	2.63	-1.97
SMPD3	NM_018667	-1.32	-1.46	1.85
SNAP29	NM_004782	-1.05	0.46	0.08
SNX25	NM_031953	-0.87	0.23	0.06
SNX9	NM_016224	-0.04	0.62	1.14
SOHLH2	NM_017826	-1.24	-2.83	-3.80
SORD	NM_003104	-0.64	0.35	0.96
SORD	NM_003104	-0.42	0.32	1.20
SOSTDC1	NM_015464	3.16	-1.37	-2.56
SOX10	NM_006941	-0.89	-3.02	-2.55
SPAG17	NM_206996	1.08	3.37	3.19
SPATA20	NM_022827	-1.04	-0.83	0.66
SPC25	NM_020675	-1.34	-2.59	-2.42
SPDEF	NM_012391	0.09	2.20	3.60
SPEF2	NM_024867	0.64	2.93	2.94
SPEG	AK055387	1.20	-0.92	-0.22
SPG11	NM_025137	0.22	0.79	1.37
SPOP	NM_001007226	-0.45	-0.11	1.29
SPOP	NM_001007226	-0.29	0.14	1.36
SPRED2	NM_181784	-0.42	1.83	1.00
SPSB1	NM_025106	0.97	0.79	-0.14
SPSB1	NM_025106	1.00	1.04	-0.38
SRF	NM_003131	1.04	0.36	0.04
SRPK1	NM_003137	0.08	-0.77	-1.21
SSBP2		-2.13	-2.00	0.53
SSH3	NM_017857	-0.36	1.00	1.06
SSH3	NM_017857	-0.32	1.06	1.01
ST3GAL5	NM_003896	-1.04	1.06	0.35
ST3GAL5	NM_003896	-1.57	0.95	-0.04
STAC	NM_003149	1.65	-1.40	-2.17
STAC	NM_003149	1.69	-1.90	-3.16
STARD10	NM_006645	0.23	1.33	2.50
STMN1	NM_203401	0.05	-1.62	-0.83
STRA8	NM_182489	2.71	0.79	0.45
STRN3	NM_014574	-1.31	0.52	0.09
STRN3	NM_014574	-0.77	0.82	0.73
SUOX	NM_000456	0.08	1.41	1.15
SUV39H2	NM_024670	0.09	-0.84	-1.35
SYT9	NM_175733	0.90	0.92	2.89
SYTL2	NM_032943	-2.08	-0.64	0.35

TADA2B	NM_152293	-0.29	1.22	1.34
TADA2B	NM_152293	-0.10	0.92	0.85
TAGAP	NM_138810	2.32	0.29	0.52
TANC2	NM_025185	-1.14	0.07	0.97
TANC2	AK021886	-1.17	-0.07	0.62
TBC1D9	NM_015130	-0.89	1.50	3.93
TBPL1	NM_004865	0.08	-0.42	-0.97
TBX19	NM_005149	1.29	0.15	-0.04
TCEAL1	NM_001006640	-0.07	0.87	1.69
TCEAL3	NM_001006933	-0.43	0.13	0.88
TCEAL4	NM_024863	0.38	0.53	1.56
TCEAL6	NM_001006938	-0.39	0.20	0.90
TCF7L1	NM_031283	1.05	-2.47	-1.92
TCTN1	NM_024549	-0.24	0.19	0.96
TCTN1	BC030993	-2.39	-2.15	-0.80
TEAD4	NM_003213	0.16	-0.19	-1.12
TEX10	NM_017746	-0.31	-0.78	-1.40
TFCP2L1	NM_014553	6.03	3.88	2.83
TFF1	NM_003225	-2.71	-2.24	1.37
TFF1	NM_003225	-4.16	-3.26	1.33
TFF3	NM_003226	-1.16	2.44	5.61
TFF3	NM_003226	-0.84	2.23	3.55
TFF3	NM_003226	-2.37	2.34	5.25
TFF3	NM_003226	-1.89	2.54	5.33
THPO	NM_000460	-1.79	-2.26	-0.54
THSD4	NM_024817	-1.42	0.79	2.42
TIFA	NM_052864	1.12	0.27	0.05
TLE3	NM_005078	-0.21	0.55	0.83
TM7SF2	NM_003273	-1.64	0.20	-0.04
TMBIM6	NM_003217	0.13	0.97	1.46
TMC4	NM_144686	0.71	2.10	2.50
TMC5	NM_024780	-1.57	4.14	2.62
TMEM121	NM_025268	-1.07	-1.68	0.27
TMEM123	NM_052932	0.23	0.00	-1.07
TMEM123	NM_052932	0.45	0.06	-0.94
TMEM141	NM_032928	-0.60	0.54	0.30
TMEM192	NM_001100389	-1.16	0.65	-0.17
TMEM192	NM_001100389	-0.99	0.80	0.03
TMEM38A	NM_024074	0.51	-0.44	-0.91
TMEM45B	NM_138788	-3.86	1.25	-1.01
TMEM50B	NM_006134	0.43	1.08	1.72
TMEM62	NM_024956	0.59	2.23	2.13
TMEM65	NM_194291	1.16	0.29	-0.34

TMEM74	NM_153015	0.28	-1.37	-2.34
TMEM86A	NM_153347	0.75	3.10	2.54
TMEM87B	NM_032824	0.75	1.75	1.87
TMEM87B	NM_032824	0.05	1.86	1.77
TMSB15B	NM_194324	1.60	-2.29	-0.63
TNNI2	NM_003282	-1.15	-3.25	-2.85
TOP1MT	NM_052963	0.50	-0.37	-0.97
TP53I11	NM_001076787	-0.31	1.77	1.20
TPCN1	NM_001143819	-1.18	-0.05	-0.09
TPCN1	NM_001143819	-1.11	0.19	0.17
TPX2	NM_012112	-0.19	-1.35	-1.33
TRAF3IP1	NM_015650	-0.53	0.87	0.25
TRDMT1	NM_004412	0.83	-0.85	-1.27
TRDMT1	NM_004412	0.67	-1.44	-1.53
TRIM29	NM_012101	0.89	-1.47	-2.77
TSC22D1	NM_183422	0.10	1.52	1.17
TSC22D3	NM_004089	1.32	2.62	2.43
TSLP	NM_033035	-1.99	-4.78	-4.84
TSNAXIP1	NM_018430	-0.20	0.27	1.64
TSPAN1	NM_005727	0.60	2.58	3.79
TTC12	NM_017868	0.29	1.81	1.25
TTC8	NM_144596	-1.45	-0.90	-0.11
TTK	NM_003318	0.03	-0.84	-1.72
TTLL4	NM_014640	0.69	-0.57	-1.11
TYMS	NM_001071	-0.70	-2.24	-2.15
UBASH3B	NM_032873	0.84	-0.37	-1.36
UBE2E3	NM_006357	0.57	-0.55	-1.17
UBE2E3	NM_006357	0.47	-0.80	-1.78
UBR3	NM_172070	-0.36	0.81	0.44
UBXN10	BX648631	-1.52	1.14	0.67
UCK2	NM_012474	0.50	-0.76	-0.70
UEVLD	NM_001040697	-0.21	0.50	1.04
UGT8	NM_003360	3.21	0.14	-0.26
UGT8	U62899	3.03	0.09	-0.45
UGT8	AL137342	3.35	0.31	-0.68
ULK1	NM_003565	-0.64	-0.18	0.37
UPRT	NM_145052	0.02	1.18	1.19
URB2	NM_014777	0.82	0.02	-0.40
USP1	NM_003368	0.47	-1.00	-0.61
USP31	NM_020718	0.57	-0.57	-0.37
USP6NL	NM_014688	0.98	0.33	-0.36
VEZT	NM_017599	0.19	1.07	1.28
VGLL1	NM_016267	4.07	2.26	0.29

VRK2	NM_006296	1.31	0.79	0.19
WDR52	NM_001164496	0.29	1.83	1.38
WFS1	NM_006005	-0.35	0.74	0.84
WNT11	NM_004626	0.35	-2.58	-1.61
WNT6	NM_006522	1.22	-1.96	-2.42
WWP1	NM_007013	-0.45	0.66	1.34
WWTR1	NM_015472	0.57	0.26	-1.55
WWTR1	NM_015472	0.55	0.77	-2.13
XBP1	NM_005080	0.00	1.73	3.38
XBP1	NM_001079539	0.22	2.51	3.53
XPO5	NM_020750	0.75	-0.60	-0.49
YBX1	NM_004559	-0.01	-1.00	-1.03
YBX1	NM_004559	-0.22	-1.34	-1.37
YBX1	NM_004559	-0.27	-1.36	-1.43
YBX1	NM_004559	-0.05	-1.11	-1.07
YPEL2	NM_001005404	0.83	1.43	2.15
ZCCHC11	NM_001009881	-0.42	-1.92	-1.96
ZMAT1	NM_032441	1.17	2.09	2.97
ZMYND10	NM_015896	-0.72	0.35	1.21
ZNF136	NM_003437	-1.05	-0.02	0.03
ZNF217	NM_006526	-0.31	0.52	0.89
ZNF232	NM_014519	0.74	-0.34	-0.12
ZNF238	NM_006352	1.37	2.39	-0.29
ZNF238	NM_006352	0.84	1.11	-1.01
ZNF281	NM_012482	-0.48	0.36	0.61
ZNF286A	AF086305	0.00	-2.00	-2.26
ZNF304	NM_020657	0.16	1.40	1.01
ZNF318	NM_014345	0.91	-0.33	-0.21
ZNF462	NM_021224	1.44	-1.05	-0.78
ZNF467	BC038972	-0.63	1.35	1.25
ZNF507	NM_014910	0.52	-0.20	-0.56
ZNF597	NM_152457	-1.39	1.17	0.84
ZSWIM5	NM_020883	-1.93	-1.92	-0.44
ZW10	NM_004724	-0.92	-0.87	-2.00
