# Supplementary material for: Integrated Genomic and Epigenomic Analysis of Breast Cancer Brain Metastasis
Source: PLoS One. 2014 Jan 29;9(1):e85448. doi: 10.1371/journal.pone.0085448 (PMC3906004; doi:10.1371/journal.pone.0085448)
Supplement: File S1 — Supporting figures and tables. Figure S1: Combined Network for Upstream Analysis of FOXM1 and TBX2. The downstream genes connected to FOXM1 and TBX2 were illustrated as a network in IPA. The mRNA expression ratios are listed below the gene nodes. The legend within figure describes the node and edge color keys. Figure S2: Word Cloud Analysis of Cluster Enrichments. We have used word clouds to visually summarize the textual results from the enrichment analysis of each gene cluster as observed in Figure 3. The results were generated using www.wordle.net web resource. The larger the word, the more times it is mentioned in the enrichment categories. Supplementary Tables in File S1. Table S1a. Table S1b. Table S2. Table S3a. Table S3b. Table S4a. Figure S1. Table S4b. Table S5a–b. Table S6a–b. Table S7. Table S8a–f. Table S9a–f. Figure S2. Table S10. Table S11a–c. Table S11d. Table S12. Table S13. Table S14. (ZIP) [file pone.0085448.s001.zip › Supplementary Table S8a.pdf]

Supplementary Table 8a. List of Cluster 1 genes on heatmap

(See Figure 3 in main text). Values represent normalized Log2 ratios.

Gene Symbol	GenBank Accession	Basal-like	Her2+/ER-ve	Luminal B	ProbeName
FSIP1	NM_152597	-2.06	1.33	2.99	A_23_P353125
RET	NM_020975	-2.17	0.94	2.72	A_23_P202245
MAGED2	NM_201222	0.74	1.15	2.64	A_23_P33894
MAGED2	NM_201222	0.86	1.25	2.60	A_24_P160263
CA12	NM_001218	-2.07	0.04	2.57	A_24_P330518
TMC4	NM_144686	0.71	2.10	2.50	A_23_P330461
C11orf52	NM_080659	1.51	3.19	2.43	A_23_P1722
TSC22D3	NM_004089	1.32	2.62	2.43	A_23_P217688
THSD4	NM_024817	-1.42	0.79	2.42	A_23_P148249
NEK11	NM_024800	1.03	2.55	2.36	A_23_P211973
SLC4A8	NM_004858	0.44	1.22	2.34	A_23_P72912
MARVELD2	AK055094	0.98	1.61	2.32	A_23_P401675
MEGF9	NM_001080497	0.87	1.77	2.31	A_32_P129894
REPS2	NM_004726	0.56	0.84	2.28	A_32_P100109
POTEC	NM_001137671	-1.83	-0.64	2.26	A_23_P56855
MMEL1	NM_033467	1.00	2.40	2.23	A_23_P138294
CA12	NM_001218	-2.11	-0.20	2.23	A_23_P372234
ERGIC1	NM_001031711	-0.02	1.54	2.16	A_23_P333218
YPEL2	NM_001005404	0.83	1.43	2.15	A_24_P787947
RET	NM_020630	-0.93	0.48	2.14	A_24_P343695
C16orf71	NM_139170	0.16	1.92	2.14	A_23_P414281
NXPH3	NM_007225	0.72	0.23	2.14	A_24_P940086
TMEM62	NM_024956	0.59	2.23	2.13	A_23_P49041
RNF103	NM_005667	0.93	2.00	2.06	A_23_P56709
APBB2	NM_004307	0.59	0.41	2.00	A_24_P234701
MCF2L	AK022184	0.37	0.33	1.97	A_23_P99496
GPR160	NM_014373	-0.82	1.58	1.97	A_23_P167005
KIAA1407	NM_020817	0.54	1.77	1.95	A_23_P419213
NEK11	NM_145910	1.02	2.22	1.94	A_23_P155301
CCDC96	NM_153376	0.20	1.60	1.92	A_32_P69930
KIF16B	NM_024704	0.49	0.31	1.91	A_23_P17503
SLC7A8	NM_182728	0.18	1.18	1.90	A_23_P205489
NR2E3	NM_014249	-0.89	0.09	1.89	A_23_P205867
P2RX4	NM_002560	0.29	1.14	1.87	A_23_P53623
C9orf98	NM_152572	0.15	1.27	1.87	A_23_P83200
TMEM87B	NM_032824	0.75	1.75	1.87	A_23_P91076
LRRC48	NM_031294	-0.30	0.81	1.85	A_23_P255701

APBB2	NM_173075	0.68	0.20	1.84	A_23_P10701
LOC90246	NR_026954	0.34	1.54	1.83	A_24_P532180
FAM174B	NM_207446	-0.44	1.57	1.82	A_23_P100001
PCDH1	NM_032420	0.28	1.33	1.79	A_24_P234838
GALNT10	AK021777	-0.27	1.34	1.79	A_23_P19102
TMEM87B	NM_032824	0.05	1.86	1.77	A_23_P303155
ERGIC1	NM_001031711	-0.37	1.38	1.77	A_24_P89257
FAM176B	NM_018166	0.17	0.35	1.76	A_23_P62831
C14orf45	NM_025057	0.36	1.20	1.76	A_23_P76983
LRRC27	NM_030626	0.25	1.58	1.75	A_32_P186157
LASS6	NM_203463	0.23	1.28	1.73	A_24_P289366
KBTBD4	NM_016506	0.62	1.39	1.72	A_23_P12950
TMEM50B	NM_006134	0.43	1.08	1.72	A_24_P305623
N4BP2L2	NM_033111	0.34	1.56	1.71	A_23_P65262
CYB5R1	NM_016243	0.50	1.70	1.71	A_23_P52101
TCEAL1	NM_001006640	-0.07	0.87	1.69	A_23_P73801
ATG16L1	NM_030803	0.35	1.50	1.69	A_32_P113508
RABEP1	NM_004703	-0.33	0.22	1.68	A_24_P945147
KIAA1370	NM_019600	-0.24	1.63	1.68	A_24_P357576
ANXA9	NM_003568	-2.08	0.10	1.67	A_23_P103617
ATP8B1	NM_005603	0.00	1.39	1.67	A_23_P107597
RNASEL	NM_021133	-0.21	1.40	1.67	A_23_P390172
ERGIC1	NM_001031711	-0.31	1.22	1.67	A_23_P404871
SELENBP1	NM_003944	-0.38	1.77	1.63	A_23_P74619
RBM47	NM_019027	0.27	2.20	1.62	A_24_P226108
RABEP1	NM_004703	-0.59	0.36	1.61	A_24_P399174
ATP6AP1	NM_001183	0.49	0.50	1.57	A_23_P250462
FFAR2	NM_005306	-0.10	1.18	1.57	A_23_P397391
PBLD	NM_022129	-0.16	1.26	1.56	A_24_P112395
MAN2B2	NM_015274	0.28	1.30	1.56	A_23_P250379
TCEAL4	NM_024863	0.38	0.53	1.56	A_23_P259166
SEC16A	NM_014866	-0.04	1.66	1.56	A_23_P251303
EPS8L1	NM_133180	0.11	2.28	1.56	A_23_P208779
KIAA1370	NM_019600	-0.33	1.38	1.55	A_23_P99853
IQCD	NM_138451	-0.13	1.02	1.52	A_24_P390060
C4orf34	NM_174921	-0.50	1.47	1.51	A_23_P112634
IL13RA1	NM_001560	0.37	0.90	1.50	A_24_P280113
LRRC46	NM_033413	-0.01	0.94	1.50	A_23_P152949
SH3BP4	NM_014521	-0.13	1.38	1.49	A_23_P79259
LOC645431	NR_024334	0.17	0.43	1.49	A_24_P109766
BCAS4	NM_001010974	-0.80	1.33	1.47	A_24_P143492
TMBIM6	NM_003217	0.13	0.97	1.46	A_24_P355876
FAM179B	NM_015091	-0.13	1.87	1.45	A_23_P3102

PBLD	NM_022129	0.04	1.09	1.45	A_23_P149998
CCDC125	NM_176816	-0.29	0.79	1.44	A_23_P432591
LRBA	NM_006726	-0.04	1.40	1.44	A_24_P360078
P4HTM	NM_177938	-0.18	0.92	1.42	A_23_P113317
FECH	NM_001012515	-0.06	0.47	1.40	A_32_P151933
MAST4	NM_001164664	-0.09	0.69	1.38	A_23_P110571
CHST15	NM_015892	-0.58	1.24	1.38	A_23_P383986
WDR52	NM_001164496	0.29	1.83	1.38	A_23_P110090
LASS2	NM_181746	0.35	0.65	1.38	A_23_P63010
CIRBP	NM_001280	-0.20	1.06	1.37	A_23_P142322
SPG11	NM_025137	0.22	0.79	1.37	A_23_P65699
MAN2B2	NM_015274	0.19	1.20	1.37	A_23_P250380
SERF2	NM_001018108	0.36	0.87	1.37	A_24_P313334
POLD4	NM_021173	0.30	1.12	1.36	A_23_P360215
LASP1	NM_006148	0.05	1.21	1.36	A_23_P89187
KIAA0232	NM_014743	0.26	1.35	1.35	A_23_P327069
WWP1	NM_007013	-0.45	0.66	1.34	A_23_P146990
TADA2B	NM_152293	-0.29	1.22	1.34	A_24_P687582
RAP2C	NM_021183	0.44	1.68	1.33	A_23_P147826
NME3	NM_002513	-0.30	0.92	1.33	A_23_P152115
BAG3	NM_004281	0.37	1.58	1.32	A_23_P47077
FBXL5	NM_033535	0.17	0.88	1.31	A_23_P213247
CDK17	NM_002595	-0.13	1.60	1.31	A_23_P33376
ANKRD42	NM_182603	-0.78	1.68	1.30	A_32_P69166
DALRD3	NM_018114	0.00	1.06	1.30	A_23_P135611
CCDC24	NM_152499	0.05	0.73	1.29	A_24_P310864
C6orf1	NM_178508	0.15	0.68	1.29	A_23_P411379
FAM174A	NM_198507	-0.11	0.41	1.29	A_23_P30283
SLC22A5	NM_003060	-0.04	0.44	1.28	A_24_P174755
ANKRD42	NM_182603	-0.89	1.44	1.28	A_24_P357572
VEZT	NM_017599	0.19	1.07	1.28	A_23_P204609
ERBB3	NM_001982	0.00	0.60	1.27	A_23_P349416
RUNDC1	NM_173079	0.23	1.73	1.26	A_24_P395621
NDFIP1	NM_030571	0.24	0.90	1.26	A_23_P81247
ZNF467	BC038972	-0.63	1.35	1.25	A_23_P59470
TTC12	NM_017868	0.29	1.81	1.25	A_24_P73075
HSPA1L	NM_005527	-0.37	0.26	1.23	A_23_P70547
ZMYND10	NM_015896	-0.72	0.35	1.21	A_23_P29663
SORD	NM_003104	-0.42	0.32	1.20	A_32_P89691
TP53I11	NM_001076787	-0.31	1.77	1.20	A_24_P160969
UPRT	NM_145052	0.02	1.18	1.19	A_23_P159865
KIF9	NM_022342	-0.91	0.86	1.19	A_24_P225878
TSC22D1	NM_183422	0.10	1.52	1.17	A_23_P162739

C6orf1	NM_178508	-0.15	0.38	1.17	A_23_P81993
CPEB3	NM_014912	-0.11	1.63	1.16	A_23_P46813
FUCA1	NM_000147	0.05	1.37	1.15	A_23_P11543
SUOX	NM_000456	0.08	1.41	1.15	A_23_P150857
LCMT2	NM_014793	-0.02	0.32	1.14	A_23_P106505
SDSL	NM_138432	-0.22	1.08	1.13	A_23_P53439
P4HTM	NM_177938	-0.15	0.73	1.13	A_23_P113311
LOC100129034	NR_027406	-0.19	1.66	1.12	A_32_P91042
GPD1L	NM_015141	-0.35	1.07	1.11	A_23_P318284
POLD4	NM_021173	-0.17	1.01	1.10	A_23_P127367
PAFAH2	NM_000437	0.06	1.34	1.09	A_24_P71153
ELMOD2	NM_153702	0.07	1.64	1.07	A_23_P305692
CCDC159	NM_001080503	-0.10	1.02	1.07	A_24_P75920
ANKRA2	NM_023039	-0.10	0.85	1.06	A_23_P41634
ARHGEF16	NM_014448	-0.17	1.10	1.05	A_23_P114670
ARFIP1	NM_001025595	0.42	1.57	1.04	A_24_P166094
NUDT16	NM_152395	0.15	1.19	1.01	A_23_P310560
ZNF304	NM_020657	0.16	1.40	1.01	A_24_P228266
SPRED2	NM_181784	-0.42	1.83	1.00	A_32_P225854
FYCO1	NM_024513	-0.08	0.70	1.00	A_23_P212339
COG3	NM_031431	0.36	1.37	1.00	A_24_P296070
ELF1	NM_172373	-0.28	0.82	0.99	A_23_P2801
C12orf72	NM_173802	-0.20	1.25	0.99	A_23_P404120
SORD	NM_003104	-0.64	0.35	0.96	A_32_P127153
SETD1B	NM_015048	-0.30	0.34	0.91	A_23_P319895
ARFIP2	NM_012402	-0.21	0.67	0.90	A_23_P139228
C5orf44	NM_024941	-0.27	0.47	0.88	A_23_P121825
TADA2B	NM_152293	-0.10	0.92	0.85	A_23_P396541
WFS1	NM_006005	-0.35	0.74	0.84	A_23_P121499
RHOC	NM_175744	-0.29	0.53	0.83	A_23_P12514
TLE3	NM_005078	-0.21	0.55	0.83	A_23_P342934
ANKRA2	NM_023039	-0.45	0.61	0.81	A_23_P159012
OVGP1	NM_002557	-0.40	1.70	0.64	A_23_P103756
DNAJC30	NM_032317	0.04	1.09	0.62	A_23_P157170
ARRDC1	NM_152285	-0.11	0.94	0.56	A_23_P391607
