# Supplementary material for: Integrated Genomic and Epigenomic Analysis of Breast Cancer Brain Metastasis
Source: PLoS One. 2014 Jan 29;9(1):e85448. doi: 10.1371/journal.pone.0085448 (PMC3906004; doi:10.1371/journal.pone.0085448)
Supplement: File S1 — Supporting figures and tables. Figure S1: Combined Network for Upstream Analysis of FOXM1 and TBX2. The downstream genes connected to FOXM1 and TBX2 were illustrated as a network in IPA. The mRNA expression ratios are listed below the gene nodes. The legend within figure describes the node and edge color keys. Figure S2: Word Cloud Analysis of Cluster Enrichments. We have used word clouds to visually summarize the textual results from the enrichment analysis of each gene cluster as observed in Figure 3. The results were generated using www.wordle.net web resource. The larger the word, the more times it is mentioned in the enrichment categories. Supplementary Tables in File S1. Table S1a. Table S1b. Table S2. Table S3a. Table S3b. Table S4a. Figure S1. Table S4b. Table S5a–b. Table S6a–b. Table S7. Table S8a–f. Table S9a–f. Figure S2. Table S10. Table S11a–c. Table S11d. Table S12. Table S13. Table S14. (ZIP) [file pone.0085448.s001.zip › Supplementary Table S8b.pdf]

Supplementary Table 8b. List of Cluster 2 genes on heatmap

(See Figure 3 in main text). Values represent normalized Log2 ratios.

Gene Symbol	GenBank Accession	Basal-like	Her2+/ER-ve	Luminal B	ProbeName
LRRC4C	NM_020929	0.87	1.53	3.70	A_23_P24457
SPDEF	NM_012391	0.09	2.20	3.60	A_23_P111194
XBP1	NM_001079539	0.22	2.51	3.53	A_24_P100228
FAM5B	NM_021165	0.24	1.04	3.53	A_23_P35277
XBP1	NM_005080	0.00	1.73	3.38	A_23_P120845
FAM134B	NM_001034850	0.65	0.98	3.25	A_24_P266048
KIF12	NM_138424	0.12	1.35	3.19	A_23_P386356
FAM134B	NM_001034850	0.81	0.88	3.13	A_23_P167599
CA12	AK000158	0.08	0.67	3.09	A_23_P163336
CES8	NM_173815	0.75	1.60	3.02	A_23_P361544
ZMAT1	NM_032441	1.17	2.09	2.97	A_24_P11100
LFNG	NM_001040167	-0.62	1.21	2.96	A_23_P8452
FLJ40194	AK097513	1.25	0.51	2.94	A_24_P418152
SYT9	NM_175733	0.90	0.92	2.89	A_24_P255471
PGAP3	NM_033419	0.14	1.63	2.68	A_24_P275828
HPX	NM_000613	-0.42	1.49	2.62	A_23_P161998
CA12	AK000158	0.20	1.01	2.62	A_23_P163338
SIDT1	NM_017699	-2.01	1.76	2.58	A_23_P132515
STARD10	NM_006645	0.23	1.33	2.50	A_23_P36345
DNAH7	NM_018897	-0.10	2.62	2.43	A_23_P33583
MAST4	NM_198828	-0.04	0.50	2.40	A_24_P84340
SLC40A1	NM_014585	0.17	3.01	2.37	A_23_P102391
PRR15	NM_175887	-0.46	1.32	2.31	A_23_P431346
KITLG	NM_000899	-0.47	2.72	2.28	A_23_P204654
CAPN8	NM_001143962	-0.94	2.17	2.27	A_23_P83381
MLPH	NM_024101	-1.63	1.47	2.24	A_23_P165783
INPP4B	BC005273	-0.72	1.40	2.16	A_24_P915492
ABCG1	NM_207627	0.32	2.09	2.13	A_23_P166297
MLPH	NM_024101	-2.19	1.46	2.12	A_23_P165778
PLEKHF2	NM_024613	-0.07	1.10	2.07	A_23_P20275
PNPLA4	NM_004650	-1.07	1.04	2.04	A_24_P943815
FBP1	NM_000507	-1.26	0.49	1.97	A_23_P257111
BCL2	NM_000633	0.11	-1.16	1.90	A_23_P352266
LOC149134	AK022825	-0.15	-0.19	1.90	A_24_P803885
AFF3	NM_002285	-1.04	-1.48	1.89	A_23_P373464
CCDC87	NM_018219	-0.37	1.38	1.88	A_23_P127484
CAPN13	AK074418	-0.72	2.09	1.85	A_23_P345068
SMPD3	NM_018667	-1.32	-1.46	1.85	A_23_P163567
GATA3	NM_001002295	-1.54	-1.21	1.83	A_23_P75056
MLPH	NM_001042467	-1.73	0.97	1.82	A_23_P154400
PRDM6	NM_001136239	-0.30	-0.40	1.81	A_32_P222684
RGS11	NM_003834	-0.58	-0.20	1.76	A_23_P118122
EVL	NM_016337	-0.27	-0.73	1.72	A_23_P140427
CACNA1D	NM_000720	-0.86	1.73	1.72	A_23_P365767
MSL1	BC039449	-0.64	0.28	1.66	A_24_P332837
SLC39A6	NM_012319	-1.01	-0.88	1.66	A_23_P50167
FUT8	NM_178154	-0.80	0.20	1.65	A_23_P313632
GPRC5C	AK000249	-0.09	2.23	1.65	A_23_P346670

TSNAXIP1	NM_018430	-0.20	0.27	1.64	A_23_P129425
SLC39A6	NM_012319	-1.15	-0.86	1.61	A_24_P156049
RASL11B	NM_023940	-0.23	-0.80	1.57	A_23_P69738
CADPS2	NM_017954	-0.42	1.83	1.56	A_24_P246710
DNAJC12	NM_021800	-1.60	-0.59	1.49	A_23_P127220
FGD3	NM_033086	-0.28	-0.81	1.49	A_24_P153840
GPRC5C	AK000249	-1.08	2.55	1.44	A_23_P346673
GNA14	NM_004297	-1.82	1.24	1.44	A_23_P169479
CHRD	NM_003741	-0.82	-0.61	1.43	A_23_P502047
DCLK1	NM_004734	-1.52	-1.62	1.38	A_23_P369994
ACSM1	NM_052956	-1.14	1.80	1.37	A_23_P106933
SPOP	NM_001007226	-0.29	0.14	1.36	A_23_P107257
CALCOCO2	NM_005831	0.08	0.34	1.33	A_23_P4223
C1QTNF3	NM_181435	-0.92	0.07	1.32	A_23_P122067
SPOP	NM_001007226	-0.45	-0.11	1.29	A_24_P270890
DCLK1	NM_004734	-1.01	-0.81	1.29	A_24_P928627
CCND1	NM_053056	-1.07	-0.67	1.28	A_24_P124550
FOXP1	NM_032682	-0.60	0.54	1.28	A_24_P362737
CCND1	NM_053056	-1.75	-1.21	1.26	A_23_P202837
HTR7P	NR_002774	-0.61	-0.19	1.20	A_32_P174572
CIRBP	NR_023312	-0.39	0.12	1.16	A_23_P413456
ERGIC1	NM_001031711	-0.90	1.08	1.14	A_24_P97770
SNX9	NM_016224	-0.04	0.62	1.14	A_24_P179044
HNMT	NM_006895	-0.09	0.36	1.13	A_23_P56734
NUCB2	NM_005013	-0.57	0.07	1.13	A_23_P13364
NPDC1	NM_015392	-1.05	0.14	1.11	A_23_P146572
COL24A1	NM_152890	-1.03	-0.46	1.09	A_23_P74701
DCLK1	NM_004734	-1.80	-2.11	1.09	A_32_P108889
CIRBP	NR_023312	-0.69	-0.12	1.08	A_23_P377616
SSH3	NM_017857	-0.36	1.00	1.06	A_24_P266734
C1QTNF3	NM_181435	-0.85	-0.12	1.04	A_23_P122068
OBFC1	NM_024928	-0.25	0.42	1.04	A_24_P759674
UEVLD	NM_001040697	-0.21	0.50	1.04	A_23_P203445
GLOD5	NM_001080489	-1.44	1.16	1.03	A_23_P95619
CST3	NM_000099	-0.36	-0.25	1.03	A_24_P216294
CCDC30	AY639646	-0.48	0.17	1.02	A_24_P28430
SSH3	NM_017857	-0.32	1.06	1.01	A_23_P150147
NUCB2	AK097398	-1.12	-0.33	1.00	A_24_P595460
KLHDC1	NM_172193	-0.13	0.66	1.00	A_23_P422766
LCA5L	NM_152505	-0.98	1.19	0.98	A_32_P48466
CXXC5	NM_016463	-0.79	-0.34	0.98	A_24_P930062
TANC2	NM_025185	-1.14	0.07	0.97	A_24_P942068
CASC4	NM_138423	-0.30	0.60	0.97	A_23_P140469
CXXC5	NM_016463	-1.00	-0.59	0.96	A_23_P399001
PLIN5	NM_001013706	-0.23	-0.60	0.96	A_24_P272222
TCTN1	NM_024549	-0.24	0.19	0.96	A_23_P76402
C6orf97	NM_025059	-2.59	-0.92	0.96	A_23_P93514
CACNA2D2	NM_001005505	-2.36	-0.82	0.95	A_23_P346900
EXD3	NM_017820	-0.58	0.78	0.94	A_24_P49183
SAR1B	NM_001033503	-0.49	0.05	0.91	A_24_P916845
ELF1	NM_172373	-0.70	0.92	0.91	A_24_P78590
KIAA0556	NM_015202	-0.18	0.88	0.91	A_23_P381203
NAIP	NM_004536	-0.87	0.45	0.91	A_24_P72139

TCEAL6	NM_001006938	-0.39	0.20	0.90	A_32_P192545
C14orf79	NM_174891	-0.49	0.75	0.90	A_23_P420981
MOAP1	NM_022151	-0.36	-0.22	0.88	A_23_P205389
TCEAL3	NM_001006933	-0.43	0.13	0.88	A_23_P434442
ITGB5	NM_002213	-0.74	0.69	0.86	A_23_P166633
LOC646976	AK096082	-1.24	0.00	0.85	A_24_P350546
NUDT4	NM_199040	-0.42	0.68	0.85	A_24_P335263
ZNF597	NM_152457	-1.39	1.17	0.84	A_23_P3753
FSTL4	NM_015082	-1.51	-0.94	0.84	A_24_P266131
CST5	NM_001900	-0.66	-0.50	0.82	A_23_P170453
SEPHS2	NM_012248	-0.66	0.36	0.81	A_23_P146798
EVL	NM_016337	-0.97	-1.33	0.81	A_23_P129038
KCNJ11	NM_000525	-0.77	0.05	0.80	A_23_P1973
BBS1	NM_024649	-0.43	0.74	0.80	A_24_P184305
CCDC74B	NM_207310	-1.20	-0.80	0.79	A_23_P381102
SFXN5	NM_144579	-0.31	0.04	0.79	A_24_P63030
NUDT4	NM_199040	-0.39	0.77	0.78	A_32_P117723
NUDT4	NM_199040	-0.40	0.67	0.78	A_24_P50753
SLC4A8	NM_004858	-1.19	-0.38	0.78	A_24_P75680
GALNT10	NM_198321	-1.15	0.82	0.77	A_23_P7706
C2orf81	NM_001145054	-0.57	-0.12	0.75	A_23_P131449
NEK9	NM_033116	-0.83	0.42	0.75	A_23_P3131
GPRC5C	NM_022036	-1.52	0.93	0.74	A_32_P109029
MED13L	NM_015335	-0.77	-0.96	0.74	A_24_P911508
ITPR1	NM_002222	-0.93	0.77	0.74	A_23_P92042
MBOAT7	NM_024298	-0.81	-0.12	0.73	A_23_P208516
STRN3	NM_014574	-0.77	0.82	0.73	A_23_P65410
RGS11	AK294448	-1.50	-1.10	0.72	A_24_P325118
CCDC74B	NM_207310	-1.35	-0.89	0.71	A_23_P401718
NBEA	NM_015678	-1.39	-1.45	0.71	A_23_P65278
LIMA1	NM_016357	-1.01	0.03	0.71	A_23_P151267
C9orf116	NM_001048265	-0.57	0.67	0.71	A_23_P422115
EXOC6	NM_019053	-0.56	0.19	0.71	A_23_P169576
RALGPS2	NM_152663	-1.66	0.56	0.70	A_24_P173746
RAPGEF3	NM_006105	-0.87	0.90	0.68	A_23_P151307
LOC100133050	NR_027503	-0.68	-0.20	0.67	A_32_P39003
UBXN10	BX648631	-1.52	1.14	0.67	A_32_P205053
NUDT4	NM_199040	-0.88	0.70	0.66	A_24_P67946
SPATA20	NM_022827	-1.04	-0.83	0.66	A_23_P118633
IFT88	NM_175605	-0.41	0.20	0.65	A_23_P48339
C14orf79	NM_174891	-1.06	0.32	0.64	A_23_P376870
KRT18	NM_000224	-1.25	0.27	0.64	A_32_P151544
GPC1	NM_002081	-0.45	-0.50	0.64	A_23_P209904
GUSBP1	NR_027028	-0.73	-0.26	0.63	A_24_P84822
IL6ST	CR621148	-0.87	-0.23	0.63	A_32_P140656
COMMD10	NM_016144	-0.46	-0.43	0.62	A_23_P252403
TANC2	AK021886	-1.17	-0.07	0.62	A_23_P402908
CMBL	NM_138809	-2.12	-0.95	0.62	A_23_P144668
C14orf79	NM_174891	-1.23	0.22	0.62	A_23_P412707
ZNF281	NM_012482	-0.48	0.36	0.61	A_23_P311087
BBS4	NM_033028	-0.64	-0.38	0.60	A_23_P99967
MCCC2	NM_022132	-0.76	0.54	0.60	A_23_P18887
GALNT10	NM_198321	-1.62	0.55	0.59	A_24_P910923

APPL2	NM_018171	-0.48	0.15	0.59	A_23_P105747
CTAGE1	NM_172241	-0.30	1.14	0.59	A_24_P305223
FLJ30901	AK056490	-1.54	-1.41	0.56	A_23_P343104
LEO1	NM_138792	-0.63	0.06	0.56	A_23_P314222
GPR68	NM_003485	-2.14	-2.28	0.55	A_24_P931443
COL4A3BP	NM_001130105	-0.71	0.69	0.55	A_24_P29277
HAGHL	NM_032304	-0.50	-0.68	0.54	A_24_P356373
KRT18	L32537	-1.01	0.22	0.54	A_24_P924957
KAZALD1	AK172864	-1.33	-0.89	0.54	A_24_P192727
SSBP2		-2.13	-2.00	0.53	A_32_P107219
PAX9	NM_006194	-1.88	1.81	0.53	A_32_P70818
SFXN5	NM_144579	-0.87	-0.47	0.52	A_23_P108819
HSPB1	NM_001540	-0.87	-0.36	0.50	A_23_P257704
IER3	NM_003897	-1.31	-0.55	0.49	A_23_P42257
PRR13	NM_001005354	-0.55	0.67	0.49	A_24_P349466
GPRC5C	NM_022036	-1.53	0.76	0.49	A_23_P38167
NR1D1	NM_021724	-1.39	-0.54	0.48	A_24_P250227
HSPB1	NM_001540	-0.92	-0.39	0.48	A_24_P86537
KRR1	NM_007043	-0.70	0.44	0.47	A_32_P326819
KIAA0564	NM_015058	-0.59	0.61	0.46	A_23_P432077
HSPB1	NM_001540	-1.14	-0.43	0.46	A_32_P76247
ETV6	NM_001987	1.56	1.28	0.45	A_23_P105264
C9orf75	NM_173691	-0.83	0.07	0.45	A_23_P323836
RXRA	AK090416	-0.77	-0.28	0.45	A_24_P930985
KRT18	NM_000224	-1.46	0.04	0.45	A_23_P99320
ACADSB	NM_001609	-2.23	0.22	0.44	A_32_P31945
ANKRA2	NM_023039	-0.90	0.45	0.43	A_24_P337397
GRPEL1	AF070525	-0.87	1.00	0.42	A_24_P166045
CDYL2	NM_152342	-1.92	-0.41	0.41	A_23_P371865
KRT18	NM_000224	-1.31	0.01	0.41	A_24_P42136
FLJ40504	NR_028334	-1.58	-0.03	0.40	A_23_P373708
HTR7	NM_019859	-1.36	-0.93	0.39	A_23_P500381
CCND1	NM_053056	-1.80	-1.68	0.38	A_24_P193011
FAM120AOS	AK093641	-0.94	-0.13	0.38	A_32_P21646
ULK1	NM_003565	-0.64	-0.18	0.37	A_24_P73370
CCDC111	NM_152683	-0.57	0.48	0.37	A_23_P358470
C20orf112	AK097804	-1.47	0.83	0.37	A_23_P303548
CIRBP	NR_023312	-0.90	-0.52	0.36	A_23_P312652
SYTL2	NM_032943	-2.08	-0.64	0.35	A_24_P85085
ST3GAL5	NM_003896	-1.04	1.06	0.35	A_23_P136573
NAV1	NM_020443	-1.26	-0.58	0.34	A_24_P102880
PRRT2	NM_145239	-1.38	-2.14	0.33	A_23_P66017
CPEB2	NM_182485	-1.80	-0.54	0.32	A_32_P225355
ARMC9	AB058771	-0.96	-0.28	0.31	A_24_P945408
COL4A3BP	AF136450	-0.74	0.54	0.31	A_23_P61748
PPP1R3C	NM_005398	-2.40	-2.18	0.30	A_23_P35414
PGPEP1	NM_017712	-0.49	0.56	0.30	A_23_P218531
TMEM141	NM_032928	-0.60	0.54	0.30	A_23_P94591
TMEM121	NM_025268	-1.07	-1.68	0.27	A_23_P61987
C3orf62	NM_198562	-0.95	-0.24	0.27	A_32_P831725
GLI3	NM_000168	-1.28	-2.90	0.26	A_23_P111531
TRAF3IP1	NM_015650	-0.53	0.87	0.25	A_23_P5359
LOC442249	XR_019231	-1.63	-0.18	0.24	A_24_P256063

AGGF1	NM_018046	-0.82	-0.33	0.24	A_23_P250554
LOC220429	NR_003268	-0.68	0.70	0.22	A_24_P358054
NR1D1	NM_021724	-1.15	-0.60	0.22	A_23_P420873
AFF4	NM_014423	-0.90	-0.34	0.22	A_24_P394408
ACADSB	NM_001609	-1.59	0.11	0.18	A_23_P158570
TPCN1	NM_001143819	-1.11	0.19	0.17	A_23_P218086
PCSK4	NM_017573	-1.76	-0.91	0.15	A_23_P16648
SLC22A18	NM_183233	-1.27	-0.26	0.15	A_23_P139260
EME2	AK074080	-1.49	0.03	0.15	A_23_P366125
RND1	NM_014470	-1.32	0.54	0.15	A_23_P53370
STRN3	NM_014574	-1.31	0.52	0.09	A_23_P392076
SNAP29	NM_004782	-1.05	0.46	0.08	A_24_P48862
EPB41L5	NM_020909	-1.24	-0.38	0.08	A_24_P944640
RHOH	NM_004310	-2.37	-0.94	0.07	A_23_P58132
SNX25	NM_031953	-0.87	0.23	0.06	A_24_P303097
PAAF1	NM_025155	-0.90	0.13	0.03	A_23_P139339
TMEM192	NM_001100389	-0.99	0.80	0.03	A_23_P253677
ZNF136	NM_003437	-1.05	-0.02	0.03	A_23_P147121
NOL3	NM_003946	-1.24	-1.10	0.02	A_23_P206371
EHMT1	NM_024757	-1.13	-0.19	0.01	A_23_P20894
LPPR2	NM_022737	-1.39	-0.20	0.00	A_23_P153461
GAMT	NM_138924	-1.15	-0.82	-0.01	A_24_P19228
LOC254057	AK024653	-2.35	-0.68	-0.03	A_24_P450092
MEIS3	NM_001009813	-1.36	-1.19	-0.03	A_24_P207503
EPOR	NM_000121	-1.21	-0.49	-0.03	A_23_P367899
ST3GAL5	NM_003896	-1.57	0.95	-0.04	A_23_P311869
MEIS3	NM_001009813	-1.74	-1.39	-0.04	A_23_P78795
TM7SF2	NM_003273	-1.64	0.20	-0.04	A_23_P116037
DPP7	NM_013379	-1.21	-0.41	-0.04	A_23_P32975
FRMD6	NM_001042481	-1.27	-1.95	-0.08	A_24_P330303
CPEB2	NM_182485	-1.66	-0.90	-0.08	A_24_P340390
NPC1L1	NM_013389	-2.00	-1.97	-0.08	A_23_P20075
TPCN1	NM_001143819	-1.18	-0.05	-0.09	A_24_P244575
PLCD4	NM_032726	-2.82	-1.94	-0.10	A_23_P385105
ADSSL1	NM_199165	-1.79	-1.00	-0.10	A_23_P76823
TTC8	NM_144596	-1.45	-0.90	-0.11	A_32_P169735
TMEM192	NM_001100389	-1.16	0.65	-0.17	A_24_P20524
EPOR	NM_000121	-1.34	-0.70	-0.17	A_23_P381954
C19orf51	NM_178837	-1.86	-0.92	-0.19	A_23_P335486
MTHFR	NM_005957	-1.12	-0.01	-0.22	A_23_P400078
KIAA1683	NM_025249	-1.60	-1.47	-0.28	A_23_P130974
ANKRD36BP1	NR_026844	1.39	0.75	-0.29	A_23_P97123
POLK	NM_016218	-1.63	-0.84	-0.38	A_24_P303160
GAMT	NM_000156	-2.23	-1.54	-0.41	A_23_P108143
ZSWIM5	NM_020883	-1.93	-1.92	-0.44	A_23_P383118
THPO	NM_000460	-1.79	-2.26	-0.54	A_23_P121459
DAK	NM_015533	-1.77	-0.38	-0.57	A_23_P36129
GATA2	NM_032638	-2.51	-1.24	-0.63	A_24_P165998
POLK	NM_016218	-1.85	-1.28	-0.67	A_23_P386450
GATA2	NM_032638	-3.10	-1.11	-0.68	A_23_P110022
TCTN1	BC030993	-2.39	-2.15	-0.80	A_24_P282274
GSR	BC035691	-2.62	-0.09	-0.84	A_32_P31618
MALAT1	NR_002819	-2.32	-1.72	-0.96	A_24_P873659

SLC27A2	NM_003645	-3.43	-2.16	-1.06	A_23_P140450
RHBG	NM_020407	-2.50	-2.27	-1.27	A_23_P51690
