# Supplementary material for: Integrated Genomic and Epigenomic Analysis of Breast Cancer Brain Metastasis
Source: PLoS One. 2014 Jan 29;9(1):e85448. doi: 10.1371/journal.pone.0085448 (PMC3906004; doi:10.1371/journal.pone.0085448)
Supplement: File S1 — Supporting figures and tables. Figure S1: Combined Network for Upstream Analysis of FOXM1 and TBX2. The downstream genes connected to FOXM1 and TBX2 were illustrated as a network in IPA. The mRNA expression ratios are listed below the gene nodes. The legend within figure describes the node and edge color keys. Figure S2: Word Cloud Analysis of Cluster Enrichments. We have used word clouds to visually summarize the textual results from the enrichment analysis of each gene cluster as observed in Figure 3. The results were generated using www.wordle.net web resource. The larger the word, the more times it is mentioned in the enrichment categories. Supplementary Tables in File S1. Table S1a. Table S1b. Table S2. Table S3a. Table S3b. Table S4a. Figure S1. Table S4b. Table S5a–b. Table S6a–b. Table S7. Table S8a–f. Table S9a–f. Figure S2. Table S10. Table S11a–c. Table S11d. Table S12. Table S13. Table S14. (ZIP) [file pone.0085448.s001.zip › Supplementary Table S8c.pdf]

Supplementary Table 8c. List of Cluster 3 genes on heatmap

(See Figure 3 in main text). Values represent normalized Log2 ratios.

Gene Symbol	GenBank Accession	Basal-like	Her2+/ER-ve	Luminal B	ProbeName
OGFRL1	NM_024576	2.15	0.42	0.06	A_23_P7791
AKR1E2	AB040820	2.15	1.01	0.10	A_23_P1322
FOXC1	NM_001453	2.03	-1.08	-0.57	A_32_P205110
SLC6A2	NM_001043	2.02	-0.46	0.00	A_23_P358345
ITGB8	NM_002214	2.01	-0.08	0.03	A_23_P123060
FOXC1	NM_001453	1.88	-1.41	-0.96	A_23_P390504
PRICKLE1	NM_153026	1.84	-0.23	-0.24	A_23_P408285
SLC6A2	NM_001172504	1.77	0.48	0.15	A_24_P910660
MLLT4	BC014505	1.66	0.57	-0.01	A_23_P344694
RASD2	NM_014310	1.65	0.02	-0.21	A_24_P357100
FOXL1	NM_005250	1.64	-0.07	0.19	A_23_P365081
PELI1	NM_020651	1.60	1.20	0.25	A_23_P120345
C22orf23	AK097339	1.58	0.20	-0.50	A_24_P941407
NRG2	NM_013982	1.54	-1.59	-1.17	A_23_P213699
FZD7	NM_003507	1.52	-1.36	-1.11	A_23_P209449
ITGB8	NM_002214	1.51	-0.69	-1.05	A_24_P759477
BPI	NM_001725	1.48	-0.01	-0.24	A_23_P131789
KHDC1	NM_030568	1.47	-0.12	0.17	A_24_P280762
ZNF462	NM_021224	1.44	-1.05	-0.78	A_23_P60499
HLA-DOB	NM_002120	1.44	0.17	-0.39	A_23_P30736
FAM83B	NM_001010872	1.32	-1.35	-0.77	A_24_P66780
VRK2	NM_006296	1.31	0.79	0.19	A_23_P119992
TBX19	NM_005149	1.29	0.15	-0.04	A_23_P137705
PTPN14	NM_005401	1.28	0.22	-1.09	A_23_P149111
GCNT2	NM_001491	1.28	-0.22	-1.10	A_24_P397489
NRG2	NM_004883	1.23	-0.29	-0.60	A_23_P349857
MPZL2	NM_144765	1.23	0.84	-1.32	A_23_P150379
FAM171A1	NM_001010924	1.21	-0.32	-1.01	A_23_P44964
SPEG	AK055387	1.20	-0.92	-0.22	A_23_P338919
TMEM65	NM_194291	1.16	0.29	-0.34	A_24_P256583
PPP2R3A	NM_002718	1.13	1.52	-0.42	A_23_P10401
TIFA	NM_052864	1.12	0.27	0.05	A_24_P350686
PPP1CB	NM_002709	1.11	0.10	0.42	A_24_P396720
PPPDE1	NM_016076	1.07	0.24	-0.09	A_23_P201445
CHST3	NM_004273	1.06	0.07	-0.41	A_24_P269779
SRF	NM_003131	1.04	0.36	0.04	A_24_P346277
LRP12	NM_013437	1.03	-0.06	-0.71	A_23_P8906

ATL2	NM_022374	1.03	0.09	-0.30	A_23_P209619
SPSB1	NM_025106	1.00	1.04	-0.38	A_23_P200096
ECE2	NM_032331	1.00	0.51	-0.36	A_23_P92261
ITGB8	NM_002214	1.00	-0.62	-1.48	A_24_P273599
USP6NL	NM_014688	0.98	0.33	-0.36	A_23_P138426
SPSB1	NM_025106	0.97	0.79	-0.14	A_24_P96961
PPP2R3A	NM_002718	0.93	1.27	-0.36	A_24_P388433
ZNF318	NM_014345	0.91	-0.33	-0.21	A_23_P145175
OSR1	NM_145260	0.88	-0.22	-1.13	A_23_P323272
ILF2	NM_004515	0.86	-0.02	-0.31	A_23_P257956
MRPL14	NM_032111	0.86	0.01	-0.33	A_23_P124035
BYSL	NM_004053	0.85	-0.11	-0.44	A_23_P145197
MED30	NM_080651	0.85	0.13	-0.59	A_23_P31866
UBASH3B	NM_032873	0.84	-0.37	-1.36	A_24_P192933
SIN3B	BC025026	0.84	-0.06	-0.89	A_24_P93901
TRDMT1	NM_004412	0.83	-0.85	-1.27	A_24_P252705
URB2	NM_014777	0.82	0.02	-0.40	A_23_P74914
CLIC4	NM_013943	0.80	-0.63	-0.18	A_23_P259189
PTK7	NM_002821	0.78	0.36	-0.76	A_24_P320545
C21orf91	NM_017447	0.78	-0.53	-0.92	A_23_P211015
FLJ25694	AK127969	0.78	-0.69	-0.66	A_23_P342751
FAM36A	NM_198076	0.75	-0.01	-0.34	A_32_P106646
KLHL18	BC015962	0.75	-0.54	-0.94	A_24_P916686
XPO5	NM_020750	0.75	-0.60	-0.49	A_23_P256855
LRP12	NM_013437	0.74	-0.09	-1.15	A_24_P415012
ZNF232	NM_014519	0.74	-0.34	-0.12	A_23_P4294
LIN9	NM_173083	0.73	-0.48	-0.35	A_32_P233304
POLH	NM_006502	0.72	-0.43	-0.81	A_32_P69492
NUDCD1	NM_032869	0.71	0.07	-0.39	A_23_P123343
TTLL4	NM_014640	0.69	-0.57	-1.11	A_23_P142697
C15orf41	NM_032499	0.68	-0.24	-0.61	A_23_P77201
TRDMT1	NM_004412	0.67	-1.44	-1.53	A_23_P115636
ATP11C	NM_001010986	0.66	-0.24	-0.86	A_23_P251730
GPSM2	NM_013296	0.65	-0.29	-0.48	A_23_P63402
RIOK1	NM_153005	0.65	-0.37	-0.44	A_24_P8088
MICALL1	NM_033386	0.65	0.13	-1.05	A_23_P68922
CLIP4	NM_024692	0.64	-0.14	-1.24	A_23_P209232
NFIL3	NM_005384	0.63	-0.85	-0.77	A_23_P32253
CERK	NM_022766	0.61	0.05	-1.16	A_23_P211659
FLJ40330	NR_015424	0.58	-0.19	-0.64	A_24_P341089
WWTR1	NM_015472	0.57	0.26	-1.55	A_23_P29769
ATP11C	NM_173694	0.57	-0.14	-0.66	A_24_P399942
UBE2E3	NM_006357	0.57	-0.55	-1.17	A_24_P148450

USP31	NM_020718	0.57	-0.57	-0.37	A_24_P390583
NUDT5	NM_014142	0.56	-0.35	-0.62	A_23_P1199
WWTR1	NM_015472	0.55	0.77	-2.13	A_32_P6868
ZNF507	NM_014910	0.52	-0.20	-0.56	A_23_P208812
TMEM38A	NM_024074	0.51	-0.44	-0.91	A_23_P101392
UCK2	NM_012474	0.50	-0.76	-0.70	A_23_P487
SEH1L	NM_031216	0.49	-0.36	-0.74	A_24_P407930
USP1	NM_003368	0.47	-1.00	-0.61	A_23_P11652
UBE2E3	NM_006357	0.47	-0.80	-1.78	A_23_P11192
LIN9	NM_173083	0.45	-0.46	-0.55	A_23_P301995
TMEM123	NM_052932	0.45	0.06	-0.94	A_24_P309415
CREB3L2	BC063666	0.42	0.15	-1.04	A_24_P180654
GNB4	NM_021629	0.36	-1.08	-1.02	A_32_P184916
GTPBP4	NM_012341	0.36	-0.50	-0.78	A_23_P12874
FAF1	NM_007051	0.35	-0.70	-0.34	A_23_P96853
EIF2C2	NM_012154	0.30	-0.30	-0.81	A_23_P112159
PPP2R5D	NM_180976	0.28	-0.73	-0.64	A_24_P294931
KIF1C	BC040307	0.28	-0.58	-0.75	A_32_P149288
FNDC3B	BC012204	0.27	0.92	-1.30	A_24_P919304
POLR2F	NM_021974	0.24	-0.11	-0.92	A_23_P80321
TMEM123	NM_052932	0.23	0.00	-1.07	A_23_P202964
TBPL1	NM_004865	0.08	-0.42	-0.97	A_23_P168276
JRKL	NM_003772	0.01	0.48	-1.68	A_23_P202737
AURKB	NM_004217	-0.62	-1.57	-1.83	A_23_P130182
CCNA2	NM_001237	-0.75	-1.79	-2.24	A_23_P58321
MTHFD1L	NM_015440	-0.83	-1.66	-2.09	A_23_P214907
