# Supplementary material for: Integrated Genomic and Epigenomic Analysis of Breast Cancer Brain Metastasis
Source: PLoS One. 2014 Jan 29;9(1):e85448. doi: 10.1371/journal.pone.0085448 (PMC3906004; doi:10.1371/journal.pone.0085448)
Supplement: File S1 — Supporting figures and tables. Figure S1: Combined Network for Upstream Analysis of FOXM1 and TBX2. The downstream genes connected to FOXM1 and TBX2 were illustrated as a network in IPA. The mRNA expression ratios are listed below the gene nodes. The legend within figure describes the node and edge color keys. Figure S2: Word Cloud Analysis of Cluster Enrichments. We have used word clouds to visually summarize the textual results from the enrichment analysis of each gene cluster as observed in Figure 3. The results were generated using www.wordle.net web resource. The larger the word, the more times it is mentioned in the enrichment categories. Supplementary Tables in File S1. Table S1a. Table S1b. Table S2. Table S3a. Table S3b. Table S4a. Figure S1. Table S4b. Table S5a–b. Table S6a–b. Table S7. Table S8a–f. Table S9a–f. Figure S2. Table S10. Table S11a–c. Table S11d. Table S12. Table S13. Table S14. (ZIP) [file pone.0085448.s001.zip › Supplementary Table S8d.pdf]

Supplementary Table 8d. List of Cluster 4 genes on heatmap

(See Figure 3 in main text). Values represent normalized Log2 ratios.

Gene Symbol	GenBank Accession	Basal-like	Her2+/ER-ve	Luminal B	ProbeName
B3GNT5	NM_032047	2.44	0.52	-0.80	A_23_P18372
ANXA8L2	NM_001630	2.40	-2.17	-1.86	A_32_P105549
CYP39A1	NM_016593	2.31	-0.26	-0.47	A_23_P133712
KRT16P2	NR_029392	2.25	-1.08	-1.45	A_32_P62963
BCL11A	NM_018014	2.23	-0.44	-2.05	A_24_P402588
L3MBTL4	NM_173464	2.20	-0.03	-1.54	A_23_P326474
PLEKHG4B	NM_052909	2.14	-0.51	-0.77	A_32_P129269
KRT16	NM_005557	2.02	-1.84	-2.53	A_24_P392991
STAC	NM_003149	1.69	-1.90	-3.16	A_24_P234415
STAC	NM_003149	1.65	-1.40	-2.17	A_23_P121061
TMSB15B	NM_194324	1.60	-2.29	-0.63	A_23_P96599
KRT14	NM_000526	1.57	-0.63	-1.87	A_24_P265346
BCL11A	NM_022893	1.51	-1.77	-3.74	A_24_P411186
PLEKHG4B	NM_052909	1.51	-0.39	-0.16	A_23_P81640
CCDC67	NM_181645	1.40	0.43	-1.39	A_32_P153954
ZNF238	NM_006352	1.37	2.39	-0.29	A_24_P299663
BCL11A	NM_022893	1.34	-0.67	-2.04	A_23_P218584
WNT6	NM_006522	1.22	-1.96	-2.42	A_23_P119916
SFRS13B	NM_080743	1.20	-0.59	-2.51	A_23_P110903
LY6K	NM_017527	1.15	-2.08	-1.92	A_23_P397285
HIF3A	NM_022462	1.12	0.05	-1.97	A_23_P374339
LOC643650	BC033221	1.08	-1.29	-1.19	A_23_P359214
TCF7L1	NM_031283	1.05	-2.47	-1.92	A_23_P142872
IL17RD	NM_017563	1.03	-0.34	-1.44	A_32_P188860
KRT17	NM_000422	0.93	-2.79	-2.73	A_23_P96158
TRIM29	NM_012101	0.89	-1.47	-2.77	A_23_P203267
C11orf75	NM_020179	0.87	1.88	-0.46	A_23_P75430
ZNF238	NM_006352	0.84	1.11	-1.01	A_23_P200512
KRT16P3	NR_029393	0.69	-0.69	-2.36	A_32_P168973
KIF1B	NM_183416	0.69	-0.80	-1.19	A_24_P649624
BTG3	NM_006806	0.65	-0.18	-1.80	A_23_P80068
LOC401317	BC087859	0.62	-1.37	-1.00	A_32_P219135
PTPN14	NM_005401	0.61	-0.94	-1.08	A_24_P2648
NRTN	NM_004558	0.60	-1.68	-1.70	A_23_P90359
LOC729088	CR602569	0.59	-1.03	-0.60	A_32_P36143
CAPN6	NM_014289	0.57	-1.78	-2.03	A_23_P217570
C6orf173	NM_001012507	0.57	-1.38	-1.59	A_24_P462899

PM20D2	NM_001010853	0.55	-1.33	-2.64	A_32_P86118
OBSCN	NM_052843	0.54	-0.93	-0.70	A_24_P119685
PDE7A	NM_002603	0.52	-0.87	-0.74	A_23_P123478
PHGDH	NM_006623	0.52	-1.71	-1.66	A_23_P85783
SLC27A6	NM_001017372	0.51	-1.96	-2.95	A_23_P41789
TOP1MT	NM_052963	0.50	-0.37	-0.97	A_24_P248053
HSPC159	NM_014181	0.48	-0.18	-1.18	A_23_P430818
C6orf173	NM_001012507	0.47	-1.52	-1.77	A_32_P143245
CSDA	NM_003651	0.43	-0.20	-1.10	A_23_P25224
CKS1B	NM_001826	0.40	-0.99	-0.95	A_32_P192430
CNTNAP3	NM_033655	0.38	-1.86	-2.55	A_23_P9135
SLC25A37	AF495725	0.37	-0.79	-1.56	A_24_P64100
OSBPL3	NM_015550	0.36	-0.73	-1.47	A_23_P215525
WNT11	NM_004626	0.35	-2.58	-1.61	A_24_P253003
NDC80	NM_006101	0.34	-1.42	-1.32	A_24_P14156
GPR161	NM_153832	0.34	-0.94	-1.09	A_23_P354314
CKS1B	NM_001826	0.33	-1.02	-0.92	A_23_P45917
SEPHS1	BC064610	0.32	-1.01	-0.54	A_24_P90022
SLC19A3	NM_025243	0.31	-1.82	-2.25	A_23_P39871
ANP32E	NM_030920	0.31	-2.02	-1.70	A_24_P225468
TMEM74	NM_153015	0.28	-1.37	-2.34	A_23_P390139
CHAC2	NM_001008708	0.27	-0.32	-1.44	A_32_P194264
CRABP1	NM_004378	0.21	-1.58	-1.60	A_23_P117882
OSBPL3	NM_015550	0.21	-0.63	-2.19	A_24_P599340
NFE2L3	NM_004289	0.20	-1.50	-2.08	A_23_P42718
BOP1	NM_015201	0.20	-0.68	-1.26	A_23_P43800
PGM1	NM_002633	0.18	-0.64	-0.94	A_23_P52031
KIF1B	NM_183416	0.17	-1.01	-1.40	A_24_P145066
LOC729683	CR594811	0.16	-1.40	-0.72	A_32_P145010
TEAD4	NM_003213	0.16	-0.19	-1.12	A_23_P94795
RPS27A	NM_002954	0.16	-0.58	-0.97	A_32_P24581
CSDA	NM_003651	0.15	-0.46	-1.40	A_24_P625382
DMD	NM_004019	0.15	-0.79	-1.44	A_24_P342388
BTG3	BC028229	0.13	-1.16	-2.16	A_32_P33723
SEH1L	NM_031216	0.10	-0.54	-1.36	A_23_P78311
SUV39H2	NM_024670	0.09	-0.84	-1.35	A_23_P202392
LRRC42	NM_052940	0.09	-0.64	-0.95	A_23_P51278
CDCA3	NM_031299	0.08	-0.87	-1.16	A_23_P162476
FBL	NM_001436	0.08	-0.90	-1.02	A_23_P78888
SRPK1	NM_003137	0.08	-0.77	-1.21	A_23_P19543
MRPL37	NM_016491	0.08	-0.83	-1.02	A_23_P135474
OSBPL3	NM_015550	0.08	-1.51	-2.39	A_24_P377499
CTSL2	NM_001333	0.08	-1.45	-1.94	A_23_P146456

FAM123B	NM_152424	0.06	-0.87	-1.18	A_23_P308150
STMN1	NM_203401	0.05	-1.62	-0.83	A_23_P200866
FZD9	NM_003508	0.04	-1.32	-1.06	A_23_P59613
NDC80	NM_006101	0.04	-1.40	-1.34	A_23_P50108
TTK	NM_003318	0.03	-0.84	-1.72	A_23_P259586
FAM64A	NM_019013	0.02	-1.77	-1.53	A_23_P49878
LBR	NM_002296	0.01	-1.60	-1.65	A_23_P200493
GEMIN4	NM_015721	0.01	-1.19	-1.05	A_23_P66872
FKBP1A	NM_054014	0.00	-0.41	-1.69	A_23_P397238
ZNF286A	AF086305	0.00	-2.00	-2.26	A_24_P910833
YBX1	NM_004559	-0.01	-1.00	-1.03	A_24_P375002
HEATR1	NM_018072	-0.01	-0.95	-1.08	A_23_P103628
AGBL5	NM_001035507	-0.01	-1.00	-1.39	A_23_P154466
ANP32E	NM_030920	-0.02	-2.10	-1.91	A_23_P160934
CENPA	NM_001809	-0.02	-1.11	-1.51	A_24_P413884
MYO10	NM_012334	-0.02	-0.36	-1.24	A_24_P46357
FOXM1	NM_202002	-0.03	-0.95	-1.77	A_23_P151150
CENPF	NM_016343	-0.03	-1.47	-1.34	A_23_P401
C15orf23	NM_001142761	-0.03	-1.01	-1.23	A_23_P140705
CKS1B	NM_001826	-0.03	-1.00	-1.06	A_32_P206698
FBL	NM_001436	-0.04	-1.13	-1.29	A_23_P78892
FANCE	NM_021922	-0.04	-1.49	-1.50	A_23_P42335
DEK	NM_003472	-0.04	-1.27	-1.42	A_23_P254702
C1orf163	NM_023077	-0.04	-1.20	-1.18	A_23_P347508
QKI	NM_006775	-0.04	-1.14	-1.71	A_24_P941322
YBX1	NM_004559	-0.05	-1.11	-1.07	A_24_P101391
NMT2	NM_004808	-0.05	-1.68	-1.25	A_23_P138686
MTSS1L	NM_138383	-0.07	-1.14	-1.12	A_32_P84084
GMPS	NM_003875	-0.07	-0.42	-1.23	A_23_P21033
FBXO31	AK026130	-0.08	-1.29	-1.17	A_23_P89030
LOC645195	AK123450	-0.08	-1.30	-1.24	A_32_P224234
C9orf40	NM_017998	-0.10	-0.97	-1.15	A_23_P43425
E2F2	NM_004091	-0.11	-1.18	-1.02	A_23_P408955
CDCA7L	NM_018719	-0.11	-0.42	-1.65	A_24_P274795
GEMIN4	NM_015721	-0.11	-1.15	-1.23	A_23_P66867
ANKS6	NM_173551	-0.11	-0.96	-1.80	A_23_P362183
PRPF38A	NM_032864	-0.13	-1.17	-0.81	A_24_P97001
SERBP1	NM_001018067	-0.13	-1.02	-1.19	A_23_P359111
DNAH14	NM_001145154	-0.15	-1.37	-1.56	A_32_P87531
NFE2L3	NM_004289	-0.15	-1.46	-2.63	A_24_P136653
MSH2	NM_000251	-0.18	-0.88	-1.18	A_23_P102471
TPX2	NM_012112	-0.19	-1.35	-1.33	A_23_P68610
CIRH1A	NM_032830	-0.21	-1.23	-1.07	A_24_P25346

MCM10	NM_182751	-0.22	-1.60	-1.78	A_24_P412088
YBX1	NM_004559	-0.22	-1.34	-1.37	A_23_P34767
MRPL2	NM_015950	-0.22	-0.83	-1.58	A_23_P7941
KIF20A	NM_005733	-0.24	-1.46	-1.09	A_23_P256956
EIF5A2	NM_020390	-0.25	-1.41	-1.76	A_24_P385739
PTPLA	NM_014241	-0.25	-2.78	-2.34	A_23_P161352
MAGOH	NM_002370	-0.25	-1.83	-1.85	A_23_P200216
ANKRD36	NM_001164315	-0.26	-1.10	-1.79	A_24_P336931
LOC100128355	XM_002343797	-0.26	-1.06	-1.37	A_24_P204474
YBX1	NM_004559	-0.27	-1.36	-1.43	A_32_P218989
RAD51AP1	NM_006479	-0.28	-0.44	-1.67	A_23_P99292
MCM10	NM_182751	-0.29	-1.42	-1.77	A_23_P161474
SH2D2A	NM_003975	-0.29	-1.33	-1.76	A_23_P160618
PSMG1	NM_003720	-0.30	-1.21	-1.38	A_23_P68717
KIFC1	NM_002263	-0.30	-1.89	-1.59	A_23_P133956
MAP7D3	NM_024597	-0.30	-1.31	-1.19	A_24_P177631
REXO2	NM_015523	-0.30	-0.44	-1.37	A_23_P150365
CDCA8	NM_018101	-0.31	-1.75	-1.94	A_23_P375
TEX10	NM_017746	-0.31	-0.78	-1.40	A_23_P112412
DNAH14	NM_144989	-0.31	-1.44	-1.74	A_23_P333951
CDCA2	NM_152562	-0.32	-2.04	-2.19	A_23_P385861
GCSH	NM_004483	-0.36	-1.41	-1.54	A_23_P117933
CDC20	NM_001255	-0.38	-1.82	-1.76	A_23_P149200
SKA3	BC013418	-0.38	-1.30	-1.62	A_23_P340909
CTPS	NM_001905	-0.41	-1.36	-1.78	A_23_P21706
ZCCHC11	NM_001009881	-0.42	-1.92	-1.96	A_23_P34433
C3orf26	NM_032359	-0.42	-1.41	-1.61	A_23_P132874
PTTG1	NM_004219	-0.42	-1.47	-1.54	A_23_P7636
C15orf42	NM_152259	-0.43	-1.40	-1.88	A_23_P345707
CCNB2	NM_004701	-0.44	-1.74	-1.56	A_23_P65757
LMNB2	NM_032737	-0.44	-1.16	-1.44	A_23_P67725
FAM27E3	BC119675	-0.45	-0.48	-2.14	A_23_P348979
ASS1	NM_000050	-0.46	0.23	-2.17	A_23_P31921
C2orf3	EF158467	-0.46	-1.09	-1.52	A_24_P51037
NCAPD2	NM_014865	-0.46	-1.50	-1.68	A_23_P25293
MTHFD1L	AY374131	-0.47	-1.45	-1.55	A_23_P214908
KIF2C	NM_006845	-0.48	-1.79	-1.75	A_23_P34788
CDKN2C	NM_078626	-0.49	-2.08	-1.58	A_23_P85460
ASPM	NM_018136	-0.54	-1.90	-1.93	A_23_P52017
PPPDE1	BC020640	-0.54	-1.43	-2.60	A_24_P922808
REXO2	NM_015523	-0.54	-0.67	-1.57	A_24_P316364
DIAPH3	NM_001042517	-0.57	-2.27	-2.10	A_32_P150891
CKS2	NM_001827	-0.58	-1.91	-1.78	A_23_P71727

C15orf42	NM_152259	-0.63	-1.53	-1.95	A_32_P109296
DIAPH3	NM_030932	-0.64	-2.12	-2.07	A_23_P419254
FANCI	NM_018193	-0.66	-1.73	-2.02	A_24_P902509
ACSS2	NM_018677	-0.68	-0.96	-2.12	A_24_P156295
CDCA5	NM_080668	-0.68	-1.41	-1.93	A_23_P104651
HPDL	NM_032756	-0.70	-2.10	-2.83	A_23_P74449
TYMS	NM_001071	-0.70	-2.24	-2.15	A_23_P50096
GSG2	AK056691	-0.71	-1.73	-2.06	A_24_P76521
C9orf40	NM_017998	-0.72	-2.16	-2.82	A_24_P43876
ADAT2	NM_182503	-0.72	-1.01	-1.93	A_24_P186204
NT5DC2	NM_022908	-0.76	-2.09	-2.39	A_23_P44836
DLGAP5	NM_014750	-0.77	-1.97	-1.63	A_23_P88331
ARHGAP11A	NM_014783	-0.79	-2.38	-2.11	A_23_P136805
ASPM	NM_018136	-0.81	-2.23	-2.27	A_24_P911179
RAD54L	NM_003579	-0.82	-1.95	-2.11	A_23_P74115
AMOTL1	NM_130847	-0.83	-0.89	-2.40	A_24_P329815
OIP5	NM_007280	-0.86	-2.20	-2.10	A_23_P379614
MYO19	NM_001033580	-0.87	-0.94	-1.89	A_23_P100868
ADAT2	NM_182503	-0.88	-1.65	-2.48	A_23_P134014
KIF18B	BC048263	-0.88	-2.01	-2.26	A_24_P680947
ZW10	NM_004724	-0.92	-0.87	-2.00	A_23_P64204
PIM1	NM_002648	-0.92	-2.35	-2.37	A_23_P345118
CENPN	NM_018455	-0.93	-2.37	-2.14	A_23_P88740
AMOTL1	NM_130847	-0.93	-1.01	-2.18	A_23_P138796
LOC149351	BC036441	-0.97	-2.36	-2.98	A_24_P520767
LOC100293193	XR_079078	-1.00	-0.99	-2.63	A_24_P235520
PSAT1	NM_058179	-1.00	-1.31	-2.84	A_23_P259692
LOC100293193	XR_079078	-1.02	-1.08	-3.12	A_32_P112623
CHEK1	NM_001274	-1.07	-1.89	-2.60	A_23_P116123
SLC25A37	AF113696	-1.18	-2.47	-2.78	A_23_P216004
FSCN1	NM_003088	-1.21	-2.44	-2.24	A_23_P168531
SKP2	NM_032637	-1.32	-2.43	-2.53	A_23_P156310
SPC25	NM_020675	-1.34	-2.59	-2.42	A_23_P51085
CDC45L	NM_003504	-1.42	-2.23	-2.77	A_23_P57379
KRT34	NM_021013	-1.63	-3.40	-3.14	A_23_P101054
CASC5	NM_170589	-2.06	-2.45	-3.18	A_24_P378331
