# Supplementary material for: Integrated Genomic and Epigenomic Analysis of Breast Cancer Brain Metastasis
Source: PLoS One. 2014 Jan 29;9(1):e85448. doi: 10.1371/journal.pone.0085448 (PMC3906004; doi:10.1371/journal.pone.0085448)
Supplement: File S1 — Supporting figures and tables. Figure S1: Combined Network for Upstream Analysis of FOXM1 and TBX2. The downstream genes connected to FOXM1 and TBX2 were illustrated as a network in IPA. The mRNA expression ratios are listed below the gene nodes. The legend within figure describes the node and edge color keys. Figure S2: Word Cloud Analysis of Cluster Enrichments. We have used word clouds to visually summarize the textual results from the enrichment analysis of each gene cluster as observed in Figure 3. The results were generated using www.wordle.net web resource. The larger the word, the more times it is mentioned in the enrichment categories. Supplementary Tables in File S1. Table S1a. Table S1b. Table S2. Table S3a. Table S3b. Table S4a. Figure S1. Table S4b. Table S5a–b. Table S6a–b. Table S7. Table S8a–f. Table S9a–f. Figure S2. Table S10. Table S11a–c. Table S11d. Table S12. Table S13. Table S14. (ZIP) [file pone.0085448.s001.zip › Supplementary Table S8e.pdf]

Supplementary Table 8e. List of Cluster 5 genes on heatmap

(See Figure 3 in main text). Values represent normalized Log2 ratios.

Gene Symbol	GenBank Accession	Basal-like	Her2+/ER-ve	Luminal B	ProbeName
TFCP2L1	NM_014553	6.03	3.88	2.83	A_23_P5301
KRT6B	NM_005555	6.02	-0.31	0.58	A_23_P76249
ELF5	NM_198381	5.86	1.33	1.98	A_24_P227141
RARRES1	NM_002888	5.86	2.77	0.29	A_23_P18078
DSC3	NM_024423	5.40	0.60	0.34	A_24_P344416
ELF5	NM_198381	5.00	0.38	1.24	A_23_P13465
C8orf85	NM_001025357	4.74	0.93	1.99	A_32_P181527
KRT6A	NM_005554	4.69	2.64	1.87	A_23_P87653
EN1	NM_001426	4.66	-1.18	-0.89	A_23_P56404
GABRP	NM_014211	4.63	0.54	0.34	A_23_P328545
KRT6C	NM_173086	4.45	1.31	1.34	A_23_P366936
KRT5	NM_000424	4.35	0.81	0.47	A_23_P218047
SLC34A2	NM_006424	4.30	4.00	0.51	A_23_P133036
C5orf46	NM_206966	4.19	-0.11	0.82	A_23_P19176
GSDMC	NM_031415	4.14	3.36	1.37	A_23_P60120
VGLL1	NM_016267	4.07	2.26	0.29	A_23_P253123
GPRIN2	AB011086	3.96	1.78	1.11	A_23_P343382
DSC2	NM_024422	3.89	1.80	1.16	A_23_P4494
SAA1	NM_000331	3.85	1.75	0.16	A_24_P335092
HIST1H1A	NM_005325	3.70	-0.62	-0.88	A_23_P70448
LGALS7B	NM_001042507	3.63	0.63	0.10	A_23_P108062
DSC3	NM_024423	3.57	0.48	0.26	A_23_P208029
ANXA8L2	BC008813	3.52	-0.27	-0.02	A_23_P395054
SAA2	NM_030754	3.50	1.93	0.68	A_23_P306203
KRT16	NM_005557	3.50	-0.74	-0.99	A_23_P38537
C6orf15	NM_014070	3.47	0.42	0.18	A_24_P72364
UGT8	AL137342	3.35	0.31	-0.68	A_24_P942589
ATP6V1C2	NM_001039362	3.31	1.86	-0.55	A_23_P250914
IL12RB2	NM_001559	3.26	-0.01	0.04	A_23_P72077
UGT8	NM_003360	3.21	0.14	-0.26	A_24_P103264
SOSTDC1	NM_015464	3.16	-1.37	-2.56	A_23_P145841
LGALS7B	NM_001042507	3.14	0.68	0.68	A_24_P348118
LGALS7	NM_002307	3.04	0.53	0.49	A_24_P238250
UGT8	U62899	3.03	0.09	-0.45	A_23_P72747
ART3	NM_001179	2.94	-1.50	-1.64	A_23_P80918
CNGA1	NM_000087	2.93	1.62	0.55	A_24_P256722
KRT14	NM_000526	2.93	1.11	1.51	A_23_P4335
CHODL	NM_024944	2.91	0.90	-0.45	A_23_P68669

C4orf7	NM_152997	2.87	-0.37	-1.02	A_23_P362694
CDH3	NM_001793	2.87	1.89	0.45	A_23_P49155
PROM1	NM_006017	2.80	-0.78	-2.92	A_23_P258463
C5orf23	NM_024563	2.78	-1.46	-0.71	A_23_P58676
HORMAD1	NM_032132	2.75	-1.64	-2.98	A_32_P199884
RDH10	NM_172037	2.71	2.15	0.38	A_24_P261032
STRA8	NM_182489	2.71	0.79	0.45	A_24_P203308
LAD1	NM_005558	2.63	2.56	0.79	A_23_P415510
LMO4	NM_006769	2.49	-0.32	0.92	A_23_P380181
RGMA	NM_020211	2.45	0.47	0.03	A_23_P372308
TAGAP	NM_138810	2.32	0.29	0.52	A_23_P339588
EXPH5	NM_015065	2.31	3.19	1.27	A_23_P403335
NTN1	NM_004822	2.29	1.90	0.67	A_32_P53524
C1orf198	NM_032800	2.24	0.99	0.79	A_32_P42574
RDH10	NM_172037	2.22	1.49	-0.21	A_32_P25050
EXPH5	NM_015065	2.21	2.83	0.92	A_24_P937691
KRTAP6-3	NM_181605	2.12	0.93	1.21	A_24_P391604
CYB5R2	NM_016229	1.98	-0.26	0.36	A_23_P2181
FGFBP1	NM_005130	1.90	-1.14	-3.24	A_23_P30126
NPR3	NM_000908	1.77	-1.85	-1.76	A_23_P327451
NPR3	NM_000908	1.65	-1.85	-1.70	A_23_P253536
LEMD1	NM_001001552	1.63	-0.54	-3.99	A_24_P696761
SLPI	NM_003064	1.53	2.52	-1.57	A_23_P91230
SLPI	NM_003064	1.51	2.63	-1.97	A_24_P190472
