# Supplementary material for: Integrated Genomic and Epigenomic Analysis of Breast Cancer Brain Metastasis
Source: PLoS One. 2014 Jan 29;9(1):e85448. doi: 10.1371/journal.pone.0085448 (PMC3906004; doi:10.1371/journal.pone.0085448)
Supplement: File S1 — Supporting figures and tables. Figure S1: Combined Network for Upstream Analysis of FOXM1 and TBX2. The downstream genes connected to FOXM1 and TBX2 were illustrated as a network in IPA. The mRNA expression ratios are listed below the gene nodes. The legend within figure describes the node and edge color keys. Figure S2: Word Cloud Analysis of Cluster Enrichments. We have used word clouds to visually summarize the textual results from the enrichment analysis of each gene cluster as observed in Figure 3. The results were generated using www.wordle.net web resource. The larger the word, the more times it is mentioned in the enrichment categories. Supplementary Tables in File S1. Table S1a. Table S1b. Table S2. Table S3a. Table S3b. Table S4a. Figure S1. Table S4b. Table S5a–b. Table S6a–b. Table S7. Table S8a–f. Table S9a–f. Figure S2. Table S10. Table S11a–c. Table S11d. Table S12. Table S13. Table S14. (ZIP) [file pone.0085448.s001.zip › Supplementary Table S8f.pdf]

Supplementary Table 8f. List of Cluster 6 genes on heamap

(See Figure 3 in main text). Values represent normalized Log2 ratios.

Gene Symbol	GenBank Accession	Basal-like	Her2+/ER-ve	Luminal B	ProbeName
DACH1	NM_080759	0.61	4.10	6.74	A_23_P32577
ANKRD30A	NM_052997	0.52	3.68	5.90	A_23_P12533
CEACAM6	BC005008	-0.20	4.35	5.77	A_23_P421483
TFF3	NM_003226	-1.16	2.44	5.61	A_24_P245778
TFF3	NM_003226	-1.89	2.54	5.33	A_23_P257296
TFF3	NM_003226	-2.37	2.34	5.25	A_23_P393099
LOC145837	NR_026979	0.08	3.27	5.18	A_32_P46594
CEACAM7	NM_006890	0.98	4.23	4.95	A_24_P228302
CCDC48	NM_024768	1.64	3.32	4.67	A_23_P166566
AGR3	NM_176813	-3.09	-0.17	4.58	A_23_P42811
ESR1	NM_000125	-0.68	1.30	4.41	A_23_P309739
SLC44A4	NM_025257	-1.24	4.30	4.36	A_23_P93349
C20orf26	NM_015585	1.27	2.47	4.34	A_32_P4262
FLT3	NM_004119	0.93	0.91	4.22	A_23_P99442
PRR15	NM_175887	-0.90	2.59	4.06	A_32_P154911
CAPN13	NM_144575	-0.02	4.27	4.03	A_23_P101972
C20orf114	NM_033197	0.77	0.83	3.99	A_23_P154784
TBC1D9	NM_015130	-0.89	1.50	3.93	A_23_P41487
C2orf55	NM_207362	2.34	3.79	3.88	A_24_P272313
SLC44A4	NM_025257	-1.16	3.76	3.87	A_24_P684183
GP2	NM_001007240	-0.29	1.26	3.86	A_24_P190007
AGR2	NM_006408	-4.68	1.25	3.83	A_23_P31407
DNALI1	NM_003462	0.73	2.69	3.81	A_23_P160377
TSPAN1	NM_005727	0.60	2.58	3.79	A_23_P160167
CAPN13	NM_144575	0.78	4.25	3.75	A_24_P336983
LOC440335	NR_029454	1.13	3.89	3.69	A_24_P229884
AR	NM_000044	-1.58	1.52	3.60	A_23_P113111
FOXA1	NM_004496	-2.59	1.80	3.58	A_24_P347431
TFF3	NM_003226	-0.84	2.23	3.55	A_24_P289208
FAM47E	NM_001136570	0.88	2.94	3.49	A_32_P83811
CTSO	NM_001334	1.77	4.26	3.43	A_23_P110175
FOXA1	NM_004496	-2.78	1.71	3.41	A_23_P37127
RSPH1	NM_080860	0.98	2.61	3.38	A_23_P102950
GALNT6	NM_007210	0.23	2.43	3.28	A_23_P204133
PARP9	NM_031458	2.01	3.57	3.24	A_23_P69383
SPAG17	NM_206996	1.08	3.37	3.19	A_23_P319783
DYNLRB2	NM_130897	-1.03	2.01	3.19	A_23_P94840
C14orf25	BC038110	-1.04	2.03	3.13	A_24_P393596

LRFN2	NM_020737	-0.15	2.23	3.07	A_32_P82111
EFCAB6	NM_022785	0.80	2.77	3.07	A_23_P68978
C17orf28	NM_030630	0.57	3.00	2.99	A_23_P118412
SPEF2	NM_024867	0.64	2.93	2.94	A_32_P179396
LRRC6	NM_012472	0.90	2.81	2.87	A_23_P112004
ABCC11	NM_033151	-0.33	2.66	2.82	A_23_P141076
RBM47	NM_019027	0.91	3.36	2.79	A_23_P132910
FLJ38379	AK095698	-0.19	2.55	2.78	A_24_P194661
KLHDC9	NM_001007255	0.91	2.49	2.72	A_23_P86100
KIAA1244	NM_020340	-0.07	2.58	2.67	A_32_P188186
SLC2A10	NM_030777	0.04	2.15	2.65	A_24_P271323
TMC5	NM_024780	-1.57	4.14	2.62	A_23_P15101
TMEM86A	NM_153347	0.75	3.10	2.54	A_32_P66035
FAM84B	NM_174911	1.17	3.29	2.46	A_24_P329487
EPB41L5	BC032822	0.94	2.77	2.15	A_23_P209298
RAB27B	NM_004163	-0.62	2.62	1.83	A_23_P107612
